# Seismic prediction of shale oil lithofacies associations based on sedimentary facies patterns: A case study of the shahejie formation in the Huanghekou Sag

**DOI:** 10.1371/journal.pone.0332314

**Published:** 2025-09-18

**Authors:** He Zhao, Hongliang Wang, Guangyuan Wang, Lin Wan

**Affiliations:** 1 School of Energy Resources, China University of Geosciences, Beijing, China; 2 Key Laboratory of Marine Reservoir Evolution and Hydrocarbon Enrichment Mechanism, China University of Geosciences, Beijing, China; 3 China National Offshore Oil Corporation, Tianjin Branch, Tianjin, China; Sichuan University of Science and Engineering, CHINA

## Abstract

The lithofacies play a pivotal role in studying development patterns, reservoir characteristics, and sweet spot predictions of shale oil. Lithofacies classification typically relies on core observations and conventional well logging analyses, whereas seismic attribute extraction is often employed in regions with sparse or absent wells. However, seismic attribute extraction entails considerable computation and time, and exclusive reliance on seismic attribute analysis can result in multiple interpretations. This paper emphasizes predicting shale oil lithofacies associations based on seismic reflection characteristics and sedimentary facies patterns which can can help avoid these issues. The lithofacies classification scheme has identified seven lithofacies and associations by means of core observations, testing data, and logging curve analysis of the Shahejie Formation in the Huanghekou Sag. Through well-seismic calibration, the seismic reflections and sedimentary patterns of different lithofacies associations were examined to formulate a seismic facies identification chart and propose six models. For areas without wells, based on the distribution of sedimentary facies and in combination with seismic reflection characteristics, identification and delineation are conducted on a planar scale to analyze the distribution features of lithofacies associations. The results of predicting the distribution of shale oil lithofacies associations in the Shahejie Formation indicate that the development pattern of lithofacies associations is basically consistent with that of sedimentary facies units. The primary models developed in the study area encompass delta, sublacustrine fan, and shore-shallow lake. The approach of identifying shale oil lithofacies associations based on seismic reflection and sedimentary backgrounds offers a novel means for discerning lithofacies and associations in sections devoid of cores and specialized well logging data.

## Introduction

The continental shale formations possess relatively favorable geological conditions in China, providing a geological foundation for the large-scale development of continental shale oil resources [1]. Compared to conventional oil and gas, shale oil lacks conventional cap rocks, traps, and migration processes, making the control of lithofacies particularly prominent [2]. The classification of shale oil lithofacies plays a crucial role in studying the development patterns, reservoir characteristics, and sweet spot predictions of shale oil. Currently, the classification of shale oil lithofacies generally relies on core observations, well logging analyses, and experimental test data. Based on rock composition, sedimentary structures, organic matter abundance, and other factors, the Paleogene shale in the Jiyang Depression has been divided into three categories and sixteen types of lithofacies: laminar, layered, and massive [3]. Lithofacies classification is also conducted according to the vertical characteristics of the depositional paleoenvironment [4–5]. Additionally, shale lithofacies assemblages are classified based on factors influencing the formation of fracture networks during shale fracturing [6]. By studying the types of shale lithofacies and their assemblages, analyzing the genesis of major lithofacies assemblages, and clarifying the developmental and distribution characteristics of shale lithofacies assemblages, further insights can be gained [7].

For shale oil lithofacies classification in areas with no wells or few wells, utilizing seismic attribute analysis is an effective method in geological research [8]. Commonly used attributes include root mean square amplitude, instantaneous amplitude, instantaneous frequency, instantaneous phase, among others [9]. By integrating seismic data with drilling information and based on depositional parameters represented by seismic attributes, the lithofacies are characterized through neural network fusion of sensitive depositional parameters. Sensitive attributes are selected from seismic attributes to improve prediction accuracy [10–11]. However, the choice of seismic attribute extraction algorithms has a significant impact on the results, requiring repeated deliberation and comparison to find the optimal parameter associations, which involves substantial computational effort and time consumption. Purely applying seismic attribute analysis leads to multiple solutions, where identical seismic attributes may correspond to various geological interpretations, increasing prediction uncertainty.

This study innovatively establishes a seismic identification methodology for shale oil lithofacies assemblages based on sedimentary pattern constraints. With lithofacies analysis as the core, the petrological characteristics of shale oil formations are systematically characterized through detailed core description, whole-rock X-ray diffraction analysis, and organic geochemical testing. A rational classification scheme for shale oil reservoir lithofacies is proposed using the “mineral content - bedding characteristics - organic matter content” approach. Fisher discriminant analysis is employed to develop quantitative identification models of logging responses for different lithofacies. Through well-seismic calibration technology, the study pioneers the construction of a seismic reflection characteristic identification chart for lithofacies assemblages, enabling planar distribution prediction of lithofacies combinations.

The innovation of this technology lies in the organic integration of lithofacies units with sedimentary facies units, effectively reducing the ambiguity of seismic interpretation through sedimentary background constraints. It provides a reliable lithofacies identification solution for intervals lacking core data and specialized logging. The research results offer significant guidance for predicting favorable lithofacies zones and optimizing exploration and development targets in continental shale oil systems.

## Data and method

**Fig 1:**
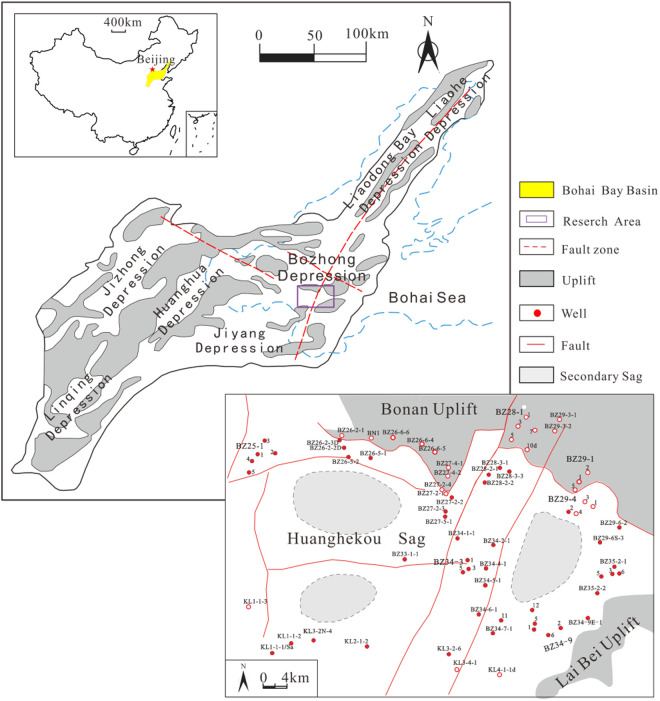
Geological and structural of the Huanghekou Sag (Modified from “Map of China”, Map Review Number: GS(2023)2767 and “Map of China’s Topography”, Map Review Number: GS(2016)1609; Source: China Standard Map Service - http://bzdt.ch.mnr.gov.cn/).

The Huanghekou Sag is located in the southeast of the Bohai Bay Basin, with a length of 70 km to 80 km from east to west and a width of 40 to 50 km from north to south, covering an area of approximately 3, 600 km². The basement depth is about 7 km (Fig 1) [12]. As of 2020, 16 oil and gas fields have been discovered in the Huanghekou Sag, making it one of the important oil and gas producing areas in the Bohai Bay Basin [13–14]. It is a hydrocarbon-rich sag that has undergone multiple cycles of water depth changes, possessing geological conditions conducive to the development of multiple sets of source rocks [15–17]. From bottom to top, four main sets of source rocks are primarily developed: the fourth Member of the Shahejie Formation (E_2_s_4_) and Kongdian Formation (E_1_k), the third Member of the Shahejie Formation (E_2_s_3_), the first Member of the Shahejie Formation (E_2_s_1_), the third Member of the Dongying Formation (E_3_d_3_), with thickness centers mainly located within the northwest sub-sag, southwest sub-sag, and eastern sag; dark mudstones are mainly distributed in the middle of E_2_s_3_, E_2_s_1_, and E_3_d_3_ [18]. Among them, the source rocks of E_2_s_3_ and E_2_s_1_ are characterized by good organic matter types and high thermal evolution degrees, making them the most favorable source rocks.

Currently, the drilling wells in the Huanghekou Sag are mainly distributed around the sag’s periphery and within the central uplift belt, mostly for conventional reservoir drilling, while shale oil is mainly developed in the center. In response to the current situation of few wells in shale oil horizons but abundant seismic data in the Huanghekou Sag, this study conducts seismic prediction analysis of shale oil lithofacies associations based on facies patterns.

### Data

In this study, approximately 130 meters of observed cores were in wells B-2, B-3, B-5, and B-21. There were 22 shale core samples collected with the depth from 3412.0m to 3426.6m in well B-2, 3546.5m to 3658.4m in well B-3, 3542.0m to 3547.81m in well B-5, and 3090.5m to 3092.0m in well B-21. The experimental data included 62 XRD data and 28 TOC data. Logging data from 32 wells were utilized, such as GR, DT, NPHI, SP, LLD, and RHOB, which are relatively sensitive to lithology. Additionally, the seismic data covering an area of approximately was 3, 000 km².

### Method

(1) Based on core observations and test data analysis, a lithofacies classification scheme was established to determine the types of lithofacies developed.
(2) Through lithological-electrical analysis, Fisher discriminant analysis was conducted using SPSS software. Single-well lithofacies classification was carried out based on well logging curve data to determine the types of lithofacies assemblages in individual wells.
(3) Landmark software was used for well-to-seismic calibration. Based on key properties such as external reflection structures, internal reflection characteristics, amplitude, frequency, and continuity of different lithofacies assemblages, a seismic identification chart for lithofacies assemblages was established.
(4) By applying the seismic identification method for shale oil lithofacies associations based on depositional facies, interpretation and prediction of shale oil were conducted, and a plan-view distribution map of lithofacies associations in the study area was drawn.

## Results and discussion

### Determination of shale oil lithofacies and its associations

**Fig 2:**
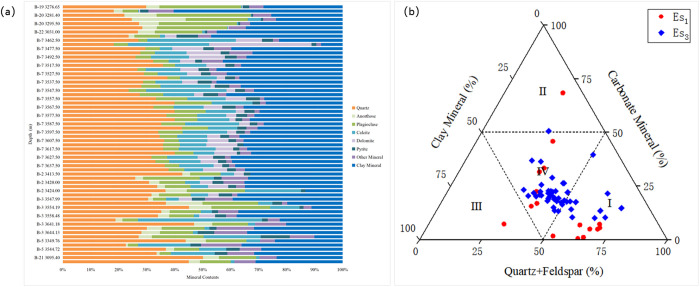
Mineral composition of the Shahejie Formation in Huanghekou Sag (62 samples) (a. Mineral composition of the sample, b.Ternary diagram of shale; I Quartz-Feldspar dominated shale,II Carbonate dominated shale, III Clay dominated shale, IV Mixed mineral shale).

The lithofacies of shale oil are classified based on mineral content, bedding structure, and organic matter content. X-ray diffraction analysis reveals that the study area is predominantly characterized by quartz and clay. The content of quartz is from 18.20% to 50.10%, with an average of 34.81% and the content of clay is from from 10.10% to 39.30%, with an average of 28.52%. Secondary minerals include potassium feldspar, plagioclase, calcite, dolomite, and pyrite (Fig 2).

**Fig 3:**
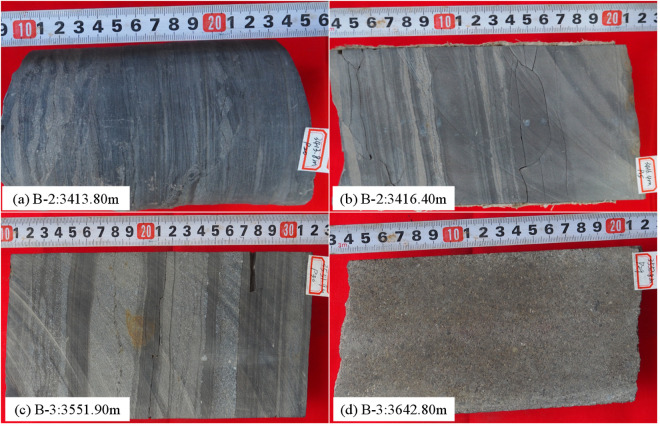
The main bedding types of the Shahejie Formation in Huanghekou Sag (a. Layered clay dominated shale; b. Layered quartz-feldspar dominated shale; c. Layered mixed mineral shale; d. Massive sandstone).

The primary lithofacies are quartz-feldspar dominated shale and mixed mineral shale. Based on core observations with visual standards, layers thicker than 10 mm are classified as massive structures, while those thinner than 10 mm are classified as layered structures (Fig 3) [19–21].

**Fig 4:**
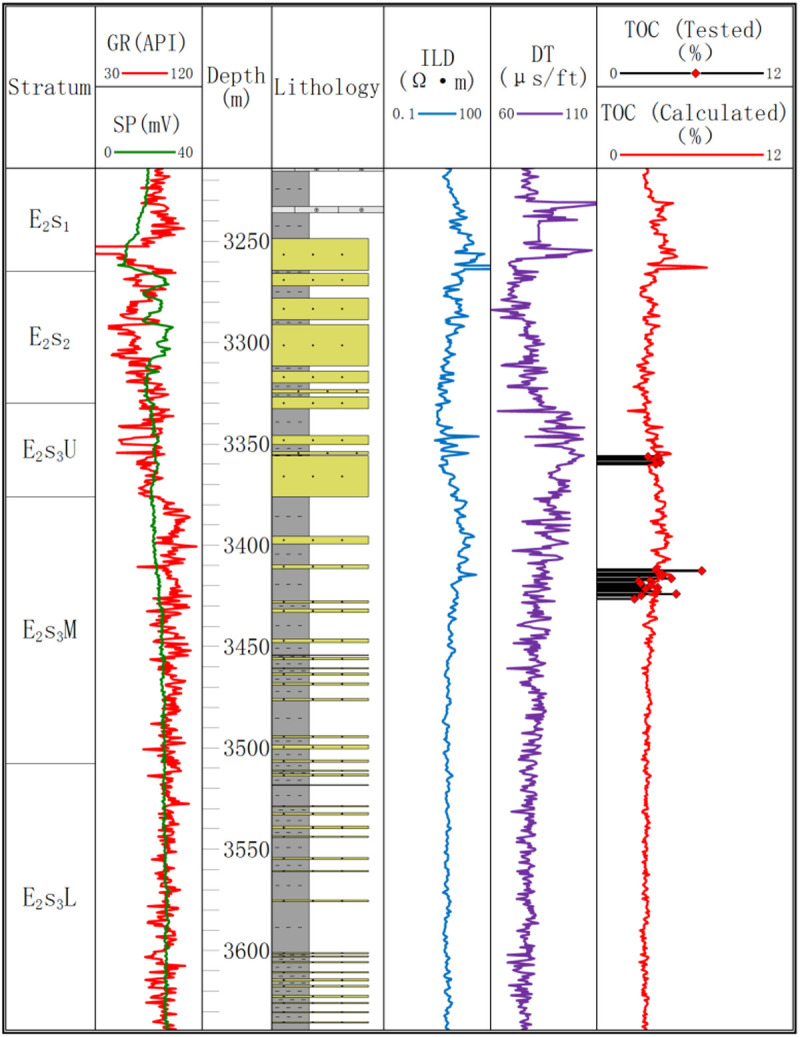
The measured and fitted TOC content of Well B-2.

The total organic carbon content is categorized into high organic matter with TOC ≥ 2% and low organic matter with TOC < 2%. The measured TOC of the samples ranges from 2% to 8.97%, with an average content of 4.07%. All mudstone intervals belong to high organic matter layers. According to ∆log R calculations, the proportion of high organic matter exceeds approximately 95% (Fig 4).

**Fig 5:**
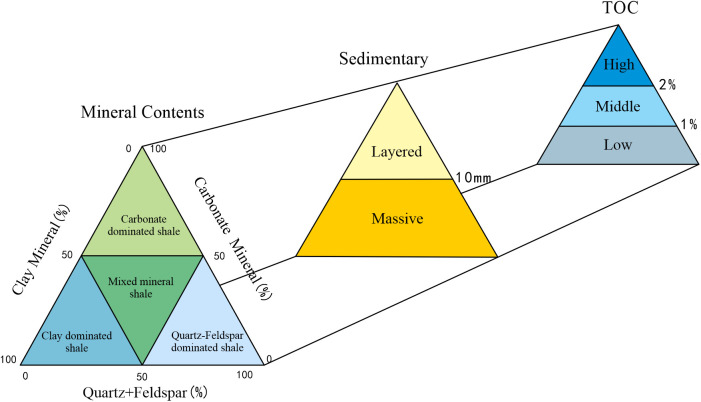
Lithofacies division scheme of the Shahejie Formation in Huanghekou Sag.

Considering the characteristics of the samples in the study area, a suitable classification scheme tailored to the study area is developed (Fig 5).

**Fig 6:**
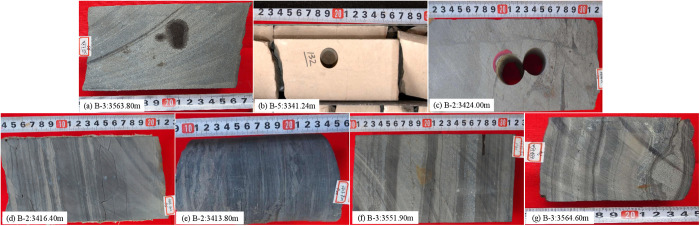
The main lithofacies types of the Shahejie Formation in the Huanghekou Sag (a. High-organic layered limestone; b. Massive thick sandstone; c. Massive thin sandstone; d. High-organic layered quartz-feldspar dominated shale; e. High-organic layered clay dominated shale; f. High-organic layered mixed mineral shale; g. High-organic layered carbonate dominated shale).

The Shahejie Formation in the Huanghekou Sag mainly identifies seven lithofacies:

(1) High-organic layered limestone: Primarily distributed at the top of E_2_s_3_, it mainly consists of oolitic limestone, limestone, and dolomite (Fig 6a).
(2) Massive thick sandstone: Primarily distributed in E_2_s_2_ at wells B-2 and B-5, it has a light gray to gray color and is mainly composed of coarse to medium-grained siltstone (Fig 6b).
(3) Massive thin sandstone: Primarily distributed in E_2_s_3_, it has a thickness of 1.0m to 2.5m, a light gray to gray color, and is mainly composed of siltstone, with visible mudstone tear fragments (Fig 6c).
(4) High-organic layered quartz-feldspar dominated shale: Primarily distributed in the middle section of E_2_s_3_ at well B-3 and the lower section of E_2_s_3_ at well B-5, it appears gray to dark gray in core observations. The thickness of single layer is generally 1mm to 10mm. Microscopic observations reveal a large number of white quartz grains (Fig 6d).
(5) High-organic layered clay dominated shale: Primarily distributed near the maximum flooding surface in the middle section of E_2_s_3_, TOC generally ranging from 3.54% to 5.66% with an average of 4.26%. Core observations show a gray to dark gray color, well-developed bedding, and a single layer thickness generally ranging from 1mm to 10 mm. The main clay mineral is illite (Fig 6e).
(6) High-organic layered mixed mineral shale: Primarily distributed in the lower section of E_2_s_3_ at well B-1 and the middle to lower sections at well B-2, it appears gray and exhibits wavy bedding, representing a transitional stage between quartz-feldspar dominated shale and aclay dominated shale (Fig 6f).
(7) High-organic layered carbonate dominated shale: Primarily distributed in E_2_s_1_ at well B-5, with a light gray to gray color, it produces a small amount of bubbles when dropped with hydrochloric acid (Fig 6g).

**Fig 7:**
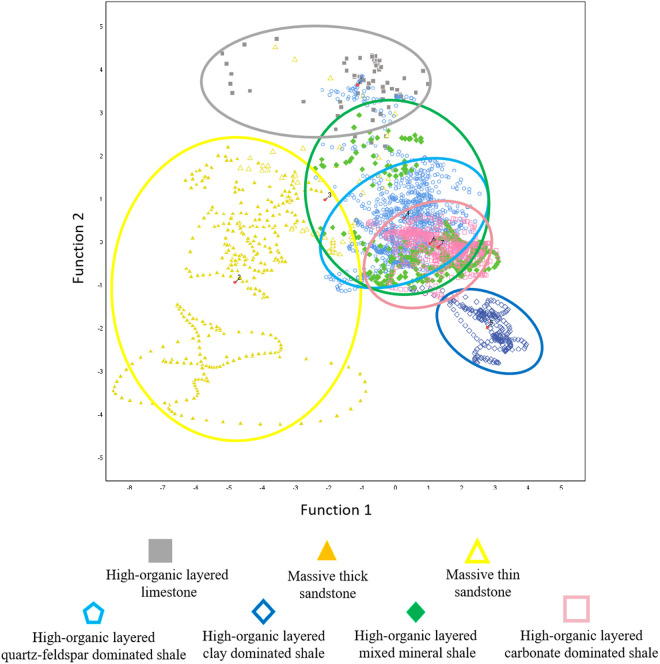
Fisher discriminant analysis data scatter plot.

Logging curves can reflect different sedimentary evolutions and lithofacies characteristics [22–24]. Analysis of the commonly used logging data in the study area reveals the following: The GR values for mudstone and shale intervals concentrate between 80API and 120API, while those for sandstone and limestone are lower, ranging from 60API to 85API; the SP values for mudstone and shale intervals are concentrated between 20mV and 45mV, with limestone exhibiting lower SP values, while sandstone intervals show a broader distribution range for SP; resistivity and compensated density values are higher in sandstone and limestone intervals compared to mudstone and shale, while acoustic time difference and compensated neutron values are overall lower in mudstone and shale intervals. Establishing logging response patterns for different shale lithofacies, Fisher discriminant scatter plots indicate that different lithofacies have distinct clustering centers (Fig 7).

**Fig 8:**
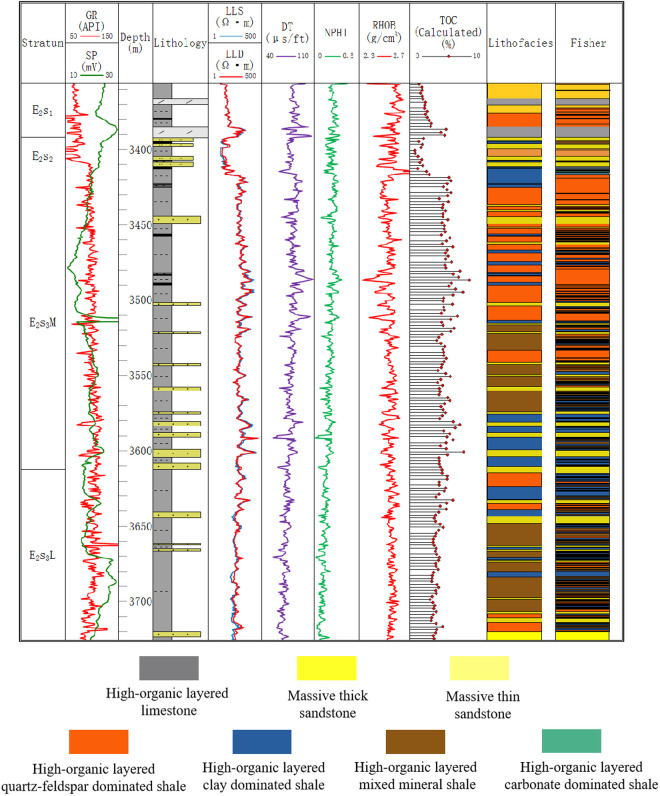
Application of lithofacies discriminant analysis in Well B-3.

Based on the discriminant equation group, calculations were performed for the entire well section, and the lithofacies corresponding to the equation with the maximum Y value was selected as the lithofacies determined by discriminant analysis, which was then verified through well sections with more complete data. Well B-3 is dominated by high-organic layered quartz-feldspar dominated shale, mixed mineral shale, and massive thin sandstone. The lithofacies identification results obtained by discriminant analysis are more detailed (Fig 8). The sandstone in the middle section of E_2_s_3_ is subdivided into multiple thin sandstone layers, which corresponds to the tooth-like characteristics presented by GR, reflecting the characteristics of intercalated shale oil and better showcasing the location of shale oil reservoirs. Both core identification methods and Fisher discriminant methods reflect the same lithofacies change process from bottom to top in Well B-3: from mixed mineral shale to clay dominated shale, then back to mixed mineral shale, and finally transitioning to quartz-feldspar dominated shale. The clay dominated shale intervals identified by discriminant analysis are thinner with the depth from 3460m to 3470m and 3570m to 3610m, further demonstrating the advantage of discriminant analysis in distinguishing subtle sedimentary features. The lithofacies prediction rate using Fisher discriminant analysis exceeds 80%, indicating that identifying lithofacies through logging curve characteristics is feasible and can be applied in lithofacies prediction for well sections lacking core data, laying the foundation for subsequent well-seismic integration analysis.

**Fig 9:**
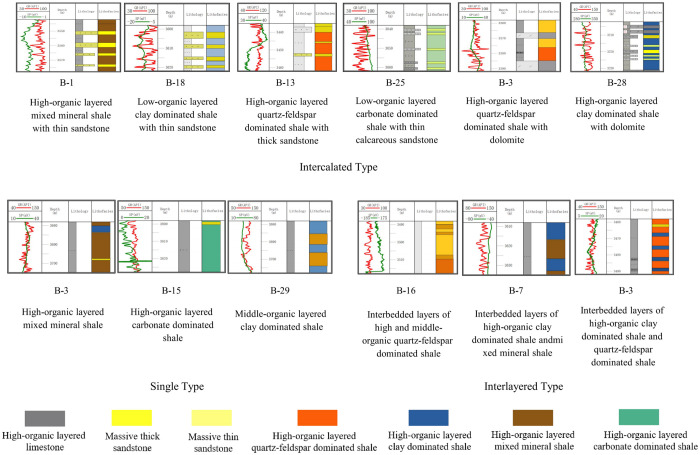
Lithofacies associations division of the Shahejie Formation in the Huanghekou Sag.

The depositional environment serves as the primary basis for classifying lithofacies and their associations types. Factors such as climate, provenance, lake water medium properties, and water depth control the development and distribution of fine-grained sedimentary rocks, including various types of mudstone and shale in semi-deep to deep lake environments [25–27]. The research on the classification of shale Lithofacies associations requires both theoretical support and geological significance, aiming to divide shale formations into genetically meaningful stratigraphic units that can be identified through well logging [28–31]. In the Shahejie Formation shale formations of the Huanghekou Sag, the Lithofacies associations can be classified into three major categories: single type, interlayered type and intercalated type (Fig 9).

### Seismic response characteristics of lithofacies associations

**Fig 10:**
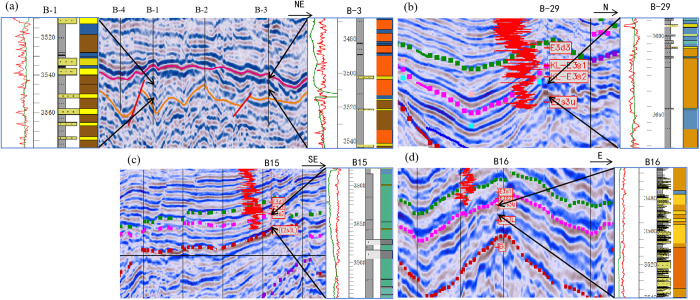
Typical lithofacies associations by the data of well-seismic (a. Mixed mineral shale and quartz-feldspar shale with thin sandstone, Distal part of lake-bottom fan; b. Interbedded layers of clay shale and mixed mineral shale, Semi-deep and deep lake; c. Carbonate shale with thin calcareous sandstone, Mixed shoal-bar platform; d. Quartz-feldspar shale, Pre-delta).

**Fig 11:**
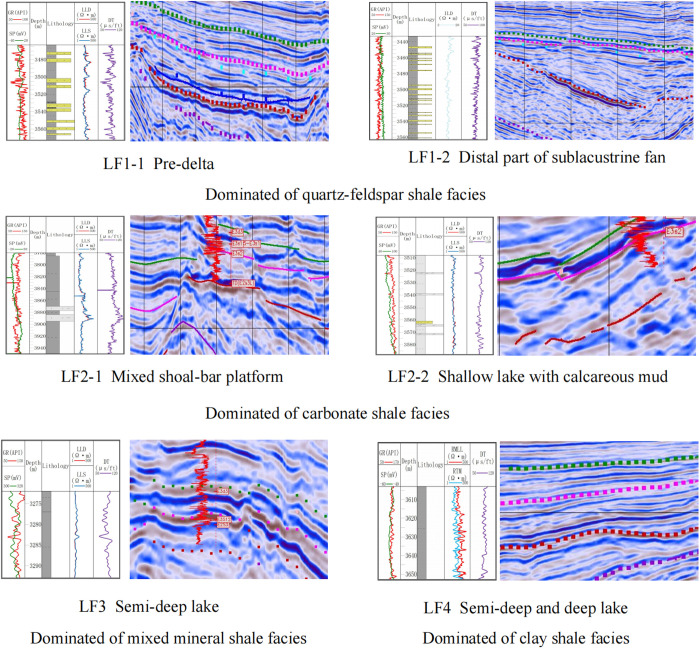
Seismic identification chart of lithofacies associations.

By comparing the seismic reflection characteristics between conventional sandstone and shale oil facies zones, it utilizes amplitude and frequency to predict the distribution range of the main fine-grained sedimentary shale oil facies zones. The amplitude-intensity relationship can partially reflect the difference between shale oil and sandstone; sandstone bodies generally exhibit high-angle, progradational weak reflection axes; mudstone shows continuous weak reflections with multiple visible parallel axes [32–36]. For the shale oil layer series in the Huanghekou Sag, it conducted well-seismic calibration in areas with wells to identify the characteristics of lithofacies associations (Fig 10), established a seismic lithofacies associations identification chart (Fig 11), and analyzed the seismic reflection characteristics of different shale oil.

There are six main seismic reflection characteristics of lithofacies associations identified in the Huanghekou Sag.

The lithofacies associations dominated of quartz-feldspar shale have two manifestations. The first one represented in pre-delta with the serial number LF1–1, often developed in the distal areas of progradational reflections. It exhibits wedge-shaped to sheet-shaped external features with medium to strong amplitude, low frequency, and moderate continuity. Internally, it presents parallel to subparallel patterns. The second one called LF1–2 develops in the distal part of a subaqueous fan with chaotic reflections at the base. It appears as hill-shaped and lenticular-shaped with low-angle reflections at the fan tip. It has medium to strong amplitude, low frequency, and moderate continuity.

There are also two manifestations dominated by carbonate shale. The first one named LF2−1 represents a mixed shoal-bar platform and often developed on underwater uplifts. It exhibits wavy reflections with medium to strong amplitude, low to medium frequency, and good continuity. The second, LF2−2, represents shallow lake with calcareous mud, characterized by medium amplitude, low to medium frequency, and good continuity, too.

The lithofacies associations LF-3, dominated by mixed mineral shale, is mainly developed in the semi-deep lake, serving as a transitional stage between quartz-feldspar and clay shale. It exhibits sheet-shaped or plate-shaped external features with medium to strong amplitude, medium to strong frequency, and fairly good continuity in parallel reflections.

The lithofacies associations LF-4, dominated by clay shale, is mainly developed in the semi-deep to deep lake, occurring in the center of the depression. Its morphology is similar to that of mixed shale, exhibiting sheet-shaped and flat-shaped external features with low to medium amplitude, low to medium frequency, and fairly good continuity in parallel reflections.

### Identification of lithofacies associations based on sedimentary facies

**Fig 12:**
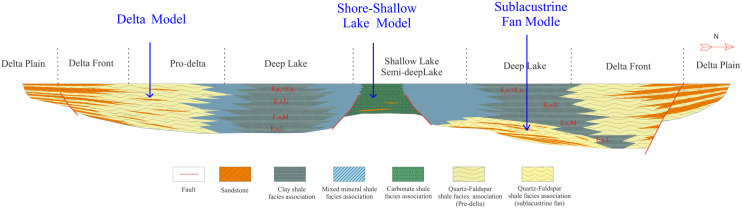
Shale oil lithofacies development model of the Shahejie Formation in the Huanghekou sag.

Seismic waveforms, representing the geometrical characteristics of seismic events in seismic data, encompass diverse information related to sequences, structures, and reservoir characteristics. Based on seismic facies identification charts, a method for identifying lithofacies associations considering the sedimentary background is proposed. The superposition relationships among lithofacies associations reflect different sedimentary environments. Three main patterns are developed in the study area (Fig 12).

(1) Delta Model (LF1-1→LF3→LF4)

The fan delta front in the study area developed on the downthrown side of the northern boundary fault of the depression, forming a progradational fan delta system characterized by interbedded sandstones and mudstones, with well-developed underwater distributary channels and mouth bar microfacies. Along the source direction, the main sedimentary facies are from pro-delta to front delta to lacustrine mud, with seismic reflections exhibiting strong-strong-weak characteristics. Low-angle strong reflections indicate the development of quartz-feldspar or mixed mineral shale interbedded with thin sandstone in the pro-delta; strong-amplitude parallel reflections suggest mixed shale associations; and weak-amplitude parallel reflections represent clay shale associations.

(2) Sublacustrine Fan Model (LF4←LF3←LF1-2→LF3→LF4)

In the gentle slope zone of the study area, distal turbidite fans are present, where lenticular sand bodies are intercalated within deep-lacustrine mudstones. Thick layers of dark mudstones and oil shales, rich in organic matter, are widely distributed in the deep to semi-deep lacustrine facies, representing high-quality source rock intervals. In the fan midsection and mound areas, weak reflections indicate sandstone-dominated or sandstone-mixed mudstone interbedding; fan-end low-angle strong reflections indicate quartz-feldspar or mixed mineral shale interbedded with thin sandstone associations; and weak amplitude parallel reflections represent the associations dominated of clay shale.

(3) Shore-Shallow Lake Model (LF4←LF3←LF2-1→LF3→LF4 and LF2-2→LF4)

The shore-shallow lacustrine facies in the study area formed as the lake shoreline retreated toward the depression center. The sediments are dominated by siltstones and mudstones, exhibiting wave-generated ripple marks and bioturbation structures. Subaqueous uplift-shallow lake transgression backgrounds with stronger reflections represent carbonate shale or mixed sedimentary rock associations; slope-shallow lake transgression backgrounds with weak or medium-amplitude parallel reflections indicate the development of clay or carbonate shale.

The seismic discrimination of lithofacies should be conducted on the basis of seismic sedimentary facies interpretation. Lithofacies units are roughly consistent with sedimentary facies units, and there is an intrinsic relationship between the distribution of lithofacies and sedimentary facies. The interpretation and prediction of sedimentary facies through well logging curves for main lithologies and sandstone diagenetic facies can aid in predicting lithofacies. Certain lithofacies are prone to confusion with other diagenetic facies or lithofacies that have similar wave impedances. Determining lithofacies within the context of sedimentary facies should effectively reduce errors.

### Characteristics of lithofacies associations in major profiles

**Fig 13:**
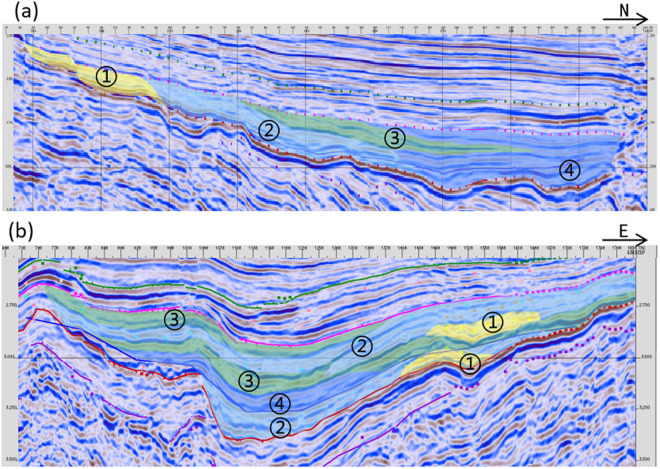
Lithofacies associations identification of the Shahejie Formation in the Huanghekou Sag (a. In the direction along the sediment source; b. In the direction perpendicular to the sediment source; ①Sandstone; ②associations dominated of clay shale; ③associations dominated of mixed mineral shale; ④associations dominated of quartz-feldspar shale).

Based on the characteristics of shale oil lithofacies and their associations development model in E_2_s_3_ of the Huanghekou Sag, profiles along and perpendicular to the sediment source direction were selected for verification (Fig 13).

In the direction along the sediment source, the overall trend is a gradual decrease in grain size, an increase in clay content, and a weakening of hydrodynamic forces. This is manifested by the development sequence of sandstone to quartz-feldspar shale facies associations to mixed shale ones to clay shale ones. The quartz-feldspar shale and some mixed shale facies associations exhibit low-angle progradational reflections, while the bedding features of the clay shale in front gradually become more pronounced, and the continuity of seismic reflection axes gradually enhances (Fig 13a). In the direction perpendicular to the sediment source, multiple isolated lenticular sandstone bodies are observed. Grain size decreases from the sediment source injection point towards both sides, with sandstone developing in the middle and quartz-feldspar shale facies associations dominating on both sides. The thickness of sandstone decreases, and the variation in seismic reflection strength in a single profile is smaller compared to the direction along the sediment source (Fig 13b).

**Fig 14:**
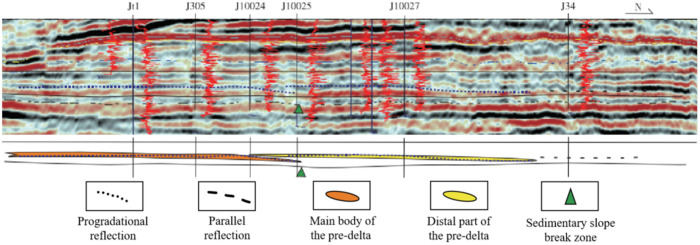
Sedimentary profile interpreted in north-south direction of the Lucaogou Formation.

Previous research results indicate that the development pattern of shale oil horizons in the Lucaogou Formation also exhibits similar characteristics. The main “sweet spot” reservoirs for shale oil exploration in the Permian Lucaogou Formation of the Jimusar Sag are finer-grained sandstones. Regarding their sedimentary origins, various viewpoints include delta fronts, subaqueous fans, and lacustrine beach bars [37–39]. In the comparative profile between Well Jt1 and Well J34, the base interface of the Lucaogou Formation is nearly horizontal, and two periods of relatively distinct low-angle progradational seismic reflections are developed from south to north. The progradational termination points are located in the northern area of Well J10027, and they change into medium-amplitude, well-continuous, parallel seismic reflections in the area of Well J34 (Fig 14). The progradational bodies above the sedimentary slope break are interpreted as the main depositional area of the pre-delta, dominated by sandstones. The progradational ones below the slope break are interpreted as the distal depositional area of the pre-delta, where the lithofacies associations dominated of quartz-feldspar shale develop. The parallel reflections are interpreted as deep lacustrine facies, where the associations of clay shale develop.

### Planar development characteristics of lithofacies associations in E_2_s_3_

**Fig 15:**
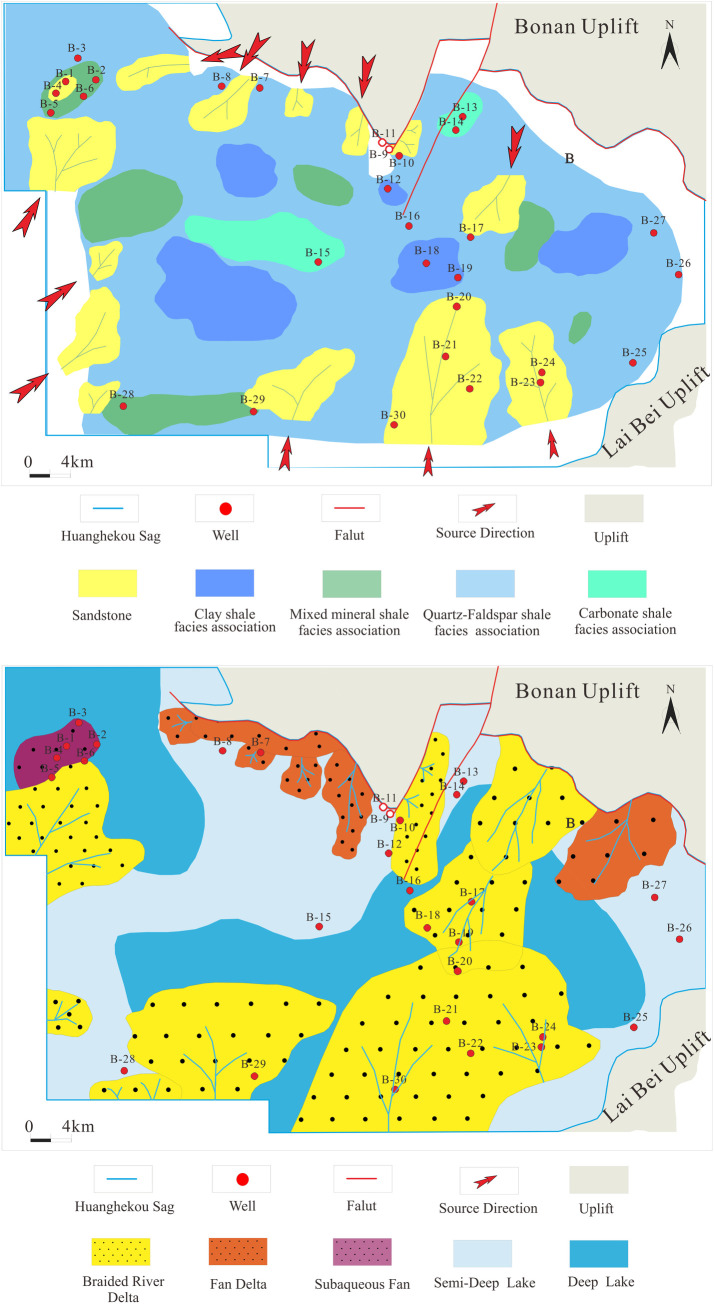
Lithofacies associations and sedimentary facies of the Shahejie Formation in the Huanghekou Sag (Modified from “Map of China’s Topography”, Map Review Number: GS(2016)1609; Source: China Standard Map Service - http://bzdt.ch.mnr.gov.cn/).

Based on the lithofacies associations characteristics of individual wells and the lithofacies associations development patterns identified from seismic profiles, combined with the depositional background of the Huanghekou Sag, we conducted planar identification and delineation to produce a planar distribution map of lithofacies associations (Fig 15).

The deposition period of E_2_s_3_ corresponds to the third rift episode, during which the fault depression was in an expansion phase. Due to tectonic movements, stratigraphic uplift and erosion, parts of the lower and upper E_2_s_3_ are missing in the study area, and they are in obvious unconformable contact with the overlying and underlying strata. The main fault type developed in the area is listric normal faults, and the stratigraphic thickness exhibits a trend of being higher in the south and lower in the north, controlling the changes in the depositional center within the area. The main shale oil horizons develop in the middle part of E_2_s_3_, which corresponds to the period of maximum flooding. The sediment source mainly comes from the south of the basin, and the developed braided river deltas are mainly large lobe bodies distributed in a northeast-southwest orientation. Both the delta front facies and the pre-delta facies have a wide range. The delta front is dominated by medium to coarse sandstones with a high sand-to-mud ratio. The pre-delta is dominated by quartz-feldspar shales intercalated with thin sandstone layers, with a reduced sand-to-mud ratio. In the central areas of the subsidence depressions, semi-deep to deep lacustrine facies are mainly developed, dominated by clay shales. The fault development between the northwest and southwest sub-basins has caused local uplifts, forming platforms, which shallow the water bodies in this area, favoring the formation of carbonate rocks and promoting the development of carbonate shale facies associations. The lithofacies are mainly composed of carbonate shales intercalated with thin sandstone and limestone layers.

In summary, the technology of seismic lithofacies associations identification guided by depositional models demonstrates certain feasibility. By focusing on lithofacies and maintaining a rough consistency between lithofacies units and depositional facies units, the impact of multiple interpretations of seismic reflection characteristics can be reduced, effectively minimizing errors. This provides a new approach for identifying lithofacies and facies associations in intervals lacking core samples and special logging data.

## Conclusions

(1) A method for identifying shale oil lithofacies associations based on sedimentary facies patterns has been established. The study area mainly exhibits three models: Delta, sublacustrine fan, and shore-shallow lake. This provides a new approach for identifying lithofacies and facies associations in intervals lacking core samples and special logging data.
(2) Six lithofacies associations models have been developed in the study area: pre-delta quartz-feldspar shale facies associations, sublacustrine fan terminal quartz-feldspar shale facies associations, mixed beach-bar local platform carbonate shale facies associations, shallow lake carbonate shale facies associations, semi-deep lake mixed mineral shale facies associations, and semi-deep to deep lake clay shale facies associations.
(3) Applying seismic reflection feature to predict the distribution of shale oil lithofacies associations in regions without wells of E_2_s_3_ in the Huanghekou Sag. The pre-delta was relatively developed, and the overall lithofacies associations was dominated by quartz-feldspar shale intercalated with thin sandstone. Locally, platforms developed lithofacies associations of carbonate shale with thin limestone. In the semi-deep lake facies, mixed mineral shale with thin sandstone was present. In the central deep lake facies of the sub-basins, clay shale was dominant. The development patterns of the lithofacies units were kept roughly consistent with the depositional facies units.

## Supporting information

Table S1 Mineral content data. (PDF)

## References

[1] Hu SY, Zhao WZ, Hou LH, Yang Z, Zhu RK, Wu ST, et al. Development potential and technical strategy of continental shale oil in China. Petroleum Exploration and Development. 2020;47(4):877–87. doi: 10.1016/s1876-3804(20)60103-3

[2] Liu HM, Yu BS, Xie ZH, Han SY, Shen ZH, Bai CY. Characteristics and implications of micro-lithofacies in lacustrine-basin organic-rich shale: a case study of Jiyang depression, Bohai Bay Basin. Acta Petrolei Sinica. 2018;39(12):1328–43.

[3] Liu HM, Zhang S, Wang XJ, Zhang PF, Li JL, Wang Y. Types and characteristies of shale lithofacies combinations in continental faulted basins: a case study from upper sub-member of ES, in Dongying sag, Jiyang depression. Earth Science. 2023;48(01):30–48.

[4] Liu HM, Wang Y, Yang YH, Zhang S. Sedimentary environment and lithofacies of fine-grained hybrid sedimentary in Dongying sag: a case of fine-grained sedimentary system of the Es4. Earth Science. 2020;45(10):3543–55.

[5] Ning FX, Wang XJ, Hao XF, Yang WQ, Ding JH. Fine-grained sedimentary rock lithofacies assemblage characteristics in Dongying depression. Journal of Southwest Petroleum University (Science & Technology Edition). 2020;42(04):55–65.

[6] Shen C, Ren L, Zhao JZ, Chen MP. Division of shale lithofacies associations and their impact on fracture network formation in the Silurian Longmaxi Formation, Sichuan Basin. Oil & Gas Geology. 2021;42(01):98–106.

[7] Zhang S, Liu HM, Zhang PF, Li JL, Wang Y, Chen T, et al. Lithofacies assemblages and shale oil enrichment patterns in gentle slopes of the Dongying sagC. Abstract Collection of the 17th National Conference on Palaeogeography and Sedimentology, Sinopec Shengli Oilfield Exploration and Development Research Institute. 2023. p. 2.

[8] Zhang ZW. Prediction of shale oil reservoir dessert in the first section of Qing-1 in the south of Chagan Lake, Songliao Basin. Xi’an Shiyou University; 2020.

[9] Li T, Yin XZ. Analysis on the geological significance of seismic attributes. Complex Hydrocarbon Reservoirs. 2009;2(03):25–8.

[10] Liu XW, Wang XY, Liu YW, Zhang JQ, Liu J, Liu Q. Acta Petrolei Sinica. 2023;44(12):2270–85.

[11] Zeng YL, Long SF, Wu MM, Gao N, Yang C. Reservoir prediction technique based on GeoEast seismic attribute analysis and its application in shale oil exploration and development in Huanxian. Oil Geophysical Prospec. 2022;57(S1):196–201.

[12] Tang YN. Research on the fault activity of central Huanghekou Sag and their controlling on hydrocarbon migration and accumulation. China University of Geosciences (Beijing); 2020.

[13] Cao L, Li C, Qin RS, Mu PF, Zhang PZ. Sedimentary characteristics and quantitative prediction of Neogene thin interbed sand-bodies in Bohai Sea Area. Journal of Xi’an Shiyou University (Natural Science Edition). 2023;38(02):16–24.

[14] Du Z. Hydrocarbon resource assessment on calibrated units in Huanghekou SagD. China University of Petroleum (East China); 2020.

[15] Wang S, Wang FL, Chen RT, Cheng FQ, Liu MX. Organic geochemical characteristies of source rocks in Huanghekou Sag. Journal of Xi’an Shiyou University (Natural Science Edition). 2022;37(03):9–15.

[16] Li S. Source-sink-diagenesis-reservoir integrated high-quality mixed-siliciclastic-carbonate reservoir prediction of Paleogene mixed sediments in Huanghekou sag, Bohai Bay Basin. China University of Geosciences (Wuhan); 2020.

[17] Yang HF, Xu CG, Niu CM, Qian G, Li ZY, Gao FY. Quantitative evaluation of hydrocarbon accumulation pattern and the controlling factors in the Neogene of Huanghekou Sag, Bohai Bay Basin. Oil & Gas Geology. 2020;41(02):259–69.

[18] Liu CZ, Jiang P, Jiang ZQ. Main controlling factors and enrichment pattern of paleogene and neogene hydrocarbon accumulation in Huanghekou Sag. Special Oil and Gas Reservoirs. 2018;25(03):61–6.

[19] Yang Y. Enrichment and high production regularities of shale oil reservoirs in continental rift basin: a case study of Jiyang Depression, Bohai Bay Basin. Petroleum Geology and Recovery Efficiency. 2023;30(01):1–20.

[20] Peng J, Sun NL, Lu K, Xu YL, Chen FL. Shale oil reservoir of the Palaeogene Shahejie Formation in the Dongpu Sag: petrology and pore micro structural characteristics. Earth Science Frontiers. 2023;30(04):128–41.

[21] Zhao XZ, Pu XG, Yan JH, Jin FM, Shi ZN, Chai GQ, et al. Cycles of fine-grained sedimentation and their influences on organic matter distribution in the second member of Paleogene Kongdian Formation in Cangdong Sag, Bohai Bay Basin, East China. Petroleum Exploration and Development. 2023;50(3):534–46. doi: 10.1016/s1876-3804(23)60408-2

[22] Ju-Hua L, Xiao-Qin Z, Cui-Hao L, Hai L, Shiduo L, Fengyu L, et al. Research on the comprehensive dessert evaluation method in shale oil reservoirs based on fractal characteristics of conventional logging curves. Sci Rep. 2025;15(1):9318. doi: 10.1038/s41598-025-93224-w 40102482 PMC11920218

[23] Wang G, Jin Z, Liu G, Liu Q, Liu Z, Wang H, et al. Geological implications of gamma ray (GR) anomalies in marine shales: A case study of the Ordovician-Silurian Wufeng-Longmaxi succession in the Sichuan Basin and its periphery, Southwest China. Journal of Asian Earth Sciences. 2020;199:104359. doi: 10.1016/j.jseaes.2020.104359

[24] Che SQ. Shale lithofacies identification and classification by using logging data: a case of Wufeng-Longmaxi Formation in Fuling Gas Field, Sichuan Basin. Lithologic Reservoirs. 2018;30(01):121–32.

[25] Wang Z, Dong HC, Fan TE, Hu GY, Gao YF. Logging lithofacies analysis based on unsupervised learning. Geophysical Prospecting for Petroleum. 2021;60(03):403–13.

[26] Liu ZB, Liu GX, Hu ZQ, Feng DJ, Zhu T, Bian RK. Lithofacies types and assemblage features of continental shale strata and their significance for shale gas exploration: a case study of the Middle and Lower Jurassic strata in the Sichuan Basin. Natural Gas Industry. 2019;39(12):10–21.

[27] Chen SY, Zhang S, Liu HM, Yan JH. Discussion on mixing of fine-grained sediments in lacustrine deep water. Journal of Palaeogeography. 2017;19(02):271–84.

[28] Lin ZK, Zhang SL, Li CH, Wang M, Yan JP, Cai JG. Types of shale lithofacies assemblage and its significance for shale oil exploration: a case study of Shahejie Formation in Boxing Sag. Petroleum Reservoir Evaluation and Development. 2023;13(01):39–51.

[29] Li MW, Ma XX, Jin ZJ, Li ZM, Jiang QG, Wu SQ. Diversity in the lithofacies assemblages of marine and lacustrine shale strata and significance for unconventional petroleum exploration in China. Oil & Gas Geology. 2022;43(01):1–25.

[30] Zhou LH, Chen CW, Gan HJ, Zhang GX, Zhang YK, Liu HT. Shale formation environment and comprehensive evaluation of shale oil potential of the Lower First Member of Shahejie Formation in Qikou Sag. Bulletin of Geological Science and Technology. 2022;41(05):19–30.

[31] Yang WQ. Shale lithofacies characteristics and development rule of the lower Es3 and upper Es4, Dongying sagD. China University of Petroleum (East China); 2018.

[32] Guo SW, Li BL, Wu XS, Xing X, Guo CX. Application of fusion seismie acquisition, processing and interpretation technology in shale oil exploration in Cangdong Sag. Oil Geophysical Prospecting. 2022;57(S2):147–53.

[33] Li YY, Chen X, Gao Y, Deng Y, Peng SC, Zhang F. Sedimentary morphologys and distributions of shale oil ‘sweet spot’ by the data of well to seismic analysis: a case study of the lower sweet pot in Lucaogou Formation of Jimsar Sag. Fault-block Oil & Gas Field. 2023;30(02):186–95.

[34] Wang ZW, Fu LY, Liu J, Cai ZX. Multiscale characterization and connectivity analysis of 3D seismic attributes of ultra-deep carbonate conduction system in Shunbei, Tarim Basin. Chinese Journal of Geophysics. 2023;66(01):83–94.

[35] Wang LH. Study on seismic prediction method of shale oil reservoir sweet spot: a case study of Mahu Basin. China University of Petroleum (Beijing); 2022.

[36] Hu QZ, Zhang XC, Deng RB, Chen Q. Application of seismic facies analysis technique in shale oil sweet spot prediction. In: Proceedings of the 2020 Joint Annual Meeting of Chinese Geosciences. 2020. p. 4.

[37] Yin SL, Chen GY, Xu CF, Xiong XY, Zhao J, Hu K. Lithofacies architecture of lacustrine fine-grained mixed reservoirs and its control over sweet spot: a case study of Permian Lucaogou Formation shale oil reservoir in the Jimsar Sag, Juggar Basin [J]. Oil & Gas Geology. 2022;43(05):1180–93.

[38] Li SQ, Yin SL, Gao Y, Zhang F, Li YY, Peng SC. Study on sedimentary microfacies of mixed fine-grained rocks in Lucaogou Formation, Jimsar Sag, Junggar Basin. Natural Gas Geoscience. 2020;31(02):235–49.

[39] Yang ZH, Li SL, Yu XH, Wang B, Feng XR. Sedimentary characteristics and facies model of deep-water fan in sand-rich lake of the middle Permian Lucaogou Formation in southern Junggar Basin. Journal of Palaeogeography (Chinese Edition). 2018;20(06):989–1000.

