# Supplementary material for: Seismic prediction of shale oil lithofacies associations based on sedimentary facies patterns: A case study of the shahejie formation in the Huanghekou Sag
Source: PLoS One. 2025 Sep 18;20(9):e0332314. doi: 10.1371/journal.pone.0332314 (PMC12445544; doi:10.1371/journal.pone.0332314)
Supplement: Table S1 — (PDF) [file pone.0332314.s001.pdf]

Table S1 Mineral content data

No.	well+depth	Mineral content								
		Quartz	Anorthose	Plagioclase	Calcite	Dolomite	Pyrite	Other Mineral	Clay Mineral	合计
HH1	B-19 3276.65	29.80	4.70	29.20	0.30	0.30	2.30	0	28.30	100.00
HH2	B-20 3276.00	18.20	3.20	5.50	0.30	0.40	3.00	0	60.40	100.00
HH3	B-20 3281.40	22.00	11.50	33.60	0.30		0.60	0	25.50	100.00
HH4	B-20 3287.00	24.60	19.30	22.20	4.70		0.80	0	23.80	100.00
HH5	B-20 3295.50	27.20	7.10	27.90	0.50		0.70	0	33.10	100.00
HH6	B-20 3297.50	28.40	5.20	25.10	0.30	0.30		0	34.60	100.00
HH7	B-22 3031.00	26.80	6.10	16.90	1.00		0.80	0	45.00	100.00
HH8	B-22 3035.00	23.50	2.10	9.10	1.80	4.10	7.70	0	47.00	100.00
HH9	B-7 3462.50	27.00		3.20	31.60	9.40	3.90	0	22.70	100.00
HH10	B-7 3467.50	18.20		5.10	29.00	36.60	1.70	0	7.70	100.00
HH11	B-7 3477.50	29.50		5.10	4.50	11.10	3.80	0	39.80	100.00
HH12	B-7 3487.50	27.70		5.20	11.30	12.20	3.10	0	32.20	100.00
HH13	B-7 3492.50	26.00		5.50	13.60	12.80	4.20	0	34.40	100.00
HH14	B-7 3497.50	32.30		5.50	4.10	5.40	2.40	0	42.80	100.00
HH15	B-7 3517.50	35.80		4.30	11.20	6.80	3.20	0	36.30	100.00
HH16	B-7 3522.50	26.70		2.90	12.40	6.50	3.80	0	45.10	100.00
HH17	B-7 3527.50	29.00		4.90	10.50	5.00	3.40	0	41.40	100.00
HH18	B-7 3532.50	37.40		4.40	13.20	3.00	3.50	0	36.90	100.00
HH19	B-7 3537.50	28.70		4.40	9.50	5.60	5.00	0	45.10	100.00
HH20	B-7 3542.50	28.10		6.80	17.90	1.50	5.20	0	36.40	100.00
HH21	B-7 3547.50	23.50		6.30	28.70	3.10	3.50	0	31.70	100.00
HH22	B-7 3552.50	27.50		8.40	11.00	3.30	5.30	0	41.60	100.00
HH23	B-7 3557.50	33.30		11.20	13.70	7.30	4.90	0	28.00	100.00
HH24	B-7 3562.50	39.90		13.30	14.60		2.70	0	27.50	100.00
HH25	B-7 3567.50	32.40		7.20	10.70	6.70	4.70	0	35.40	100.00
HH26	B-7 3572.50	39.90		11.10	10.80	3.10	3.60	0	29.70	100.00
HH27	B-7 3577.50	37.00		11.00	10.40	3.60	3.90	0	31.50	100.00
HH28	B-7 3582.50	40.00		8.10	11.40	2.20	2.40	0	33.20	100.00
HH29	B-7 3587.50	35.20		8.50	19.30	3.80	2.70	0	28.20	100.00
HH30	B-7 3592.50	42.60		9.30	10.50		3.20	0	30.40	100.00
HH31	B-7 3597.50	34.10		8.70	15.90	3.30	3.00	0	33.00	100.00
HH32	B-7 3602.50	34.60		6.30	10.70	4.70	3.10	0	36.90	100.00
HH33	B-7 3607.50	36.30		5.10	10.30	7.50	2.70	0	35.40	100.00
HH34	B-7 3612.50	35.80		6.00	9.50	11.10	2.10	0	35.50	100.00
HH35	B-7 3617.50	35.90		5.50	13.20	6.30	2.20	0	34.10	100.00
HH36	B-7 3622.50	40.00		6.70	7.40	8.10	1.20	0	32.70	100.00
HH37	B-7 3627.50	31.70		5.60	10.90	5.50	4.20	0	40.00	100.00
HH38	B-7 3632.50	33.10		4.90	11.20	6.30	2.90	0	39.50	100.00
HH39	B-7 3637.50	36.40		5.20	12.30	3.10	2.40	0	38.40	100.00
HH40	B-7 3647.50	37.80		5.60	6.70	8.60	3.10	0	34.90	100.00
H1	B-2 3413.50	42.70	2.80	10.90	4.80	3.50	2.30	0	33.00	100.00
H2	B-2 3416.90	25.90	2.80	20.20	5.10	0.00	2.70	0	33.80	100.00
H3	B-2 3420.00	30.80	3.70	9.20	5.30	4.50	3.90	0	35.10	100.00
H4	B-2 3423.70	33.70	2.80	9.10	6.30	7.20	2.80	0	34.90	100.00
H5	B-2 3424.00	36.70	7.60	20.80	20.00		1.60	0	13.30	100.00

H6	B-2 3425.25	31.70	2.70	11.30	6.20	3.80	4.80	0	37.60	100.00
H7	B-3 3547.99	30.50	1.70	4.70	4.50	4.70	4.70	0	39.30	100.00
H8	B-3 3549.19	36.80	13.10	19.90	5.10	0.00	3.40	0	19.80	100.00
H10	B-3 3554.19	45.10	8.00	21.00	10.50		1.40	0	11.10	100.00
H11	B-3 3555.06	35.20	3.00	11.10	4.00		2.20	0	37.30	100.00
H12	B-3 3558.48	34.00	4.00	8.80	1.90	5.70	3.90	0	37.90	100.00
H16	B-3 3564.60	18.80	2.50	5.50	44.40		2.00	0	22.50	100.00
H18	B-3 3641.18	46.90	6.50	12.80	2.50			0	20.20	100.00
H19	B-3 3642.47	30.00	5.30	15.30	1.80		2.50	0	31.70	100.00
H20	B-3 3644.13	28.10	6.60	11.80	1.70		5.90	0	34.10	100.00
H21	B-3 3645.85	27.10	4.70	18.50	20.40		10.40	0	10.10	100.00
H23	B-5 3349.76	43.90	7.20	14.50	4.90			0	24.20	100.00
H25	B-5 3542.65	22.50	0.80	3.60	18.70		3.80	0	36.10	100.00
H26	B-5 3544.72	36.80	3.00	8.90	9.70		4.80	0	29.10	100.00
H27	B-5 3546.55	33.50	1.10	4.00	15.70		4.50	0	31.60	100.00
H28	B-21 3095.40	50.10	5.70	13.30	1.20			0	23.60	100.00
H29	B-21 3104.40	45.00	4.00	11.00	1.30			0	31.10	100.00

Table S2 Fisher discriminant analysis data

No.	Actual group	The top group					The second group			Discrimination score					
		Prediction Group	Probability	Degree of freedom	P(G=g | D=d)	The square Mahalis distance calculated relative to the center of mass	Prediction Group	P(G=g | D=d)	The square Mahalis distance calculated relative to the center of mass	Function 1	Function 2	Function 3	Function 4	Function 5	Function 6
1	1	1	0.998	6	1	0.509	4	0	20.177	-1.233	3.81	0.023	1.791	-1.199	0.388
2	1	1	0.996	6	1	0.622	4	0	20.437	-1.235	3.843	-0.019	1.817	-1.139	0.421
3	1	1	0.986	6	1	0.977	4	0	21.956	-1.469	3.956	-0.036	1.88	-0.98	0.495
4	1	1	0.936	6	1	1.818	4	0	23.655	-1.544	4.133	-0.082	1.986	-0.67	0.678
5	1	1	0.834	6	1	2.794	4	0	24.543	-1.47	4.239	-0.184	2.057	-0.383	0.855
6	1	1	0.746	6	1	3.485	4	0	23.742	-1.061	4.253	-0.345	2.07	-0.223	0.958
7	1	1	0.585	6	1	4.686	4	0	23.274	-0.663	4.26	-0.532	2.066	-0.034	1.043
8	1	1	0.663	6	1	4.105	4	0	22.669	-0.652	4.212	-0.474	2.058	-0.15	0.979
9	1	1	0.744	6	1	3.499	4	0	21.755	-0.613	4.136	-0.421	2.035	-0.275	0.891
10	1	1	0.811	6	1	2.98	4	0	20.53	-0.505	4.026	-0.379	1.999	-0.423	0.799
11	1	1	0.86	6	1	2.571	4	0	20.094	-0.486	3.981	-0.325	1.989	-0.541	0.731
12	1	1	0.733	6	1	3.58	4	0	20.543	-0.432	4.038	-0.507	1.989	-0.286	0.795
13	1	1	0.588	6	1	4.664	4	0	21.593	-0.532	4.116	-0.645	2.009	0.013	0.875
14	1	1	0.394	6	1	6.264	4	0	22.972	-0.566	4.207	-0.837	2.017	0.322	0.956
15	1	1	0.241	6	0.999	7.967	4	0	24.547	-0.598	4.296	-1	2.033	0.604	1.033
16	1	1	0.37	6	1	6.492	4	0	24.332	-0.713	4.309	-0.715	2.109	0.439	0.997
17	1	1	0.52	6	1	5.185	4	0	23.705	-0.706	4.284	-0.461	2.161	0.236	0.939
18	1	1	0.646	6	1	4.227	4	0	22.357	-0.471	4.2	-0.29	2.174	-0.011	0.869
19	1	1	0.763	6	1	3.353	4	0	22.259	-0.476	4.186	-0.035	2.228	-0.209	0.809
20	1	1	0.918	6	1	2.013	4	0	21.435	-0.584	4.073	0.1	2.17	-0.609	0.688
21	1	1	0.981	6	1	1.119	4	0	20.599	-0.63	3.942	0.186	2.08	-0.999	0.528

22	1	1	0.976	6	1	1.226	4	0	18.883	-0.244	3.674	0.2	1.927	-1.551	0.318
23	1	1	0.873	6	0.999	2.455	4	0	18.111	0.132	3.4	0.187	1.756	-2.073	0.089
24	1	1	0.874	6	0.999	2.451	4	0.001	16.375	0.164	3.363	-0.121	1.629	-1.785	0.004
25	1	1	0.895	6	0.998	2.253	4	0.001	15.349	0.026	3.376	-0.358	1.543	-1.471	-0.054
26	1	1	0.929	6	0.998	1.895	4	0.001	15.245	-0.36	3.471	-0.564	1.496	-1.053	-0.065
27	1	1	0.901	6	0.998	2.194	4	0.001	16.065	-0.819	3.573	-0.744	1.466	-0.615	-0.052
28	1	1	0.963	6	0.999	1.448	4	0	17.866	-1.054	3.694	-0.543	1.617	-0.783	0.166
29	1	1	0.975	6	1	1.236	4	0	17.881	-0.8	3.661	-0.461	1.674	-1.101	0.297
30	1	1	0.942	6	0.999	1.744	4	0	17.752	-0.379	3.582	-0.451	1.69	-1.444	0.397
31	1	1	0.821	6	0.999	2.903	4	0	18.45	0.052	3.512	-0.415	1.724	-1.816	0.516
32	1	1	0.97	6	1	1.337	4	0	20.585	-0.676	3.715	-0.143	1.873	-1.713	0.618
33	1	1	0.984	6	1	1.043	4	0	22.514	-1.089	3.822	0.051	1.961	-1.708	0.662
34	1	1	0.981	6	1	1.103	4	0	22.8	-1.025	3.798	0.154	1.976	-1.875	0.636
35	1	1	0.977	6	1	1.206	4	0	22.722	-0.954	3.733	0.238	1.972	-2.022	0.608
36	1	1	0.999	6	1	0.308	4	0	17.888	-1.137	3.428	0.149	1.631	-1.614	-0.129
37	1	1	0.97	6	0.999	1.326	4	0.001	16.498	-1.664	3.233	0.115	1.348	-1.074	-0.801
38	1	1	0.62	6	0.988	4.419	3	0.007	14.204	-1.736	2.91	-0.015	0.97	-0.696	-1.601
39	1	1	0.15	6	0.855	9.456	3	0.108	13.596	-1.799	2.596	-0.146	0.603	-0.321	-2.379
40	1	1	0.118	6	0.786	10.171	4	0.116	13.997	-1.219	2.431	-0.129	0.547	-0.432	-2.559
41	1	1	0.075	6	0.63	11.451	4	0.272	13.128	-0.726	2.292	-0.09	0.508	-0.517	-2.723
42	1	4	0.045	6	0.461	12.88	1	0.409	13.12	-0.283	2.168	-0.037	0.479	-0.586	-2.876
43	1	1	0.039	6	0.468	13.238	4	0.429	13.413	-0.317	2.208	0.126	0.541	-0.505	-2.954
44	1	1	0.097	6	0.696	10.722	4	0.246	12.801	-0.287	2.434	0.225	0.753	-0.391	-2.649
45	1	1	0.27	6	0.949	7.581	4	0.043	13.75	-0.689	2.803	0.456	1.056	-0.168	-2.275
46	1	1	0.382	6	0.967	6.384	4	0.028	13.502	-0.444	2.953	0.534	1.235	-0.147	-2.007

47	1	1	0.468	6	0.977	5.616	4	0.018	13.591	-0.202	3.106	0.585	1.408	-0.099	-1.735
48	1	1	0.72	6	0.995	3.682	4	0.004	14.927	0.246	3.268	0.879	1.724	-0.867	-1.135
49	1	1	0.647	6	0.999	4.217	4	0.001	18.979	0.618	3.444	1.191	2.043	-1.61	-0.547
50	1	1	0.298	6	1	7.251	4	0	25.769	0.98	3.649	1.501	2.374	-2.349	0.057
51	1	1	0.088	6	1	11.004	4	0	35.362	0.838	3.953	1.959	2.809	-2.941	0.793
52	1	1	0.363	6	1	6.565	4	0	29	0.265	3.776	1.908	2.448	-2.607	0.348
53	1	1	0.665	6	1	4.086	4	0	23.631	-0.1	3.537	1.806	2.046	-2.338	-0.136
54	1	1	0.8	6	1	3.072	4	0	19.457	-0.378	3.298	1.67	1.624	-2.091	-0.654
55	1	1	0.751	6	0.999	3.443	4	0.001	16.582	-0.664	3.049	1.535	1.193	-1.842	-1.196
56	1	1	0.324	6	1	6.964	4	0	24.798	-1.632	3.108	2.338	0.941	-2.718	-0.877
57	1	1	0.013	6	1	16.101	4	0	39.699	-2.774	3.236	3.211	0.743	-3.566	-0.508
58	1	1	0	6	1	33.766	2	0	54.682	-4.387	3.489	4.198	0.612	-4.268	-0.105
59	1	1	0	6	1	50.787	2	0	69.523	-4.915	3.437	4.887	0.285	-5.278	0.15
60	1	1	0	6	1	41.156	2	0	59.504	-4.972	3.654	4.252	0.374	-4.614	0.443
61	1	1	0	6	1	32.512	2	0	51.068	-4.944	3.878	3.559	0.449	-3.947	0.736
62	1	1	0	6	1	27.389	2	0	44.976	-5.084	4.111	2.921	0.547	-3.233	1.056
63	1	1	0	6	1	24.464	2	0	41.268	-5.216	4.353	2.282	0.655	-2.522	1.399
64	1	1	0.008	6	1	17.302	2	0	39.631	-4.548	4.566	1.556	0.987	-1.862	1.77
65	1	1	0.072	6	1	11.564	3	0	36.632	-3.584	4.691	0.756	1.261	-1.293	2.083
66	2	2	0.66	6	0.971	4.124	3	0.029	11.155	-4.901	0.638	-0.935	0.165	0.664	0.089
67	2	2	0.654	6	0.972	4.17	3	0.028	11.283	-4.934	0.54	-0.956	0.059	0.823	-0.176
68	2	2	0.634	6	0.976	4.32	3	0.024	11.758	-5.009	0.412	-0.982	-0.076	0.923	-0.476
69	2	2	0.61	6	0.98	4.496	3	0.02	12.324	-5.043	0.255	-0.964	-0.213	0.951	-0.821
70	2	2	0.505	6	0.983	5.312	3	0.017	13.455	-5.107	0.074	-0.994	-0.39	1.016	-1.215
71	2	2	0.498	6	0.986	5.364	3	0.014	13.887	-5.078	-0.071	-0.934	-0.482	0.921	-1.421

72	2	2	0.433	6	0.99	5.915	3	0.01	15.074	-5.085	-0.258	-0.865	-0.615	0.829	-1.72
73	2	2	0.427	6	0.982	5.966	3	0.018	13.922	-4.881	-0.323	-0.911	-0.665	0.756	-1.761
74	2	2	0.38	6	0.97	6.399	3	0.03	13.323	-4.701	-0.419	-0.953	-0.744	0.691	-1.869
75	2	2	0.423	6	0.943	6.006	3	0.057	11.603	-4.485	-0.401	-0.998	-0.716	0.612	-1.743
76	2	2	0.431	6	0.889	5.93	3	0.111	10.093	-4.278	-0.388	-1.072	-0.703	0.568	-1.634
77	2	2	0.502	6	0.868	5.329	3	0.132	9.096	-4.195	-0.315	-1.061	-0.615	0.522	-1.455
78	2	2	0.577	6	0.864	4.743	3	0.136	8.436	-4.159	-0.276	-1.048	-0.553	0.383	-1.303
79	2	2	0.664	6	0.908	4.096	3	0.092	8.686	-4.26	-0.227	-0.961	-0.524	0.248	-1.202
80	2	2	0.731	6	0.921	3.6	3	0.079	8.504	-4.314	-0.126	-0.925	-0.464	0.208	-1.02
81	2	2	0.769	6	0.933	3.309	3	0.067	8.593	-4.38	-0.055	-0.9	-0.413	0.19	-0.906
82	2	2	0.793	6	0.946	3.122	3	0.054	8.858	-4.459	0	-0.874	-0.378	0.187	-0.833
83	2	2	0.826	6	0.965	2.859	3	0.035	9.485	-4.623	0.197	-0.813	-0.223	0.303	-0.572
84	2	2	0.827	6	0.978	2.857	3	0.022	10.407	-4.749	0.426	-0.706	-0.019	0.375	-0.243
85	2	2	0.763	6	0.984	3.353	3	0.016	11.596	-4.961	0.525	-0.756	0.038	0.553	-0.165
86	2	2	0.663	6	0.988	4.103	3	0.012	12.917	-5.172	0.634	-0.83	0.101	0.723	-0.052
87	2	2	0.653	6	0.995	4.177	3	0.005	14.803	-5.393	0.651	-0.689	0.119	0.765	-0.106
88	2	2	0.573	6	0.997	4.777	3	0.003	16.493	-5.551	0.735	-0.606	0.182	0.916	-0.049
89	2	2	0.585	6	0.998	4.682	3	0.002	17.676	-5.644	0.646	-0.452	0.126	0.973	-0.36
90	2	2	0.593	6	0.999	4.622	3	0.001	19.264	-5.769	0.535	-0.272	0.063	0.917	-0.675
91	2	2	0.494	6	1	5.393	3	0	21.498	-5.912	0.339	-0.155	-0.1	0.964	-1.176
92	2	2	0.332	6	1	6.879	3	0	25.477	-6.114	0.072	0.078	-0.288	0.825	-1.783
93	2	2	0.18	6	1	8.882	3	0	28.334	-6.183	-0.242	0.097	-0.531	0.68	-2.337
94	2	2	0.046	6	1	12.844	3	0	34.294	-6.297	-0.636	0.271	-0.8	0.396	-3.054
95	2	2	0.032	6	1	13.817	3	0	33.134	-6.204	-0.706	-0.05	-0.946	0.586	-3.176
96	2	2	0.011	6	1	16.468	3	0	33.621	-6.156	-0.823	-0.423	-1.16	0.867	-3.417

97	2	2	0.023	6	1	14.705	3	0	30.426	-5.958	-0.799	-0.515	-1.121	0.767	-3.25
98	2	2	0.033	6	0.999	13.715	3	0.001	27.631	-5.792	-0.787	-0.711	-1.128	0.747	-3.105
99	2	2	0.08	6	0.999	11.277	3	0.001	25.349	-5.588	-0.776	-0.51	-0.999	0.343	-2.939
100	2	2	0.163	6	0.999	9.202	3	0.001	22.892	-5.375	-0.738	-0.398	-0.872	0.014	-2.718
101	2	2	0.203	6	1	8.514	3	0	24.034	-5.19	-0.801	0.07	-0.704	-0.66	-2.663
102	2	2	0.203	6	1	8.508	3	0	25.111	-4.961	-0.814	0.455	-0.518	-1.192	-2.537
103	2	2	0.09	6	1	10.934	3	0	29.383	-4.72	-0.86	1.008	-0.351	-1.866	-2.528
104	2	2	0.015	6	1	15.85	3	0	36.609	-4.482	-0.933	1.643	-0.184	-2.584	-2.582
105	2	2	0.005	6	1	18.411	3	0	40.129	-4.255	-0.846	2.037	0.071	-2.996	-2.347
106	2	2	0.001	6	1	23.157	1	0	41.825	-4.003	-0.736	2.522	0.385	-3.554	-2.033
107	2	2	0.001	6	1	23.845	1	0	40.474	-3.857	-0.677	2.653	0.499	-3.556	-1.953
108	2	2	0	6	0.999	26.876	1	0.001	41.761	-3.73	-0.68	2.954	0.606	-3.671	-2.014
109	2	2	0.001	6	0.998	22.516	1	0.002	35.269	-3.641	-0.448	2.732	0.742	-3.343	-1.638
110	2	2	0.002	6	0.992	20.768	1	0.007	30.621	-3.546	-0.219	2.637	0.927	-3.217	-1.223
111	2	2	0.015	6	0.994	15.724	1	0.005	26.254	-3.589	-0.086	2.241	0.906	-2.746	-1.002
112	2	2	0.06	6	0.99	12.08	1	0.009	21.582	-3.541	0.128	1.896	0.98	-2.274	-0.635
113	2	2	0.256	6	0.995	7.757	1	0.003	19.629	-3.628	0.204	1.39	0.859	-1.605	-0.632
114	2	2	0.532	6	0.994	5.092	3	0.004	16.349	-3.686	0.309	0.907	0.777	-0.967	-0.562
115	2	2	0.735	6	0.988	3.57	3	0.011	12.636	-3.799	0.351	0.428	0.625	-0.267	-0.654
116	2	2	0.702	6	0.952	3.811	3	0.047	9.807	-3.854	0.481	-0.148	0.531	0.492	-0.555
117	2	2	0.724	6	0.981	3.647	3	0.019	11.518	-4.218	0.114	-0.216	0.159	0.502	-1.297
118	2	2	0.435	6	0.993	5.898	3	0.007	15.953	-4.642	-0.315	-0.295	-0.279	0.516	-2.165
119	2	2	0.247	6	0.994	7.879	3	0.006	18.117	-4.771	-0.486	-0.394	-0.52	0.54	-2.545
120	2	2	0.084	6	0.994	11.141	3	0.006	21.525	-4.925	-0.688	-0.523	-0.808	0.645	-3.016
121	2	2	0.051	6	0.986	12.553	3	0.014	20.997	-4.66	-0.864	-0.683	-0.966	0.543	-3.177

122	2	2	0.02	6	0.971	15.042	3	0.029	22.069	-4.441	-1.096	-0.8	-1.165	0.39	-3.448
123	2	2	0.012	6	0.936	16.318	3	0.064	21.678	-4.235	-1.226	-0.962	-1.285	0.253	-3.513
124	2	2	0.007	6	0.833	17.733	3	0.166	20.955	-3.984	-1.319	-1.154	-1.385	0.237	-3.547
125	2	2	0.006	6	0.713	18.059	3	0.287	19.883	-3.921	-1.31	-1.362	-1.422	0.335	-3.471
126	2	2	0.005	6	0.532	18.62	3	0.468	18.876	-3.857	-1.297	-1.6	-1.464	0.467	-3.39
127	2	2	0.007	6	0.53	17.782	3	0.47	18.02	-3.969	-1.22	-1.695	-1.449	0.59	-3.238
128	2	3	0.008	6	0.505	17.491	2	0.495	17.533	-4.09	-1.146	-1.847	-1.457	0.778	-3.099
129	2	2	0.011	6	0.582	16.578	3	0.418	17.237	-4.208	-1.107	-1.833	-1.412	0.759	-3.01
130	2	2	0.016	6	0.716	15.578	3	0.284	17.432	-4.359	-1.1	-1.769	-1.38	0.635	-2.96
131	2	2	0.032	6	0.84	13.831	3	0.16	17.142	-4.601	-0.941	-1.69	-1.248	0.775	-2.774
132	2	2	0.051	6	0.859	12.513	3	0.141	16.127	-4.755	-0.691	-1.714	-1.07	1.133	-2.46
133	2	2	0.058	6	0.88	12.198	3	0.12	16.178	-4.825	-0.538	-1.651	-0.967	1.374	-2.346
134	2	2	0.07	6	0.916	11.647	3	0.084	16.428	-4.934	-0.412	-1.558	-0.875	1.486	-2.241
135	2	2	0.061	6	0.92	12.048	3	0.08	16.939	-4.926	-0.407	-1.515	-0.879	1.609	-2.272
136	2	2	0.055	6	0.938	12.314	3	0.062	17.759	-4.948	-0.435	-1.429	-0.899	1.653	-2.352
137	2	2	0.039	6	0.934	13.247	3	0.066	18.549	-4.941	-0.466	-1.448	-0.936	1.757	-2.448
138	2	2	0.018	6	0.926	15.336	3	0.074	20.39	-4.983	-0.537	-1.539	-1.04	1.932	-2.633
139	2	2	0.025	6	0.946	14.496	3	0.054	20.238	-4.983	-0.56	-1.42	-1.016	1.797	-2.658
140	2	2	0.028	6	0.966	14.108	3	0.034	20.828	-5.033	-0.628	-1.306	-1.037	1.623	-2.756
141	2	2	0.047	6	0.967	12.766	3	0.033	19.508	-4.94	-0.55	-1.198	-0.923	1.627	-2.603
142	2	2	0.063	6	0.96	11.958	3	0.04	18.331	-4.826	-0.453	-1.135	-0.81	1.728	-2.439
143	2	2	0.11	6	0.971	10.355	3	0.029	17.39	-4.827	-0.285	-0.954	-0.649	1.681	-2.245
144	2	2	0.205	6	0.985	8.479	3	0.015	16.789	-4.886	-0.16	-0.733	-0.507	1.466	-2.078
145	2	2	0.26	6	0.992	7.715	3	0.008	17.329	-5.001	-0.103	-0.59	-0.42	1.374	-2.011
146	2	2	0.375	6	0.997	6.451	3	0.003	18.088	-5.147	-0.074	-0.359	-0.321	1.084	-1.945

147	2	2	0.4	6	0.997	6.214	3	0.003	17.602	-5.025	-0.211	-0.336	-0.394	0.804	-2.077
148	2	2	0.4	6	0.995	6.209	3	0.005	16.877	-4.856	-0.308	-0.341	-0.441	0.628	-2.162
149	2	2	0.37	6	0.991	6.492	3	0.009	15.855	-4.7	-0.346	-0.446	-0.48	0.631	-2.196
150	2	2	0.347	6	0.98	6.727	3	0.02	14.525	-4.529	-0.361	-0.583	-0.506	0.657	-2.181
151	2	2	0.233	6	0.973	8.068	3	0.027	15.254	-4.522	-0.438	-0.687	-0.627	0.724	-2.395
152	2	2	0.119	6	0.962	10.144	3	0.038	16.617	-4.546	-0.54	-0.843	-0.793	0.849	-2.67
153	2	2	0.093	6	0.937	10.854	3	0.063	16.255	-4.391	-0.612	-0.913	-0.854	0.822	-2.75
154	2	2	0.074	6	0.897	11.519	3	0.103	15.84	-4.233	-0.677	-0.981	-0.907	0.782	-2.812
155	2	2	0.06	6	0.837	12.078	3	0.163	15.356	-4.118	-0.701	-1.071	-0.944	0.806	-2.828
156	2	2	0.045	6	0.707	12.861	3	0.292	14.628	-3.959	-0.688	-1.189	-0.962	0.95	-2.814
157	2	2	0.041	6	0.574	13.116	3	0.426	13.713	-3.933	-0.528	-1.32	-0.904	1.275	-2.655
158	2	2	0.042	6	0.628	13.036	3	0.372	14.084	-4.056	-0.504	-1.336	-0.927	1.256	-2.666
159	2	2	0.052	6	0.738	12.479	3	0.262	14.546	-4.304	-0.435	-1.359	-0.931	1.279	-2.584
160	2	2	0.06	6	0.822	12.078	3	0.178	15.14	-4.55	-0.363	-1.383	-0.935	1.306	-2.5
161	2	2	0.045	6	0.867	12.905	3	0.133	16.65	-4.827	-0.323	-1.502	-0.984	1.424	-2.51
162	2	2	0.015	6	0.916	15.709	3	0.084	20.485	-5.225	-0.393	-1.693	-1.167	1.569	-2.733
163	2	2	0.042	6	0.849	13.092	3	0.151	16.544	-5.062	-0.093	-1.721	-0.903	1.659	-2.189
164	2	2	0.061	6	0.69	12.061	3	0.31	13.662	-4.895	0.229	-1.838	-0.65	1.836	-1.608
165	2	2	0.127	6	0.656	9.94	3	0.344	11.228	-4.689	0.433	-1.665	-0.394	1.71	-1.184
166	2	2	0.192	6	0.61	8.689	3	0.39	9.58	-4.492	0.634	-1.517	-0.149	1.598	-0.763
167	2	2	0.293	6	0.559	7.312	3	0.441	7.789	-4.143	0.798	-1.244	0.142	1.296	-0.365
168	2	2	0.346	6	0.528	6.736	3	0.471	6.968	-3.83	0.957	-0.99	0.435	0.852	0.095
169	2	2	0.499	6	0.751	5.357	3	0.246	7.586	-3.729	0.904	-0.557	0.566	0.25	0.118
170	2	2	0.331	6	0.726	6.886	3	0.252	9.005	-3.414	1.066	-0.254	0.851	-0.009	0.477
171	2	2	0.317	6	0.789	7.038	3	0.162	10.203	-3.259	1.029	0.104	0.928	-0.041	0.292

172	2	2	0.337	6	0.874	6.826	3	0.071	11.86	-3.19	0.89	0.517	0.92	-0.129	-0.1
173	2	2	0.262	6	0.87	7.691	1	0.079	12.494	-3.2	0.925	0.822	1.066	-0.502	0.009
174	2	2	0.128	6	0.685	9.92	1	0.286	11.669	-3.162	1	1.205	1.283	-0.972	0.219
175	2	2	0.12	6	0.766	10.118	1	0.217	12.642	-3.276	0.94	1.396	1.274	-1.163	0.117
176	2	2	0.087	6	0.808	11.037	1	0.183	14.001	-3.381	0.874	1.672	1.288	-1.433	0.006
177	2	2	0.125	6	0.912	9.982	1	0.083	14.773	-3.52	0.83	1.601	1.209	-1.381	-0.1
178	2	2	0.132	6	0.946	9.836	1	0.051	15.689	-3.635	0.794	1.642	1.178	-1.449	-0.177
179	2	2	0.291	6	0.99	7.34	1	0.008	17.08	-3.859	0.711	1.36	0.983	-1.165	-0.389
180	2	2	0.475	6	0.997	5.557	3	0.001	18.723	-4.081	0.625	1.107	0.795	-0.909	-0.606
181	2	2	0.674	6	0.997	4.018	3	0.003	15.968	-4.217	0.489	0.723	0.542	-0.594	-0.851
182	2	2	0.767	6	0.995	3.325	3	0.005	13.852	-4.378	0.327	0.279	0.236	-0.164	-1.181
183	2	2	0.753	6	0.973	3.435	3	0.027	10.628	-4.172	0.331	-0.151	0.142	0.093	-1.104
184	2	2	0.646	6	0.868	4.229	3	0.131	8.006	-3.976	0.328	-0.607	0.03	0.38	-1.045
185	2	2	0.492	6	0.534	5.409	3	0.465	5.686	-3.632	0.351	-0.928	-0.012	0.472	-0.914
186	2	3	0.691	6	0.856	3.896	2	0.142	7.484	-3.291	0.371	-1.316	-0.086	0.683	-0.819
187	2	3	0.843	6	0.938	2.724	2	0.058	8.278	-3.01	0.431	-1.422	-0.015	0.502	-0.556
188	2	3	0.925	6	0.969	1.944	2	0.026	9.177	-2.773	0.461	-1.529	0.031	0.236	-0.317
189	2	3	0.869	6	0.933	2.498	2	0.063	7.894	-2.956	0.47	-1.398	0.108	0.007	-0.159
190	2	3	0.802	6	0.917	3.055	2	0.079	7.966	-3.013	0.602	-1.362	0.265	0.03	0.176
191	2	3	0.733	6	0.877	3.584	2	0.119	7.583	-3.119	0.661	-1.279	0.326	0.064	0.247
192	2	3	0.639	6	0.791	4.282	2	0.206	6.974	-3.26	0.696	-1.169	0.378	-0.023	0.312
193	2	3	0.549	6	0.586	4.963	2	0.412	5.668	-3.491	0.623	-1.037	0.337	-0.124	0.153
194	2	2	0.573	6	0.638	4.773	3	0.361	5.915	-3.684	0.588	-0.888	0.338	-0.236	0.071
195	2	2	0.731	6	0.86	3.598	3	0.139	7.237	-4.095	0.501	-0.817	0.224	-0.096	-0.237
196	2	2	0.799	6	0.961	3.074	3	0.039	9.489	-4.568	0.344	-0.762	0.033	0.085	-0.702

197	2	2	0.803	6	0.972	3.047	3	0.028	10.125	-4.678	0.365	-0.735	0.042	0.118	-0.706
198	2	2	0.803	6	0.978	3.047	3	0.022	10.644	-4.769	0.409	-0.713	0.073	0.153	-0.661
199	2	2	0.836	6	0.979	2.776	3	0.021	10.451	-4.663	0.298	-0.643	0.045	0.023	-0.751
200	2	2	0.859	6	0.982	2.585	3	0.018	10.641	-4.573	0.165	-0.536	0.009	-0.155	-0.878
201	2	2	0.854	6	0.981	2.625	3	0.019	10.565	-4.445	0.067	-0.473	-0.015	-0.34	-0.961
202	2	2	0.837	6	0.977	2.773	3	0.023	10.257	-4.268	0.003	-0.411	-0.012	-0.428	-1.015
203	2	2	0.805	6	0.968	3.027	3	0.032	9.829	-4.104	-0.065	-0.404	-0.034	-0.455	-1.081
204	2	2	0.768	6	0.954	3.317	3	0.046	9.369	-3.928	-0.116	-0.397	-0.037	-0.498	-1.107
205	2	2	0.685	6	0.941	3.939	3	0.059	9.494	-3.754	-0.248	-0.357	-0.111	-0.569	-1.275
206	2	2	0.564	6	0.926	4.84	3	0.073	9.923	-3.607	-0.406	-0.341	-0.222	-0.604	-1.508
207	2	2	0.562	6	0.947	4.86	3	0.052	10.647	-3.547	-0.44	-0.185	-0.148	-0.717	-1.487
208	2	2	0.541	6	0.961	5.023	3	0.038	11.471	-3.49	-0.484	-0.037	-0.093	-0.775	-1.512
209	2	2	0.495	6	0.973	5.387	3	0.026	12.66	-3.403	-0.47	0.186	0.021	-1.063	-1.399
210	2	2	0.346	6	0.986	6.738	3	0.011	15.703	-3.316	-0.476	0.561	0.17	-1.537	-1.303
211	2	2	0.454	6	0.987	5.729	3	0.011	14.676	-3.426	-0.381	0.462	0.221	-1.457	-1.108
212	2	2	0.57	6	0.986	4.797	3	0.013	13.471	-3.507	-0.259	0.351	0.292	-1.336	-0.871
213	2	2	0.727	6	0.991	3.624	3	0.009	13.08	-3.784	-0.16	0.243	0.326	-1.191	-0.701
214	2	2	0.86	6	0.988	2.573	3	0.012	11.417	-3.934	0.057	0.035	0.428	-0.754	-0.383
215	2	2	0.887	6	0.994	2.332	3	0.006	12.598	-4.293	0.355	0.063	0.592	-0.419	-0.121
216	2	2	0.817	6	0.997	2.937	3	0.003	14.564	-4.648	0.653	0.085	0.752	-0.056	0.13
217	2	2	0.652	6	0.996	4.181	3	0.004	15.394	-4.646	0.886	0.076	0.929	0.184	0.435
218	2	2	0.353	6	0.992	6.669	3	0.008	16.28	-4.602	1.188	-0.098	1.117	0.62	0.856
219	2	2	0.378	6	0.997	6.419	3	0.002	18.539	-4.707	1.19	0.274	1.219	0.292	0.809
220	2	2	0.359	6	0.999	6.608	1	0.001	21.558	-4.834	1.137	0.827	1.329	-0.218	0.66
221	2	2	0.283	6	0.999	7.435	1	0.001	21.319	-4.832	1.244	0.792	1.408	-0.176	0.83

222	2	2	0.197	6	0.997	8.598	1	0.002	20.979	-4.796	1.374	0.767	1.511	-0.093	1.027
223	2	2	0.2	6	0.984	8.565	1	0.01	17.737	-4.242	1.358	0.402	1.451	0.018	1.113
224	2	2	0.202	6	0.93	8.523	3	0.042	14.727	-3.783	1.264	0.063	1.333	-0.052	1.11
225	2	2	0.169	6	0.714	9.08	3	0.226	11.38	-3.33	1.156	-0.272	1.207	-0.062	1.102
226	2	3	0.188	6	0.57	8.75	2	0.356	9.692	-2.941	0.995	-0.566	1.051	-0.237	1.044
227	2	3	0.194	6	0.497	8.661	2	0.463	8.802	-3.12	0.935	-0.617	0.981	-0.438	1
228	2	2	0.208	6	0.499	8.43	3	0.479	8.514	-3.259	0.917	-0.721	0.928	-0.513	1.004
229	2	3	0.198	6	0.595	8.597	2	0.379	9.498	-3.222	1.032	-0.841	0.994	-0.296	1.167
230	2	3	0.204	6	0.744	8.49	2	0.237	10.78	-3.2	1.143	-1.047	1.024	0.029	1.305
231	2	3	0.136	6	0.777	9.733	2	0.186	12.588	-3.135	1.305	-1.037	1.126	0.094	1.559
232	2	3	0.091	6	0.822	10.915	2	0.125	14.69	-3.074	1.468	-1.09	1.204	0.259	1.793
233	2	3	0.054	6	0.778	12.402	2	0.114	16.244	-3.02	1.564	-1.037	1.309	0.205	2.01
234	2	3	0.031	6	0.653	13.89	1	0.205	16.208	-3.029	1.611	-0.94	1.391	-0.032	2.192
235	2	3	0.035	6	0.568	13.56	1	0.214	15.512	-3.102	1.502	-0.822	1.338	-0.325	2.084
236	2	3	0.028	6	0.502	14.179	1	0.309	15.149	-3.021	1.528	-0.758	1.385	-0.351	2.154
237	2	3	0.06	6	0.622	12.09	1	0.257	13.856	-2.606	1.361	-0.81	1.274	-0.428	1.987
238	2	3	0.118	6	0.681	10.162	1	0.165	13	-2.235	1.15	-0.843	1.129	-0.56	1.748
239	2	3	0.094	6	0.631	10.821	1	0.214	12.986	-2.389	1.168	-0.743	1.178	-0.589	1.767
240	2	3	0.069	6	0.55	11.687	1	0.278	13.051	-2.534	1.197	-0.644	1.237	-0.619	1.808
241	2	3	0.07	6	0.537	11.658	1	0.229	13.357	-2.732	1.179	-0.591	1.223	-0.604	1.729
242	2	3	0.083	6	0.465	11.173	2	0.388	11.532	-2.988	1.096	-0.52	1.149	-0.615	1.52
243	2	3	0.053	6	0.426	12.428	2	0.384	12.636	-3.059	1.227	-0.534	1.254	-0.514	1.733
244	2	3	0.028	6	0.392	14.178	2	0.319	14.589	-3.089	1.399	-0.565	1.391	-0.365	2.015
245	2	2	0.022	6	0.456	14.767	1	0.28	15.742	-3.327	1.52	-0.456	1.471	-0.239	2.107
246	2	2	0.021	6	0.609	14.922	1	0.26	16.624	-3.588	1.62	-0.301	1.55	-0.218	2.183

247	2	2	0.017	6	0.851	15.403	1	0.118	19.359	-4.13	1.809	-0.082	1.657	-0.038	2.274
248	2	2	0.017	6	0.97	15.509	1	0.026	22.732	-4.764	1.91	0.217	1.714	-0.076	2.251
249	2	2	0.021	6	0.986	14.898	1	0.012	23.651	-4.952	1.866	0.274	1.664	-0.215	2.191
250	2	2	0.021	6	0.99	14.955	1	0.009	24.405	-5.077	1.867	0.353	1.66	-0.28	2.181
251	2	2	0.014	6	0.991	15.969	1	0.008	25.654	-5.112	1.969	0.201	1.667	0.04	2.292
252	2	2	0.004	6	0.978	19.26	1	0.019	27.121	-5.042	2.177	0.072	1.788	0.361	2.611
253	2	2	0.016	6	0.996	15.643	3	0.003	27.564	-5.341	1.987	-0.138	1.495	0.634	2.15
254	2	2	0.046	6	0.998	12.799	3	0.002	25.217	-5.693	1.761	-0.41	1.149	0.908	1.64
255	2	2	0.062	6	0.999	12.002	3	0.001	25.035	-5.893	1.648	-0.492	0.989	1.16	1.323
256	2	2	0.04	6	0.998	13.196	3	0.002	25.626	-6.001	1.626	-0.685	0.871	1.66	1.125
257	2	2	0.03	6	0.998	13.979	3	0.002	26.54	-6.131	1.549	-0.771	0.743	2.059	0.781
258	2	2	0.018	6	0.998	15.301	3	0.002	28.253	-6.319	1.409	-0.872	0.545	2.487	0.3
259	2	2	0.01	6	0.996	16.811	3	0.004	28.082	-6.113	1.439	-0.963	0.556	2.815	0.254
260	2	2	0.013	6	0.997	16.207	3	0.003	27.693	-6.155	1.231	-0.999	0.364	2.863	-0.173
261	2	2	0.024	6	0.996	14.531	3	0.004	25.666	-5.994	1.322	-0.923	0.525	2.576	0.151
262	2	2	0.036	6	0.995	13.496	3	0.005	24.091	-5.783	1.47	-0.829	0.751	2.243	0.607
263	2	2	0.043	6	0.995	13.013	3	0.005	23.45	-5.787	1.458	-0.873	0.74	2.127	0.614
264	2	2	0.056	6	0.995	12.272	3	0.005	22.866	-5.821	1.399	-0.889	0.687	2.037	0.5
265	2	2	0.065	6	0.994	11.889	3	0.006	22.133	-5.729	1.493	-0.871	0.807	1.738	0.808
266	2	2	0.042	6	0.991	13.071	3	0.009	22.447	-5.537	1.708	-0.841	1.059	1.359	1.397
267	2	2	0.121	6	0.994	10.084	3	0.006	20.447	-5.646	1.467	-0.813	0.847	1.129	0.986
268	2	2	0.249	6	0.996	7.852	3	0.004	19.136	-5.745	1.243	-0.785	0.652	0.882	0.616
269	2	2	0.598	6	0.995	4.586	3	0.005	15.066	-5.508	0.788	-0.825	0.294	0.44	0.005
270	2	2	0.724	6	0.986	3.649	3	0.014	12.088	-5.174	0.411	-0.965	-0.038	0.333	-0.559
271	2	2	0.666	6	0.99	4.08	3	0.01	13.233	-5.339	0.351	-0.97	-0.148	0.304	-0.838

272	2	2	0.579	6	0.991	4.727	3	0.009	14.199	-5.447	0.336	-0.979	-0.222	0.368	-1.067
273	2	2	0.004	6	1	19.331	3	0	59.961	-6.806	-3.796	1.092	-0.902	0.723	-2.138
274	2	2	0.003	6	1	20.108	3	0	61.287	-6.832	-3.841	1.153	-0.909	0.723	-2.202
275	2	2	0.002	6	1	20.637	3	0	62.13	-6.831	-3.879	1.196	-0.913	0.722	-2.249
276	2	2	0.002	6	1	20.883	3	0	62.359	-6.794	-3.914	1.212	-0.914	0.722	-2.281
277	2	2	0.002	6	1	21.086	3	0	62.361	-6.736	-3.954	1.212	-0.922	0.725	-2.318
278	2	2	0.002	6	1	21.244	3	0	62.096	-6.646	-4.001	1.198	-0.935	0.721	-2.362
279	2	2	0.002	6	1	20.637	3	0	60.421	-6.446	-4.041	1.169	-0.931	0.677	-2.355
280	2	2	0.003	6	1	19.845	3	0	57.813	-6.108	-4.105	1.124	-0.938	0.575	-2.353
281	2	2	0.005	6	1	18.713	3	0	54.552	-5.697	-4.151	1.094	-0.915	0.429	-2.294
282	2	2	0.007	6	1	17.72	3	0	51.085	-5.194	-4.194	1.086	-0.873	0.222	-2.204
283	2	2	0.008	6	1	17.234	3	0	48.068	-4.66	-4.229	1.1	-0.818	-0.013	-2.096
284	2	2	0.008	6	1	17.286	3	0	45.454	-4.074	-4.246	1.142	-0.736	-0.286	-1.948
285	2	2	0.006	6	1	18.118	4	0	41.08	-3.469	-4.251	1.193	-0.64	-0.566	-1.775
286	2	2	0.003	6	1	19.664	4	0	36.35	-2.838	-4.232	1.231	-0.528	-0.841	-1.556
287	2	2	0.002	6	0.99	21.252	4	0.004	32.04	-2.254	-4.166	1.221	-0.401	-1.036	-1.267
288	2	2	0.001	6	0.805	22.492	5	0.123	26.247	-1.756	-4.048	1.164	-0.26	-1.145	-0.906
289	2	5	0.002	6	0.574	21.316	2	0.21	23.325	-1.339	-3.894	1.018	-0.128	-1.139	-0.499
290	2	5	0.007	6	0.665	17.8	4	0.12	21.226	-1.055	-3.7	0.748	-0.017	-0.983	-0.042
291	2	5	0.014	6	0.557	15.921	7	0.177	18.207	-0.888	-3.487	0.426	0.08	-0.751	0.428
292	2	5	0.012	6	0.308	16.305	7	0.257	16.664	-0.912	-3.264	0.06	0.151	-0.45	0.885
293	2	6	0.013	6	0.368	16.134	7	0.27	16.753	-1.071	-3.076	-0.263	0.191	-0.184	1.259
294	2	6	0.009	6	0.374	17.048	2	0.195	18.353	-1.409	-2.922	-0.509	0.206	0.041	1.534
295	2	2	0.019	6	0.797	15.199	6	0.083	19.733	-1.94	-2.825	-0.582	0.207	0.103	1.681
296	2	2	0.079	6	0.992	11.32	3	0.004	22.251	-2.69	-2.728	-0.542	0.219	0.137	1.771

297	2	2	0.225	6	1	8.186	3	0	23.892	-3.487	-2.684	-0.411	0.21	0.066	1.766
298	2	2	0.401	6	1	6.202	3	0	27.247	-4.311	-2.671	-0.208	0.193	-0.069	1.707
299	2	2	0.46	6	1	5.682	3	0	31.9	-5.08	-2.684	0.012	0.165	-0.234	1.62
300	2	2	0.383	6	1	6.37	3	0	37.238	-5.738	-2.708	0.236	0.138	-0.408	1.54
301	2	2	0.253	6	1	7.803	3	0	42.72	-6.301	-2.726	0.444	0.116	-0.545	1.482
302	2	2	0.147	6	1	9.499	3	0	47.677	-6.735	-2.747	0.629	0.094	-0.653	1.441
303	2	2	0.097	6	1	10.741	3	0	50.962	-6.971	-2.745	0.783	0.101	-0.693	1.464
304	2	2	0.082	6	1	11.225	3	0	52.193	-6.991	-2.779	0.889	0.092	-0.733	1.487
305	2	2	0.08	6	1	11.292	3	0	52.49	-6.927	-2.794	0.983	0.113	-0.72	1.537
306	2	2	0.088	6	1	11.012	3	0	51.964	-6.765	-2.805	1.079	0.163	-0.693	1.6
307	2	2	0.098	6	1	10.708	3	0	51.29	-6.577	-2.81	1.174	0.234	-0.656	1.668
308	2	2	0.11	6	1	10.371	3	0	50.47	-6.369	-2.823	1.26	0.316	-0.605	1.713
309	2	2	0.118	6	1	10.163	3	0	49.719	-6.159	-2.828	1.335	0.411	-0.555	1.775
310	2	2	0.123	6	1	10.039	3	0	49.129	-5.969	-2.848	1.398	0.5	-0.505	1.806
311	2	2	0.124	6	1	10.004	3	0	48.726	-5.817	-2.873	1.444	0.571	-0.46	1.824
312	2	2	0.122	6	1	10.052	3	0	48.572	-5.701	-2.894	1.488	0.637	-0.418	1.834
313	2	2	0.119	6	1	10.134	3	0	48.538	-5.611	-2.921	1.525	0.689	-0.39	1.832
314	2	2	0.112	6	1	10.31	3	0	48.583	-5.529	-2.929	1.556	0.748	-0.378	1.864
315	2	2	0.106	6	1	10.472	3	0	48.607	-5.455	-2.937	1.581	0.799	-0.368	1.892
316	2	2	0.101	6	1	10.625	3	0	48.512	-5.38	-2.931	1.593	0.852	-0.354	1.937
317	2	2	0.096	6	1	10.777	3	0	48.245	-5.294	-2.911	1.588	0.908	-0.329	2.006
318	2	2	0.088	6	1	10.999	3	0	47.804	-5.187	-2.866	1.563	0.979	-0.295	2.12
319	2	2	0.085	6	1	11.107	3	0	46.879	-5.053	-2.817	1.503	1.035	-0.238	2.23
320	2	2	0.084	6	1	11.14	3	0	45.503	-4.878	-2.768	1.417	1.076	-0.18	2.327
321	2	2	0.083	6	1	11.191	3	0	43.971	-4.694	-2.718	1.312	1.106	-0.116	2.418

322	2	2	0.081	6	1	11.265	3	0	42.3	-4.495	-2.671	1.193	1.124	-0.062	2.496
323	2	2	0.079	6	1	11.32	4	0	40.463	-4.304	-2.637	1.077	1.126	-0.017	2.544
324	2	2	0.079	6	1	11.317	4	0	38.565	-4.12	-2.618	0.973	1.11	-0.001	2.553
325	2	2	0.077	6	1	11.386	4	0	36.787	-3.94	-2.605	0.889	1.092	-0.018	2.551
326	2	2	0.074	6	1	11.518	4	0	35.083	-3.76	-2.597	0.822	1.072	-0.065	2.539
327	2	2	0.069	6	1	11.7	4	0	33.809	-3.618	-2.588	0.8	1.059	-0.146	2.524
328	2	2	0.065	6	1	11.851	4	0	32.977	-3.527	-2.568	0.797	1.052	-0.217	2.52
329	2	2	0.06	6	1	12.091	4	0	32.468	-3.461	-2.532	0.802	1.058	-0.285	2.546
330	2	2	0.055	6	1	12.348	4	0	32.371	-3.439	-2.477	0.8	1.075	-0.325	2.609
331	2	2	0.049	6	1	12.653	4	0	32.586	-3.446	-2.406	0.79	1.1	-0.348	2.701
332	2	2	0.044	6	1	12.957	4	0	33.292	-3.505	-2.32	0.767	1.131	-0.347	2.826
333	2	2	0.042	6	1	13.053	4	0	34.269	-3.609	-2.249	0.744	1.146	-0.349	2.923
334	2	2	0.039	6	1	13.246	4	0	35.757	-3.754	-2.171	0.732	1.169	-0.368	3.038
335	2	2	0.044	6	1	12.911	3	0	37.561	-3.997	-2.14	0.761	1.159	-0.444	3.061
336	2	2	0.05	6	1	12.589	3	0	39.054	-4.328	-2.104	0.795	1.15	-0.52	3.079
337	2	2	0.059	6	1	12.119	3	0	40.721	-4.716	-2.104	0.824	1.104	-0.618	3.033
338	2	2	0.068	6	1	11.735	3	0	42.926	-5.168	-2.139	0.869	1.028	-0.754	2.92
339	2	2	0.066	6	1	11.812	3	0	45.606	-5.628	-2.186	0.904	0.938	-0.903	2.798
340	2	2	0.055	6	1	12.324	3	0	48.803	-6.103	-2.256	0.935	0.823	-1.068	2.641
341	2	2	0.033	6	1	13.71	3	0	53.232	-6.559	-2.336	1.048	0.727	-1.316	2.474
342	2	2	0.015	6	1	15.848	3	0	58.717	-7.005	-2.444	1.223	0.626	-1.614	2.258
343	2	2	0.005	6	1	18.777	3	0	64.957	-7.378	-2.555	1.463	0.539	-1.951	2.056
344	2	2	0.001	6	1	22.267	3	0	71.632	-7.678	-2.699	1.746	0.43	-2.311	1.824
345	2	2	0	6	1	26.353	3	0	78.837	-7.927	-2.841	2.073	0.324	-2.665	1.595
346	2	2	0	6	1	29.994	3	0	85.018	-8.115	-2.981	2.361	0.188	-2.912	1.372

347	2	2	0	6	1	33.279	3	0	90.515	-8.281	-3.129	2.618	0.017	-3.068	1.119
348	2	2	0	6	1	35.243	3	0	93.932	-8.387	-3.259	2.806	-0.183	-3.069	0.884
349	2	2	0	6	1	36.065	3	0	95.631	-8.464	-3.373	2.929	-0.404	-2.932	0.653
350	2	2	0	6	1	35.88	3	0	95.745	-8.5	-3.467	2.997	-0.64	-2.677	0.446
351	2	2	0	6	1	33.696	3	0	92.719	-8.429	-3.541	2.932	-0.794	-2.307	0.232
352	2	2	0	6	1	30.577	3	0	87.891	-8.248	-3.636	2.757	-0.835	-1.963	-0.04
353	2	2	0	6	1	26.827	3	0	81.809	-7.993	-3.696	2.539	-0.829	-1.572	-0.259
354	2	2	0.001	6	1	23.074	3	0	75.267	-7.669	-3.746	2.295	-0.781	-1.186	-0.473
355	2	2	0.003	6	1	19.569	3	0	68.771	-7.316	-3.758	2.053	-0.712	-0.801	-0.612
356	2	2	0.011	6	1	16.586	3	0	62.72	-6.955	-3.749	1.818	-0.647	-0.431	-0.707
357	2	2	0.028	6	1	14.127	3	0	57.268	-6.604	-3.718	1.593	-0.575	-0.102	-0.746
358	2	2	0.054	6	1	12.355	3	0	52.735	-6.288	-3.683	1.385	-0.52	0.195	-0.77
359	2	2	0.081	6	1	11.266	3	0	49.571	-6.075	-3.645	1.208	-0.478	0.422	-0.776
360	2	2	0.102	6	1	10.587	3	0	47.607	-5.982	-3.581	1.077	-0.438	0.634	-0.75
361	2	2	0.117	6	1	10.186	3	0	46.364	-5.947	-3.529	0.969	-0.408	0.782	-0.727
362	2	2	0.136	6	1	9.742	3	0	45.464	-5.965	-3.459	0.882	-0.366	0.875	-0.654
363	2	2	0.15	6	1	9.441	3	0	44.859	-6.001	-3.404	0.802	-0.337	0.936	-0.595
364	2	2	0.17	6	1	9.059	3	0	44.249	-6.042	-3.346	0.73	-0.303	0.951	-0.508
365	2	2	0.196	6	1	8.62	3	0	43.508	-6.066	-3.29	0.658	-0.269	0.942	-0.408
366	2	2	0.224	6	1	8.201	3	0	42.709	-6.071	-3.245	0.587	-0.244	0.91	-0.317
367	2	2	0.263	6	1	7.672	3	0	41.633	-6.028	-3.21	0.525	-0.216	0.841	-0.212
368	2	2	0.303	6	1	7.194	3	0	40.392	-5.928	-3.207	0.468	-0.208	0.745	-0.137
369	2	2	0.343	6	1	6.765	3	0	39.318	-5.826	-3.206	0.434	-0.194	0.633	-0.058
370	2	2	0.366	6	1	6.533	3	0	38.686	-5.734	-3.227	0.431	-0.191	0.513	-0.018
371	2	2	0.384	6	1	6.359	3	0	38.334	-5.664	-3.241	0.451	-0.178	0.394	0.033

372	2	2	0.39	6	1	6.308	3	0	38.472	-5.635	-3.252	0.502	-0.159	0.284	0.075
373	2	2	0.384	6	1	6.365	3	0	38.956	-5.638	-3.264	0.567	-0.143	0.187	0.105
374	2	2	0.384	6	1	6.359	3	0	39.533	-5.666	-3.245	0.643	-0.098	0.097	0.191
375	2	2	0.4	6	1	6.21	3	0	39.904	-5.696	-3.175	0.715	-0.011	0.009	0.371
376	2	2	0.403	6	1	6.181	3	0	40.612	-5.77	-3.079	0.793	0.091	-0.069	0.578
377	2	2	0.399	6	1	6.224	3	0	41.157	-5.818	-2.944	0.859	0.228	-0.161	0.868
378	2	2	0.367	6	1	6.529	3	0	41.85	-5.857	-2.789	0.915	0.379	-0.266	1.194
379	2	2	0.306	6	1	7.161	3	0	42.746	-5.883	-2.627	0.963	0.535	-0.379	1.535
380	2	2	0.232	6	1	8.088	3	0	43.821	-5.899	-2.468	1	0.685	-0.497	1.867
381	2	2	0.161	6	1	9.234	3	0	45.049	-5.913	-2.318	1.025	0.822	-0.604	2.176
382	2	2	0.108	6	1	10.423	3	0	46.268	-5.928	-2.188	1.038	0.936	-0.696	2.439
383	2	2	0.083	6	1	11.187	3	0	47.242	-5.984	-2.106	1.044	0.992	-0.742	2.574
384	2	2	0.065	6	1	11.886	3	0	48.131	-6.039	-2.025	1.05	1.042	-0.775	2.691
385	2	2	0.055	6	1	12.341	3	0	48.914	-6.115	-1.964	1.062	1.07	-0.782	2.749
386	2	2	0.047	6	1	12.744	3	0	49.685	-6.197	-1.9	1.08	1.097	-0.767	2.794
387	2	2	0.04	6	1	13.194	3	0	50.5	-6.276	-1.832	1.101	1.131	-0.747	2.844
388	2	2	0.034	6	1	13.674	3	0	51.301	-6.353	-1.756	1.118	1.17	-0.711	2.9
389	2	2	0.029	6	1	14.031	3	0	51.685	-6.438	-1.674	1.074	1.195	-0.615	2.965
390	2	2	0.025	6	1	14.484	3	0	51.921	-6.523	-1.579	0.986	1.221	-0.481	3.056
391	2	2	0.022	6	1	14.785	3	0	51.635	-6.576	-1.501	0.852	1.228	-0.334	3.141
392	2	2	0.023	6	1	14.646	3	0	50.674	-6.588	-1.47	0.699	1.2	-0.201	3.169
393	2	2	0.027	6	1	14.237	3	0	49.253	-6.567	-1.47	0.533	1.147	-0.087	3.162
394	2	2	0.038	6	1	13.344	3	0	47.224	-6.492	-1.524	0.381	1.063	-0.026	3.092
395	2	2	0.054	6	1	12.364	3	0	44.976	-6.378	-1.596	0.237	0.97	0.002	3.01
396	2	2	0.081	6	1	11.251	3	0	42.414	-6.204	-1.697	0.106	0.865	-0.026	2.912

397	2	2	0.113	6	1	10.292	3	0	39.999	-5.997	-1.804	0.002	0.768	-0.106	2.823
398	2	2	0.147	6	1	9.504	3	0	37.666	-5.744	-1.924	-0.081	0.674	-0.242	2.742
399	2	2	0.157	6	1	9.311	3	0	36.425	-5.528	-2.011	-0.1	0.619	-0.441	2.722
400	2	2	0.142	6	1	9.609	3	0	36.161	-5.38	-2.061	-0.082	0.59	-0.649	2.757
401	2	2	0.107	6	1	10.443	3	0	36.853	-5.287	-2.07	-0.03	0.598	-0.879	2.862
402	2	2	0.068	6	1	11.748	3	0	38.619	-5.296	-2.033	0.05	0.631	-1.104	3.022
403	2	2	0.041	6	1	13.138	3	0	40.886	-5.381	-1.989	0.142	0.656	-1.307	3.169
404	2	2	0.022	6	1	14.788	3	0	43.929	-5.566	-1.917	0.248	0.685	-1.475	3.333
405	2	2	0.012	6	1	16.365	3	0	47.372	-5.806	-1.861	0.391	0.695	-1.64	3.438
406	2	2	0.006	6	1	18.242	3	0	51.546	-6.101	-1.793	0.571	0.71	-1.79	3.537
407	2	2	0.003	6	1	19.83	3	0	55.494	-6.376	-1.755	0.782	0.701	-1.93	3.557
408	3	3	0.845	6	0.973	2.706	4	0.012	11.58	-1.673	2.171	-1.747	0.063	0.095	-0.555
409	3	3	0.859	6	0.999	2.579	1	0.001	17.62	-2.786	2.144	-1.96	-0.216	0.433	-0.648
410	3	3	0.606	6	0.998	4.526	2	0.001	17.584	-3.693	2.072	-2.225	-0.524	0.708	-0.757
411	3	3	0.284	6	0.996	7.413	2	0.004	18.502	-4.38	1.921	-2.546	-0.886	0.915	-0.933
412	3	3	0.139	6	0.997	9.672	2	0.003	21.401	-4.666	1.694	-2.982	-1.317	1.004	-1.182
413	3	3	0.205	6	0.998	8.473	2	0.002	21.248	-4.427	1.673	-2.957	-1.285	1.022	-1.194
414	3	3	0.339	6	0.999	6.806	2	0.001	21.794	-4.022	1.628	-2.986	-1.287	0.993	-1.256
415	3	3	0.667	6	1	4.068	2	0	25.33	-2.944	1.363	-3.204	-1.427	0.779	-1.429
416	3	3	0.679	6	0.999	3.986	6	0	19.447	-1.862	1.094	-3.394	-1.559	0.539	-1.604
417	3	3	0.769	6	0.983	3.31	6	0.015	11.733	-0.979	1.118	-2.935	-1.228	0.057	-1.454
418	3	3	0.667	6	0.793	4.072	6	0.172	7.125	-0.376	1.23	-2.402	-0.83	-0.34	-1.221
419	3	3	0.541	6	0.44	5.018	6	0.375	5.34	-0.214	1.447	-1.752	-0.356	-0.603	-0.919
420	3	4	0.497	6	0.391	5.371	6	0.388	5.39	-0.05	1.692	-1.109	0.127	-0.865	-0.603
421	3	4	0.481	6	0.545	5.507	6	0.187	7.646	-0.491	1.513	-0.652	0.034	-1.082	-1.102

422	3	4	0.289	6	0.587	7.358	3	0.212	9.394	-1.084	1.383	-0.159	-0.03	-1.252	-1.574
423	3	1	0.054	6	0.354	12.387	3	0.349	12.416	-2.256	1.423	0.447	-0.001	-1.216	-1.962
424	3	2	0.015	6	0.649	15.729	1	0.224	17.855	-3.418	1.47	1.08	0.046	-1.211	-2.332
425	3	2	0.026	6	0.82	14.375	1	0.129	18.078	-3.851	1.651	1.082	0.158	-1.08	-2.13
426	3	2	0.031	6	0.837	13.875	1	0.122	17.729	-3.998	1.742	1.041	0.223	-1.063	-1.984
427	3	2	0.033	6	0.729	13.683	1	0.194	16.328	-3.834	1.742	0.892	0.219	-1.116	-1.894
428	3	1	0.031	6	0.424	13.896	3	0.315	14.492	-3.004	1.521	0.58	0.075	-1.374	-1.948
429	3	1	0.361	6	0.899	6.587	4	0.074	11.588	-1.79	1.747	0.503	0.472	-1.853	-1.068
430	3	1	0.541	6	0.931	5.024	4	0.057	10.616	-0.495	1.933	0.442	0.863	-2.386	-0.196
431	3	1	0.594	6	0.995	4.613	4	0.004	15.645	-0.249	2.445	0.61	1.449	-2.568	0.87
432	3	1	0.251	6	1	7.824	4	0	24.209	-0.01	2.947	0.78	2.025	-2.748	1.913
433	3	1	0.172	6	1	9.037	4	0	30.348	-0.974	3.363	0.277	2.156	-2.484	2.661
434	3	1	0.028	6	1	14.133	3	0	39.544	-1.959	3.772	-0.248	2.273	-2.188	3.394
435	3	1	0.001	6	1	23.955	3	0	47.406	-3.032	4.216	-0.721	2.426	-1.892	4.167
436	3	1	0	6	1	34.638	3	0	55.351	-3.621	4.495	-1.314	2.474	-1.745	4.828
437	3	3	0.356	6	1	6.639	6	0	23.518	-2.335	0.108	-3.935	-2.056	1.101	0.1
438	3	3	0.354	6	1	6.654	6	0	23.978	-2.403	0.148	-3.943	-1.999	1.165	0.117
439	3	3	0.366	6	1	6.532	6	0	24.287	-2.479	0.173	-3.92	-1.946	1.2	0.105
440	3	3	0.4	6	1	6.209	6	0	25.73	-2.8	0.23	-3.777	-1.85	1.245	0.084
441	3	3	0.405	6	1	6.164	2	0	24.08	-3.121	0.287	-3.634	-1.754	1.291	0.063
442	3	3	0.469	6	1	5.606	2	0	23.017	-3.119	0.27	-3.554	-1.692	1.213	-0.012
443	3	3	0.601	6	1	4.562	2	0	21.73	-3.037	0.336	-3.436	-1.532	1.08	0.097
444	3	3	0.741	6	1	3.523	6	0	21.089	-2.674	0.269	-3.401	-1.501	0.854	0.077
445	3	3	0.795	6	0.999	3.112	6	0	18.527	-2.353	0.167	-3.411	-1.52	0.666	-0.014
446	3	3	0.818	6	0.999	2.928	6	0.001	16.703	-2.118	0.086	-3.396	-1.523	0.504	-0.084

447	3	3	0.815	6	0.998	2.954	6	0.002	15.143	-1.89	-0.003	-3.38	-1.536	0.349	-0.175
448	3	3	0.831	6	0.997	2.82	6	0.003	14.764	-1.885	-0.055	-3.298	-1.536	0.273	-0.252
449	3	3	0.85	6	0.997	2.66	6	0.003	14.273	-1.878	-0.11	-3.188	-1.528	0.17	-0.332
450	3	3	0.85	6	0.997	2.662	6	0.003	14.274	-1.905	-0.168	-3.131	-1.526	0.094	-0.424
451	3	3	0.838	6	0.997	2.766	6	0.003	14.584	-1.959	-0.232	-3.107	-1.535	-0.039	-0.487
452	3	3	0.798	6	0.996	3.085	6	0.004	14.083	-1.825	-0.279	-3.149	-1.55	-0.191	-0.546
453	3	3	0.753	6	0.993	3.431	6	0.006	13.508	-1.673	-0.307	-3.198	-1.551	-0.326	-0.573
454	3	3	0.792	6	0.995	3.132	6	0.005	13.815	-1.79	-0.279	-3.15	-1.52	-0.37	-0.507
455	3	3	0.813	6	0.996	2.968	6	0.003	14.437	-1.912	-0.262	-3.142	-1.519	-0.307	-0.496
456	3	3	0.792	6	0.997	3.137	6	0.002	15.3	-1.971	-0.261	-3.228	-1.572	-0.089	-0.559
457	3	3	0.753	6	0.998	3.435	6	0.002	16.292	-2.03	-0.259	-3.313	-1.624	0.129	-0.622
458	3	3	0.678	6	0.998	3.992	6	0.001	17.141	-2.005	-0.241	-3.453	-1.686	0.388	-0.658
459	3	3	0.567	6	0.998	4.823	6	0.001	17.876	-1.886	-0.117	-3.69	-1.681	0.816	-0.516
460	3	3	0.455	6	0.999	5.722	6	0.001	19.142	-1.868	-0.03	-3.868	-1.671	1.111	-0.433
461	3	3	0.387	6	0.999	6.329	6	0.001	20.101	-1.879	0.023	-3.969	-1.664	1.276	-0.391
462	3	3	0.273	6	0.998	7.545	6	0.002	19.809	-1.588	-0.021	-4.142	-1.715	1.348	-0.401
463	3	3	0.221	6	0.994	8.244	6	0.006	18.573	-1.274	-0.061	-4.208	-1.725	1.319	-0.398
464	3	3	0.313	6	0.993	7.085	6	0.006	17.16	-1.303	0.015	-4.038	-1.599	1.281	-0.278
465	3	3	0.418	6	0.992	6.046	6	0.008	15.809	-1.324	0.101	-3.87	-1.463	1.24	-0.136
466	3	3	0.609	6	0.99	4.499	6	0.01	13.79	-1.353	0.213	-3.594	-1.287	1.089	-0.003
467	3	3	0.921	6	0.97	1.985	6	0.027	9.173	-1.305	0.344	-2.895	-0.942	0.473	0.219
468	3	3	0.953	6	0.929	1.598	6	0.059	7.096	-1.217	0.428	-2.464	-0.718	0.053	0.372
469	3	3	0.94	6	0.888	1.768	6	0.088	6.394	-1.179	0.492	-2.221	-0.579	-0.188	0.475
470	4	1	0.276	6	0.954	7.518	4	0.038	13.953	-0.045	2.903	1.063	1.158	-0.475	-2.162
471	4	1	0.325	6	0.977	6.956	4	0.02	14.708	-0.354	2.909	1.189	1.196	-0.555	-2.17

472	4	1	0.468	6	0.991	5.61	4	0.008	15.136	-0.601	3.028	1.258	1.282	-0.547	-1.948
473	4	1	0.604	6	0.996	4.541	4	0.003	15.853	-0.847	3.149	1.326	1.367	-0.539	-1.728
474	4	1	0.702	6	0.998	3.816	4	0.002	16.139	-0.903	3.236	1.336	1.413	-0.585	-1.56
475	4	1	0.785	6	0.999	3.188	4	0.001	16.369	-0.927	3.311	1.339	1.454	-0.643	-1.397
476	4	1	0.838	6	0.998	2.764	4	0.002	15.674	-0.837	3.321	1.227	1.428	-0.664	-1.295
477	4	1	0.87	6	0.998	2.488	4	0.002	14.807	-0.662	3.298	1.126	1.395	-0.737	-1.207
478	4	1	0.885	6	0.996	2.345	4	0.003	13.775	-0.425	3.266	0.982	1.353	-0.804	-1.101
479	4	1	0.911	6	0.997	2.094	4	0.003	13.886	-0.446	3.291	0.978	1.362	-0.847	-1.027
480	4	1	0.867	6	0.998	2.511	4	0.002	14.919	-0.5	3.235	1.24	1.4	-1.053	-1.163
481	4	1	0.804	6	0.999	3.036	4	0.001	16.322	-0.631	3.205	1.493	1.446	-1.209	-1.283
482	4	1	0.732	6	0.999	3.588	4	0.001	18.777	-0.947	3.251	1.788	1.538	-1.308	-1.349
483	4	1	0.638	6	1	4.283	4	0	20.832	-1.237	3.292	1.992	1.591	-1.313	-1.421
484	4	1	0.68	6	1	3.977	4	0	19.872	-1.408	3.347	1.792	1.511	-0.923	-1.448
485	4	1	0.663	6	0.999	4.104	4	0.001	19.237	-1.583	3.4	1.567	1.424	-0.507	-1.466
486	4	1	0.59	6	0.999	4.643	4	0.001	19.02	-1.738	3.455	1.34	1.345	-0.098	-1.461
487	4	1	0.5	6	0.999	5.349	4	0.001	18.825	-1.785	3.473	1.168	1.256	0.163	-1.516
488	4	1	0.454	6	0.999	5.728	4	0.001	19.71	-1.933	3.477	1.242	1.235	0.116	-1.578
489	4	1	0.362	6	0.999	6.573	4	0.001	21.536	-2.205	3.51	1.346	1.227	0.109	-1.642
490	4	1	0.398	6	0.999	6.234	4	0.001	19.914	-1.983	3.392	1.332	1.135	-0.053	-1.766
491	4	1	0.437	6	0.998	5.881	4	0.002	18.331	-1.738	3.299	1.284	1.055	-0.186	-1.838
492	4	1	0.546	6	0.996	4.979	4	0.003	16.411	-1.431	3.306	1.105	1.016	-0.191	-1.72
493	4	1	0.629	6	0.995	4.351	4	0.004	15.286	-1.238	3.345	0.957	1	-0.161	-1.578
494	4	1	0.694	6	0.996	3.873	4	0.004	14.928	-1.179	3.421	0.843	1.009	-0.09	-1.41
495	4	1	0.73	6	0.996	3.605	4	0.003	15.064	-1.197	3.515	0.745	1.013	-0.014	-1.271
496	4	1	0.765	6	0.997	3.339	4	0.002	15.404	-1.292	3.539	0.802	0.994	-0.108	-1.232

497	4	1	0.79	6	0.998	3.149	4	0.002	15.773	-1.421	3.554	0.847	0.977	-0.167	-1.168
498	4	1	0.814	6	0.998	2.963	4	0.002	15.248	-1.384	3.507	0.85	0.915	-0.276	-1.16
499	4	1	0.849	6	0.998	2.674	4	0.002	15.013	-1.331	3.488	0.89	0.907	-0.4	-1.128
500	4	1	0.88	6	0.999	2.392	4	0.001	15.558	-1.266	3.538	0.97	1.032	-0.456	-1.096
501	4	1	0.897	6	0.999	2.236	4	0.001	16.147	-1.217	3.575	1.057	1.15	-0.508	-1.081
502	4	1	0.913	6	0.999	2.071	4	0.001	16.819	-1.168	3.618	1.147	1.279	-0.561	-1.038
503	4	1	0.927	6	1	1.914	4	0	17.411	-1.12	3.67	1.182	1.391	-0.593	-0.985
504	4	1	0.952	6	1	1.613	4	0	17.391	-1.085	3.718	1.043	1.433	-0.575	-0.899
505	4	1	0.967	6	1	1.383	4	0	17.191	-0.985	3.758	0.883	1.46	-0.576	-0.833
506	4	1	0.978	6	1	1.175	4	0	17.516	-0.985	3.822	0.757	1.52	-0.548	-0.712
507	4	1	0.978	6	1	1.171	4	0	17.472	-1.005	3.844	0.615	1.513	-0.49	-0.674
508	4	1	0.941	6	0.999	1.758	4	0.001	16.17	-1.078	3.71	0.493	1.277	-0.397	-0.933
509	4	1	0.847	6	0.998	2.689	4	0.002	15.217	-1.15	3.577	0.369	1.041	-0.305	-1.194
510	4	1	0.67	6	0.993	4.048	4	0.005	14.57	-1.215	3.447	0.214	0.795	-0.188	-1.455
511	4	1	0.523	6	0.984	5.167	4	0.011	14.071	-1.165	3.333	0.129	0.618	-0.181	-1.676
512	4	1	0.674	6	0.993	4.021	4	0.006	14.151	-0.975	3.381	0.332	0.781	-0.442	-1.604
513	4	1	0.799	6	0.997	3.081	4	0.002	15.146	-0.933	3.478	0.57	0.973	-0.656	-1.507
514	4	1	0.865	6	0.999	2.531	4	0.001	16.252	-0.811	3.547	0.817	1.157	-0.922	-1.427
515	4	1	0.849	6	0.999	2.673	4	0.001	17.069	-0.687	3.553	0.981	1.25	-1.121	-1.461
516	4	1	0.564	6	0.997	4.843	4	0.003	16.548	-0.548	3.29	0.983	1.023	-1.213	-1.97
517	4	1	0.255	6	0.989	7.775	4	0.01	16.868	-0.427	3.031	0.989	0.799	-1.301	-2.474
518	4	1	0.079	6	0.961	11.321	4	0.037	17.853	-0.343	2.781	0.978	0.575	-1.35	-2.966
519	4	1	0.03	6	0.913	13.935	4	0.081	18.783	-0.323	2.62	0.989	0.436	-1.328	-3.284
520	4	1	0.061	6	0.909	12.056	4	0.083	16.847	-0.158	2.777	0.837	0.517	-1.021	-3.019
521	4	1	0.121	6	0.934	10.092	4	0.059	15.603	-0.159	2.984	0.727	0.631	-0.663	-2.724

522	4	1	0.159	6	0.929	9.264	4	0.062	14.668	0.022	3.141	0.569	0.708	-0.36	-2.466
523	4	1	0.172	6	0.916	9.029	4	0.072	14.122	0.147	3.281	0.385	0.734	-0.044	-2.234
524	4	1	0.147	6	0.876	9.498	4	0.107	13.696	0.055	3.319	0.243	0.565	0.188	-2.171
525	4	1	0.105	6	0.788	10.516	4	0.184	13.424	0.027	3.341	0.054	0.375	0.426	-2.119
526	4	1	0.062	6	0.626	11.996	4	0.321	13.332	0.063	3.347	-0.152	0.173	0.645	-2.081
527	4	1	0.05	6	0.557	12.584	4	0.379	13.354	0.046	3.367	-0.219	0.062	0.742	-2.044
528	4	1	0.117	6	0.775	10.177	4	0.2	12.883	0.172	3.358	0.176	0.261	0.234	-2.06
529	4	1	0.18	6	0.896	8.889	4	0.093	13.428	0.337	3.352	0.555	0.451	-0.283	-2.093
530	4	1	0.223	6	0.956	8.206	4	0.039	14.59	0.473	3.32	0.962	0.663	-0.798	-2.065
531	4	1	0.206	6	0.978	8.458	4	0.019	16.296	0.558	3.287	1.29	0.833	-1.192	-2.081
532	4	1	0.225	6	0.982	8.189	4	0.016	16.477	0.48	3.325	1.261	0.902	-1.09	-2.111
533	4	4	0.143	6	0.593	9.593	1	0.22	11.577	-2.402	1.477	0.942	-0.054	-0.079	1.056
534	4	4	0.423	6	0.844	6.001	1	0.07	10.989	-1.824	1.267	0.78	-0.168	-0.233	0.881
535	4	4	0.737	6	0.914	3.551	3	0.026	10.683	-1.296	1.076	0.647	-0.294	-0.358	0.736
536	4	4	0.959	6	0.883	1.501	6	0.058	6.938	-0.59	0.819	0.415	-0.378	-0.578	0.486
537	4	4	0.986	6	0.744	1.003	6	0.159	4.085	0.173	0.48	0.184	-0.496	-0.871	0.098
538	4	4	0.962	6	0.606	1.454	6	0.267	3.093	0.614	0.285	-0.001	-0.626	-0.942	-0.102
539	4	6	0.849	6	0.445	2.671	4	0.379	2.989	1.214	0.062	-0.252	-0.768	-1.036	-0.275
540	4	6	0.878	6	0.51	2.413	4	0.32	3.347	1.3	0.019	-0.407	-0.833	-0.953	-0.369
541	4	6	0.87	6	0.578	2.49	4	0.243	4.222	1.505	-0.038	-0.549	-0.891	-0.929	-0.492
542	4	6	0.852	6	0.496	2.645	4	0.305	3.618	1.412	-0.02	-0.36	-0.824	-0.933	-0.488
543	4	6	0.811	6	0.403	2.981	4	0.378	3.106	1.307	-0.015	-0.143	-0.744	-0.949	-0.502
544	4	4	0.834	6	0.449	2.793	6	0.319	3.474	1.251	0.015	0.066	-0.703	-0.954	-0.519
545	4	4	0.866	6	0.473	2.524	6	0.31	3.364	1.191	0.09	0.085	-0.658	-1.002	-0.339
546	4	4	0.903	6	0.536	2.178	6	0.264	3.596	1.067	0.161	0.191	-0.612	-1.025	-0.243

547	4	4	0.926	6	0.586	1.933	6	0.221	3.887	0.965	0.208	0.299	-0.557	-1.024	-0.207
548	4	4	0.959	6	0.678	1.502	6	0.16	4.392	0.803	0.31	0.458	-0.558	-0.962	-0.112
549	4	4	0.978	6	0.767	1.18	7	0.121	4.876	0.593	0.418	0.633	-0.552	-0.894	-0.008
550	4	4	0.984	6	0.797	1.03	7	0.108	5.034	0.546	0.525	0.677	-0.573	-0.818	0.124
551	4	4	0.986	6	0.813	0.999	7	0.1	5.191	0.563	0.65	0.694	-0.595	-0.753	0.253
552	4	4	0.985	6	0.854	1.006	7	0.083	5.666	0.393	0.753	0.795	-0.576	-0.639	0.478
553	4	4	0.986	6	0.907	0.981	7	0.054	6.623	0.063	0.749	0.922	-0.673	-0.495	0.488
554	4	4	0.976	6	0.93	1.215	7	0.041	7.469	-0.087	0.689	1.055	-0.765	-0.557	0.369
555	4	4	0.937	6	0.952	1.807	7	0.028	8.834	-0.29	0.602	1.243	-0.867	-0.675	0.218
556	4	4	0.86	6	0.964	2.579	7	0.022	10.168	-0.386	0.507	1.413	-0.972	-0.849	0.019
557	4	4	0.764	6	0.972	3.346	7	0.017	11.448	-0.46	0.432	1.526	-1.085	-0.985	-0.13
558	4	4	0.769	6	0.974	3.312	7	0.014	11.805	-0.577	0.419	1.412	-1.178	-0.941	-0.157
559	4	4	0.764	6	0.975	3.351	6	0.011	12.247	-0.677	0.41	1.302	-1.29	-0.893	-0.177
560	4	4	0.746	6	0.974	3.485	6	0.012	12.223	-0.833	0.387	1.176	-1.334	-0.861	-0.224
561	4	4	0.725	6	0.963	3.645	6	0.015	11.961	-1.132	0.387	0.86	-1.442	-0.628	-0.274
562	4	4	0.801	6	0.943	3.063	6	0.026	10.235	-1.003	0.349	0.568	-1.508	-0.492	-0.256
563	4	4	0.884	6	0.93	2.36	6	0.04	8.655	-0.77	0.341	0.415	-1.505	-0.402	-0.169
564	4	4	0.927	6	0.908	1.924	6	0.057	7.462	-0.636	0.372	0.25	-1.453	-0.322	-0.132
565	4	4	0.953	6	0.883	1.591	6	0.074	6.542	-0.562	0.454	0.089	-1.375	-0.174	-0.068
566	4	4	0.984	6	0.828	1.039	6	0.129	4.761	-0.152	0.453	-0.07	-1.3	-0.086	0.011
567	4	4	0.987	6	0.722	0.972	6	0.222	3.332	0.287	0.452	-0.257	-1.238	0.012	0.113
568	4	4	0.975	6	0.596	1.237	6	0.327	2.44	0.613	0.465	-0.436	-1.138	0.124	0.182
569	4	4	0.962	6	0.477	1.465	6	0.381	1.913	1.118	0.464	-0.349	-0.875	0.039	0.197
570	4	4	0.966	6	0.54	1.402	6	0.282	2.703	1.287	0.522	-0.016	-0.806	-0.027	0.269
571	4	4	0.95	6	0.586	1.63	6	0.208	3.702	1.438	0.46	0.307	-0.834	-0.195	0.163

572	4	4	0.953	6	0.699	1.6	7	0.174	4.38	1.348	0.514	0.752	-0.786	-0.312	0.162
573	4	4	0.95	6	0.822	1.641	7	0.122	5.458	1.093	0.597	1.189	-0.817	-0.311	0.175
574	4	4	0.9	6	0.798	2.202	7	0.136	5.742	1.205	0.438	1.288	-0.827	-0.502	-0.115
575	4	4	0.678	6	0.605	3.992	7	0.221	6.008	1.522	0.135	1.229	-0.633	-0.907	-0.691
576	4	4	0.649	6	0.881	4.207	7	0.076	9.111	1.129	0.295	1.903	-1.148	-0.618	-0.396
577	4	4	0.601	6	0.979	4.562	7	0.015	12.91	0.427	0.546	2.103	-1.725	-0.165	0.206
578	4	4	0.293	6	0.991	7.316	7	0.006	17.627	0.476	0.698	2.211	-2.367	0.098	0.688
579	4	4	0.021	6	0.997	14.932	7	0.001	28.011	0.605	0.986	2.634	-3.214	0.469	1.458
580	4	4	0.042	6	0.996	13.065	7	0.002	25.356	0.712	0.923	2.462	-3.059	0.256	1.377
581	4	4	0.075	6	0.992	11.45	7	0.003	22.815	0.893	0.863	2.215	-2.937	0.113	1.311
582	4	4	0.153	6	0.989	9.377	6	0.005	20.048	0.845	0.854	1.933	-2.752	0.154	1.235
583	4	4	0.268	6	0.986	7.613	6	0.007	17.392	0.736	0.876	1.645	-2.582	0.243	1.176
584	4	4	0.441	6	0.972	5.841	6	0.016	14.002	0.819	0.787	1.286	-2.39	0.233	1.054
585	4	4	0.558	6	0.938	4.888	6	0.036	11.432	1.13	0.718	1.067	-2.119	0.08	1.025
586	4	4	0.76	6	0.923	3.38	6	0.043	9.524	0.951	0.771	0.945	-1.742	0.066	1.01
587	4	4	0.911	6	0.915	2.09	6	0.043	8.184	0.627	0.818	0.861	-1.345	0.091	0.981
588	4	4	0.959	6	0.854	1.512	6	0.076	6.345	0.775	0.838	0.62	-1.081	0.052	0.882
589	4	4	0.985	6	0.77	1.025	6	0.119	4.754	0.87	0.841	0.419	-0.791	-0.08	0.671
590	4	4	0.988	6	0.73	0.939	7	0.134	4.323	0.964	0.639	0.498	-0.708	-0.387	0.376
591	4	4	0.967	6	0.676	1.376	7	0.171	4.12	1.035	0.421	0.587	-0.571	-0.74	0.051
592	4	4	0.886	6	0.643	2.337	7	0.184	4.834	1.186	0.247	0.713	-0.607	-1.011	-0.18
593	4	4	0.7	6	0.618	3.829	7	0.178	6.318	1.257	0.011	0.938	-0.677	-1.276	-0.561
594	4	4	0.488	6	0.476	5.443	5	0.239	6.817	1.501	-0.18	0.879	-0.798	-1.41	-0.871
595	4	5	0.46	6	0.522	5.68	4	0.213	7.468	1.964	-0.359	0.531	-0.954	-1.432	-1.079
596	4	4	0.591	6	0.451	4.639	6	0.253	5.796	1.394	-0.287	0.256	-1.058	-0.952	-1.106

597	4	4	0.554	6	0.396	4.921	6	0.289	5.549	1.482	-0.343	0.135	-1.125	-0.875	-1.111
598	4	4	0.733	6	0.531	3.585	6	0.268	4.95	1.157	-0.245	0.148	-1.125	-0.686	-1.045
599	4	4	0.952	6	0.612	1.602	6	0.229	3.572	0.922	0.056	0.176	-0.883	-0.527	-0.584
600	4	4	0.978	6	0.68	1.175	6	0.18	3.831	0.864	0.168	0.313	-0.899	-0.504	-0.375
601	4	4	0.976	6	0.793	1.213	6	0.102	5.309	0.669	0.189	0.635	-0.958	-0.62	-0.368
602	4	4	0.929	6	0.832	1.901	7	0.08	6.572	0.703	0.145	0.889	-1.051	-0.807	-0.416
603	4	4	0.817	6	0.83	2.931	7	0.076	7.72	0.864	0.056	1.053	-1.181	-0.991	-0.487
604	4	4	0.888	6	0.9	2.323	7	0.048	8.165	0.547	0.176	1.116	-1.271	-0.823	-0.374
605	4	4	0.915	6	0.95	2.046	7	0.025	9.344	0.142	0.344	1.211	-1.417	-0.574	-0.195
606	4	4	0.927	6	0.95	1.925	6	0.024	9.268	0.028	0.339	1.127	-1.391	-0.566	-0.251
607	4	4	0.942	6	0.951	1.738	7	0.024	9.085	-0.054	0.382	1.103	-1.313	-0.523	-0.267
608	4	4	0.943	6	0.947	1.725	7	0.026	8.934	-0.073	0.359	1.079	-1.262	-0.563	-0.312
609	4	4	0.932	6	0.944	1.856	7	0.027	8.952	-0.092	0.315	1.058	-1.224	-0.657	-0.387
610	4	4	0.911	6	0.946	2.094	7	0.026	9.286	-0.192	0.279	1.074	-1.187	-0.738	-0.435
611	4	4	0.894	6	0.948	2.262	7	0.025	9.501	-0.287	0.253	1.085	-1.139	-0.785	-0.464
612	4	4	0.939	6	0.941	1.774	6	0.029	8.754	-0.296	0.309	0.97	-1.073	-0.677	-0.391
613	4	4	0.97	6	0.933	1.337	6	0.033	8.016	-0.309	0.367	0.862	-1.007	-0.551	-0.301
614	4	4	0.987	6	0.92	0.967	6	0.041	7.191	-0.292	0.427	0.72	-0.939	-0.433	-0.271
615	4	4	0.996	6	0.881	0.636	6	0.069	5.727	-0.232	0.531	0.43	-0.797	-0.455	-0.073
616	4	4	0.994	6	0.893	0.716	6	0.062	6.049	-0.34	0.627	0.422	-0.797	-0.405	0.131
617	4	4	0.985	6	0.918	1.004	6	0.044	7.077	-0.543	0.682	0.516	-0.819	-0.34	0.21
618	4	4	0.985	6	0.921	1.011	6	0.044	7.078	-0.513	0.677	0.512	-0.893	-0.341	0.219
619	4	4	0.979	6	0.932	1.156	6	0.037	7.616	-0.555	0.713	0.584	-0.937	-0.328	0.261
620	4	4	0.989	6	0.922	0.909	6	0.043	7.03	-0.219	0.656	0.673	-0.951	-0.483	0.241
621	4	4	0.986	6	0.897	0.989	6	0.055	6.587	0.214	0.58	0.762	-0.968	-0.672	0.206

622	4	4	0.973	6	0.899	1.268	7	0.052	6.986	0.345	0.581	0.932	-0.939	-0.754	0.221
623	4	4	0.94	6	0.902	1.764	7	0.048	7.642	0.348	0.559	0.957	-0.967	-0.965	0.3
624	4	4	0.889	6	0.893	2.308	7	0.052	7.995	0.493	0.542	1.011	-0.972	-1.129	0.374
625	4	4	0.845	6	0.896	2.704	7	0.053	8.362	0.615	0.586	1.111	-0.999	-1.147	0.508
626	4	4	0.769	6	0.827	3.313	7	0.086	7.85	0.964	0.504	1.043	-0.889	-1.324	0.458
627	4	4	0.621	6	0.708	4.415	7	0.131	7.788	1.345	0.409	0.968	-0.792	-1.524	0.396
628	4	4	0.536	6	0.589	5.059	7	0.163	7.632	1.545	0.307	0.855	-0.72	-1.631	0.288
629	4	4	0.371	6	0.38	6.49	5	0.267	7.193	1.888	0.161	0.719	-0.666	-1.772	0.2
630	4	4	0.416	6	0.398	6.064	7	0.218	7.266	1.787	0.235	0.649	-0.484	-1.777	0.18
631	4	4	0.408	6	0.461	6.135	7	0.187	7.941	1.449	0.021	0.712	-0.527	-1.926	-0.473
632	4	4	0.475	6	0.533	5.557	6	0.185	7.678	1.129	-0.128	0.577	-0.646	-1.777	-0.872
633	4	4	0.567	6	0.552	4.819	6	0.249	6.411	0.861	-0.219	0.25	-0.765	-1.462	-1.159
634	4	4	0.576	6	0.569	4.756	6	0.31	5.969	0.465	-0.326	-0.117	-0.945	-1.029	-1.493
635	4	4	0.515	6	0.564	5.23	6	0.34	6.245	0.141	-0.371	-0.366	-1.002	-0.741	-1.69
636	4	4	0.625	6	0.595	4.382	6	0.308	5.701	0.002	-0.267	-0.343	-0.876	-0.705	-1.53
637	4	4	0.752	6	0.649	3.443	6	0.248	5.369	-0.267	-0.12	-0.289	-0.684	-0.64	-1.3
638	4	4	0.812	6	0.583	2.974	6	0.31	4.238	-0.027	-0.051	-0.38	-0.661	-0.668	-1.156
639	4	4	0.635	6	0.605	4.31	6	0.287	5.804	0.16	-0.204	-0.194	-0.779	-0.891	-1.53
640	4	4	0.494	6	0.652	5.397	6	0.25	7.313	0.039	-0.337	-0.119	-0.897	-0.913	-1.77
641	4	4	0.364	6	0.643	6.552	6	0.269	8.294	-0.094	-0.461	-0.257	-1.08	-0.793	-1.968
642	4	4	0.214	6	0.649	8.34	6	0.258	10.181	-0.326	-0.602	-0.351	-1.248	-0.704	-2.224
643	4	4	0.139	6	0.636	9.679	6	0.229	11.722	-0.589	-0.685	-0.436	-1.359	-0.559	-2.37
644	4	4	0.134	6	0.553	9.776	6	0.295	11.031	-0.474	-0.679	-0.649	-1.418	-0.397	-2.365
645	4	4	0.106	6	0.445	10.469	6	0.373	10.82	-0.377	-0.69	-0.916	-1.513	-0.177	-2.4
646	4	6	0.086	6	0.437	11.084	4	0.341	11.579	-0.303	-0.707	-1.178	-1.613	0.05	-2.455

647	4	6	0.358	6	0.496	6.618	4	0.411	6.993	0.094	-0.464	-0.877	-1.249	-0.365	-1.893
648	4	6	0.605	6	0.496	4.532	4	0.408	4.926	0.375	-0.294	-0.683	-0.983	-0.723	-1.469
649	4	6	0.697	6	0.482	3.851	4	0.402	4.215	0.567	-0.17	-0.516	-0.775	-1.09	-1.141
650	4	6	0.729	6	0.492	3.613	4	0.353	4.278	0.866	-0.049	-0.397	-0.572	-1.437	-0.756
651	4	6	0.596	6	0.494	4.597	4	0.295	5.631	1.176	-0.012	-0.282	-0.44	-1.836	-0.523
652	4	6	0.408	6	0.44	6.139	4	0.339	6.661	1.071	-0.078	-0.122	-0.446	-2.145	-0.55
653	4	6	0.221	6	0.377	8.243	4	0.365	8.307	1.02	-0.165	0.063	-0.427	-2.494	-0.615
654	4	4	0.15	6	0.478	9.451	6	0.274	10.564	0.827	-0.187	0.353	-0.449	-2.765	-0.614
655	4	4	0.099	6	0.499	10.678	6	0.373	11.263	0.431	-0.211	0.028	-0.636	-3.03	-0.368
656	4	4	0.113	6	0.478	10.296	6	0.448	10.424	0.188	-0.103	-0.267	-0.724	-2.966	-0.06
657	4	6	0.211	6	0.512	8.386	4	0.428	8.743	0.078	0.092	-0.546	-0.716	-2.641	0.3
658	4	6	0.329	6	0.569	6.913	4	0.371	7.77	-0.015	0.305	-0.854	-0.789	-2.257	0.692
659	4	6	0.358	6	0.57	6.613	4	0.347	7.608	-0.126	0.561	-1.067	-0.83	-1.894	1.133
660	4	6	0.315	6	0.623	7.065	4	0.309	8.468	0.028	0.775	-1.16	-0.731	-1.751	1.584
661	4	6	0.189	6	0.678	8.733	4	0.265	10.612	0.183	1.012	-1.276	-0.652	-1.655	2.131
662	4	6	0.794	6	0.545	3.115	4	0.368	3.9	0.716	1.028	-0.98	-0.641	0.086	1.255
663	4	6	0.884	6	0.609	2.361	4	0.301	3.771	1.075	0.854	-0.992	-0.796	0.109	1.013
664	4	6	0.878	6	0.514	2.41	4	0.396	2.934	0.825	0.858	-0.901	-0.841	0.358	0.826
665	4	4	0.89	6	0.529	2.297	6	0.384	2.936	0.581	0.89	-0.724	-0.886	0.534	0.716
666	4	4	0.965	6	0.618	1.408	6	0.286	2.952	0.407	0.899	-0.486	-0.709	0.383	0.616
667	4	4	0.993	6	0.678	0.747	6	0.212	3.074	0.226	0.885	-0.256	-0.464	0.166	0.46
668	4	4	0.998	6	0.791	0.499	6	0.117	4.323	0.064	0.933	0.109	-0.441	0.057	0.478
669	4	4	0.995	6	0.855	0.687	7	0.073	5.618	-0.111	1.04	0.331	-0.43	0.274	0.516
670	4	4	0.995	6	0.892	0.665	7	0.059	6.089	-0.179	1.022	0.56	-0.493	0.195	0.457
671	4	4	0.99	6	0.927	0.885	7	0.043	7.042	-0.322	0.944	0.861	-0.563	-0.109	0.347

672	4	4	0.956	6	0.953	1.55	7	0.029	8.555	-0.443	0.866	1.177	-0.686	-0.395	0.239
673	4	4	0.803	6	0.974	3.047	7	0.015	11.375	-0.801	0.886	1.509	-0.773	-0.555	0.241
674	4	4	0.885	6	0.963	2.351	7	0.022	9.917	-0.265	0.736	1.351	-0.957	-0.828	0.228
675	4	4	0.814	6	0.914	2.963	6	0.04	9.215	0.587	0.513	1.153	-1.207	-1.222	0.182
676	4	4	0.764	6	0.935	3.347	6	0.031	10.179	0.346	0.548	1.228	-1.176	-1.353	0.28
677	4	4	0.932	6	0.924	1.865	6	0.049	7.734	0.098	0.705	0.702	-1.277	-0.802	0.563
678	4	4	0.957	6	0.907	1.543	6	0.067	6.766	-0.116	0.748	0.366	-1.293	-0.522	0.607
679	4	4	0.954	6	0.902	1.584	6	0.069	6.726	-0.366	0.734	0.208	-1.305	-0.372	0.527
680	4	4	0.937	6	0.887	1.806	6	0.073	6.813	-0.563	0.696	0.069	-1.338	-0.299	0.415
681	4	4	0.919	6	0.852	2.003	6	0.085	6.61	-0.664	0.662	-0.106	-1.362	-0.182	0.317
682	4	4	0.854	6	0.792	2.627	3	0.121	6.378	-0.919	0.773	-0.259	-1.315	0.191	0.355
683	4	4	0.55	6	0.519	4.95	3	0.442	5.271	-1.507	0.959	-0.389	-1.238	0.714	0.409
684	4	4	0.6	6	0.514	4.567	3	0.39	5.12	-1.015	0.817	-0.781	-1.401	0.914	0.245
685	4	4	0.883	6	0.696	2.369	6	0.227	4.611	-0.151	0.92	-0.658	-1.124	0.493	0.666
686	4	4	0.926	6	0.647	1.93	6	0.284	3.575	0.386	0.94	-0.497	-0.935	0.182	0.89
687	4	4	0.919	6	0.555	2.005	6	0.347	2.944	0.871	0.839	-0.39	-0.847	-0.104	0.886
688	4	4	0.864	6	0.533	2.544	6	0.332	3.494	1.233	0.834	-0.165	-0.768	-0.355	0.965
689	4	4	0.727	6	0.47	3.628	6	0.347	4.235	1.64	0.779	-0.031	-0.756	-0.563	0.992
690	4	4	0.757	6	0.472	3.401	6	0.351	3.993	1.655	0.739	-0.044	-0.825	-0.512	0.865
691	4	4	0.736	6	0.44	3.561	6	0.378	3.866	1.78	0.667	-0.085	-0.932	-0.48	0.699
692	4	4	0.864	6	0.517	2.541	6	0.327	3.457	1.51	0.695	-0.062	-0.927	-0.315	0.631
693	4	4	0.939	6	0.644	1.78	6	0.217	3.953	1.256	0.611	0.236	-0.922	-0.541	0.415
694	4	4	0.985	6	0.768	1.021	6	0.131	4.564	0.819	0.68	0.432	-0.835	-0.539	0.413
695	4	4	0.994	6	0.853	0.739	6	0.076	5.585	0.332	0.855	0.552	-0.7	-0.405	0.566
696	4	4	0.991	6	0.867	0.85	7	0.066	6.01	0.182	0.9	0.587	-0.614	-0.393	0.653

697	4	4	0.98	6	0.89	1.14	7	0.057	6.619	-0.026	0.957	0.659	-0.538	-0.341	0.79
698	4	4	0.988	6	0.868	0.945	7	0.065	6.114	-0.036	0.97	0.505	-0.466	-0.282	0.721
699	4	4	0.991	6	0.838	0.846	6	0.082	5.492	-0.087	0.996	0.315	-0.365	-0.187	0.646
700	4	4	0.994	6	0.806	0.718	6	0.106	4.772	0.007	0.989	0.172	-0.381	-0.111	0.594
701	4	4	0.987	6	0.795	0.964	6	0.114	4.856	0.026	1.022	0.165	-0.316	-0.296	0.664
702	4	4	0.986	6	0.722	0.996	6	0.163	3.969	0.328	0.957	0.072	-0.32	-0.427	0.639
703	4	4	0.979	6	0.624	1.156	6	0.23	3.152	0.652	0.911	-0.051	-0.326	-0.446	0.584
704	4	4	0.951	6	0.51	1.623	6	0.306	2.648	0.995	0.854	-0.157	-0.327	-0.445	0.595
705	4	6	0.888	6	0.407	2.318	4	0.374	2.489	1.417	0.729	-0.277	-0.381	-0.515	0.52
706	4	4	0.898	6	0.403	2.221	6	0.376	2.357	1.356	0.61	-0.173	-0.409	-0.648	0.349
707	4	4	0.894	6	0.414	2.264	6	0.358	2.556	1.354	0.449	-0.077	-0.461	-0.797	0.157
708	4	4	0.933	6	0.494	1.85	6	0.297	2.871	1.127	0.414	0.048	-0.451	-0.876	-0.013
709	4	4	0.871	6	0.548	2.473	6	0.238	4.145	1.057	0.279	0.273	-0.458	-1.184	-0.308
710	4	4	0.958	6	0.737	1.52	7	0.132	4.967	0.42	0.42	0.49	-0.357	-1.067	-0.282
711	4	4	0.481	6	0.419	5.501	7	0.253	6.506	1.583	0.267	0.569	-0.28	-1.789	-0.064
712	4	4	0.442	6	0.436	5.831	7	0.253	6.922	1.545	0.254	0.669	-0.25	-1.883	-0.075
713	4	4	0.375	6	0.444	6.448	7	0.252	7.584	1.528	0.244	0.78	-0.211	-2.017	-0.114
714	4	4	0.343	6	0.452	6.765	7	0.252	7.934	1.513	0.256	0.825	-0.186	-2.086	-0.153
715	4	4	0.394	6	0.448	6.263	7	0.256	7.379	1.489	0.244	0.748	-0.188	-1.998	-0.124
716	4	4	0.486	6	0.454	5.459	7	0.261	6.565	1.414	0.228	0.664	-0.18	-1.856	-0.057
717	4	4	0.516	6	0.406	5.217	7	0.263	6.088	1.524	0.245	0.49	-0.228	-1.748	-0.111
718	4	4	0.261	6	0.374	7.702	7	0.25	8.51	1.634	0.228	0.694	-0.169	-2.238	-0.156
719	4	4	0.184	6	0.336	8.814	5	0.25	9.408	1.721	0.219	0.745	-0.141	-2.407	-0.099
720	4	4	0.191	6	0.293	8.704	5	0.261	8.938	1.845	0.245	0.623	-0.146	-2.328	-0.025
721	4	4	0.198	6	0.272	8.583	7	0.258	8.694	1.914	0.273	0.552	-0.133	-2.263	0.065

722	4	7	0.228	6	0.265	8.143	6	0.251	8.251	2	0.301	0.414	-0.133	-2.146	0.158
723	4	6	0.705	6	0.441	3.79	7	0.256	4.875	1.986	0.371	-0.166	-0.315	-1.244	0.163
724	4	6	0.635	6	0.419	4.307	7	0.257	5.284	2.127	0.301	-0.125	-0.331	-1.324	0.073
725	4	6	0.56	6	0.318	4.878	7	0.287	5.084	1.926	0.354	0.171	-0.285	-1.378	-0.012
726	4	4	0.618	6	0.358	4.433	7	0.297	4.809	1.727	0.412	0.313	-0.222	-1.369	0.041
727	4	4	0.656	6	0.397	4.15	7	0.305	4.682	1.625	0.459	0.394	-0.162	-1.361	0.097
728	4	4	0.441	6	0.527	5.847	7	0.26	7.259	1.359	0.302	0.948	-0.135	-1.919	-0.193
729	4	4	0.346	6	0.566	6.734	7	0.237	8.475	1.26	0.245	1.128	-0.135	-2.103	-0.298
730	4	4	0.417	6	0.571	6.057	7	0.239	7.799	1.236	0.265	1.046	-0.146	-1.992	-0.27
731	4	4	0.532	6	0.609	5.09	7	0.233	7.016	1.117	0.322	0.986	-0.135	-1.836	-0.218
732	4	4	0.642	6	0.648	4.258	7	0.218	6.442	0.972	0.379	0.933	-0.127	-1.7	-0.177
733	4	4	0.693	6	0.666	3.882	7	0.207	6.224	0.875	0.409	0.889	-0.116	-1.65	-0.091
734	4	4	0.723	6	0.674	3.656	7	0.201	6.077	0.795	0.418	0.85	-0.094	-1.62	0.024
735	4	4	0.787	6	0.497	3.173	6	0.3	4.18	1.598	0.187	0.136	-1.137	-0.74	-0.283
736	4	4	0.521	6	0.408	5.179	6	0.25	6.161	1.744	-0.063	0.331	-1.167	-1.283	-0.497
737	4	6	0.415	6	0.502	6.069	4	0.429	6.383	0.552	0.64	-0.522	-0.577	-1.875	1.251
738	4	6	0.473	6	0.617	5.57	4	0.319	6.89	0.686	0.693	-0.785	-0.588	-1.731	1.401
739	4	6	0.775	6	0.704	3.261	4	0.221	5.581	1.011	0.831	-1.078	-0.551	-0.859	1.368
740	4	6	0.8	6	0.517	3.071	4	0.393	3.62	0.771	0.565	-0.58	-0.624	-1.245	0.778
741	4	6	0.791	6	0.472	3.138	4	0.436	3.297	0.783	0.441	-0.447	-0.726	-1.323	0.495
742	4	6	0.86	6	0.534	2.576	4	0.376	3.278	0.916	0.444	-0.584	-0.794	-1.168	0.453
743	4	6	0.907	6	0.552	2.132	4	0.363	2.971	0.899	0.466	-0.672	-0.824	-0.957	0.467
744	4	6	0.934	6	0.564	1.843	4	0.355	2.766	0.866	0.472	-0.746	-0.861	-0.771	0.478
745	4	6	0.936	6	0.552	1.819	4	0.369	2.627	0.825	0.461	-0.74	-0.889	-0.745	0.416
746	4	6	0.943	6	0.551	1.726	4	0.373	2.507	0.785	0.45	-0.769	-0.928	-0.673	0.337

747	4	6	0.947	6	0.546	1.681	4	0.381	2.399	0.748	0.447	-0.788	-0.963	-0.589	0.303
748	4	4	0.861	6	0.53	2.568	6	0.389	3.183	0.664	0.333	-0.322	-0.925	-1.188	0.161
749	4	4	0.774	6	0.635	3.276	6	0.283	4.89	0.563	0.26	-0.002	-0.899	-1.572	0.081
750	4	4	0.708	6	0.683	3.769	6	0.24	5.859	0.498	0.216	0.136	-0.919	-1.737	0.057
751	4	4	0.613	6	0.747	4.474	6	0.183	7.289	0.348	0.197	0.321	-0.87	-1.953	-0.006
752	4	4	0.565	6	0.786	4.839	6	0.153	8.11	0.191	0.202	0.393	-0.858	-2.044	-0.047
753	4	4	0.653	6	0.763	4.175	6	0.183	7.033	0.024	0.211	0.165	-0.88	-1.856	-0.052
754	4	4	0.727	6	0.759	3.627	6	0.19	6.402	-0.239	0.267	-0.026	-0.891	-1.63	0.001
755	4	4	0.706	6	0.646	3.786	6	0.294	5.363	-0.118	0.207	-0.298	-0.872	-1.613	-0.033
756	4	6	0.672	6	0.542	4.036	4	0.3	5.219	-0.228	-0.081	-1.261	-1.257	-0.895	-0.578
757	4	6	0.562	6	0.51	4.856	3	0.284	6.029	-0.309	-0.158	-1.604	-1.482	-0.509	-0.834
758	4	6	0.535	6	0.503	5.069	3	0.27	6.311	-0.291	-0.161	-1.552	-1.509	-0.444	-0.963
759	4	6	0.518	6	0.554	5.205	4	0.221	7.049	-0.171	-0.213	-1.546	-1.566	-0.391	-1.097
760	4	6	0.519	6	0.584	5.198	4	0.243	6.95	-0.065	-0.223	-1.479	-1.584	-0.372	-1.183
761	4	6	0.647	6	0.669	4.222	4	0.255	6.151	0.271	-0.237	-1.324	-1.486	-0.545	-1.115
762	4	6	0.727	6	0.724	3.629	4	0.219	6.023	0.713	-0.28	-1.185	-1.37	-0.767	-1.082
763	4	6	0.785	6	0.653	3.186	4	0.276	4.905	0.819	-0.207	-0.935	-1.291	-0.801	-0.923
764	4	6	0.736	6	0.501	3.557	4	0.391	4.053	0.925	-0.096	-0.438	-1.069	-1.275	-0.559
765	4	4	0.544	6	0.437	5.002	6	0.437	5.004	0.947	-0.144	-0.23	-1.109	-1.639	-0.536
766	4	6	0.317	6	0.433	7.047	4	0.422	7.097	0.936	-0.349	-0.213	-1.362	-1.95	-0.743
767	4	4	0.207	6	0.562	8.456	6	0.342	9.452	0.484	-0.446	-0.093	-1.494	-2.203	-0.961
768	4	4	0.145	6	0.694	9.542	6	0.251	11.573	-0.011	-0.467	-0.026	-1.599	-2.33	-1.089
769	4	4	0.13	6	0.662	9.88	6	0.285	11.565	-0.149	-0.48	-0.212	-1.694	-2.297	-1.078
770	4	4	0.127	6	0.63	9.938	6	0.303	11.399	-0.312	-0.445	-0.386	-1.77	-2.235	-1.001
771	4	4	0.1	6	0.545	10.652	6	0.375	11.4	-0.296	-0.487	-0.569	-1.804	-2.303	-0.961

772	4	3	0.02	6	0.391	15.004	6	0.378	15.073	-0.601	-0.902	-1.277	-2.316	-2.019	-1.661
773	4	4	0.978	6	0.808	1.167	6	0.104	5.275	0.106	0.754	0.395	-0.386	-0.917	0.228
774	4	4	0.961	6	0.669	1.477	6	0.208	3.815	0.689	0.623	0.158	-0.543	-1.002	0.117
775	4	4	0.963	6	0.638	1.451	6	0.238	3.421	0.763	0.606	0.069	-0.598	-0.93	0.138
776	4	4	0.967	6	0.618	1.387	6	0.261	3.113	0.776	0.605	-0.013	-0.635	-0.856	0.152
777	4	4	0.928	6	0.565	1.905	6	0.301	3.169	1.008	0.546	-0.024	-0.702	-0.941	0.22
778	4	4	0.772	6	0.44	3.287	6	0.388	3.538	1.473	0.426	-0.073	-0.82	-1.081	0.301
779	4	4	0.875	6	0.549	2.438	6	0.306	3.606	1.158	0.557	0.03	-0.718	-1.036	0.404
780	4	4	0.957	6	0.629	1.54	6	0.271	3.224	0.715	0.653	-0.083	-0.731	-0.831	0.429
781	4	4	0.971	6	0.699	1.304	6	0.221	3.607	0.411	0.69	-0.053	-0.721	-0.82	0.42
782	4	4	0.968	6	0.798	1.368	6	0.139	4.861	0.036	0.747	0.131	-0.65	-0.917	0.402
783	4	4	0.94	6	0.831	1.765	6	0.112	5.779	-0.035	0.697	0.291	-0.651	-1.126	0.356
784	4	4	0.903	6	0.854	2.178	6	0.093	6.603	-0.133	0.648	0.399	-0.654	-1.276	0.324
785	4	4	0.892	6	0.85	2.284	6	0.101	6.549	-0.136	0.661	0.339	-0.702	-1.297	0.36
786	4	4	0.87	6	0.843	2.487	6	0.108	6.593	-0.127	0.681	0.295	-0.72	-1.352	0.403
787	4	4	0.836	6	0.85	2.78	6	0.105	6.956	-0.11	0.713	0.313	-0.765	-1.412	0.514
788	4	4	0.893	6	0.776	2.271	6	0.173	5.27	-0.276	0.556	-0.241	-1.23	-0.917	0.213
789	4	4	0.859	6	0.707	2.586	6	0.181	5.309	-0.538	0.548	-0.519	-1.355	-0.539	0.128
790	4	4	0.855	6	0.679	2.621	6	0.168	5.411	-0.641	0.635	-0.593	-1.315	-0.288	0.233
791	4	4	0.817	6	0.63	2.933	3	0.215	5.084	-0.796	0.752	-0.664	-1.255	-0.013	0.34
792	4	4	0.74	6	0.553	3.529	3	0.326	4.586	-0.973	0.859	-0.725	-1.196	0.246	0.442
793	4	4	0.683	6	0.508	3.954	3	0.4	4.43	-1.124	0.941	-0.703	-1.141	0.358	0.494
794	4	3	0.617	6	0.55	4.444	4	0.4	5.081	-1.413	1.074	-0.66	-1.102	0.581	0.587
795	4	3	0.677	6	0.565	3.996	4	0.363	4.884	-1.242	1.018	-0.861	-1.078	0.669	0.467
796	4	4	0.794	6	0.765	3.119	3	0.131	6.642	-1.038	1.076	-0.227	-0.643	-0.362	0.749

797	4	4	0.855	6	0.798	2.614	6	0.129	6.256	-0.568	0.922	-0.114	-0.529	-0.933	0.693
798	4	4	0.895	6	0.686	2.254	6	0.238	4.376	-0.255	0.692	-0.35	-0.583	-1.034	0.373
799	4	4	0.862	6	0.52	2.56	6	0.41	3.036	0.247	0.466	-0.565	-0.769	-1.151	0.142
800	4	6	0.857	6	0.646	2.597	4	0.282	4.253	0.93	0.181	-0.83	-1.003	-1.285	-0.155
801	4	6	0.875	6	0.691	2.444	4	0.242	4.542	0.993	0.09	-0.952	-1.107	-1.138	-0.334
802	4	6	0.862	6	0.741	2.556	4	0.198	5.197	1.064	-0.023	-1.1	-1.241	-0.985	-0.544
803	4	6	0.828	6	0.762	2.845	4	0.181	5.721	1.039	-0.117	-1.2	-1.342	-0.844	-0.751
804	4	6	0.864	6	0.812	2.542	4	0.133	6.166	1.254	-0.093	-1.387	-1.35	-0.416	-0.803
805	4	6	0.959	6	0.575	1.506	4	0.316	2.705	1.188	0.557	-0.723	-0.779	-0.579	0.265
806	4	6	0.845	6	0.63	2.7	4	0.22	4.803	1.755	0.42	-0.627	-0.805	-1.06	0.286
807	4	6	0.824	6	0.531	2.882	4	0.31	3.959	1.511	0.479	-0.411	-0.676	-1.152	0.388
808	4	6	0.818	6	0.452	2.923	4	0.383	3.253	1.313	0.514	-0.272	-0.576	-1.116	0.471
809	4	4	0.886	6	0.509	2.343	6	0.34	3.149	0.977	0.657	-0.123	-0.459	-1.021	0.541
810	4	4	0.94	6	0.638	1.765	6	0.23	3.808	0.6	0.806	0.035	-0.333	-0.925	0.612
811	4	4	0.965	6	0.714	1.414	6	0.174	4.236	0.312	0.887	0.094	-0.302	-0.798	0.6
812	4	4	0.972	6	0.794	1.294	6	0.118	5.114	-0.067	0.979	0.171	-0.258	-0.652	0.571
813	4	4	0.976	6	0.815	1.219	6	0.104	5.338	-0.156	0.998	0.194	-0.286	-0.586	0.535
814	4	4	0.966	6	0.832	1.403	6	0.087	5.909	-0.263	1.142	0.203	-0.224	-0.375	0.66
815	4	4	0.981	6	0.809	1.105	6	0.106	5.17	-0.079	1.077	0.164	-0.293	-0.398	0.603
816	4	4	0.987	6	0.811	0.953	6	0.109	4.969	-0.052	0.994	0.176	-0.374	-0.493	0.492
817	4	4	0.989	6	0.746	0.904	6	0.163	3.95	0.324	0.789	0.115	-0.541	-0.715	0.318
818	4	4	0.965	6	0.65	1.415	6	0.24	3.407	0.737	0.597	0.038	-0.701	-0.898	0.16
819	4	4	0.904	6	0.507	2.167	6	0.359	2.859	1.157	0.498	-0.143	-0.809	-0.88	0.143
820	4	6	0.84	6	0.503	2.747	4	0.335	3.561	1.628	0.382	-0.363	-0.934	-0.845	0.128
821	4	6	0.821	6	0.587	2.899	4	0.239	4.701	1.924	0.337	-0.527	-1.008	-0.781	0.121

822	4	6	0.709	6	0.617	3.763	4	0.161	6.451	2.257	0.222	-0.605	-1.101	-0.904	0.024
823	4	4	0.924	6	0.561	1.954	6	0.308	3.152	1.213	0.631	-0.046	-0.861	-0.671	0.354
824	4	4	0.992	6	0.764	0.803	6	0.139	4.211	0.677	0.846	0.288	-0.722	-0.47	0.393
825	4	4	0.995	6	0.793	0.665	6	0.116	4.506	0.637	0.822	0.387	-0.756	-0.432	0.314
826	4	4	0.998	6	0.827	0.518	6	0.098	4.793	0.511	0.766	0.442	-0.85	-0.397	0.191
827	4	4	0.998	6	0.834	0.491	6	0.095	4.827	0.481	0.681	0.449	-0.925	-0.392	0.028
828	4	4	0.997	6	0.846	0.541	6	0.09	5.027	0.426	0.614	0.471	-0.988	-0.41	-0.092
829	4	4	0.989	6	0.844	0.906	6	0.089	5.397	0.484	0.527	0.551	-0.998	-0.643	-0.183
830	4	4	0.964	6	0.843	1.429	6	0.088	5.952	0.543	0.442	0.636	-1.017	-0.871	-0.268
831	4	4	0.908	6	0.834	2.121	6	0.09	6.576	0.602	0.349	0.703	-1.007	-1.114	-0.376
832	4	4	0.747	6	0.847	3.479	6	0.071	8.423	0.604	0.255	0.932	-0.955	-1.49	-0.496
833	4	4	0.563	6	0.792	4.851	6	0.09	9.209	0.815	0.116	0.928	-0.975	-1.755	-0.634
834	4	4	0.483	6	0.725	5.484	6	0.119	9.093	0.939	0.009	0.802	-1.009	-1.844	-0.724
835	4	4	0.259	6	0.509	7.722	5	0.237	9.25	1.341	-0.166	0.653	-1.107	-2.079	-0.927
836	4	5	0.217	6	0.651	8.294	6	0.153	11.188	2.018	-0.361	0.308	-1.256	-2.259	-1.118
837	4	6	0.943	6	0.688	1.726	4	0.225	3.961	1.329	0.526	-0.937	-0.859	-0.801	0.352
838	4	6	0.926	6	0.628	1.927	4	0.281	3.535	1.2	0.609	-0.816	-0.8	-0.869	0.413
839	4	6	0.881	6	0.464	2.391	4	0.448	2.459	0.713	0.754	-0.6	-0.657	-0.859	0.54
840	4	4	0.918	6	0.683	2.019	6	0.238	4.131	-0.006	0.997	-0.335	-0.459	-0.738	0.723
841	4	4	0.922	6	0.659	1.977	6	0.256	3.868	0.162	0.947	-0.292	-0.435	-0.793	0.738
842	4	4	0.923	6	0.651	1.959	6	0.253	3.852	0.248	0.91	-0.222	-0.378	-0.79	0.804
843	4	4	0.898	6	0.539	2.22	6	0.342	3.126	0.68	0.884	-0.309	-0.411	-0.828	0.755
844	4	4	0.642	6	0.57	4.258	6	0.297	5.564	0.96	0.631	0.101	-0.497	-1.768	0.43
845	4	4	0.374	6	0.546	6.457	6	0.288	7.74	1.105	0.412	0.279	-0.534	-2.282	0.181
846	4	4	0.252	6	0.44	7.816	6	0.337	8.348	1.322	0.252	0.212	-0.598	-2.461	-0.004

847	4	4	0.837	6	0.656	2.767	6	0.291	4.39	1.053	0.813	-0.235	-1.485	-0.343	0.726
848	4	4	0.91	6	0.723	2.105	6	0.23	4.397	0.786	0.886	-0.209	-1.43	-0.067	0.693
849	4	4	0.831	6	0.781	2.822	6	0.181	5.749	0.805	0.982	-0.148	-1.644	0.351	0.835
850	4	4	0.734	6	0.864	3.572	6	0.11	7.689	0.678	1.062	0.038	-1.842	0.606	0.989
851	4	4	0.689	6	0.905	3.911	6	0.074	8.906	0.845	0.986	0.405	-2.041	0.145	1.013
852	4	4	0.519	6	0.931	5.198	6	0.051	11	1.065	0.907	0.785	-2.228	-0.358	1.047
853	4	4	0.306	6	0.956	7.16	6	0.028	14.216	1.132	0.859	1.211	-2.405	-0.808	1.097
854	4	4	0.106	6	0.97	10.482	6	0.014	18.903	1.228	0.798	1.652	-2.705	-1.218	1.15
855	4	4	0.182	6	0.926	8.856	6	0.06	14.324	0.331	0.056	0.598	-2.844	-1.658	-0.193
856	4	4	0.13	6	0.497	9.87	6	0.444	10.092	0.561	-0.473	-0.537	-2.295	-1.744	-1.141
857	4	6	0.829	6	0.828	2.835	4	0.131	6.53	0.851	0.342	-1.838	-1.581	0.154	-0.18
858	4	6	0.876	6	0.61	2.43	4	0.312	3.769	0.369	0.733	-1.394	-1.203	0.161	0.257
859	4	6	0.838	6	0.5	2.763	4	0.44	3.02	0.565	0.965	-0.998	-1.074	0.008	0.709
860	4	4	0.868	6	0.632	2.5	6	0.309	3.931	0.587	1.158	-0.551	-1.045	0.03	0.92
861	4	4	0.871	6	0.66	2.474	6	0.278	4.199	0.872	1.052	-0.283	-1.137	-0.272	0.927
862	4	4	0.83	6	0.69	2.832	6	0.255	4.824	0.982	0.874	-0.099	-1.329	-0.632	0.87
863	4	4	0.667	6	0.656	4.069	6	0.283	5.753	1.265	0.658	0.007	-1.541	-1.054	0.709
864	4	4	0.371	6	0.595	6.481	6	0.304	7.823	1.627	0.459	0.126	-1.797	-1.477	0.62
865	4	6	0.544	6	0.601	5	4	0.338	6.152	0.667	1.134	-0.959	-0.581	-1.211	1.451
866	4	6	0.658	6	0.639	4.138	4	0.305	5.62	0.578	1.128	-1.178	-0.723	-0.86	1.329
867	4	6	0.693	6	0.577	3.881	4	0.361	4.818	0.386	1.106	-1.137	-0.782	-0.735	1.182
868	4	6	0.726	6	0.524	3.632	4	0.405	4.146	0.236	1.052	-1.106	-0.87	-0.653	0.984
869	4	4	0.272	6	0.657	7.56	6	0.278	9.28	0.633	-0.103	0.015	-1.706	-2.118	-0.713
870	4	4	0.234	6	0.587	8.06	6	0.35	9.091	0.604	-0.154	-0.187	-1.717	-2.13	-0.831
871	4	4	0.293	6	0.514	7.307	6	0.431	7.661	0.629	-0.132	-0.416	-1.721	-1.862	-0.864

872	4	6	0.357	6	0.516	6.624	4	0.433	6.975	0.645	-0.123	-0.648	-1.72	-1.629	-0.923
873	4	6	0.445	6	0.58	5.805	4	0.375	6.677	0.651	-0.101	-0.843	-1.752	-1.385	-0.918
874	4	6	0.581	6	0.723	4.71	4	0.231	6.99	0.67	-0.036	-1.423	-1.893	-0.436	-1.001
875	4	6	0.601	6	0.784	4.56	4	0.174	7.567	0.856	0.076	-1.658	-1.891	0.141	-0.946
876	4	6	0.698	6	0.783	3.844	4	0.176	6.827	1.077	0.248	-1.592	-1.714	0.418	-0.771
877	4	6	0.657	6	0.819	4.146	4	0.137	7.726	1.504	0.4	-1.639	-1.624	0.806	-0.545
878	4	6	0.6	6	0.84	4.571	4	0.1	8.833	2.001	0.432	-1.599	-1.506	0.892	-0.451
879	4	6	0.663	6	0.684	4.101	4	0.222	6.349	1.954	0.524	-1.035	-1.52	0.656	-0.325
880	4	6	0.632	6	0.454	4.334	4	0.431	4.434	1.831	0.666	-0.485	-1.51	0.488	-0.152
881	4	4	0.619	6	0.553	4.43	6	0.305	5.621	2.024	0.7	-0.008	-1.559	0.23	-0.023
882	4	4	0.487	6	0.688	5.455	6	0.122	8.916	2.059	0.457	0.769	-1.767	-0.464	-0.381
883	4	4	0.347	6	0.758	6.728	5	0.131	10.241	1.961	0.371	1.254	-1.95	-0.735	-0.51
884	4	4	0.264	6	0.789	7.664	5	0.131	11.255	1.978	0.379	1.447	-2.141	-0.723	-0.449
885	4	4	0.248	6	0.876	7.865	5	0.073	12.825	1.808	0.45	1.698	-2.252	-0.65	-0.35
886	4	4	0.227	6	0.94	8.158	5	0.032	14.935	1.544	0.51	1.938	-2.398	-0.576	-0.252
887	4	4	0.181	6	0.956	8.866	5	0.024	16.261	1.395	0.483	1.997	-2.593	-0.653	-0.249
888	4	4	0.133	6	0.963	9.81	5	0.022	17.403	1.317	0.444	2.032	-2.79	-0.735	-0.255
889	4	4	0.105	6	0.982	10.494	5	0.01	19.76	1.023	0.47	2.163	-2.952	-0.76	-0.199
890	4	4	0.055	6	0.995	12.336	6	0.002	24.609	0.37	0.479	2.184	-3.419	-0.682	-0.135
891	4	4	0.089	6	0.989	10.995	6	0.006	21.375	0.471	0.283	1.784	-3.314	-0.858	-0.388
892	4	4	0.169	6	0.964	9.085	6	0.019	16.98	0.862	0.199	1.323	-3.083	-0.857	-0.431
893	4	4	0.321	6	0.951	6.998	6	0.032	13.784	0.861	0.269	1.026	-2.815	-0.712	-0.417
894	4	4	0.517	6	0.92	5.215	6	0.06	10.668	0.83	0.321	0.667	-2.524	-0.622	-0.42
895	4	4	0.601	6	0.891	4.561	6	0.09	9.146	0.725	0.346	0.39	-2.415	-0.635	-0.36
896	4	4	0.66	6	0.841	4.121	6	0.139	7.721	0.581	0.353	0.075	-2.286	-0.68	-0.346

897	4	4	0.634	6	0.798	4.315	6	0.181	7.279	0.474	0.352	-0.158	-2.31	-0.692	-0.258
898	4	4	0.72	6	0.663	3.681	6	0.31	5.2	0.795	0.78	-0.541	-1.996	-0.09	0.48
899	4	4	0.627	6	0.571	4.366	6	0.396	5.097	0.992	0.967	-0.715	-1.822	0.153	0.842
900	4	4	0.616	6	0.538	4.446	6	0.426	4.916	1.047	0.975	-0.748	-1.754	0.14	0.896
901	4	4	0.575	6	0.518	4.759	6	0.444	5.069	1.082	1.027	-0.79	-1.712	0.222	1.002
902	4	4	0.61	6	0.564	4.496	6	0.398	5.192	0.964	1.071	-0.724	-1.644	0.207	1.091
903	4	4	0.633	6	0.549	4.324	6	0.412	4.901	1.178	0.897	-0.571	-1.722	-0.257	0.916
904	4	4	0.536	6	0.523	5.06	6	0.427	5.464	1.455	0.669	-0.378	-1.811	-0.839	0.67
905	4	4	0.657	6	0.718	4.147	6	0.233	6.397	1.112	1.175	-0.079	-1.382	-0.489	1.303
906	4	4	0.831	6	0.86	2.82	6	0.108	6.963	0.395	1.253	0.145	-1.233	-0.463	1.185
907	4	4	0.871	6	0.863	2.474	6	0.105	6.678	0.423	1.144	0.21	-1.284	-0.623	0.967
908	4	4	0.812	6	0.911	2.975	6	0.067	8.21	0.265	0.786	0.562	-1.474	-1.26	0.328
909	4	4	0.739	6	0.915	3.54	6	0.066	8.812	0.175	0.651	0.572	-1.555	-1.442	0.131
910	4	4	0.789	6	0.88	3.152	6	0.098	7.549	0.217	0.706	0.306	-1.559	-1.294	0.275
911	4	4	0.822	6	0.821	2.897	6	0.153	6.257	0.258	0.74	-0.003	-1.551	-1.111	0.398
912	4	4	0.809	6	0.75	2.997	6	0.22	5.452	0.28	0.761	-0.272	-1.546	-0.993	0.5
913	4	4	0.76	6	0.694	3.381	6	0.273	5.249	0.252	0.688	-0.428	-1.57	-1.063	0.473
914	4	4	0.688	6	0.637	3.917	6	0.324	5.269	0.172	0.584	-0.584	-1.624	-1.145	0.423
915	4	4	0.58	6	0.558	4.725	6	0.395	5.416	0.141	0.473	-0.753	-1.681	-1.303	0.275
916	4	6	0.7	6	0.611	3.828	4	0.344	4.976	0.544	0.929	-1.209	-1.289	-0.654	1.014
917	4	6	0.675	6	0.769	4.016	4	0.196	6.753	1.087	1.041	-1.45	-1.205	-0.486	1.273
918	4	6	0.669	6	0.828	4.059	4	0.139	7.622	1.47	0.951	-1.477	-1.244	-0.662	1.181
919	4	4	0.358	6	0.584	6.61	6	0.331	7.745	0.96	0.479	0.04	-0.984	-2.288	0.25
920	4	4	0.274	6	0.575	7.543	6	0.352	8.523	0.844	0.414	-0.026	-1.056	-2.472	0.311
921	4	6	0.145	6	0.693	9.555	4	0.165	12.431	0.488	1.244	-2.161	-2.097	0.523	1.646

922	4	6	0.132	6	0.795	9.834	4	0.157	13.082	0.939	1.451	-2.124	-1.867	0.917	1.728
923	4	6	0.165	6	0.662	9.165	4	0.28	10.882	0.749	1.571	-1.765	-1.639	1.012	1.69
924	4	4	0.279	6	0.522	7.482	6	0.404	7.995	0.353	1.603	-1.251	-1.481	0.791	1.501
925	4	4	0.603	6	0.689	4.55	6	0.256	6.53	0.205	1.472	-0.78	-1.316	0.475	1.24
926	4	4	0.841	6	0.8	2.734	6	0.155	6.017	0.02	1.334	-0.368	-1.154	0.154	1.035
927	4	4	0.89	6	0.824	2.302	6	0.129	6.008	-0.157	1.272	-0.259	-1.075	-0.097	0.91
928	4	4	0.899	6	0.856	2.211	6	0.094	6.635	-0.409	1.231	-0.101	-0.978	-0.35	0.8
929	4	4	0.912	6	0.852	2.084	6	0.1	6.371	-0.392	1.11	-0.048	-0.928	-0.665	0.63
930	4	4	0.885	6	0.839	2.345	6	0.103	6.547	-0.531	0.977	-0.045	-0.842	-0.947	0.384
931	4	4	0.88	6	0.797	2.395	6	0.157	5.642	-0.172	0.887	-0.141	-0.974	-1.1	0.402
932	4	4	0.82	6	0.673	2.914	6	0.282	4.656	0.369	0.846	-0.321	-1.069	-1.205	0.543
933	4	4	0.729	6	0.636	3.615	6	0.318	5.004	0.517	0.917	-0.358	-1.063	-1.275	0.812
934	4	4	0.643	6	0.547	4.247	6	0.406	4.842	0.753	0.958	-0.503	-1.099	-1.21	0.99
935	4	4	0.676	6	0.549	4.008	6	0.406	4.61	0.715	1.101	-0.643	-1.171	-0.674	1.181
936	4	4	0.56	6	0.542	4.875	6	0.416	5.404	0.705	1.255	-0.815	-1.293	-0.066	1.446
937	4	4	0.387	6	0.488	6.334	6	0.462	6.441	0.628	1.446	-1.089	-1.243	0.49	1.587
938	4	4	0.687	6	0.683	3.924	6	0.268	5.799	0.37	1.379	-0.694	-1.243	0.514	1.158
939	4	4	0.854	6	0.765	2.63	6	0.191	5.406	0.278	1.281	-0.441	-1.255	0.378	0.877
940	4	4	0.924	6	0.838	1.955	6	0.124	5.771	0.098	1.249	-0.182	-1.242	0.138	0.757
941	4	4	0.967	6	0.837	1.377	6	0.126	5.159	0.184	1.083	-0.055	-1.216	-0.221	0.535
942	4	4	0.977	6	0.837	1.206	6	0.124	5.027	0.24	0.944	0.071	-1.168	-0.535	0.33
943	4	4	0.962	6	0.799	1.456	6	0.15	4.797	0.52	0.816	0.145	-1.118	-0.812	0.223
944	4	4	0.864	6	0.719	2.539	6	0.2	5.099	0.96	0.624	0.236	-1.076	-1.198	0.081
945	4	4	0.832	6	0.774	2.813	6	0.145	6.164	0.853	0.641	0.464	-0.954	-1.397	0.035
946	4	4	0.954	6	0.743	1.586	6	0.19	4.311	0.616	0.747	0.082	-0.966	-0.951	0.089

947	4	4	0.952	6	0.663	1.605	6	0.271	3.394	0.658	0.729	-0.203	-1.032	-0.784	0.058
948	4	4	0.932	6	0.592	1.857	6	0.345	2.934	0.67	0.691	-0.417	-1.093	-0.722	-0.003
949	4	4	0.886	6	0.509	2.34	6	0.433	2.662	0.699	0.619	-0.624	-1.175	-0.715	-0.085
950	4	6	0.877	6	0.508	2.42	4	0.436	2.726	0.743	0.59	-0.794	-1.21	-0.663	-0.132
951	4	6	0.909	6	0.571	2.111	4	0.363	3.019	1.053	0.625	-0.821	-1.134	-0.624	-0.033
952	4	6	0.921	6	0.616	1.991	4	0.302	3.416	1.332	0.685	-0.846	-1.044	-0.551	0.08
953	4	6	0.896	6	0.684	2.246	4	0.213	4.575	1.759	0.693	-0.902	-0.981	-0.512	0.183
954	4	6	0.772	6	0.691	3.285	4	0.168	6.115	2.136	0.604	-0.782	-0.927	-0.891	0.228
955	4	6	0.545	6	0.547	4.993	4	0.403	5.602	0.653	1.343	-1.042	-0.928	-0.61	1.447
956	4	4	0.477	6	0.5	5.533	6	0.44	5.787	0.468	1.544	-0.955	-0.816	-0.261	1.571
957	4	4	0.422	6	0.533	6.013	6	0.403	6.572	0.503	1.689	-0.912	-0.74	0.039	1.703
958	4	4	0.648	6	0.679	4.213	6	0.262	6.12	0.187	1.578	-0.645	-0.832	-0.117	1.389
959	4	4	0.794	6	0.738	3.115	6	0.213	5.597	0.203	1.424	-0.444	-0.964	-0.265	1.145
960	4	4	0.873	6	0.805	2.462	6	0.154	5.775	0.159	1.346	-0.209	-1.029	-0.343	0.996
961	4	4	0.919	6	0.797	2.004	6	0.163	5.178	0.474	1.131	-0.061	-1.197	-0.547	0.722
962	4	4	0.865	6	0.721	2.528	6	0.227	4.844	0.995	0.904	-0.026	-1.37	-0.727	0.508
963	4	4	0.878	6	0.729	2.411	6	0.223	4.783	0.976	0.993	-0.1	-1.431	-0.417	0.582
964	4	4	0.857	6	0.731	2.599	6	0.224	4.965	0.952	1.11	-0.204	-1.478	-0.054	0.696
965	4	4	0.759	6	0.704	3.383	6	0.251	5.45	1.012	1.222	-0.358	-1.538	0.324	0.83
966	4	4	0.909	6	0.855	2.108	6	0.115	6.119	0.76	0.946	0.152	-1.756	-0.053	0.245
967	4	4	0.87	6	0.904	2.49	6	0.073	7.531	0.704	0.764	0.482	-1.908	-0.421	-0.052
968	4	4	0.792	6	0.914	3.135	6	0.064	8.445	0.711	0.664	0.612	-1.981	-0.719	-0.154
969	4	4	0.627	6	0.944	4.366	6	0.04	10.701	0.665	0.611	0.893	-2.167	-0.977	-0.168
970	4	4	0.477	6	0.964	5.534	6	0.025	12.808	0.557	0.623	1.081	-2.373	-1.112	-0.112
971	4	4	0.421	6	0.949	6.021	6	0.041	12.328	0.66	0.567	0.803	-2.614	-1.021	0.035

972	4	4	0.309	6	0.925	7.133	6	0.063	12.51	0.805	0.483	0.569	-2.87	-0.988	0.214
973	4	6	0.788	6	0.623	3.166	4	0.294	4.669	0.874	1.35	-1.109	-0.422	-0.549	0.986
974	4	6	0.802	6	0.626	3.054	4	0.289	4.601	0.951	1.337	-1.084	-0.435	-0.579	0.926
975	4	6	0.806	6	0.504	3.021	4	0.396	3.504	1.015	1.122	-0.654	-0.502	-1.029	0.48
976	4	4	0.815	6	0.498	2.95	6	0.404	3.367	0.824	1.068	-0.46	-0.519	-1.138	0.315
977	4	4	0.868	6	0.574	2.508	6	0.341	3.552	0.501	1.138	-0.456	-0.496	-1.018	0.361
978	4	4	0.82	6	0.473	2.908	6	0.442	3.042	0.75	1.07	-0.604	-0.582	-1.042	0.302
979	4	6	0.832	6	0.573	2.81	4	0.345	3.823	1.077	0.965	-0.774	-0.697	-1.145	0.19
980	4	6	0.806	6	0.587	3.024	4	0.34	4.114	1.023	0.901	-0.796	-0.756	-1.277	0.198
981	4	6	0.766	6	0.584	3.336	4	0.351	4.351	0.902	0.84	-0.802	-0.802	-1.418	0.203
982	4	6	0.689	6	0.618	3.912	4	0.324	5.205	0.97	0.721	-0.814	-0.918	-1.641	0.153
983	4	6	0.805	6	0.79	3.028	4	0.167	6.136	1.21	0.903	-1.337	-0.935	-1.154	0.611
984	4	6	0.582	6	0.59	4.709	4	0.345	5.785	0.465	1.461	-1.238	-0.68	-0.715	1.252
985	4	6	0.549	6	0.586	4.958	4	0.357	5.949	0.591	1.458	-1.144	-0.705	-0.827	1.328
986	4	6	0.518	6	0.576	5.201	4	0.368	6.099	0.649	1.45	-1.061	-0.679	-0.977	1.357
987	4	6	0.489	6	0.581	5.439	4	0.361	6.389	0.76	1.453	-1.002	-0.639	-1.079	1.406
988	4	6	0.478	6	0.587	5.529	4	0.353	6.543	0.815	1.536	-1.036	-0.61	-0.902	1.487
989	4	6	0.429	6	0.563	5.951	4	0.372	6.783	0.771	1.661	-1.049	-0.545	-0.701	1.603
990	4	6	0.421	6	0.651	6.018	4	0.285	7.667	1.096	1.677	-1.166	-0.583	-0.575	1.676
991	4	6	0.539	6	0.702	5.04	4	0.227	7.298	1.606	1.482	-1.093	-0.749	-0.639	1.341
992	4	6	0.57	6	0.558	4.797	4	0.367	5.637	1.197	1.531	-0.895	-0.716	-0.498	1.317
993	4	4	0.566	6	0.576	4.83	6	0.356	5.794	0.56	1.652	-0.691	-0.631	-0.366	1.456
994	4	4	0.569	6	0.541	4.806	6	0.392	5.449	0.679	1.602	-0.715	-0.68	-0.465	1.395
995	4	4	0.555	6	0.502	4.914	6	0.434	5.209	0.837	1.548	-0.736	-0.761	-0.567	1.341
996	4	4	0.602	6	0.484	4.552	6	0.453	4.681	0.823	1.466	-0.758	-0.766	-0.673	1.186

997	4	6	0.649	6	0.479	4.207	4	0.459	4.291	0.818	1.376	-0.785	-0.775	-0.803	1.011
998	4	6	0.675	6	0.479	4.009	4	0.46	4.088	0.758	1.298	-0.777	-0.781	-0.92	0.895
999	4	4	0.791	6	0.499	3.138	6	0.439	3.397	0.615	1.236	-0.766	-0.814	-0.735	0.639
1000	4	6	0.86	6	0.549	2.575	4	0.389	3.263	0.857	1.086	-0.923	-0.902	-0.644	0.428
1001	4	6	0.918	6	0.719	2.02	4	0.224	4.356	1.167	0.962	-1.252	-0.949	-0.506	0.318
1002	4	6	0.902	6	0.785	2.184	4	0.169	5.257	1.302	0.945	-1.465	-1.091	-0.253	0.326
1003	4	6	0.866	6	0.82	2.523	4	0.139	6.07	1.395	0.941	-1.614	-1.2	-0.024	0.344
1004	4	6	0.797	6	0.856	3.093	4	0.106	7.27	1.713	0.9	-1.677	-1.312	0.065	0.333
1005	4	6	0.641	6	0.901	4.266	4	0.065	9.529	2.171	0.793	-1.799	-1.482	0.115	0.267
1006	4	6	0.528	6	0.62	5.123	4	0.269	6.794	2.222	1.205	-0.815	-1.234	0.288	0.692
1007	4	4	0.482	6	0.488	5.5	6	0.4	5.895	2.072	1.223	-0.311	-1.389	0.151	0.689
1008	4	4	0.615	6	0.729	4.457	6	0.18	7.251	1.852	1.225	0.289	-1.485	-0.081	0.669
1009	4	4	0.586	6	0.851	4.679	6	0.085	9.286	1.738	1.249	0.733	-1.63	-0.161	0.734
1010	4	4	0.58	6	0.908	4.722	6	0.052	10.459	1.396	1.504	0.784	-1.627	0.229	1.037
101	4	4	0.443	6	0.957	5.827	6	0.023	13.278	0.834	1.857	0.869	-1.601	0.71	1.405

1															
1012	4	4	0.307	6	0.945	7.154	6	0.03	14.048	1.053	1.895	0.775	-1.639	0.872	1.634
1013	4	4	0.231	6	0.929	8.095	6	0.04	14.384	1.351	1.767	0.687	-1.814	1.226	1.531
1014	4	4	0.192	6	0.949	8.688	6	0.027	15.809	1.199	1.848	0.78	-1.882	1.476	1.472
1015	4	4	0.196	6	0.973	8.62	7	0.015	17	0.882	1.937	1.07	-1.892	1.499	1.397
1016	4	4	0.19	6	0.975	8.727	7	0.014	17.154	1.057	1.863	1.268	-2.004	1.382	1.301
1017	4	4	0.174	6	0.975	8.997	7	0.015	17.34	1.275	1.769	1.391	-2.13	1.287	1.188
1018	4	4	0.132	6	0.98	9.839	7	0.012	18.644	1.286	1.746	1.453	-2.307	1.38	1.15
1019	4	4	0.1	6	0.981	10.64	7	0.011	19.666	1.403	1.673	1.479	-2.503	1.431	1.054
1020	4	4	0.062	6	0.989	11.991	7	0.006	22.177	1.119	1.746	1.612	-2.668	1.656	1.106
1021	4	4	0.039	6	0.994	13.266	7	0.004	24.458	0.889	1.808	1.959	-2.747	1.712	1.092
1022	4	4	0.015	6	0.997	15.723	7	0.002	28.282	0.593	1.888	2.235	-2.919	1.839	1.249
1023	4	4	0.004	6	0.999	19.318	7	0.001	33.73	0.077	2.015	2.459	-3.105	2.056	1.507
102	4	4	0.002	6	0.999	20.622	7	0.001	35.452	0.207	1.988	2.421	-3.362	2.069	1.544

4																
1025	4	4	0.001	6	0.999	23.012	7	0	38.501	0.349	1.988	2.477	-3.675	2.071	1.628
1026	4	4	0.001	6	0.999	21.884	7	0	37.14	0.494	1.882	2.531	-3.697	1.822	1.521
1027	4	4	0.002	6	0.999	21.452	7	0.001	36.612	0.636	1.785	2.632	-3.752	1.583	1.421
1028	4	4	0.003	6	0.999	19.666	7	0.001	34.265	0.792	1.64	2.658	-3.688	1.283	1.232
1029	4	4	0.007	6	0.998	17.847	7	0.001	31.73	0.973	1.581	2.399	-3.617	1.012	1.313
1030	4	4	0.012	6	0.997	16.329	6	0.001	29.423	1.031	1.501	2.226	-3.549	0.772	1.321
1031	4	4	0.015	6	0.996	15.798	6	0.002	28.204	1.139	1.372	2.132	-3.601	0.541	1.241
1032	4	4	0.049	6	0.995	12.643	6	0.003	24.314	0.951	1.211	1.971	-3.375	0.236	0.99
1033	4	4	0.138	6	0.993	9.701	6	0.004	20.633	0.727	1.079	1.8	-3.063	-0.017	0.814
1034	4	4	0.223	6	0.988	8.213	6	0.008	17.9	0.858	1.074	1.556	-2.879	-0.132	0.734
1035	4	4	0.349	6	0.975	6.706	6	0.016	14.871	1.022	1.061	1.261	-2.652	-0.287	0.588
1036	4	4	0.38	6	0.97	6.403	6	0.02	14.143	1.047	1.047	1.178	-2.589	-0.34	0.618
103	4	4	0.367	6	0.94	6.527	6	0.047	12.533	1.223	1.266	0.714	-2.418	-0.076	1.138

7															
1038	4	4	0.318	6	0.904	7.027	6	0.079	11.9	1.314	1.388	0.401	-2.299	0.13	1.412
1039	4	4	0.253	6	0.828	7.803	6	0.084	12.377	2.26	0.808	0.824	-2.347	0.47	0.078
1040	4	4	0.318	6	0.853	7.034	7	0.056	12.471	2.175	0.804	1.295	-2.074	0.037	-0.009
1041	4	4	0.417	6	0.894	6.059	7	0.05	11.826	1.873	0.812	1.569	-1.862	-0.251	-0.113
1042	4	4	0.255	6	0.829	7.77	5	0.093	12.141	2.035	0.653	1.856	-1.768	-0.774	-0.28
1043	4	4	0.111	6	0.564	10.329	5	0.354	11.26	2.41	0.464	1.934	-1.729	-1.165	-0.44
1044	4	4	0.233	6	0.758	8.069	5	0.146	11.359	2.135	0.594	1.867	-1.609	-0.898	-0.359
1045	4	4	0.383	6	0.848	6.368	7	0.074	11.255	1.874	0.709	1.826	-1.485	-0.677	-0.291
1046	4	4	0.548	6	0.902	4.969	7	0.064	10.251	1.555	0.849	1.836	-1.358	-0.463	-0.211
1047	4	4	0.825	6	0.894	2.869	7	0.071	7.935	1.307	1.134	1.112	-1.307	0.592	0.056
1048	4	4	0.8	6	0.921	3.07	7	0.051	8.848	0.925	1.326	0.94	-1.31	1.11	0.191
1049	4	4	0.797	6	0.958	3.096	7	0.029	10.102	0.43	1.379	1.165	-1.31	1.152	0.097
105	4	4	0.739	6	0.976	3.537	7	0.017	11.599	0.031	1.406	1.372	-1.328	1.146	0.005

0															
1051	4	4	0.532	6	0.988	5.092	7	0.009	14.593	-0.525	1.516	1.628	-1.332	1.245	-0.021
1052	4	4	0.573	6	0.987	4.778	7	0.01	13.988	-0.427	1.426	1.655	-1.333	1.199	-0.181
1053	4	4	0.602	6	0.984	4.554	7	0.012	13.361	-0.484	1.351	1.64	-1.192	1.163	-0.424
1054	4	4	0.563	6	0.984	4.854	7	0.012	13.606	0.013	1.222	1.753	-1.561	1.187	-0.413
1055	4	4	0.455	6	0.985	5.726	7	0.012	14.57	0.573	0.913	2.094	-1.958	0.574	-0.491
1056	4	4	0.309	6	0.99	7.129	7	0.008	16.81	0.625	0.995	2.272	-2.205	0.627	-0.277
1057	4	4	0.164	6	0.992	9.175	7	0.006	19.47	0.795	1.203	2.201	-2.526	1.134	0.134
1058	4	4	0.116	6	0.991	10.209	7	0.006	20.374	0.867	1.389	2.024	-2.548	1.555	0.405
1059	4	4	0.076	6	0.99	11.429	7	0.007	21.471	0.967	1.497	1.843	-2.615	1.87	0.569
1060	4	4	0.051	6	0.994	12.519	7	0.004	23.64	0.658	1.556	1.908	-2.744	2.013	0.603
1061	4	4	0.039	6	0.996	13.279	7	0.002	25.387	0.349	1.573	1.95	-2.858	2.085	0.553
1062	4	4	0.014	6	0.998	16.015	7	0.001	29.799	-0.056	1.65	2.119	-3.092	2.24	0.665
106	4	4	0	6	0.999	25.211	7	0.001	39.991	-0.181	2.11	2.066	-3.294	3.301	1.325

3															
1064	4	4	0	6	0.999	25.761	7	0.001	39.903	-0.268	2.199	1.887	-3.06	3.602	1.315
1065	4	4	0	6	0.999	25.691	7	0.001	40.198	-0.811	2.274	1.981	-2.752	3.64	1.208
1066	4	4	0.002	6	0.998	21.176	7	0.002	33.988	-0.091	2.025	1.797	-2.803	3.366	0.97
1067	4	4	0.014	6	0.99	16.022	7	0.006	26.15	0.868	1.636	1.512	-2.75	2.909	0.521
1068	4	4	0.004	6	0.994	18.932	7	0.004	30.004	0.984	1.639	1.907	-3.063	2.938	0.561
1069	4	4	0.001	6	0.997	23.325	7	0.002	35.792	1.036	1.666	2.397	-3.453	2.986	0.643
1070	4	4	0	6	0.997	24.41	7	0.002	36.876	1.271	1.551	2.64	-3.596	2.843	0.487
1071	4	4	0	6	0.997	27.099	7	0.002	39.355	1.547	1.505	2.97	-3.67	2.88	0.338
1072	4	4	0	6	0.996	29.339	7	0.003	41.195	1.955	1.365	3.162	-3.824	2.755	0.188
1073	4	4	0	6	0.989	31.632	5	0.006	41.686	2.533	1.104	3.15	-4.068	2.514	-0.047
1074	4	4	0	6	0.995	33.501	5	0.003	45.461	2.219	1.134	3.425	-4.241	2.578	-0.046
1075	4	4	0	6	0.998	35.585	7	0.001	49.41	1.852	1.216	3.706	-4.359	2.697	0.032
107	4	4	0	6	0.999	38.857	7	0.001	53.248	1.866	1.336	3.799	-4.514	2.911	0.228

6															
1077	4	4	0	6	0.999	42.215	7	0.001	56.9	1.981	1.431	3.852	-4.665	3.102	0.397
1078	4	4	0	6	0.999	46.926	7	0	62.752	1.768	1.641	4.002	-4.821	3.461	0.694
1079	4	4	0	6	1	51.492	7	0	69.033	1.445	1.776	4.469	-4.993	3.408	0.922
1080	4	4	0	6	1	54.201	7	0	72.742	1.241	1.822	4.756	-5.091	3.35	0.977
1081	4	4	0	6	1	57.224	7	0	76.715	1.033	1.877	4.975	-5.196	3.383	1.027
1082	4	4	0	6	1	61.335	7	0	81.719	0.885	1.956	5.244	-5.302	3.437	1.117
1083	4	4	0	6	1	61.15	7	0	81.844	0.787	1.904	5.306	-5.336	3.342	1.009
1084	4	4	0	6	1	73.796	7	0	98.129	0.104	2.079	5.63	-5.973	3.59	1.379
1085	4	4	0	6	1	101.988	7	0	131.833	-0.699	2.406	6.234	-7.065	4.12	2.073
1086	4	4	0	6	1	86.789	7	0	115.956	-1.189	2.096	5.792	-6.684	3.697	1.422
1087	4	4	0	6	1	71.214	7	0	98.141	-1.224	2.187	5.329	-5.897	3.311	1.605
1088	4	4	0	6	1	60.05	2	0	85.308	-1.549	2.239	4.918	-5.184	3.121	1.554
108	4	4	0	6	1	49.785	2	0	67.162	-2.178	2.247	4.426	-4.316	3.037	1.24

9																
1090	4	4	0	6	1	45.459	2	0	65.098	-1.914	2.177	4.137	-4.283	2.985	1.211
1091	4	4	0	6	1	41.449	3	0	62.519	-1.606	2.138	3.835	-4.23	2.959	1.243
1092	4	4	0	6	1	37.879	7	0	58.534	-1.281	2.15	3.595	-4.087	2.936	1.346
1093	4	4	0	6	1	35.112	7	0	54.505	-0.927	2.197	3.327	-3.948	2.964	1.52
1094	4	4	0	6	1	31.779	7	0	49.721	-0.598	2.215	3.053	-3.736	2.947	1.625
1095	4	4	0	6	1	26.409	7	0	43.655	-0.568	1.99	2.913	-3.602	2.549	1.303
1096	4	4	0	6	1	24.7	7	0	41.47	-0.215	1.837	2.912	-3.72	2.273	1.185
1097	4	4	0.001	6	1	23.926	7	0	39.937	0.291	1.715	2.896	-3.849	2.069	1.11
1098	4	4	0.001	6	0.999	23.084	7	0	38.629	0.45	1.633	2.921	-3.816	1.949	1.043
1099	4	4	0.001	6	0.999	23.778	7	0	39.239	0.629	1.568	3.027	-3.899	1.818	1.089
1100	4	4	0.004	6	0.999	18.826	7	0.001	33.01	0.698	1.453	2.92	-3.517	1.329	0.914
1101	4	4	0.025	6	0.997	14.467	7	0.002	27.06	0.863	1.306	2.818	-3.097	0.758	0.691
110	4	4	0.055	6	0.997	12.352	7	0.002	24.384	0.727	1.27	2.849	-2.761	0.383	0.624

2															
1103	4	4	0.152	6	0.996	9.407	7	0.003	21.038	0.491	1.278	2.444	-2.509	0.128	0.765
1104	4	4	0.298	6	0.993	7.253	7	0.004	18.076	0.395	1.337	2.097	-2.203	0.041	0.899
1105	4	4	0.44	6	0.989	5.851	7	0.007	15.741	0.345	1.464	1.778	-1.884	0.182	1.081
1106	4	4	0.628	6	0.973	4.361	7	0.016	12.592	0.456	1.566	1.339	-1.465	0.286	1.194
1107	4	4	0.498	6	0.855	5.368	6	0.124	9.231	0.66	0.421	0.316	-2.169	-1.446	0.211
1108	4	4	0.567	6	0.77	4.825	6	0.209	7.431	0.696	0.539	-0.109	-2.178	-1.097	0.417
1109	4	4	0.659	6	0.745	4.131	6	0.234	6.443	0.621	0.7	-0.302	-2.086	-0.696	0.621
1110	4	4	0.667	6	0.719	4.068	6	0.254	6.146	0.439	0.919	-0.555	-1.974	-0.151	0.899
1111	4	4	0.469	6	0.555	5.605	6	0.414	6.192	0.696	1.056	-0.958	-1.945	0.309	1.114
1112	4	4	0.742	6	0.776	3.516	6	0.199	6.234	0.767	0.757	-0.102	-1.927	-0.679	0.599
1113	4	4	0.655	6	0.859	4.163	6	0.116	8.175	0.661	0.489	0.381	-1.912	-1.316	0.169
1114	4	4	0.505	6	0.878	5.307	6	0.097	9.717	0.509	0.265	0.51	-1.978	-1.622	-0.171
111	4	7	0.95	6	0.735	1.631	4	0.217	4.07	0.596	-0.28	0.872	1.037	0.781	0.443

5																
1116	4	7	0.969	6	0.742	1.34	4	0.2	3.96	0.675	-0.342	0.77	0.989	0.785	0.389
1117	4	7	0.981	6	0.747	1.106	4	0.185	3.898	0.754	-0.402	0.674	0.939	0.787	0.338
1118	4	7	0.989	6	0.752	0.908	4	0.168	3.907	0.838	-0.467	0.589	0.901	0.769	0.278
1119	4	7	0.994	6	0.759	0.737	4	0.149	3.992	0.951	-0.543	0.535	0.869	0.71	0.211
1120	4	7	0.995	6	0.759	0.69	4	0.143	4.033	0.98	-0.59	0.529	0.863	0.654	0.155
1121	4	7	0.996	6	0.761	0.642	4	0.132	4.148	1.043	-0.647	0.514	0.852	0.59	0.095
1122	4	7	0.995	6	0.758	0.668	4	0.136	4.102	0.991	-0.661	0.508	0.856	0.587	0.06
1123	4	7	0.995	6	0.754	0.674	4	0.136	4.096	0.962	-0.68	0.464	0.849	0.607	0.026
1124	4	7	0.993	6	0.745	0.759	4	0.149	3.982	0.846	-0.666	0.426	0.851	0.674	0.011
1125	4	7	0.991	6	0.736	0.85	4	0.157	3.939	0.745	-0.645	0.369	0.861	0.747	0.017
1126	4	7	0.983	6	0.718	1.057	4	0.175	3.876	0.606	-0.618	0.324	0.854	0.844	0.015
1127	4	7	0.97	6	0.699	1.33	4	0.196	3.872	0.46	-0.59	0.29	0.853	0.931	0.011
112	4	7	0.955	6	0.682	1.568	4	0.214	3.888	0.352	-0.559	0.269	0.853	0.996	0.03

8															
112<br>9	4	7	0.931	6	0.662	1.87	4	0.237	3.927	0.229	-0.537	0.268	0.852	1.049	0.024
113<br>0	4	7	0.913	6	0.647	2.074	4	0.254	3.943	0.151	-0.515	0.281	0.851	1.078	0.041
113<br>1	4	7	0.888	6	0.63	2.321	4	0.277	3.966	0.06	-0.502	0.314	0.85	1.095	0.034
113<br>2	4	7	0.872	6	0.618	2.472	4	0.295	3.954	0.007	-0.486	0.354	0.849	1.097	0.05
113<br>3	4	7	0.848	6	0.603	2.681	4	0.316	3.969	-0.062	-0.463	0.402	0.854	1.101	0.07
113<br>4	4	7	0.848	6	0.601	2.676	4	0.321	3.926	-0.06	-0.453	0.435	0.854	1.085	0.104
113<br>5	4	7	0.836	6	0.588	2.777	4	0.337	3.893	-0.085	-0.459	0.467	0.831	1.084	0.098
113<br>6	4	7	0.841	6	0.587	2.741	4	0.337	3.853	-0.073	-0.473	0.47	0.817	1.073	0.1
113<br>7	4	7	0.841	6	0.582	2.738	4	0.34	3.811	-0.069	-0.486	0.461	0.793	1.072	0.104
113<br>8	4	7	0.845	6	0.58	2.704	4	0.337	3.789	-0.062	-0.51	0.43	0.775	1.064	0.091
113<br>9	4	7	0.842	6	0.57	2.729	4	0.34	3.765	-0.071	-0.549	0.387	0.736	1.061	0.053
114<br>0	4	7	0.835	6	0.559	2.791	4	0.342	3.773	-0.095	-0.593	0.332	0.701	1.051	0.003
114	4	7	0.836	6	0.551	2.778	4	0.338	3.759	-0.094	-0.643	0.262	0.663	1.022	-0.037

1															
1142	4	7	0.798	6	0.52	3.087	4	0.359	3.827	-0.197	-0.667	0.205	0.629	1.013	-0.071
1143	4	7	0.762	6	0.491	3.363	4	0.376	3.898	-0.288	-0.694	0.136	0.598	0.987	-0.093
1144	4	7	0.686	6	0.443	3.93	4	0.414	4.064	-0.446	-0.719	0.079	0.549	0.97	-0.134
1145	4	4	0.64	6	0.45	4.269	7	0.396	4.524	-0.603	-0.744	0.017	0.504	0.934	-0.164
1146	4	4	0.605	6	0.493	4.535	7	0.343	5.26	-0.779	-0.769	-0.03	0.451	0.89	-0.19
1147	4	4	0.562	6	0.534	4.857	7	0.292	6.066	-0.958	-0.799	-0.059	0.402	0.818	-0.225
1148	4	4	0.517	6	0.574	5.214	7	0.243	6.933	-1.137	-0.832	-0.06	0.351	0.727	-0.256
1149	4	4	0.46	6	0.61	5.68	7	0.196	7.947	-1.327	-0.872	-0.018	0.306	0.607	-0.312
1150	4	4	0.423	6	0.636	6	7	0.165	8.702	-1.459	-0.915	0.074	0.278	0.436	-0.344
1151	4	4	0.365	6	0.648	6.547	7	0.131	9.746	-1.608	-0.97	0.21	0.248	0.253	-0.414
1152	4	4	0.325	6	0.654	6.958	2	0.123	10.308	-1.679	-1.021	0.374	0.242	0.034	-0.465
1153	4	4	0.277	6	0.646	7.504	2	0.179	10.069	-1.749	-1.054	0.575	0.257	-0.177	-0.497
115	4	4	0.246	6	0.668	7.897	2	0.177	10.553	-1.729	-1.094	0.757	0.28	-0.392	-0.537

4																
1155	4	4	0.214	6	0.686	8.343	2	0.173	11.094	-1.714	-1.12	0.935	0.306	-0.548	-0.571
1156	4	4	0.204	6	0.731	8.498	2	0.119	12.136	-1.619	-1.148	1.064	0.33	-0.677	-0.608
1157	4	4	0.214	6	0.762	8.343	7	0.132	11.843	-1.505	-1.15	1.146	0.361	-0.736	-0.599
1158	4	4	0.25	6	0.768	7.842	7	0.157	11.014	-1.376	-1.131	1.155	0.39	-0.71	-0.579
1159	4	4	0.313	6	0.751	7.081	7	0.19	9.834	-1.219	-1.1	1.114	0.417	-0.643	-0.529
1160	4	4	0.383	6	0.731	6.375	7	0.212	8.855	-1.138	-1.05	1.04	0.437	-0.497	-0.481
1161	4	4	0.466	6	0.702	5.632	7	0.237	7.808	-1.043	-1.003	0.926	0.444	-0.325	-0.433
1162	4	4	0.531	6	0.684	5.103	7	0.251	7.11	-1.014	-0.941	0.815	0.454	-0.128	-0.373
1163	4	4	0.577	6	0.667	4.747	7	0.261	6.622	-0.991	-0.896	0.699	0.448	0.063	-0.344
1164	4	4	0.599	6	0.658	4.576	7	0.264	6.401	-1.002	-0.849	0.6	0.442	0.248	-0.312
1165	4	4	0.596	6	0.658	4.603	7	0.258	6.474	-1.046	-0.808	0.51	0.428	0.429	-0.305
1166	4	4	0.605	6	0.639	4.535	7	0.266	6.286	-1.016	-0.791	0.404	0.408	0.574	-0.291
116	4	4	0.6	6	0.623	4.572	7	0.27	6.245	-1.003	-0.77	0.294	0.385	0.727	-0.275

7															
1168	4	4	0.593	6	0.593	4.621	7	0.281	6.117	-0.948	-0.771	0.157	0.353	0.865	-0.275
1169	4	4	0.574	6	0.56	4.77	7	0.287	6.108	-0.896	-0.777	0.006	0.31	1.006	-0.277
1170	4	4	0.54	6	0.525	5.027	7	0.29	6.218	-0.863	-0.776	-0.146	0.281	1.133	-0.267
1171	4	4	0.507	6	0.491	5.289	7	0.288	6.36	-0.828	-0.793	-0.285	0.243	1.218	-0.273
1172	4	4	0.473	6	0.472	5.572	7	0.278	6.627	-0.847	-0.796	-0.388	0.228	1.267	-0.265
1173	4	4	0.457	6	0.453	5.708	7	0.27	6.744	-0.842	-0.84	-0.457	0.198	1.239	-0.307
1174	4	4	0.435	6	0.482	5.894	7	0.243	7.265	-0.993	-0.84	-0.43	0.205	1.174	-0.32
1175	4	4	0.424	6	0.514	5.997	7	0.22	7.698	-1.134	-0.855	-0.365	0.219	1.053	-0.349
1176	4	4	0.395	6	0.559	6.258	7	0.186	8.46	-1.331	-0.87	-0.247	0.229	0.909	-0.395
1177	4	4	0.35	6	0.581	6.698	7	0.15	9.407	-1.536	-0.882	-0.128	0.244	0.766	-0.442
1178	4	4	0.308	6	0.574	7.136	3	0.119	10.278	-1.701	-0.897	-0.014	0.247	0.635	-0.478
1179	4	4	0.267	6	0.522	7.621	2	0.207	9.467	-1.861	-0.884	0.087	0.27	0.539	-0.477
118	4	4	0.245	6	0.474	7.904	2	0.297	8.838	-1.954	-0.874	0.164	0.282	0.473	-0.462

0																
1181	4	2	0.231	6	0.437	8.095	4	0.382	8.362	-2.083	-0.829	0.236	0.319	0.471	-0.419
1182	4	4	0.25	6	0.477	7.84	2	0.323	8.617	-1.986	-0.817	0.254	0.318	0.474	-0.374
1183	4	4	0.288	6	0.556	7.375	2	0.23	9.144	-1.898	-0.782	0.273	0.334	0.518	-0.307
1184	4	4	0.372	6	0.66	6.472	7	0.128	9.757	-1.688	-0.757	0.268	0.342	0.573	-0.236
1185	4	4	0.465	6	0.691	5.639	7	0.171	8.432	-1.467	-0.714	0.271	0.363	0.656	-0.146
1186	4	4	0.56	6	0.668	4.878	7	0.22	7.097	-1.214	-0.682	0.271	0.384	0.742	-0.08
1187	4	4	0.649	6	0.617	4.207	7	0.28	5.789	-0.938	-0.635	0.28	0.42	0.83	0.017
1188	4	4	0.705	6	0.555	3.793	7	0.342	4.763	-0.686	-0.595	0.295	0.462	0.916	0.086
1189	4	4	0.747	6	0.488	3.478	7	0.408	3.839	-0.417	-0.562	0.314	0.491	0.987	0.152
1190	4	7	0.773	6	0.467	3.281	4	0.432	3.437	-0.233	-0.524	0.335	0.552	1.049	0.201
1191	4	7	0.829	6	0.519	2.837	4	0.382	3.448	-0.06	-0.507	0.355	0.591	1.099	0.218
1192	4	7	0.867	6	0.564	2.514	4	0.34	3.526	0.073	-0.484	0.367	0.644	1.13	0.242
119	4	7	0.898	6	0.601	2.228	4	0.304	3.593	0.197	-0.476	0.371	0.681	1.141	0.251

3															
1194	4	7	0.916	6	0.628	2.035	4	0.278	3.665	0.28	-0.469	0.371	0.716	1.143	0.25
1195	4	7	0.931	6	0.645	1.869	4	0.26	3.688	0.344	-0.486	0.365	0.727	1.128	0.212
1196	4	7	0.942	6	0.657	1.74	4	0.246	3.707	0.385	-0.492	0.356	0.747	1.107	0.191
1197	4	7	0.955	6	0.67	1.559	4	0.229	3.701	0.445	-0.514	0.335	0.755	1.066	0.156
1198	4	7	0.951	6	0.662	1.629	4	0.238	3.675	0.387	-0.519	0.338	0.763	1.052	0.114
1199	4	7	0.948	6	0.658	1.66	4	0.242	3.657	0.346	-0.519	0.335	0.777	1.03	0.089
1200	4	7	0.938	6	0.646	1.792	4	0.256	3.641	0.265	-0.509	0.342	0.791	1.019	0.073
1201	4	7	0.924	6	0.633	1.957	4	0.272	3.648	0.176	-0.497	0.348	0.808	1.011	0.057
1202	4	7	0.912	6	0.622	2.077	4	0.284	3.645	0.113	-0.491	0.351	0.816	0.998	0.041
1203	4	7	0.896	6	0.61	2.241	4	0.299	3.666	0.038	-0.479	0.362	0.829	0.989	0.029
1204	4	7	0.894	6	0.606	2.263	4	0.304	3.644	0.017	-0.481	0.366	0.832	0.965	0.011
1205	4	7	0.886	6	0.6	2.339	4	0.312	3.646	-0.021	-0.475	0.384	0.844	0.942	-0.003
120	4	7	0.901	6	0.607	2.194	4	0.305	3.569	0.02	-0.491	0.4	0.842	0.892	-0.024

6															
1207	4	7	0.92	6	0.619	2.001	4	0.293	3.495	0.079	-0.499	0.42	0.852	0.831	-0.024
1208	4	7	0.927	6	0.625	1.915	4	0.29	3.45	0.108	-0.507	0.459	0.858	0.775	-0.039
1209	4	7	0.939	6	0.637	1.773	4	0.28	3.413	0.161	-0.509	0.5	0.876	0.71	-0.033
1210	4	7	0.941	6	0.643	1.753	4	0.28	3.414	0.172	-0.495	0.556	0.904	0.659	-0.017
1211	4	7	0.943	6	0.65	1.727	4	0.277	3.433	0.193	-0.481	0.611	0.932	0.61	0
1212	4	7	0.94	6	0.655	1.76	4	0.278	3.473	0.194	-0.456	0.668	0.965	0.575	0.024
1213	4	7	0.935	6	0.658	1.824	4	0.28	3.532	0.186	-0.425	0.721	1	0.554	0.052
1214	4	7	0.938	6	0.669	1.79	4	0.273	3.581	0.218	-0.403	0.754	1.027	0.536	0.078
1215	4	7	0.94	6	0.677	1.769	4	0.267	3.632	0.243	-0.376	0.779	1.054	0.532	0.106
1216	4	7	0.945	6	0.688	1.698	4	0.257	3.666	0.284	-0.369	0.786	1.065	0.538	0.112
1217	4	7	0.949	6	0.692	1.642	4	0.253	3.657	0.311	-0.379	0.781	1.052	0.561	0.074
1218	4	7	0.952	6	0.695	1.612	4	0.249	3.666	0.325	-0.391	0.769	1.04	0.596	0.039
121	4	7	0.955	6	0.698	1.563	4	0.244	3.661	0.344	-0.404	0.742	1.023	0.638	0.003

9															
1220	4	7	0.952	6	0.694	1.605	4	0.247	3.673	0.328	-0.426	0.719	0.999	0.691	-0.05
1221	4	7	0.949	6	0.69	1.653	4	0.249	3.691	0.314	-0.451	0.689	0.971	0.741	-0.107
1222	4	7	0.941	6	0.682	1.759	4	0.256	3.721	0.28	-0.474	0.662	0.943	0.794	-0.164
1223	4	7	0.926	6	0.67	1.931	4	0.266	3.778	0.232	-0.508	0.636	0.905	0.846	-0.244
1224	4	7	0.917	6	0.662	2.027	4	0.271	3.813	0.206	-0.523	0.602	0.88	0.889	-0.285
1225	4	7	0.91	6	0.656	2.104	4	0.274	3.848	0.182	-0.529	0.564	0.864	0.93	-0.304
1226	4	7	0.904	6	0.65	2.161	4	0.277	3.868	0.168	-0.536	0.517	0.839	0.971	-0.326
1227	4	7	0.896	6	0.642	2.242	4	0.28	3.902	0.15	-0.545	0.464	0.813	1.015	-0.35
1228	4	7	0.898	6	0.639	2.225	4	0.277	3.897	0.152	-0.533	0.402	0.8	1.054	-0.331
1229	4	7	0.894	6	0.633	2.26	4	0.276	3.921	0.149	-0.534	0.339	0.776	1.092	-0.337
1230	4	7	0.89	6	0.625	2.3	4	0.277	3.925	0.137	-0.521	0.283	0.76	1.13	-0.317
1231	4	7	0.884	6	0.616	2.357	4	0.28	3.934	0.118	-0.507	0.234	0.747	1.161	-0.298
123	4	7	0.884	6	0.61	2.354	4	0.28	3.91	0.12	-0.501	0.195	0.73	1.174	-0.283

2															
123<br>3	4	7	0.888	6	0.607	2.316	4	0.281	3.858	0.119	-0.483	0.172	0.73	1.173	-0.245
123<br>4	4	7	0.901	6	0.606	2.197	4	0.276	3.773	0.155	-0.488	0.155	0.714	1.149	-0.238
123<br>5	4	7	0.912	6	0.607	2.079	4	0.273	3.675	0.184	-0.49	0.158	0.706	1.113	-0.23
123<br>6	4	7	0.928	6	0.61	1.913	4	0.267	3.569	0.243	-0.512	0.165	0.684	1.063	-0.25
123<br>7	4	7	0.942	6	0.616	1.744	4	0.26	3.472	0.307	-0.531	0.182	0.665	1.009	-0.269
123<br>8	4	7	0.95	6	0.62	1.631	4	0.254	3.415	0.363	-0.558	0.206	0.646	0.958	-0.308
123<br>9	4	7	0.959	6	0.627	1.51	4	0.246	3.381	0.435	-0.584	0.23	0.625	0.908	-0.346
124<br>0	4	7	0.965	6	0.635	1.416	4	0.241	3.351	0.476	-0.588	0.264	0.63	0.869	-0.352
124<br>1	4	7	0.969	6	0.643	1.348	4	0.236	3.356	0.53	-0.602	0.298	0.623	0.833	-0.381
124<br>2	4	7	0.968	6	0.647	1.364	4	0.239	3.356	0.527	-0.6	0.346	0.635	0.814	-0.399
124<br>3	4	7	0.971	6	0.655	1.313	4	0.236	3.353	0.551	-0.591	0.395	0.655	0.787	-0.396
124<br>4	4	7	0.966	6	0.656	1.392	4	0.245	3.358	0.513	-0.577	0.459	0.68	0.775	-0.408
124	4	7	0.963	6	0.658	1.444	4	0.253	3.355	0.479	-0.552	0.523	0.715	0.761	-0.396

5															
1246	4	7	0.957	6	0.658	1.533	4	0.26	3.391	0.449	-0.541	0.58	0.738	0.746	-0.413
1247	4	7	0.948	6	0.656	1.659	4	0.27	3.434	0.403	-0.527	0.633	0.762	0.742	-0.426
1248	4	7	0.945	6	0.658	1.696	4	0.27	3.48	0.404	-0.526	0.657	0.771	0.735	-0.449
1249	4	7	0.941	6	0.658	1.75	4	0.271	3.521	0.39	-0.522	0.661	0.777	0.752	-0.468
1250	4	7	0.943	6	0.662	1.725	4	0.265	3.554	0.414	-0.527	0.631	0.765	0.78	-0.491
1251	4	7	0.944	6	0.663	1.715	4	0.259	3.591	0.428	-0.528	0.567	0.746	0.842	-0.51
1252	4	7	0.953	6	0.669	1.595	4	0.244	3.614	0.475	-0.512	0.469	0.733	0.918	-0.483
1253	4	7	0.96	6	0.676	1.499	4	0.224	3.708	0.54	-0.502	0.336	0.711	1.013	-0.459
1254	4	7	0.954	6	0.67	1.575	4	0.217	3.829	0.536	-0.456	0.2	0.699	1.152	-0.392
1255	4	7	0.947	6	0.666	1.673	4	0.204	4.042	0.556	-0.407	0.052	0.692	1.284	-0.309
1256	4	7	0.923	6	0.653	1.96	4	0.202	4.301	0.513	-0.328	-0.077	0.699	1.43	-0.189
1257	4	7	0.888	6	0.637	2.324	4	0.202	4.624	0.471	-0.268	-0.198	0.696	1.556	-0.102
125	4	7	0.833	6	0.616	2.807	4	0.211	4.954	0.385	-0.187	-0.29	0.704	1.675	0.016

8															
125<br>9	4	7	0.773	6	0.593	3.284	4	0.224	5.233	0.291	-0.126	-0.359	0.704	1.76	0.1
126<br>0	4	7	0.702	6	0.563	3.816	4	0.248	5.455	0.164	-0.072	-0.399	0.692	1.828	0.161
126<br>1	4	7	0.643	6	0.534	4.252	4	0.275	5.58	0.041	-0.031	-0.418	0.684	1.859	0.21
126<br>2	4	7	0.582	6	0.498	4.706	4	0.318	5.605	-0.113	0.018	-0.399	0.673	1.866	0.259
126<br>3	4	7	0.526	6	0.46	5.139	4	0.364	5.603	-0.267	0.06	-0.366	0.663	1.853	0.304
126<br>4	4	4	0.47	6	0.419	5.595	7	0.417	5.605	-0.43	0.107	-0.318	0.655	1.824	0.344
126<br>5	4	4	0.465	6	0.478	5.64	7	0.371	6.147	-0.602	0.153	-0.26	0.647	1.791	0.385
126<br>6	4	4	0.457	6	0.525	5.702	7	0.333	6.612	-0.741	0.204	-0.217	0.649	1.745	0.435
126<br>7	4	4	0.438	6	0.569	5.871	7	0.297	7.172	-0.879	0.269	-0.174	0.661	1.703	0.513
126<br>8	4	4	0.418	6	0.598	6.047	7	0.271	7.628	-0.982	0.338	-0.146	0.686	1.652	0.603
126<br>9	4	4	0.382	6	0.633	6.38	7	0.241	8.31	-1.11	0.418	-0.098	0.715	1.609	0.706
127<br>0	4	4	0.397	6	0.644	6.242	7	0.231	8.292	-1.144	0.448	-0.069	0.733	1.517	0.723
127	4	4	0.405	6	0.664	6.167	7	0.219	8.383	-1.197	0.486	-0.008	0.762	1.417	0.749

1															
1272	4	4	0.462	6	0.671	5.663	7	0.224	7.857	-1.164	0.49	0.065	0.789	1.258	0.723
1273	4	4	0.519	6	0.69	5.199	7	0.221	7.47	-1.163	0.482	0.192	0.815	1.081	0.66
1274	4	4	0.617	6	0.691	4.445	7	0.235	6.605	-1.073	0.442	0.327	0.836	0.851	0.548
1275	4	4	0.686	6	0.697	3.933	7	0.244	6.034	-1.001	0.401	0.509	0.865	0.607	0.422
1276	4	4	0.757	6	0.686	3.4	7	0.264	5.309	-0.86	0.323	0.684	0.874	0.337	0.232
1277	4	4	0.775	6	0.675	3.262	7	0.283	5.004	-0.736	0.258	0.869	0.893	0.087	0.056
1278	4	4	0.786	6	0.64	3.179	7	0.321	4.556	-0.518	0.166	1.012	0.89	-0.159	-0.155
1279	4	4	0.76	6	0.6	3.378	7	0.362	4.387	-0.302	0.07	1.134	0.875	-0.372	-0.381
1280	4	4	0.712	6	0.553	3.741	7	0.408	4.349	-0.083	-0.005	1.225	0.867	-0.537	-0.567
1281	4	4	0.65	6	0.501	4.198	7	0.457	4.379	0.143	-0.083	1.276	0.843	-0.659	-0.769
1282	4	7	0.614	6	0.5	4.463	4	0.453	4.662	0.337	-0.136	1.313	0.828	-0.729	-0.924
1283	4	7	0.624	6	0.548	4.388	4	0.395	5.04	0.549	-0.173	1.303	0.815	-0.758	-1.05
128	4	7	0.638	6	0.585	4.287	4	0.347	5.333	0.731	-0.2	1.274	0.798	-0.742	-1.158

4																
128<br>5	4	7	0.683	6	0.626	3.956	4	0.291	5.491	0.938	-0.204	1.192	0.789	-0.693	-1.222
128<br>6	4	7	0.754	6	0.657	3.427	4	0.248	5.373	1.1	-0.191	1.076	0.776	-0.581	-1.233
128<br>7	4	7	0.834	6	0.688	2.799	4	0.203	5.241	1.29	-0.162	0.914	0.767	-0.452	-1.205
128<br>8	4	7	0.91	6	0.709	2.102	4	0.174	4.913	1.412	-0.111	0.722	0.757	-0.267	-1.133
128<br>9	4	7	0.96	6	0.723	1.487	4	0.141	4.763	1.571	-0.054	0.471	0.743	-0.073	-1.03
129<br>0	4	7	0.986	6	0.722	1.002	4	0.128	4.457	1.594	0.028	0.236	0.736	0.163	-0.902
129<br>1	4	7	0.992	6	0.707	0.801	6	0.163	3.733	1.643	0.1	-0.028	0.718	0.377	-0.771
129<br>2	4	7	0.992	6	0.673	0.798	6	0.205	3.172	1.55	0.187	-0.264	0.7	0.622	-0.643
129<br>3	4	7	0.984	6	0.632	1.029	6	0.256	2.833	1.496	0.259	-0.493	0.683	0.805	-0.521
129<br>4	4	7	0.97	6	0.585	1.321	6	0.289	2.728	1.265	0.346	-0.632	0.678	0.978	-0.398
129<br>5	4	7	0.933	6	0.532	1.847	6	0.325	2.832	1.023	0.427	-0.772	0.669	1.137	-0.285
129<br>6	4	7	0.868	6	0.477	2.503	6	0.356	3.086	0.777	0.495	-0.887	0.651	1.263	-0.175
129	4	7	0.78	6	0.425	3.229	6	0.373	3.49	0.503	0.55	-0.96	0.635	1.348	-0.098

7																
1298	4	6	0.701	6	0.385	3.818	7	0.377	3.861	0.292	0.584	-1.013	0.603	1.387	-0.03
1299	4	6	0.648	6	0.377	4.216	7	0.335	4.455	0.06	0.609	-1.02	0.578	1.392	0.011
1300	4	6	0.615	6	0.36	4.458	4	0.314	4.735	-0.119	0.612	-0.997	0.536	1.357	0.022
1301	4	4	0.605	6	0.371	4.53	6	0.32	4.829	-0.324	0.611	-0.922	0.508	1.3	0.017
1302	4	4	0.669	6	0.421	4.06	6	0.285	4.841	-0.424	0.589	-0.821	0.461	1.193	-0.026
1303	4	4	0.728	6	0.484	3.619	6	0.24	5.019	-0.539	0.567	-0.686	0.417	1.085	-0.08
1304	4	4	0.795	6	0.535	3.11	6	0.207	5.007	-0.583	0.525	-0.545	0.376	0.951	-0.17
1305	4	4	0.843	6	0.595	2.721	7	0.193	4.977	-0.64	0.491	-0.38	0.335	0.837	-0.264
1306	4	4	0.881	6	0.627	2.386	7	0.191	4.766	-0.625	0.427	-0.248	0.299	0.714	-0.4
1307	4	4	0.903	6	0.655	2.176	7	0.19	4.652	-0.605	0.393	-0.127	0.276	0.635	-0.492
1308	4	4	0.919	6	0.654	2.012	7	0.202	4.36	-0.522	0.341	-0.054	0.263	0.563	-0.599
1309	4	4	0.923	6	0.648	1.959	7	0.213	4.19	-0.437	0.293	-0.012	0.232	0.541	-0.714
131	4	4	0.922	6	0.623	1.977	7	0.233	3.94	-0.342	0.261	-0.019	0.236	0.562	-0.776

0															
131<br>1	4	4	0.912	6	0.59	2.085	7	0.255	3.763	-0.237	0.221	-0.058	0.222	0.612	-0.859
131<br>2	4	4	0.893	6	0.563	2.277	7	0.271	3.743	-0.184	0.21	-0.114	0.222	0.718	-0.894
131<br>3	4	4	0.861	6	0.526	2.564	7	0.287	3.772	-0.115	0.197	-0.207	0.213	0.847	-0.931
131<br>4	4	4	0.818	6	0.502	2.93	7	0.295	3.993	-0.101	0.213	-0.303	0.21	1.017	-0.924
131<br>5	4	4	0.75	6	0.471	3.458	7	0.3	4.362	-0.083	0.226	-0.43	0.199	1.206	-0.918
131<br>6	4	4	0.66	6	0.444	4.12	7	0.298	4.922	-0.091	0.258	-0.569	0.189	1.417	-0.88
131<br>7	4	4	0.549	6	0.402	4.956	7	0.295	5.575	-0.081	0.301	-0.746	0.191	1.635	-0.805
131<br>8	4	4	0.417	6	0.37	6.051	6	0.32	6.342	-0.109	0.369	-0.934	0.174	1.887	-0.691
131<br>9	4	6	0.306	6	0.366	7.166	4	0.329	7.379	-0.128	0.438	-1.14	0.163	2.128	-0.565
132<br>0	4	6	0.222	6	0.398	8.228	4	0.296	8.823	-0.172	0.534	-1.326	0.154	2.361	-0.386
132<br>1	4	6	0.161	6	0.423	9.225	4	0.262	10.183	-0.207	0.623	-1.491	0.159	2.539	-0.204
132<br>2	4	6	0.117	6	0.425	10.198	4	0.246	11.29	-0.255	0.748	-1.591	0.186	2.667	0.051
132	4	6	0.096	6	0.414	10.749	4	0.244	11.811	-0.303	0.852	-1.623	0.227	2.697	0.28

3															
1324	4	6	0.082	6	0.389	11.198	4	0.262	11.983	-0.362	0.978	-1.582	0.281	2.67	0.559
1325	4	6	0.076	6	0.356	11.421	4	0.296	11.792	-0.427	1.09	-1.49	0.343	2.572	0.819
1326	4	4	0.075	6	0.344	11.465	6	0.32	11.611	-0.485	1.209	-1.353	0.422	2.412	1.114
1327	4	4	0.081	6	0.403	11.248	6	0.277	11.997	-0.561	1.316	-1.2	0.479	2.239	1.386
1328	4	4	0.085	6	0.45	11.111	6	0.243	12.341	-0.639	1.397	-1.066	0.562	2.025	1.621
1329	4	4	0.087	6	0.503	11.058	6	0.208	12.83	-0.729	1.466	-0.922	0.614	1.804	1.835
1330	4	4	0.09	6	0.543	10.959	6	0.174	13.236	-0.85	1.503	-0.778	0.71	1.532	1.984
1331	4	4	0.099	6	0.589	10.683	7	0.141	13.547	-0.981	1.513	-0.598	0.777	1.227	2.082
1332	4	7	0.821	6	0.448	2.903	4	0.3	3.706	0.356	-0.114	-0.428	0.303	1.335	-0.645
1333	4	7	0.801	6	0.424	3.059	4	0.332	3.548	0.254	-0.118	-0.392	0.282	1.274	-0.705
1334	4	7	0.785	6	0.404	3.187	4	0.365	3.393	0.173	-0.114	-0.345	0.264	1.213	-0.762
1335	4	4	0.778	6	0.4	3.245	7	0.388	3.306	0.093	-0.099	-0.279	0.26	1.155	-0.81
133	4	4	0.803	6	0.421	3.044	7	0.384	3.227	0.075	-0.079	-0.207	0.267	1.084	-0.835

6															
1337	4	4	0.823	6	0.446	2.884	7	0.381	3.197	0.052	-0.056	-0.11	0.282	1.02	-0.872
1338	4	4	0.852	6	0.451	2.641	7	0.394	2.91	0.097	-0.02	-0.013	0.317	0.939	-0.864
1339	4	4	0.872	6	0.462	2.467	7	0.404	2.733	0.131	0.02	0.101	0.351	0.871	-0.87
1340	4	4	0.888	6	0.446	2.319	7	0.434	2.374	0.231	0.046	0.207	0.404	0.783	-0.856
1341	4	7	0.906	6	0.46	2.146	4	0.433	2.272	0.322	0.069	0.312	0.449	0.706	-0.863
1342	4	7	0.926	6	0.492	1.93	4	0.412	2.285	0.429	0.079	0.411	0.492	0.634	-0.863
1343	4	7	0.935	6	0.517	1.831	4	0.395	2.37	0.525	0.084	0.498	0.518	0.58	-0.884
1344	4	7	0.941	6	0.546	1.747	4	0.371	2.52	0.622	0.076	0.566	0.549	0.531	-0.906
1345	4	7	0.946	6	0.571	1.69	4	0.349	2.676	0.713	0.07	0.608	0.569	0.503	-0.927
1346	4	7	0.941	6	0.59	1.75	4	0.33	2.916	0.783	0.033	0.63	0.57	0.49	-0.996
1347	4	7	0.941	6	0.611	1.75	4	0.305	3.137	0.873	0.003	0.62	0.565	0.489	-1.046
1348	4	7	0.938	6	0.622	1.792	4	0.293	3.297	0.894	-0.019	0.595	0.569	0.522	-1.087
134	4	7	0.929	6	0.628	1.9	4	0.282	3.504	0.914	-0.057	0.553	0.553	0.562	-1.153

9																
1350	4	7	0.927	6	0.632	1.92	4	0.275	3.581	0.901	-0.065	0.503	0.557	0.618	-1.169
1351	4	7	0.924	6	0.633	1.956	4	0.268	3.676	0.89	-0.079	0.439	0.554	0.673	-1.189
1352	4	7	0.916	6	0.628	2.039	4	0.269	3.736	0.846	-0.086	0.377	0.548	0.738	-1.206
1353	4	7	0.919	6	0.626	2.011	4	0.264	3.735	0.817	-0.079	0.304	0.557	0.791	-1.184
1354	4	7	0.917	6	0.618	2.028	4	0.265	3.723	0.769	-0.068	0.232	0.561	0.847	-1.162
1355	4	7	0.92	6	0.613	2.001	4	0.261	3.711	0.735	-0.055	0.149	0.57	0.891	-1.126
1356	4	7	0.915	6	0.596	2.054	4	0.269	3.647	0.652	-0.032	0.079	0.575	0.948	-1.078
1357	4	7	0.912	6	0.582	2.077	4	0.273	3.593	0.581	-0.004	0.001	0.586	0.996	-1.014
1358	4	7	0.903	6	0.562	2.172	4	0.285	3.53	0.481	0.027	-0.064	0.596	1.048	-0.94
1359	4	7	0.889	6	0.542	2.306	4	0.297	3.511	0.382	0.056	-0.128	0.606	1.092	-0.871
1360	4	7	0.87	6	0.519	2.484	4	0.314	3.49	0.27	0.083	-0.174	0.614	1.135	-0.792
1361	4	7	0.847	6	0.498	2.69	4	0.333	3.495	0.156	0.112	-0.208	0.627	1.167	-0.715
136	4	7	0.819	6	0.475	2.92	4	0.354	3.51	0.048	0.122	-0.227	0.627	1.19	-0.655

2															
136<br>3	4	7	0.778	6	0.451	3.238	4	0.382	3.568	-0.087	0.144	-0.226	0.643	1.21	-0.594
136<br>4	4	7	0.783	6	0.443	3.204	4	0.389	3.461	-0.109	0.129	-0.226	0.631	1.191	-0.537
136<br>5	4	7	0.791	6	0.437	3.143	4	0.395	3.347	-0.132	0.129	-0.216	0.636	1.167	-0.457
136<br>6	4	7	0.81	6	0.435	2.992	4	0.396	3.179	-0.12	0.107	-0.201	0.622	1.131	-0.399
136<br>7	4	7	0.83	6	0.434	2.83	4	0.398	3.008	-0.104	0.086	-0.179	0.61	1.09	-0.342
136<br>8	4	7	0.852	6	0.437	2.643	4	0.397	2.837	-0.083	0.076	-0.152	0.612	1.043	-0.26
136<br>9	4	7	0.872	6	0.439	2.47	4	0.398	2.664	-0.061	0.054	-0.114	0.605	0.991	-0.202
137<br>0	4	7	0.883	6	0.44	2.365	4	0.405	2.53	-0.063	0.04	-0.064	0.611	0.937	-0.143
137<br>1	4	7	0.89	6	0.437	2.3	4	0.419	2.384	-0.073	0.019	0.009	0.608	0.878	-0.101
137<br>2	4	7	0.904	6	0.446	2.16	4	0.42	2.279	-0.064	-0.012	0.087	0.632	0.789	-0.078
137<br>3	4	7	0.906	6	0.447	2.14	4	0.432	2.208	-0.081	-0.056	0.188	0.643	0.703	-0.094
137<br>4	4	7	0.921	6	0.463	1.985	4	0.427	2.148	-0.039	-0.115	0.287	0.659	0.592	-0.122
137	4	7	0.928	6	0.479	1.903	4	0.421	2.16	-0.003	-0.167	0.396	0.684	0.485	-0.143

5															
1376	4	7	0.938	6	0.501	1.792	4	0.407	2.21	0.071	-0.237	0.495	0.693	0.378	-0.191
1377	4	7	0.945	6	0.526	1.703	4	0.387	2.318	0.15	-0.29	0.583	0.715	0.289	-0.209
1378	4	7	0.957	6	0.559	1.539	4	0.356	2.442	0.268	-0.337	0.647	0.736	0.211	-0.203
1379	4	7	0.971	6	0.599	1.304	4	0.314	2.599	0.422	-0.38	0.677	0.758	0.15	-0.173
1380	4	7	0.978	6	0.622	1.186	4	0.293	2.693	0.486	-0.369	0.696	0.797	0.155	-0.1
1381	4	7	0.986	6	0.65	1.002	4	0.263	2.81	0.583	-0.356	0.686	0.834	0.168	-0.006
1382	4	7	0.987	6	0.665	0.948	4	0.253	2.885	0.588	-0.295	0.67	0.893	0.232	0.126
1383	4	7	0.989	6	0.681	0.899	4	0.238	3.003	0.611	-0.243	0.63	0.94	0.304	0.255
1384	4	7	0.984	6	0.68	1.033	4	0.242	3.102	0.544	-0.171	0.597	0.987	0.41	0.371
1385	4	7	0.976	6	0.682	1.215	4	0.241	3.292	0.494	-0.098	0.549	1.035	0.51	0.504
1386	4	7	0.954	6	0.67	1.585	4	0.255	3.519	0.378	-0.018	0.51	1.08	0.627	0.617
1387	4	7	0.917	6	0.653	2.033	4	0.273	3.779	0.246	0.046	0.472	1.114	0.736	0.703
138	4	7	0.867	6	0.628	2.515	4	0.299	3.999	0.11	0.1	0.44	1.124	0.837	0.761

8															
1389	4	7	0.791	6	0.602	3.138	4	0.327	4.355	-0.038	0.165	0.415	1.149	0.926	0.838
1390	4	7	0.737	6	0.572	3.551	4	0.357	4.497	-0.146	0.191	0.397	1.131	0.989	0.861
1391	4	7	0.668	6	0.539	4.066	4	0.393	4.699	-0.279	0.219	0.399	1.124	1.036	0.881
1392	4	7	0.641	6	0.514	4.264	4	0.418	4.681	-0.328	0.225	0.409	1.089	1.046	0.894
1393	4	7	0.609	6	0.485	4.5	4	0.45	4.651	-0.404	0.224	0.445	1.06	1.04	0.881
1394	4	4	0.619	6	0.474	4.426	7	0.464	4.469	-0.407	0.207	0.493	1.004	1.008	0.87
1395	4	4	0.642	6	0.509	4.258	7	0.435	4.571	-0.447	0.19	0.566	0.954	0.978	0.842
1396	4	4	0.701	6	0.516	3.819	7	0.429	4.191	-0.367	0.144	0.628	0.881	0.909	0.799
1397	4	4	0.755	6	0.533	3.417	7	0.415	3.92	-0.302	0.095	0.705	0.792	0.851	0.746
1398	4	4	0.805	6	0.525	3.033	7	0.423	3.468	-0.202	0.028	0.756	0.738	0.767	0.66
1399	4	4	0.845	6	0.52	2.706	7	0.427	3.1	-0.09	-0.034	0.816	0.669	0.689	0.587
1400	4	4	0.865	6	0.498	2.534	7	0.449	2.743	-0.005	-0.103	0.854	0.661	0.592	0.477
140	4	4	0.88	6	0.479	2.398	7	0.467	2.447	0.091	-0.182	0.9	0.634	0.498	0.351

1															
1402	4	7	0.887	6	0.485	2.328	4	0.461	2.43	0.127	-0.265	0.944	0.644	0.405	0.187
1403	4	7	0.897	6	0.51	2.232	4	0.435	2.547	0.19	-0.352	0.982	0.653	0.308	0.02
1404	4	7	0.879	6	0.517	2.409	4	0.43	2.778	0.175	-0.412	1.04	0.674	0.249	-0.122
1405	4	7	0.856	6	0.528	2.606	4	0.421	3.057	0.161	-0.47	1.083	0.7	0.204	-0.263
1406	4	7	0.834	6	0.533	2.799	4	0.417	3.29	0.149	-0.518	1.112	0.705	0.199	-0.384
1407	4	7	0.816	6	0.54	2.946	4	0.411	3.494	0.131	-0.56	1.105	0.709	0.233	-0.499
1408	4	7	0.817	6	0.548	2.936	4	0.4	3.566	0.141	-0.588	1.061	0.693	0.305	-0.578
1409	4	7	0.831	6	0.559	2.82	4	0.386	3.563	0.148	-0.602	0.963	0.678	0.424	-0.629
1410	4	7	0.862	6	0.573	2.557	4	0.363	3.468	0.185	-0.597	0.824	0.656	0.567	-0.631
1411	4	7	0.886	6	0.587	2.34	4	0.336	3.457	0.235	-0.605	0.645	0.621	0.719	-0.643
1412	4	7	0.88	6	0.576	2.392	4	0.334	3.478	0.196	-0.577	0.471	0.581	0.905	-0.615
1413	4	7	0.878	6	0.569	2.418	4	0.322	3.555	0.187	-0.547	0.286	0.552	1.057	-0.559
141	4	7	0.835	6	0.537	2.79	4	0.338	3.714	0.076	-0.499	0.138	0.523	1.208	-0.495

4															
1415	4	7	0.781	6	0.502	3.22	4	0.353	3.924	-0.03	-0.471	-0.001	0.486	1.314	-0.451
1416	4	7	0.694	6	0.453	3.869	4	0.389	4.177	-0.204	-0.419	-0.107	0.459	1.413	-0.376
1417	4	4	0.61	6	0.428	4.493	7	0.4	4.627	-0.387	-0.391	-0.198	0.418	1.478	-0.338
1418	4	4	0.56	6	0.475	4.872	7	0.344	5.52	-0.596	-0.345	-0.267	0.387	1.535	-0.271
1419	4	4	0.501	6	0.519	5.343	7	0.289	6.513	-0.818	-0.299	-0.318	0.37	1.575	-0.211
1420	4	4	0.461	6	0.557	5.675	7	0.24	7.36	-0.99	-0.272	-0.336	0.322	1.593	-0.161
1421	4	4	0.42	6	0.586	6.028	7	0.198	8.195	-1.165	-0.227	-0.328	0.303	1.599	-0.086
1422	4	4	0.409	6	0.613	6.129	7	0.168	8.72	-1.271	-0.212	-0.29	0.252	1.583	-0.05
1423	4	4	0.4	6	0.642	6.214	7	0.145	9.186	-1.376	-0.174	-0.216	0.236	1.56	0.02
1424	4	4	0.427	6	0.676	5.969	7	0.135	9.189	-1.392	-0.159	-0.131	0.198	1.514	0.065
1425	4	4	0.453	6	0.714	5.744	7	0.128	9.188	-1.41	-0.132	-0.016	0.181	1.468	0.122
1426	4	4	0.508	6	0.749	5.283	7	0.127	8.832	-1.358	-0.115	0.102	0.156	1.405	0.176
142	4	4	0.556	6	0.785	4.902	7	0.123	8.602	-1.311	-0.071	0.249	0.137	1.354	0.275

7															
1428	4	4	0.636	6	0.788	4.298	7	0.139	7.768	-1.182	-0.064	0.35	0.169	1.246	0.315
1429	4	4	0.705	6	0.788	3.791	7	0.152	7.089	-1.056	-0.049	0.463	0.189	1.15	0.37
1430	4	4	0.767	6	0.766	3.327	7	0.178	6.246	-0.917	-0.067	0.541	0.249	1.028	0.36
1431	4	4	0.814	6	0.739	2.956	7	0.208	5.492	-0.77	-0.068	0.613	0.311	0.92	0.382
1432	4	4	0.846	6	0.697	2.692	7	0.249	4.749	-0.627	-0.092	0.657	0.389	0.813	0.365
1433	4	4	0.867	6	0.648	2.516	7	0.295	4.09	-0.482	-0.109	0.681	0.462	0.734	0.354
1434	4	4	0.878	6	0.592	2.415	7	0.347	3.485	-0.341	-0.138	0.682	0.53	0.677	0.322
1435	4	4	0.883	6	0.53	2.368	7	0.403	2.916	-0.2	-0.176	0.653	0.588	0.651	0.27
1436	4	4	0.881	6	0.477	2.382	7	0.451	2.494	-0.077	-0.192	0.61	0.627	0.681	0.245
1437	4	7	0.914	6	0.503	2.065	4	0.416	2.442	0.061	-0.212	0.543	0.665	0.725	0.219
1438	4	7	0.933	6	0.538	1.852	4	0.376	2.572	0.15	-0.206	0.476	0.693	0.813	0.215
1439	4	7	0.949	6	0.572	1.646	4	0.333	2.729	0.25	-0.218	0.393	0.706	0.904	0.183
144	4	7	0.946	6	0.59	1.683	4	0.313	2.95	0.279	-0.193	0.332	0.728	1.019	0.192

0																
1441	4	7	0.945	6	0.607	1.702	4	0.291	3.17	0.319	-0.18	0.268	0.745	1.113	0.19
1442	4	7	0.927	6	0.606	1.918	4	0.291	3.383	0.283	-0.161	0.232	0.754	1.216	0.175
1443	4	7	0.912	6	0.606	2.086	4	0.291	3.554	0.253	-0.155	0.199	0.759	1.287	0.144
1444	4	7	0.876	6	0.586	2.428	4	0.314	3.68	0.154	-0.152	0.201	0.746	1.354	0.1
1445	4	7	0.836	6	0.564	2.781	4	0.339	3.8	0.047	-0.156	0.205	0.732	1.403	0.047
1446	4	7	0.787	6	0.534	3.172	4	0.372	3.895	-0.069	-0.165	0.22	0.705	1.438	-0.008
1447	4	7	0.736	6	0.505	3.562	4	0.406	3.999	-0.186	-0.171	0.232	0.687	1.458	-0.047
1448	4	7	0.68	6	0.47	3.974	4	0.444	4.09	-0.299	-0.178	0.249	0.654	1.474	-0.085
1449	4	4	0.65	6	0.479	4.194	7	0.438	4.377	-0.41	-0.179	0.263	0.632	1.482	-0.104
1450	4	4	0.639	6	0.513	4.278	7	0.407	4.743	-0.506	-0.168	0.279	0.61	1.487	-0.098
1451	4	4	0.62	6	0.549	4.423	7	0.375	5.181	-0.617	-0.156	0.297	0.598	1.491	-0.093
1452	4	4	0.624	6	0.575	4.392	7	0.352	5.376	-0.665	-0.142	0.318	0.568	1.483	-0.06
145	4	4	0.625	6	0.599	4.383	7	0.331	5.572	-0.715	-0.115	0.345	0.555	1.472	-0.004

3																
1454	4	4	0.642	6	0.62	4.253	7	0.313	5.623	-0.726	-0.108	0.377	0.517	1.449	0.031
1455	4	4	0.655	6	0.644	4.158	7	0.293	5.731	-0.749	-0.095	0.423	0.484	1.425	0.069
1456	4	4	0.685	6	0.659	3.942	7	0.281	5.643	-0.729	-0.108	0.464	0.437	1.385	0.076
1457	4	4	0.711	6	0.672	3.749	7	0.271	5.566	-0.709	-0.105	0.513	0.402	1.345	0.11
1458	4	4	0.745	6	0.677	3.49	7	0.268	5.347	-0.657	-0.125	0.55	0.359	1.294	0.111
1459	4	4	0.776	6	0.687	3.254	7	0.26	5.198	-0.605	-0.139	0.594	0.302	1.258	0.127
1460	4	4	0.801	6	0.674	3.065	7	0.27	4.899	-0.55	-0.167	0.594	0.296	1.205	0.101
1461	4	4	0.824	6	0.66	2.875	7	0.281	4.581	-0.481	-0.183	0.59	0.29	1.16	0.105
1462	4	4	0.837	6	0.641	2.771	7	0.296	4.315	-0.439	-0.21	0.563	0.302	1.119	0.068
1463	4	4	0.849	6	0.618	2.669	7	0.314	4.024	-0.386	-0.226	0.528	0.318	1.083	0.056
1464	4	4	0.856	6	0.592	2.61	7	0.332	3.767	-0.345	-0.241	0.483	0.344	1.05	0.037
1465	4	4	0.861	6	0.567	2.565	7	0.35	3.531	-0.308	-0.258	0.434	0.368	1.018	0.017
146	4	4	0.865	6	0.541	2.531	7	0.368	3.304	-0.27	-0.275	0.383	0.392	0.984	-0.005

6															
1467	4	4	0.867	6	0.513	2.514	7	0.387	3.078	-0.231	-0.296	0.329	0.419	0.946	-0.03
1468	4	4	0.868	6	0.495	2.505	7	0.396	2.95	-0.204	-0.318	0.287	0.424	0.922	-0.066
1469	4	4	0.865	6	0.477	2.528	7	0.406	2.853	-0.178	-0.351	0.242	0.422	0.902	-0.125
1470	4	4	0.861	6	0.467	2.563	7	0.409	2.831	-0.166	-0.37	0.207	0.415	0.902	-0.169
1471	4	4	0.852	6	0.455	2.641	7	0.413	2.836	-0.15	-0.397	0.165	0.402	0.916	-0.232
1472	4	4	0.838	6	0.455	2.761	7	0.409	2.975	-0.157	-0.417	0.133	0.376	0.958	-0.301
1473	4	4	0.823	6	0.445	2.889	7	0.413	3.04	-0.142	-0.422	0.09	0.365	1.014	-0.341
1474	4	4	0.8	6	0.445	3.073	7	0.409	3.238	-0.151	-0.422	0.055	0.344	1.096	-0.389
1475	4	4	0.775	6	0.438	3.265	7	0.413	3.384	-0.146	-0.408	0.015	0.339	1.185	-0.412
1476	4	4	0.745	6	0.437	3.494	7	0.412	3.613	-0.141	-0.399	-0.016	0.317	1.289	-0.443
1477	4	4	0.715	6	0.432	3.714	7	0.414	3.799	-0.13	-0.378	-0.048	0.306	1.39	-0.452
1478	4	4	0.686	6	0.428	3.93	7	0.417	3.982	-0.112	-0.361	-0.073	0.292	1.484	-0.459
147	4	7	0.664	6	0.423	4.091	4	0.421	4.099	-0.085	-0.339	-0.095	0.287	1.564	-0.449

9															
1480	4	7	0.644	6	0.424	4.242	4	0.421	4.261	-0.078	-0.318	-0.103	0.283	1.634	-0.439
1481	4	7	0.639	6	0.428	4.282	4	0.418	4.328	-0.062	-0.295	-0.104	0.285	1.676	-0.415
1482	4	4	0.626	6	0.426	4.377	7	0.426	4.377	-0.079	-0.272	-0.089	0.291	1.707	-0.393
1483	4	4	0.636	6	0.431	4.304	7	0.427	4.322	-0.081	-0.25	-0.062	0.305	1.704	-0.359
1484	4	4	0.651	6	0.453	4.19	7	0.418	4.349	-0.136	-0.222	-0.002	0.327	1.68	-0.327
1485	4	4	0.672	6	0.478	4.033	7	0.407	4.354	-0.198	-0.198	0.077	0.352	1.634	-0.301
1486	4	4	0.702	6	0.508	3.811	7	0.392	4.329	-0.256	-0.193	0.183	0.366	1.553	-0.306
1487	4	4	0.733	6	0.543	3.582	7	0.375	4.322	-0.324	-0.19	0.32	0.389	1.442	-0.317
1488	4	4	0.77	6	0.571	3.306	7	0.361	4.223	-0.355	-0.199	0.477	0.407	1.295	-0.335
1489	4	4	0.795	6	0.601	3.112	7	0.346	4.215	-0.389	-0.209	0.666	0.435	1.119	-0.357
1490	4	4	0.808	6	0.626	3.007	7	0.333	4.267	-0.399	-0.224	0.871	0.459	0.921	-0.382
1491	4	4	0.799	6	0.648	3.075	7	0.321	4.483	-0.403	-0.241	1.09	0.488	0.71	-0.409
149	4	4	0.763	6	0.671	3.357	7	0.306	4.93	-0.415	-0.254	1.318	0.521	0.499	-0.432

2															
1493	4	4	0.708	6	0.687	3.767	7	0.294	5.464	-0.411	-0.257	1.536	0.558	0.293	-0.432
1494	4	4	0.627	6	0.708	4.37	7	0.277	6.243	-0.442	-0.263	1.736	0.592	0.115	-0.451
1495	4	4	0.549	6	0.725	4.962	7	0.263	6.991	-0.467	-0.254	1.906	0.625	-0.034	-0.441
1496	4	4	0.479	6	0.737	5.523	7	0.253	7.665	-0.493	-0.247	2.037	0.658	-0.157	-0.435
1497	4	4	0.43	6	0.75	5.941	7	0.241	8.212	-0.528	-0.235	2.126	0.678	-0.237	-0.421
1498	4	4	0.399	6	0.756	6.222	7	0.234	8.565	-0.551	-0.228	2.176	0.694	-0.297	-0.413
1499	4	4	0.382	6	0.765	6.376	7	0.226	8.811	-0.589	-0.201	2.202	0.714	-0.325	-0.375
1500	4	4	0.39	6	0.76	6.301	7	0.231	8.687	-0.568	-0.21	2.182	0.711	-0.353	-0.374
1501	4	4	0.401	6	0.757	6.204	7	0.233	8.558	-0.555	-0.216	2.157	0.707	-0.373	-0.371
1502	5	5	0.95	6	0.996	1.637	7	0.004	12.824	3.169	-2.198	1.126	-0.099	-1.098	-0.884
1503	5	5	0.949	6	0.996	1.646	7	0.004	12.831	3.267	-2.177	1.104	-0.045	-1.056	-0.875
1504	5	5	0.953	6	0.996	1.596	7	0.004	12.72	3.356	-2.155	1.046	0	-1.005	-0.851
150	5	5	0.961	6	0.995	1.475	7	0.004	12.343	3.403	-2.124	0.981	0.037	-0.93	-0.819

5															
1506	5	5	0.972	6	0.994	1.295	7	0.005	11.855	3.444	-2.081	0.882	0.077	-0.878	-0.739
1507	5	5	0.983	6	0.992	1.056	7	0.007	11.077	3.433	-2.016	0.811	0.118	-0.819	-0.617
1508	5	5	0.99	6	0.99	0.871	7	0.008	10.415	3.409	-1.961	0.741	0.15	-0.782	-0.506
1509	5	5	0.995	6	0.987	0.697	7	0.011	9.693	3.334	-1.904	0.715	0.178	-0.769	-0.391
1510	5	5	0.997	6	0.983	0.589	7	0.014	9.042	3.264	-1.84	0.697	0.216	-0.774	-0.258
1511	5	5	0.998	6	0.978	0.513	7	0.019	8.425	3.142	-1.791	0.728	0.238	-0.793	-0.16
1512	5	5	0.998	6	0.972	0.503	7	0.024	7.929	3.019	-1.749	0.778	0.257	-0.818	-0.084
1513	5	5	0.997	6	0.964	0.543	7	0.03	7.471	2.872	-1.712	0.849	0.265	-0.841	-0.02
1514	5	5	0.996	6	0.956	0.619	7	0.037	7.104	2.719	-1.692	0.935	0.258	-0.839	-0.011
1515	5	5	0.995	6	0.954	0.666	7	0.039	7.083	2.628	-1.709	0.999	0.224	-0.843	-0.06
1516	5	5	0.994	6	0.951	0.733	7	0.041	7.023	2.547	-1.718	1.059	0.197	-0.827	-0.098
1517	5	5	0.994	6	0.954	0.71	7	0.039	7.105	2.525	-1.754	1.072	0.155	-0.786	-0.192
151	5	5	0.995	6	0.954	0.691	7	0.038	7.115	2.513	-1.776	1.074	0.123	-0.732	-0.258

8															
1519	5	5	0.996	6	0.956	0.599	7	0.037	7.096	2.558	-1.795	1.022	0.105	-0.66	-0.312
1520	5	5	0.998	6	0.955	0.501	7	0.037	6.986	2.609	-1.803	0.948	0.09	-0.57	-0.343
1521	5	5	0.999	6	0.954	0.406	7	0.038	6.844	2.692	-1.794	0.848	0.1	-0.483	-0.337
1522	5	5	0.999	6	0.952	0.336	7	0.04	6.673	2.77	-1.782	0.72	0.105	-0.39	-0.315
1523	5	5	0.999	6	0.95	0.317	7	0.041	6.604	2.881	-1.757	0.582	0.115	-0.309	-0.252
1524	5	5	0.999	6	0.949	0.368	7	0.042	6.622	2.982	-1.737	0.435	0.118	-0.235	-0.196
1525	5	5	0.998	6	0.947	0.491	7	0.042	6.734	3.094	-1.707	0.289	0.12	-0.179	-0.097
1526	5	5	0.995	6	0.947	0.675	7	0.041	6.959	3.193	-1.689	0.145	0.109	-0.128	-0.025
1527	5	5	0.99	6	0.945	0.873	7	0.041	7.145	3.259	-1.668	0.024	0.086	-0.084	0.054
1528	5	5	0.981	6	0.943	1.103	7	0.041	7.398	3.331	-1.642	-0.079	0.076	-0.067	0.151
1529	5	5	0.97	6	0.938	1.336	7	0.043	7.504	3.365	-1.603	-0.159	0.062	-0.042	0.261
1530	5	5	0.964	6	0.939	1.437	7	0.042	7.662	3.394	-1.58	-0.182	0.051	-0.07	0.34
153	5	5	0.963	6	0.923	1.453	7	0.052	7.191	3.306	-1.515	-0.148	0.051	-0.069	0.451

1															
1532	5	5	0.968	6	0.91	1.364	7	0.061	6.756	3.205	-1.471	-0.082	0.039	-0.091	0.514
1533	5	5	0.974	6	0.895	1.259	7	0.073	6.263	3.084	-1.421	0.024	0.046	-0.139	0.574
1534	5	5	0.979	6	0.881	1.159	7	0.085	5.834	2.958	-1.382	0.142	0.046	-0.194	0.61
1535	5	5	0.985	6	0.882	1.009	7	0.086	5.673	2.881	-1.373	0.257	0.054	-0.291	0.595
1536	5	5	0.991	6	0.886	0.838	7	0.084	5.547	2.791	-1.381	0.377	0.047	-0.379	0.537
1537	5	5	0.995	6	0.907	0.652	7	0.07	5.775	2.78	-1.42	0.468	0.05	-0.507	0.445
1538	5	5	0.998	6	0.923	0.484	7	0.058	6.006	2.76	-1.47	0.546	0.036	-0.601	0.318
1539	5	5	0.999	6	0.945	0.36	7	0.042	6.589	2.827	-1.544	0.575	0.043	-0.72	0.184
1540	5	5	0.999	6	0.96	0.31	7	0.031	7.178	2.898	-1.612	0.586	0.048	-0.813	0.057
1541	5	5	1	6	0.966	0.284	7	0.026	7.5	2.933	-1.666	0.567	0.053	-0.848	-0.051
1542	5	5	1	6	0.97	0.291	7	0.023	7.775	2.977	-1.707	0.531	0.065	-0.869	-0.133
1543	5	5	1	6	0.967	0.267	7	0.026	7.525	2.95	-1.712	0.48	0.087	-0.826	-0.164
154	5	5	1	6	0.962	0.245	7	0.029	7.231	2.92	-1.717	0.408	0.102	-0.766	-0.194

4															
1545	5	5	1	6	0.947	0.241	7	0.04	6.581	2.832	-1.692	0.344	0.131	-0.674	-0.181
1546	5	5	1	6	0.925	0.289	7	0.055	5.922	2.743	-1.659	0.257	0.16	-0.573	-0.148
1547	5	5	0.999	6	0.9	0.371	7	0.073	5.407	2.662	-1.634	0.168	0.16	-0.469	-0.137
1548	5	5	0.998	6	0.864	0.514	7	0.095	4.923	2.584	-1.602	0.072	0.165	-0.368	-0.107
1549	5	5	0.996	6	0.844	0.644	7	0.105	4.808	2.565	-1.595	-0.042	0.133	-0.289	-0.117
1550	5	5	0.992	6	0.822	0.807	7	0.113	4.767	2.537	-1.599	-0.155	0.09	-0.209	-0.151
1551	5	5	0.987	6	0.818	0.968	7	0.109	5.005	2.564	-1.612	-0.275	0.026	-0.151	-0.197
1552	5	5	0.98	6	0.819	1.139	7	0.101	5.322	2.587	-1.635	-0.376	-0.042	-0.103	-0.267
1553	5	5	0.969	6	0.827	1.346	7	0.089	5.797	2.638	-1.66	-0.48	-0.12	-0.064	-0.342
1554	5	5	0.962	6	0.846	1.454	7	0.076	6.278	2.7	-1.684	-0.53	-0.179	-0.064	-0.416
1555	5	5	0.958	6	0.84	1.522	6	0.078	6.283	2.656	-1.684	-0.52	-0.231	-0.04	-0.481
1556	5	5	0.959	6	0.843	1.508	7	0.076	6.315	2.621	-1.683	-0.474	-0.27	-0.042	-0.546
155	5	5	0.969	6	0.833	1.355	7	0.086	5.901	2.53	-1.651	-0.35	-0.272	-0.058	-0.563

7															
1558	5	5	0.975	6	0.828	1.238	7	0.094	5.586	2.435	-1.627	-0.207	-0.276	-0.081	-0.601
1559	5	5	0.98	6	0.821	1.129	7	0.105	5.246	2.327	-1.599	-0.02	-0.265	-0.118	-0.626
1560	5	5	0.981	6	0.812	1.121	7	0.117	4.997	2.221	-1.57	0.165	-0.254	-0.151	-0.649
1561	5	5	0.978	6	0.807	1.174	7	0.125	4.897	2.131	-1.547	0.354	-0.244	-0.191	-0.671
1562	5	5	0.971	6	0.79	1.313	7	0.14	4.778	2.04	-1.514	0.505	-0.236	-0.206	-0.67
1563	5	5	0.975	6	0.829	1.241	7	0.116	5.172	2.097	-1.538	0.59	-0.247	-0.267	-0.712
1564	5	5	0.976	6	0.858	1.21	7	0.097	5.563	2.147	-1.563	0.649	-0.269	-0.312	-0.755
1565	5	5	0.984	6	0.898	1.032	7	0.071	6.122	2.287	-1.606	0.635	-0.291	-0.379	-0.788
1566	5	5	0.989	6	0.927	0.913	7	0.051	6.725	2.421	-1.65	0.607	-0.32	-0.439	-0.823
1567	5	5	0.99	6	0.951	0.859	7	0.034	7.528	2.573	-1.715	0.532	-0.363	-0.507	-0.882
1568	5	5	0.991	6	0.965	0.826	7	0.023	8.266	2.729	-1.765	0.429	-0.399	-0.571	-0.902
1569	5	5	0.991	6	0.975	0.826	7	0.016	8.991	2.902	-1.8	0.306	-0.42	-0.643	-0.886
157	5	5	0.988	6	0.98	0.93	7	0.012	9.715	3.064	-1.832	0.13	-0.453	-0.691	-0.854

0															
1571	5	5	0.987	6	0.977	0.95	7	0.013	9.586	3.089	-1.807	-0.025	-0.454	-0.687	-0.774
1572	5	5	0.984	6	0.971	1.043	7	0.015	9.449	3.117	-1.773	-0.207	-0.455	-0.668	-0.672
1573	5	5	0.983	6	0.95	1.069	6	0.026	8.253	3.021	-1.686	-0.363	-0.425	-0.599	-0.518
1574	5	5	0.975	6	0.915	1.24	6	0.049	7.113	2.919	-1.608	-0.516	-0.404	-0.528	-0.385
1575	5	5	0.964	6	0.827	1.438	6	0.103	5.604	2.685	-1.498	-0.607	-0.362	-0.424	-0.243
1576	5	5	0.943	6	0.701	1.726	6	0.181	4.435	2.448	-1.405	-0.652	-0.324	-0.342	-0.143
1577	5	5	0.896	6	0.492	2.247	6	0.304	3.214	2.111	-1.293	-0.652	-0.274	-0.238	-0.043
1578	5	6	0.866	6	0.373	2.518	5	0.329	2.771	1.772	-1.212	-0.554	-0.225	-0.175	-0.024
1579	5	6	0.9	6	0.393	2.203	7	0.303	2.721	1.418	-1.143	-0.41	-0.2	-0.104	-0.023
1580	5	6	0.882	6	0.357	2.372	7	0.344	2.447	1.058	-1.096	-0.217	-0.182	-0.044	-0.082
1581	5	7	0.862	6	0.359	2.558	6	0.288	3	0.811	-1.083	0.009	-0.198	-0.002	-0.171
1582	5	4	0.823	6	0.363	2.887	7	0.354	2.936	0.565	-1.075	0.246	-0.216	0.045	-0.276
158	5	4	0.817	6	0.43	2.934	7	0.34	3.406	0.5	-1.096	0.475	-0.277	0.076	-0.371

3															
1584	5	4	0.804	6	0.491	3.042	7	0.323	3.877	0.45	-1.099	0.692	-0.325	0.126	-0.435
1585	5	4	0.753	6	0.513	3.434	7	0.304	4.48	0.528	-1.141	0.893	-0.431	0.164	-0.518
1586	5	4	0.705	6	0.523	3.79	7	0.289	4.979	0.621	-1.159	1.057	-0.525	0.231	-0.55
1587	5	4	0.609	6	0.464	4.501	7	0.259	5.662	0.895	-1.21	1.201	-0.704	0.273	-0.548
1588	5	4	0.494	6	0.376	5.393	5	0.366	5.445	1.169	-1.253	1.323	-0.883	0.321	-0.527
1589	5	5	0.553	6	0.57	4.927	4	0.248	6.587	1.468	-1.32	1.403	-1.062	0.338	-0.515
1590	5	5	0.592	6	0.75	4.628	4	0.14	7.983	1.767	-1.381	1.473	-1.239	0.349	-0.486
1591	5	5	0.651	6	0.871	4.191	4	0.066	9.352	2.029	-1.473	1.473	-1.327	0.296	-0.521
1592	5	5	0.693	6	0.937	3.88	4	0.029	10.844	2.286	-1.564	1.466	-1.414	0.225	-0.543
1593	5	5	0.76	6	0.969	3.377	7	0.016	11.637	2.498	-1.685	1.399	-1.394	0.102	-0.65
1594	5	5	0.801	6	0.985	3.066	7	0.008	12.587	2.695	-1.816	1.328	-1.384	-0.04	-0.769
1595	5	5	0.845	6	0.99	2.706	7	0.006	12.836	2.78	-1.924	1.244	-1.276	-0.184	-0.897
159	5	5	0.876	6	0.993	2.435	7	0.005	13.151	2.869	-2.024	1.157	-1.16	-0.335	-1.004

6																
1597	5	5	0.885	6	0.994	2.351	7	0.004	13.417	2.901	-2.107	1.094	-1.069	-0.443	-1.102
1598	5	5	0.893	6	0.995	2.273	7	0.003	13.644	2.943	-2.18	1.025	-0.967	-0.55	-1.176
1599	5	5	0.897	6	0.996	2.23	7	0.003	13.819	2.979	-2.232	0.969	-0.898	-0.614	-1.214
1600	5	5	0.908	6	0.996	2.128	7	0.003	13.853	3.025	-2.27	0.896	-0.821	-0.663	-1.226
1601	5	5	0.915	6	0.996	2.054	7	0.003	13.979	3.089	-2.303	0.828	-0.771	-0.695	-1.219
1602	5	5	0.933	6	0.997	1.855	7	0.002	13.838	3.166	-2.313	0.727	-0.71	-0.704	-1.164
1603	5	5	0.947	6	0.997	1.681	7	0.002	13.749	3.244	-2.313	0.638	-0.689	-0.691	-1.082
1604	5	5	0.96	6	0.997	1.498	7	0.002	13.594	3.326	-2.304	0.532	-0.665	-0.671	-0.979
1605	5	5	0.972	6	0.996	1.3	7	0.003	13.254	3.361	-2.283	0.439	-0.656	-0.635	-0.862
1606	5	5	0.979	6	0.996	1.156	7	0.003	12.979	3.394	-2.264	0.345	-0.649	-0.603	-0.747
1607	5	5	0.986	6	0.995	0.991	7	0.003	12.519	3.362	-2.24	0.283	-0.651	-0.567	-0.644
1608	5	5	0.99	6	0.994	0.875	7	0.003	12.199	3.326	-2.227	0.247	-0.657	-0.551	-0.566
160	5	5	0.994	6	0.993	0.708	7	0.004	11.646	3.248	-2.2	0.234	-0.657	-0.53	-0.48

9																
1610	5	5	0.997	6	0.992	0.581	7	0.005	11.298	3.167	-2.191	0.277	-0.657	-0.54	-0.437
1611	5	5	0.998	6	0.992	0.481	7	0.005	11.022	3.102	-2.183	0.322	-0.644	-0.563	-0.404
1612	5	5	0.999	6	0.991	0.386	7	0.005	10.782	3.046	-2.172	0.39	-0.621	-0.598	-0.367
1613	5	5	0.999	6	0.992	0.336	7	0.005	10.74	3.038	-2.17	0.446	-0.586	-0.64	-0.34
1614	5	5	1	6	0.992	0.281	7	0.006	10.644	3.04	-2.155	0.498	-0.54	-0.68	-0.287
1615	5	5	1	6	0.992	0.235	7	0.006	10.566	3.088	-2.132	0.512	-0.472	-0.714	-0.198
1616	5	5	1	6	0.991	0.208	7	0.006	10.415	3.136	-2.099	0.491	-0.406	-0.728	-0.086
1617	5	5	1	6	0.991	0.228	7	0.006	10.338	3.192	-2.076	0.45	-0.339	-0.738	0.021
1618	5	5	1	6	0.99	0.286	7	0.007	10.203	3.235	-2.05	0.355	-0.286	-0.725	0.143
1619	5	5	0.999	6	0.987	0.328	7	0.009	9.719	3.203	-2.011	0.269	-0.217	-0.696	0.271
1620	5	5	0.999	6	0.983	0.398	7	0.011	9.296	3.153	-1.988	0.17	-0.168	-0.664	0.371
1621	5	5	0.998	6	0.975	0.473	7	0.016	8.64	3.041	-1.949	0.1	-0.106	-0.634	0.476
162	5	5	0.997	6	0.964	0.574	7	0.023	8.061	2.922	-1.921	0.038	-0.053	-0.608	0.557

2															
1623	5	5	0.994	6	0.946	0.719	7	0.035	7.326	2.761	-1.871	0.02	0.014	-0.586	0.648
1624	5	5	0.989	6	0.92	0.901	7	0.051	6.675	2.601	-1.826	0.024	0.083	-0.567	0.721
1625	5	5	0.977	6	0.876	1.193	7	0.081	5.956	2.41	-1.762	0.051	0.159	-0.54	0.805
1626	5	5	0.948	6	0.81	1.659	7	0.128	5.351	2.245	-1.678	0.093	0.26	-0.508	0.923
1627	5	5	0.898	6	0.726	2.22	7	0.189	4.91	2.103	-1.585	0.137	0.342	-0.464	1.049
1628	5	5	0.828	6	0.621	2.845	7	0.267	4.536	1.96	-1.497	0.183	0.417	-0.408	1.158
1629	5	5	0.77	6	0.539	3.301	7	0.327	4.3	1.858	-1.437	0.216	0.448	-0.345	1.225
1630	5	5	0.702	6	0.454	3.809	7	0.391	4.109	1.755	-1.376	0.245	0.478	-0.278	1.291
1631	5	5	0.684	6	0.427	3.943	7	0.407	4.039	1.715	-1.361	0.257	0.452	-0.225	1.304
1632	5	7	0.683	6	0.416	3.955	5	0.408	3.995	1.66	-1.361	0.27	0.41	-0.182	1.287
1633	5	5	0.72	6	0.442	3.677	7	0.383	3.965	1.661	-1.405	0.272	0.32	-0.155	1.218
1634	5	5	0.772	6	0.491	3.285	7	0.339	4.023	1.643	-1.469	0.288	0.215	-0.157	1.113
163	5	5	0.869	6	0.627	2.497	7	0.241	4.411	1.749	-1.583	0.291	0.063	-0.202	0.97

5															
1636	5	5	0.935	6	0.749	1.827	7	0.156	4.965	1.853	-1.7	0.306	-0.088	-0.264	0.822
1637	5	5	0.977	6	0.855	1.207	7	0.088	5.76	1.974	-1.834	0.346	-0.24	-0.355	0.644
1638	5	5	0.993	6	0.921	0.782	7	0.047	6.738	2.107	-1.957	0.396	-0.377	-0.457	0.489
1639	5	5	0.997	6	0.957	0.531	7	0.026	7.782	2.221	-2.071	0.478	-0.482	-0.576	0.331
1640	5	5	0.999	6	0.977	0.427	7	0.014	8.954	2.356	-2.169	0.58	-0.564	-0.703	0.202
1641	5	5	0.998	6	0.987	0.476	7	0.008	10.096	2.458	-2.257	0.695	-0.62	-0.831	0.07
1642	5	5	0.996	6	0.993	0.641	7	0.005	11.331	2.582	-2.332	0.835	-0.656	-0.953	-0.048
1643	5	5	0.989	6	0.996	0.899	7	0.003	12.497	2.688	-2.405	0.961	-0.653	-1.074	-0.181
1644	5	5	0.977	6	0.997	1.203	7	0.002	13.607	2.82	-2.451	1.085	-0.624	-1.187	-0.262
1645	5	5	0.961	6	0.998	1.476	7	0.001	14.578	2.986	-2.479	1.167	-0.564	-1.28	-0.301
1646	5	5	0.937	6	0.999	1.806	7	0.001	15.569	3.153	-2.505	1.238	-0.505	-1.36	-0.337
1647	5	5	0.913	6	0.999	2.076	7	0.001	16.362	3.337	-2.513	1.264	-0.428	-1.416	-0.33
164	5	5	0.885	6	0.999	2.349	7	0.001	17.09	3.52	-2.514	1.263	-0.353	-1.461	-0.303

8															
1649	5	5	0.852	6	0.999	2.643	7	0.001	17.718	3.71	-2.496	1.244	-0.257	-1.501	-0.232
1650	5	5	0.812	6	0.999	2.972	7	0	18.355	3.887	-2.481	1.196	-0.171	-1.545	-0.151
1651	5	5	0.809	6	0.999	3.002	7	0.001	18.062	3.922	-2.43	1.181	-0.068	-1.553	-0.045
1652	5	5	0.801	6	0.999	3.066	7	0.001	17.833	3.945	-2.388	1.158	0.025	-1.571	0.052
1653	5	5	0.816	6	0.999	2.941	7	0.001	17.146	3.873	-2.344	1.154	0.106	-1.576	0.135
1654	5	5	0.827	6	0.999	2.85	7	0.001	16.506	3.797	-2.302	1.146	0.183	-1.585	0.215
1655	5	5	0.845	6	0.998	2.702	7	0.001	15.891	3.7	-2.282	1.141	0.228	-1.588	0.252
1656	5	5	0.867	6	0.998	2.51	7	0.002	15.23	3.597	-2.27	1.127	0.263	-1.575	0.27
1657	5	5	0.888	6	0.998	2.316	7	0.002	14.793	3.511	-2.285	1.109	0.265	-1.56	0.241
1658	5	5	0.915	6	0.997	2.052	7	0.002	14.181	3.435	-2.289	1.068	0.268	-1.507	0.225
1659	5	5	0.935	6	0.997	1.82	7	0.002	14.005	3.422	-2.326	1.007	0.226	-1.454	0.158
1660	5	5	0.953	6	0.997	1.591	7	0.002	13.737	3.422	-2.349	0.93	0.194	-1.38	0.12
166	5	5	0.968	6	0.997	1.36	7	0.002	13.48	3.441	-2.368	0.832	0.151	-1.284	0.088

1															
1662	5	5	0.979	6	0.997	1.162	7	0.002	13.237	3.46	-2.387	0.728	0.108	-1.181	0.058
1663	5	5	0.987	6	0.997	0.963	7	0.003	12.94	3.458	-2.41	0.623	0.054	-1.063	0.01
1664	5	5	0.991	6	0.997	0.847	7	0.003	12.726	3.462	-2.425	0.526	0.012	-0.96	-0.018
1665	5	5	0.993	6	0.996	0.747	7	0.003	12.393	3.452	-2.424	0.436	-0.017	-0.855	-0.019
1666	5	5	0.995	6	0.996	0.686	7	0.003	12.306	3.416	-2.46	0.385	-0.069	-0.782	-0.096
1667	5	5	0.996	6	0.996	0.646	7	0.003	12.163	3.385	-2.477	0.346	-0.096	-0.723	-0.125
1668	5	5	0.996	6	0.996	0.63	7	0.003	12.13	3.346	-2.507	0.328	-0.13	-0.681	-0.18
1669	5	5	0.996	6	0.996	0.641	7	0.003	12.193	3.328	-2.532	0.32	-0.154	-0.659	-0.214
1670	5	5	0.996	6	0.996	0.65	7	0.003	12.232	3.318	-2.549	0.316	-0.168	-0.642	-0.226
1671	5	5	0.996	6	0.996	0.639	7	0.003	12.198	3.31	-2.553	0.318	-0.172	-0.627	-0.209
1672	5	5	0.996	6	0.996	0.617	7	0.003	12.087	3.318	-2.538	0.316	-0.158	-0.617	-0.147
1673	5	5	0.997	6	0.995	0.561	7	0.004	11.813	3.298	-2.515	0.324	-0.141	-0.596	-0.076
167	5	5	0.997	6	0.994	0.529	7	0.004	11.46	3.296	-2.465	0.317	-0.101	-0.583	0.06

4															
1675	5	5	0.998	6	0.993	0.478	7	0.006	10.835	3.237	-2.401	0.321	-0.061	-0.549	0.208
1676	5	5	0.998	6	0.99	0.51	7	0.008	10.256	3.179	-2.329	0.313	-0.017	-0.517	0.375
1677	5	5	0.997	6	0.987	0.581	7	0.01	9.813	3.115	-2.276	0.294	0.005	-0.489	0.51
1678	5	5	0.994	6	0.983	0.7	7	0.013	9.427	3.048	-2.224	0.272	0.024	-0.464	0.643
1679	5	5	0.992	6	0.981	0.817	7	0.014	9.34	3.015	-2.202	0.241	0.024	-0.467	0.734
1680	5	5	0.989	6	0.98	0.899	7	0.015	9.301	2.975	-2.191	0.23	0.018	-0.486	0.8
1681	5	5	0.986	6	0.981	0.988	7	0.013	9.584	2.995	-2.199	0.212	0.006	-0.537	0.848
1682	5	5	0.986	6	0.984	0.997	7	0.012	9.891	3.006	-2.225	0.222	-0.016	-0.597	0.853
1683	5	5	0.984	6	0.987	1.031	7	0.009	10.407	3.062	-2.261	0.216	-0.039	-0.67	0.845
1684	5	5	0.982	6	0.99	1.091	7	0.007	10.939	3.123	-2.295	0.212	-0.058	-0.738	0.84
1685	5	5	0.979	6	0.991	1.156	7	0.006	11.267	3.16	-2.314	0.196	-0.072	-0.772	0.846
1686	5	5	0.973	6	0.992	1.276	7	0.006	11.593	3.205	-2.321	0.171	-0.078	-0.8	0.877
168	5	5	0.968	6	0.991	1.366	7	0.006	11.591	3.203	-2.31	0.137	-0.08	-0.789	0.922

7															
1688	5	5	0.962	6	0.99	1.461	7	0.006	11.6	3.201	-2.3	0.107	-0.081	-0.782	0.967
1689	5	5	0.957	6	0.989	1.537	7	0.007	11.4	3.16	-2.276	0.083	-0.08	-0.756	1.021
1690	5	5	0.96	6	0.988	1.497	7	0.008	11.251	3.108	-2.276	0.102	-0.088	-0.763	1.026
1691	5	5	0.97	6	0.987	1.325	7	0.008	10.864	3.002	-2.291	0.138	-0.084	-0.776	0.978
1692	5	5	0.978	6	0.986	1.179	7	0.009	10.577	2.901	-2.307	0.206	-0.071	-0.816	0.927
1693	5	5	0.986	6	0.986	0.987	7	0.01	10.214	2.783	-2.339	0.267	-0.049	-0.845	0.823
1694	5	5	0.992	6	0.985	0.797	7	0.01	9.897	2.653	-2.388	0.33	-0.046	-0.862	0.678
1695	5	5	0.995	6	0.985	0.651	7	0.011	9.689	2.554	-2.447	0.357	-0.047	-0.85	0.509
1696	5	5	0.997	6	0.983	0.573	7	0.012	9.459	2.461	-2.498	0.363	-0.048	-0.805	0.35
1697	5	5	0.997	6	0.983	0.541	7	0.012	9.437	2.42	-2.558	0.338	-0.06	-0.748	0.188
1698	5	5	0.997	6	0.983	0.558	7	0.012	9.431	2.395	-2.603	0.324	-0.054	-0.696	0.054
1699	5	5	0.997	6	0.987	0.562	7	0.009	9.924	2.483	-2.663	0.299	-0.078	-0.674	-0.064
170	5	5	0.997	6	0.99	0.582	7	0.007	10.389	2.587	-2.705	0.278	-0.081	-0.665	-0.138

0																
1701	5	5	0.996	6	0.992	0.63	7	0.005	11.041	2.717	-2.748	0.26	-0.102	-0.678	-0.192
1702	5	5	0.994	6	0.994	0.709	7	0.004	11.701	2.85	-2.784	0.237	-0.118	-0.696	-0.227
1703	5	5	0.993	6	0.995	0.782	7	0.003	12.163	2.946	-2.805	0.188	-0.156	-0.685	-0.229
1704	5	5	0.99	6	0.996	0.868	7	0.003	12.56	3.043	-2.813	0.111	-0.189	-0.675	-0.195
1705	5	5	0.988	6	0.996	0.927	7	0.003	12.725	3.092	-2.807	0.035	-0.236	-0.64	-0.147
1706	5	5	0.983	6	0.996	1.063	7	0.003	13.016	3.113	-2.823	-0.063	-0.31	-0.608	-0.135
1707	5	5	0.984	6	0.995	1.048	7	0.003	12.72	3.047	-2.796	-0.115	-0.385	-0.548	-0.064
1708	5	5	0.983	6	0.994	1.058	7	0.003	12.463	2.985	-2.764	-0.16	-0.451	-0.508	0.023
1709	5	5	0.985	6	0.992	1.021	7	0.004	12.148	2.881	-2.735	-0.147	-0.518	-0.495	0.093
1710	5	5	0.985	6	0.991	1.015	7	0.004	11.94	2.771	-2.718	-0.117	-0.59	-0.495	0.139
1711	5	5	0.986	6	0.991	0.976	7	0.004	11.813	2.669	-2.709	-0.045	-0.63	-0.549	0.167
1712	5	5	0.988	6	0.991	0.941	7	0.004	11.744	2.575	-2.699	0.056	-0.659	-0.623	0.197
171	5	5	0.988	6	0.991	0.921	7	0.005	11.708	2.497	-2.69	0.156	-0.647	-0.731	0.226

3															
1714	5	5	0.987	6	0.992	0.951	7	0.004	11.822	2.418	-2.695	0.291	-0.638	-0.846	0.217
1715	5	5	0.985	6	0.993	1.028	7	0.004	12.222	2.4	-2.722	0.393	-0.604	-0.993	0.171
1716	5	5	0.979	6	0.995	1.161	7	0.003	12.669	2.388	-2.745	0.502	-0.565	-1.136	0.13
1717	5	5	0.972	6	0.996	1.302	7	0.003	13.19	2.417	-2.767	0.583	-0.53	-1.259	0.089
1718	5	5	0.961	6	0.997	1.483	7	0.002	13.749	2.441	-2.796	0.656	-0.506	-1.366	0.03
1719	5	5	0.953	6	0.997	1.593	7	0.002	14.117	2.469	-2.812	0.698	-0.492	-1.426	-0.017
1720	6	3	0.678	6	1	3.987	6	0	20.96	-2.161	1.739	-2.628	-1.461	-0.856	-1.75
1721	6	3	0.679	6	0.991	3.984	6	0.005	14.64	-1.375	1.854	-2.239	-1.046	-1.161	-1.434
1722	6	3	0.418	6	0.854	6.047	6	0.077	10.854	-0.663	1.982	-1.833	-0.629	-1.443	-1.13
1723	6	3	0.163	6	0.308	9.188	6	0.255	9.566	-0.196	2.164	-1.358	-0.163	-1.652	-0.766
1724	6	1	0.261	6	0.634	7.7	4	0.177	10.254	0.587	2.455	-0.738	0.459	-1.942	-0.305
1725	6	1	0.168	6	0.496	9.097	6	0.241	10.537	0.77	2.388	-0.851	0.428	-2.057	-0.365
172	6	1	0.144	6	0.438	9.578	6	0.287	10.423	0.778	2.317	-0.902	0.425	-2.124	-0.384

6															
1727	6	1	0.172	6	0.524	9.036	6	0.241	10.59	0.63	2.348	-0.959	0.451	-2.111	-0.375
1728	6	1	0.207	6	0.635	8.442	6	0.18	10.96	0.45	2.382	-1.019	0.493	-2.113	-0.386
1729	6	1	0.217	6	0.663	8.304	6	0.161	11.129	0.409	2.376	-0.992	0.488	-2.152	-0.366
1730	6	1	0.249	6	0.722	7.852	6	0.123	11.396	0.297	2.381	-0.947	0.485	-2.168	-0.359
1731	6	1	0.277	6	0.761	7.501	4	0.108	11.41	0.191	2.397	-0.938	0.472	-2.157	-0.357
1732	6	1	0.479	6	0.909	5.518	4	0.046	11.476	-0.209	2.562	-0.877	0.555	-1.998	-0.173
1733	6	1	0.627	6	0.96	4.371	4	0.021	12.01	-0.781	2.531	-0.61	0.557	-2.063	-0.384
1734	6	1	0.468	6	0.947	5.613	4	0.031	12.47	-0.737	2.361	-0.484	0.456	-2.34	-0.715
1735	6	1	0.364	6	0.948	6.551	4	0.031	13.378	-0.955	2.213	-0.301	0.39	-2.516	-0.985
1736	6	1	0.234	6	0.957	8.061	4	0.027	15.162	-1.186	2.056	-0.037	0.352	-2.76	-1.29
1737	6	1	0.153	6	0.929	9.379	4	0.041	15.644	-1.311	1.93	-0.056	0.229	-2.797	-1.45
1738	6	1	0.097	6	0.898	10.719	4	0.049	16.543	-1.493	1.824	-0.035	0.125	-2.842	-1.606
173	6	1	0.08	6	0.904	11.289	3	0.077	16.218	-2.219	1.896	0.083	0.105	-2.689	-1.698

9																
1740	6	1	0.022	6	0.74	14.749	3	0.228	17.104	-2.418	1.689	0.02	-0.104	-2.855	-1.978
1741	6	1	0.048	6	0.839	12.709	3	0.126	16.505	-2.223	1.767	0.133	-0.015	-2.671	-1.886
1742	6	1	0.077	6	0.869	11.374	3	0.066	16.533	-1.776	1.768	0.211	0.033	-2.592	-1.846
1743	6	1	0.077	6	0.742	11.398	4	0.207	13.95	-1	1.673	0.211	0.029	-2.615	-1.837
1744	6	4	0.048	6	0.553	12.722	1	0.318	13.829	0.014	1.508	0.241	0.003	-2.723	-1.857
1745	6	4	0.072	6	0.606	11.573	1	0.218	13.618	0.174	1.545	0.126	-0.004	-2.487	-1.874
1746	6	4	0.105	6	0.623	10.507	1	0.155	13.292	0.314	1.595	0.019	0.005	-2.247	-1.859
1747	6	4	0.146	6	0.626	9.529	1	0.147	12.425	0.309	1.671	-0.022	0.039	-1.988	-1.842
1748	6	4	0.175	6	0.67	8.976	6	0.144	12.057	0.479	1.65	0.047	0.025	-1.715	-1.992
1749	6	4	0.233	6	0.511	8.074	6	0.318	9.02	0.632	1.759	-0.538	0.041	-1.552	-1.592
1750	6	4	0.291	6	0.389	7.34	1	0.25	8.23	-0.122	2.113	-0.889	0.224	-1.112	-1.035
1751	6	3	0.297	6	0.367	7.262	1	0.336	7.437	-0.541	2.399	-1.338	0.337	-0.77	-0.564
175	6	3	0.305	6	0.725	7.178	1	0.238	9.404	-1.096	2.926	-2.041	0.587	-0.165	0.318

2															
1753	6	3	0.515	6	0.832	5.225	1	0.133	8.888	-1.246	2.643	-1.868	0.379	-0.432	-0.199
1754	6	3	0.625	6	0.894	4.386	1	0.064	9.648	-1.27	2.323	-1.728	0.147	-0.737	-0.739
1755	6	3	0.385	6	0.763	6.349	6	0.129	9.9	-0.607	1.806	-1.731	-0.2	-1.28	-1.389
1756	6	3	0.145	6	0.784	9.557	6	0.119	13.332	-0.691	1.299	-1.565	-0.578	-1.854	-2.185
1757	6	3	0.107	6	0.459	10.436	6	0.352	10.966	-0.162	1.322	-1.605	-0.535	-1.599	-2.177
1758	6	3	0.163	6	0.439	9.199	6	0.321	9.823	-0.176	1.531	-1.511	-0.376	-1.175	-2.05
1759	6	6	0.191	6	0.393	8.704	3	0.281	9.372	0.011	1.653	-1.429	-0.246	-0.844	-1.947
1760	6	6	0.255	6	0.454	7.77	4	0.322	8.454	0.277	1.854	-1.405	-0.075	-0.264	-1.78
1761	6	6	0.254	6	0.445	7.793	4	0.359	8.224	0.293	1.762	-1.271	-0.087	-0.481	-1.854
1762	6	4	0.227	6	0.408	8.159	6	0.406	8.168	0.24	1.683	-1.123	-0.097	-0.676	-1.943
1763	6	4	0.232	6	0.479	8.083	6	0.222	9.625	-0.245	1.745	-0.899	-0.032	-0.711	-1.954
1764	6	4	0.179	6	0.528	8.914	6	0.193	10.933	-0.324	1.594	-0.736	-0.11	-1.023	-2.181
176	6	4	0.241	6	0.542	7.966	6	0.219	9.773	-0.135	1.735	-0.724	0.031	-0.945	-1.934

5															
1766	6	6	0.688	6	0.939	3.92	3	0.024	11.255	0.394	-0.37	-2.426	-0.61	-0.944	1.212
1767	6	6	0.716	6	0.947	3.707	4	0.022	11.21	0.501	-0.493	-2.375	-0.678	-1.095	1.007
1768	6	6	0.719	6	0.951	3.689	4	0.022	11.228	0.61	-0.63	-2.295	-0.753	-1.277	0.771
1769	6	6	0.69	6	0.952	3.902	4	0.022	11.457	0.681	-0.764	-2.232	-0.826	-1.425	0.549
1770	6	6	0.673	6	0.959	4.026	4	0.017	12.106	0.888	-0.793	-2.273	-0.818	-1.475	0.538
1771	6	6	0.657	6	0.961	4.146	4	0.015	12.401	0.891	-0.829	-2.328	-0.867	-1.451	0.483
1772	6	6	0.585	6	0.969	4.68	4	0.011	13.681	0.954	-0.921	-2.53	-0.996	-1.374	0.385
1773	6	6	0.586	6	0.963	4.677	3	0.014	13.108	0.654	-0.877	-2.625	-1.036	-1.223	0.351
1774	6	6	0.558	6	0.927	4.892	3	0.051	10.682	0.303	-0.823	-2.707	-1.092	-1.029	0.29
1775	6	6	0.487	6	0.873	5.453	3	0.108	9.626	0.112	-0.852	-2.847	-1.153	-0.85	0.169
1776	6	6	0.382	6	0.735	6.382	3	0.251	8.533	-0.133	-0.88	-3.009	-1.211	-0.634	0.028
1777	6	6	0.281	6	0.627	7.447	3	0.363	8.544	-0.217	-0.927	-3.189	-1.327	-0.447	-0.092
177	6	6	0.224	6	0.573	8.192	3	0.418	8.824	-0.269	-1.021	-3.206	-1.43	-0.442	-0.295

8																
1779	6	6	0.222	6	0.501	8.219	3	0.489	8.269	-0.388	-1.031	-3.116	-1.421	-0.445	-0.335
1780	6	3	0.269	6	0.525	7.597	6	0.463	7.851	-0.477	-0.999	-3.008	-1.361	-0.441	-0.289
1781	6	3	0.305	6	0.538	7.172	6	0.448	7.536	-0.523	-0.972	-2.917	-1.323	-0.436	-0.277
1782	6	3	0.354	6	0.558	6.651	6	0.426	7.192	-0.589	-0.935	-2.819	-1.255	-0.453	-0.226
1783	6	6	0.385	6	0.509	6.351	3	0.47	6.512	-0.514	-0.831	-2.738	-1.171	-0.553	-0.03
1784	6	6	0.468	6	0.591	5.616	3	0.382	6.49	-0.447	-0.718	-2.635	-1.059	-0.698	0.165
1785	6	6	0.533	6	0.693	5.087	3	0.273	6.947	-0.339	-0.631	-2.528	-1.009	-0.816	0.41
1786	6	6	0.611	6	0.782	4.487	3	0.181	7.41	-0.155	-0.466	-2.558	-0.951	-0.615	0.73
1787	6	6	0.631	6	0.822	4.335	3	0.142	7.851	-0.052	-0.4	-2.568	-0.95	-0.522	0.866
1788	6	6	0.616	6	0.793	4.45	3	0.167	7.566	-0.116	-0.367	-2.524	-0.981	-0.478	0.915
1789	6	6	0.65	6	0.892	4.2	3	0.074	9.189	0.195	-0.435	-2.581	-1.071	-0.52	0.906
1790	6	6	0.641	6	0.945	4.262	3	0.028	11.304	0.529	-0.537	-2.626	-1.198	-0.628	0.872
179	6	6	0.594	6	0.956	4.616	3	0.02	12.35	0.624	-0.658	-2.619	-1.282	-0.861	0.761

1																
1792	6	6	0.526	6	0.966	5.139	4	0.014	13.611	0.789	-0.806	-2.59	-1.335	-1.167	0.617
1793	6	6	0.474	6	0.961	5.562	4	0.018	13.483	0.75	-0.862	-2.426	-1.427	-1.381	0.585
1794	6	6	0.229	6	0.937	8.126	3	0.053	13.879	0.516	-0.922	-3.131	-1.817	-0.93	0.561
1795	6	6	0.109	6	0.88	10.399	3	0.114	14.492	0.397	-0.984	-3.476	-2.075	-0.734	0.554
1796	6	6	0.062	6	0.903	12.008	3	0.092	16.579	0.532	-1.078	-3.613	-2.309	-0.708	0.57
1797	6	6	0.046	6	0.617	12.8	3	0.379	13.776	0.012	-1	-3.678	-2.307	-0.595	0.552
1798	6	3	0.112	6	0.85	10.321	6	0.148	13.817	-0.657	-0.82	-3.668	-2.23	-0.387	0.592
1799	6	3	0.097	6	0.81	10.731	6	0.188	13.653	-0.553	-0.845	-3.722	-2.221	-0.292	0.559
1800	6	3	0.065	6	0.689	11.876	6	0.308	13.484	-0.337	-0.92	-3.802	-2.227	-0.239	0.507
1801	6	3	0.095	6	0.815	10.79	6	0.183	13.778	-0.522	-0.818	-3.815	-2.19	-0.017	0.52
1802	6	3	0.183	6	0.892	8.831	6	0.106	13.094	-0.772	-0.722	-3.567	-2.139	0.056	0.484
1803	6	3	0.223	6	0.887	8.209	6	0.111	12.37	-0.787	-0.749	-3.502	-2.034	0.039	0.323
180	6	3	0.284	6	0.918	7.417	6	0.08	12.288	-0.908	-0.751	-3.474	-1.929	0.101	0.152

4															
1805	6	3	0.218	6	0.862	8.279	6	0.136	11.979	-0.722	-0.869	-3.524	-1.924	0.08	-0.051
1806	6	3	0.15	6	0.708	9.442	6	0.289	11.236	-0.441	-0.982	-3.56	-1.857	0.016	-0.22
1807	6	3	0.149	6	0.543	9.471	6	0.451	9.84	-0.24	-0.896	-3.488	-1.749	0.116	-0.137
1808	6	6	0.186	6	0.563	8.779	3	0.43	9.317	-0.107	-0.774	-3.443	-1.615	0.284	-0.039
1809	6	6	0.234	6	0.816	8.051	3	0.175	11.128	0.298	-0.728	-3.459	-1.584	0.433	0.069
1810	6	6	0.579	6	0.952	4.727	3	0.023	12.188	0.662	-0.923	-2.657	-1.469	-0.493	-0.099
1811	6	6	0.67	6	0.947	4.05	4	0.026	11.229	0.824	-0.942	-2.227	-1.384	-0.949	-0.125
1812	6	6	0.684	6	0.947	3.946	4	0.026	11.142	0.968	-0.893	-2.173	-1.386	-1.024	-0.073
1813	6	6	0.674	6	0.942	4.018	4	0.025	11.267	1.122	-0.91	-2.09	-1.364	-1.146	-0.043
1814	6	6	0.648	6	0.937	4.213	5	0.027	11.28	1.329	-0.916	-2.076	-1.346	-1.233	0.032
1815	6	6	0.669	6	0.954	4.056	5	0.017	12.089	1.383	-0.837	-2.256	-1.337	-1.065	0.206
1816	6	6	0.626	6	0.968	4.373	5	0.012	13.158	1.555	-0.769	-2.496	-1.339	-0.881	0.397
181	6	6	0.677	6	0.975	3.995	4	0.011	13.035	1.369	-0.594	-2.62	-1.276	-0.598	0.634

7															
1818	6	6	0.617	6	0.973	4.446	4	0.012	13.271	1.063	-0.597	-2.7	-1.352	-0.685	0.694
1819	6	6	0.641	6	0.951	4.264	3	0.024	11.623	0.641	-0.439	-2.676	-1.247	-0.607	0.847
1820	6	6	0.627	6	0.866	4.368	3	0.096	8.769	0.16	-0.152	-2.582	-1.027	-0.363	1.135
1821	6	6	0.592	6	0.89	4.634	3	0.076	9.563	0.272	-0.01	-2.67	-0.925	-0.221	1.337
1822	6	6	0.569	6	0.888	4.808	3	0.076	9.716	0.27	0.102	-2.662	-0.838	-0.191	1.456
1823	6	6	0.662	6	0.916	4.106	3	0.044	10.169	0.37	-0.035	-2.495	-0.855	-0.584	1.318
1824	6	6	0.71	6	0.935	3.754	4	0.032	10.488	0.517	-0.205	-2.274	-0.824	-1.09	1.133
1825	6	6	0.65	6	0.919	4.195	4	0.05	10.016	0.531	-0.276	-1.944	-0.879	-1.487	1.095
1826	6	6	0.569	6	0.935	4.807	3	0.033	11.478	0.472	-0.397	-2.491	-1.274	-1.07	0.936
1827	6	6	0.448	6	0.862	5.782	3	0.112	9.865	0.199	-0.353	-2.738	-1.468	-0.719	0.956
1828	6	6	0.36	6	0.821	6.592	3	0.156	9.907	0.16	-0.305	-2.87	-1.62	-0.502	1.041
1829	6	3	0.296	6	0.592	7.28	6	0.389	8.121	-0.442	-0.112	-2.898	-1.598	-0.142	1.187
183	6	3	0.485	6	0.927	5.467	6	0.067	10.736	-1.112	0.096	-2.846	-1.556	0.181	1.342

0															
1831	6	3	0.608	6	0.979	4.513	6	0.018	12.493	-1.556	0.173	-2.72	-1.536	0.268	1.336
1832	6	3	0.676	6	0.997	4.006	6	0.002	16.293	-2.251	0.301	-2.551	-1.505	0.426	1.344
1833	6	3	0.706	6	0.996	3.786	6	0.003	15.507	-2.153	0.182	-2.592	-1.597	0.307	1.201
1834	6	3	0.697	6	0.986	3.85	6	0.009	13.288	-1.891	0.322	-2.311	-1.405	0.383	1.369
1835	6	3	0.646	6	0.903	4.226	6	0.075	9.199	-1.239	0.201	-2.279	-1.347	0.14	1.277
1836	6	3	0.495	6	0.632	5.385	6	0.323	6.724	-0.676	0.008	-2.368	-1.387	-0.161	1.102
1837	6	6	0.51	6	0.685	5.269	3	0.26	7.206	-0.191	-0.174	-2.399	-1.409	-0.464	0.953
1838	6	6	0.584	6	0.902	4.69	3	0.061	10.088	0.351	-0.365	-2.506	-1.457	-0.732	0.793
1839	6	6	0.543	6	0.953	5.004	3	0.024	12.346	0.685	-0.467	-2.692	-1.473	-0.808	0.776
1840	6	6	0.424	6	0.98	5.992	4	0.007	15.764	1.136	-0.628	-2.938	-1.51	-0.93	0.72
1841	6	6	0.392	6	0.981	6.281	3	0.007	16.064	1.132	-0.609	-3.018	-1.515	-0.914	0.766
1842	6	6	0.387	6	0.986	6.331	4	0.005	16.892	1.336	-0.39	-3.148	-1.363	-0.584	1.088
184	6	6	0.383	6	0.987	6.367	4	0.005	17.08	1.464	-0.311	-3.146	-1.279	-0.487	1.221

3																
1844	6	6	0.434	6	0.986	5.9	4	0.005	16.312	1.568	-0.337	-2.971	-1.234	-0.656	1.21
1845	6	6	0.509	6	0.982	5.277	4	0.007	15.117	1.515	-0.313	-2.798	-1.141	-0.781	1.225
1846	6	6	0.595	6	0.977	4.606	4	0.01	13.78	1.426	-0.283	-2.624	-1.051	-0.855	1.215
1847	6	6	0.65	6	0.975	4.2	4	0.011	13.239	1.456	-0.298	-2.578	-1.038	-0.775	1.13
1848	6	6	0.705	6	0.973	3.787	7	0.012	12.65	1.481	-0.293	-2.537	-1.011	-0.653	1.063
1849	6	6	0.732	6	0.971	3.587	7	0.013	12.187	1.588	-0.304	-2.514	-1	-0.533	0.998
1850	6	6	0.725	6	0.953	3.643	4	0.018	11.552	1.382	-0.688	-2.183	-1.214	-1.139	0.385
1851	6	6	0.683	6	0.941	3.954	4	0.024	11.325	1.257	-0.78	-2.045	-1.257	-1.335	0.231
1852	6	6	0.747	6	0.945	3.478	4	0.024	10.837	1.267	-0.658	-2.054	-1.152	-1.249	0.445
1853	6	6	0.751	6	0.95	3.444	4	0.019	11.231	1.434	-0.576	-2.112	-1.082	-1.192	0.603
1854	6	6	0.669	6	0.951	4.056	5	0.02	11.759	1.749	-0.558	-2.197	-1.059	-1.215	0.722
1855	6	6	0.772	6	0.947	3.289	4	0.022	10.859	1.433	-0.438	-2.044	-0.93	-1.24	0.803
185	6	6	0.843	6	0.928	2.72	4	0.039	9.044	1.023	-0.286	-1.879	-0.773	-1.241	0.886

6															
1857	6	6	0.828	6	0.914	2.842	4	0.05	8.674	0.942	-0.247	-1.758	-0.718	-1.326	0.959
1858	6	6	0.753	6	0.88	3.428	4	0.072	8.446	0.895	-0.161	-1.5	-0.57	-1.545	1.136
1859	6	6	0.716	6	0.868	3.708	4	0.08	8.475	0.875	-0.131	-1.426	-0.53	-1.606	1.208
1860	6	6	0.735	6	0.87	3.57	4	0.082	8.293	0.79	-0.107	-1.477	-0.557	-1.508	1.242
1861	6	6	0.753	6	0.885	3.433	4	0.071	8.484	0.813	-0.114	-1.573	-0.576	-1.44	1.243
1862	6	6	0.76	6	0.9	3.379	4	0.061	8.778	0.842	-0.136	-1.666	-0.607	-1.403	1.23
1863	6	6	0.768	6	0.905	3.32	4	0.059	8.788	0.755	-0.194	-1.725	-0.666	-1.404	1.127
1864	6	6	0.766	6	0.907	3.33	4	0.059	8.799	0.646	-0.249	-1.775	-0.726	-1.403	1.029
1865	6	6	0.753	6	0.912	3.433	4	0.055	9.05	0.606	-0.324	-1.824	-0.781	-1.457	0.9
1866	6	6	0.766	6	0.93	3.331	4	0.034	9.934	0.48	-0.373	-2.243	-0.938	-1.082	0.75
1867	6	6	0.735	6	0.944	3.565	4	0.023	10.963	0.567	-0.445	-2.468	-1.07	-0.916	0.64
1868	6	6	0.731	6	0.953	3.595	4	0.02	11.303	0.688	-0.494	-2.504	-1.119	-0.903	0.57
186	6	6	0.71	6	0.964	3.75	4	0.016	11.928	0.862	-0.573	-2.555	-1.184	-0.901	0.469

9																
1870	6	6	0.673	6	0.971	4.031	4	0.013	12.719	1.059	-0.64	-2.605	-1.24	-0.918	0.41
1871	6	6	0.635	6	0.976	4.312	4	0.009	13.581	1.263	-0.633	-2.66	-1.199	-0.989	0.508
1872	6	6	0.566	6	0.979	4.828	7	0.007	14.701	1.514	-0.628	-2.728	-1.151	-1.074	0.62
1873	6	6	0.534	6	0.98	5.078	7	0.007	14.929	1.624	-0.586	-2.754	-1.101	-1.108	0.743
1874	6	6	0.462	6	0.979	5.663	5	0.009	15.101	1.812	-0.583	-2.768	-1.066	-1.221	0.835
1875	6	6	0.49	6	0.982	5.432	7	0.007	15.232	1.691	-0.477	-2.791	-0.99	-1.145	1.002
1876	6	6	0.493	6	0.984	5.407	7	0.007	15.218	1.575	-0.348	-2.846	-0.911	-1.031	1.201
1877	6	6	0.467	6	0.984	5.623	7	0.007	15.421	1.491	-0.233	-2.912	-0.818	-0.905	1.41
1878	6	6	0.438	6	0.984	5.87	7	0.007	15.712	1.375	-0.168	-2.96	-0.759	-0.828	1.555
1879	6	6	0.472	6	0.981	5.579	4	0.007	15.575	1.106	-0.203	-2.964	-0.808	-0.856	1.443
1880	6	6	0.489	6	0.971	5.436	3	0.014	13.872	0.817	-0.216	-2.967	-0.847	-0.845	1.351
1881	6	6	0.484	6	0.961	5.476	3	0.024	12.856	0.657	-0.257	-2.979	-0.879	-0.889	1.259
188	6	6	0.316	6	0.827	7.055	3	0.163	10.308	0.101	-0.571	-3.178	-1.225	-1.037	0.702

2															
188<br>3	6	6	0.184	6	0.678	8.815	3	0.315	10.345	-0.105	-0.764	-3.331	-1.461	-1.132	0.418
188<br>4	6	6	0.13	6	0.573	9.879	3	0.422	10.493	-0.192	-0.806	-3.484	-1.554	-1.092	0.378
188<br>5	6	6	0.083	6	0.536	11.171	3	0.46	11.474	-0.181	-0.891	-3.652	-1.69	-1.12	0.306
188<br>6	6	6	0.058	6	0.604	12.19	3	0.393	13.049	-0.04	-0.977	-3.815	-1.798	-1.157	0.232
188<br>7	6	6	0.067	6	0.549	11.786	3	0.448	12.191	-0.108	-0.901	-3.779	-1.799	-1.008	0.337
188<br>8	6	3	0.087	6	0.567	11.035	6	0.43	11.585	-0.25	-0.807	-3.75	-1.794	-0.807	0.466
188<br>9	6	3	0.075	6	0.504	11.483	6	0.493	11.524	-0.137	-0.767	-3.798	-1.836	-0.683	0.528
189<br>0	6	6	0.132	6	0.781	9.828	3	0.213	12.432	0.153	-0.814	-3.457	-1.806	-1.041	0.491
189<br>1	6	6	0.194	6	0.693	8.652	3	0.296	10.357	-0.091	-0.7	-3.119	-1.699	-1.075	0.601
189<br>2	6	6	0.219	6	0.519	8.276	3	0.466	8.489	-0.362	-0.557	-2.945	-1.636	-0.914	0.754
189<br>3	6	3	0.3	6	0.596	7.231	6	0.387	8.093	-0.56	-0.437	-2.812	-1.559	-0.795	0.859
189<br>4	6	3	0.41	6	0.723	6.121	6	0.259	8.174	-0.778	-0.306	-2.666	-1.486	-0.654	0.984
189	6	3	0.493	6	0.804	5.403	6	0.181	8.387	-0.908	-0.183	-2.664	-1.408	-0.456	1.069

5															
1896	6	3	0.541	6	0.841	5.019	6	0.144	8.542	-0.98	-0.055	-2.642	-1.344	-0.26	1.169
1897	6	3	0.633	6	0.912	4.322	6	0.078	9.237	-1.202	0.088	-2.623	-1.175	-0.044	1.256
1898	6	7	0.97	6	0.845	1.323	6	0.091	5.789	1.406	-0.326	0.058	1.249	1.072	0.764
1899	6	7	0.966	6	0.835	1.4	6	0.102	5.613	1.397	-0.336	-0.031	1.202	1.165	0.731
1900	6	7	0.959	6	0.828	1.505	6	0.113	5.498	1.502	-0.354	-0.119	1.167	1.206	0.725
1901	6	7	0.949	6	0.822	1.644	6	0.123	5.441	1.604	-0.37	-0.198	1.136	1.242	0.721
1902	6	7	0.939	6	0.815	1.775	6	0.133	5.405	1.736	-0.397	-0.266	1.107	1.248	0.706
1903	6	7	0.928	6	0.807	1.91	6	0.141	5.394	1.87	-0.428	-0.321	1.077	1.237	0.687
1904	6	7	0.918	6	0.797	2.022	6	0.149	5.374	1.976	-0.464	-0.366	1.044	1.229	0.646
1905	6	7	0.905	6	0.785	2.159	6	0.156	5.39	2.109	-0.503	-0.404	1.016	1.185	0.616
1906	6	7	0.911	6	0.784	2.089	6	0.157	5.302	2.088	-0.509	-0.402	1.007	1.183	0.587
1907	6	7	0.924	6	0.778	1.951	6	0.159	5.122	2.055	-0.54	-0.395	0.977	1.177	0.509
190	6	7	0.938	6	0.779	1.794	6	0.158	4.981	1.991	-0.549	-0.374	0.966	1.176	0.461

8																
1909	6	7	0.949	6	0.78	1.644	6	0.157	4.854	1.92	-0.556	-0.35	0.957	1.176	0.413
1910	6	7	0.958	6	0.777	1.52	6	0.156	4.727	1.874	-0.593	-0.327	0.925	1.171	0.318
1911	6	7	0.965	6	0.778	1.422	6	0.153	4.679	1.83	-0.614	-0.296	0.91	1.168	0.251
1912	6	7	0.965	6	0.773	1.407	6	0.151	4.667	1.852	-0.653	-0.28	0.882	1.147	0.174
1913	6	7	0.966	6	0.773	1.4	6	0.145	4.744	1.883	-0.686	-0.245	0.867	1.125	0.104
1914	6	7	0.964	6	0.772	1.424	6	0.139	4.859	1.931	-0.722	-0.205	0.85	1.096	0.034
1915	6	7	0.961	6	0.769	1.475	6	0.13	5.037	1.992	-0.757	-0.157	0.836	1.061	-0.036
1916	6	7	0.962	6	0.772	1.455	6	0.118	5.219	2.003	-0.775	-0.082	0.836	1.033	-0.095
1917	6	7	0.961	6	0.771	1.475	6	0.106	5.436	2.012	-0.805	-0.003	0.826	1.004	-0.177
1918	6	7	0.964	6	0.778	1.426	6	0.095	5.63	2	-0.805	0.084	0.839	0.975	-0.21
1919	6	7	0.967	6	0.783	1.383	5	0.092	5.668	1.987	-0.799	0.148	0.852	0.95	-0.225
1920	6	7	0.969	6	0.789	1.34	5	0.092	5.637	1.969	-0.791	0.219	0.866	0.925	-0.239
192	6	7	0.973	6	0.793	1.283	5	0.085	5.743	1.929	-0.779	0.224	0.862	0.933	-0.236

1															
1922	6	7	0.974	6	0.791	1.258	6	0.085	5.729	1.925	-0.769	0.198	0.852	0.938	-0.222
1923	6	7	0.976	6	0.79	1.219	6	0.093	5.496	1.926	-0.744	0.131	0.846	0.958	-0.166
1924	6	7	0.977	6	0.784	1.191	6	0.11	5.125	1.905	-0.713	0.016	0.83	1	-0.095
1925	6	7	0.976	6	0.774	1.217	6	0.131	4.77	1.894	-0.677	-0.115	0.816	1.043	-0.005
1926	6	7	0.974	6	0.758	1.266	6	0.159	4.387	1.839	-0.626	-0.261	0.809	1.097	0.105
1927	6	7	0.967	6	0.734	1.38	6	0.192	4.068	1.781	-0.589	-0.401	0.791	1.143	0.19
1928	6	7	0.957	6	0.706	1.537	6	0.227	3.803	1.687	-0.533	-0.539	0.788	1.195	0.299
1929	6	7	0.946	6	0.681	1.691	6	0.256	3.644	1.595	-0.482	-0.639	0.79	1.232	0.391
1930	6	7	0.931	6	0.656	1.871	6	0.284	3.545	1.518	-0.445	-0.729	0.79	1.262	0.46
1931	6	7	0.918	6	0.633	2.016	6	0.306	3.472	1.424	-0.419	-0.792	0.783	1.294	0.491
1932	6	7	0.912	6	0.625	2.083	6	0.315	3.454	1.412	-0.418	-0.82	0.775	1.308	0.484
1933	6	7	0.908	6	0.62	2.12	6	0.318	3.454	1.386	-0.418	-0.831	0.767	1.329	0.462
193	6	7	0.914	6	0.629	2.062	6	0.31	3.478	1.448	-0.455	-0.808	0.746	1.334	0.38

4																
1935	6	7	0.923	6	0.652	1.96	6	0.287	3.6	1.515	-0.488	-0.745	0.74	1.341	0.289
1936	6	7	0.927	6	0.673	1.92	6	0.265	3.788	1.644	-0.545	-0.683	0.722	1.334	0.172
1937	6	7	0.933	6	0.712	1.845	6	0.222	4.172	1.804	-0.576	-0.563	0.745	1.31	0.094
1938	6	7	0.927	6	0.74	1.914	6	0.185	4.688	2.013	-0.622	-0.447	0.764	1.263	0.006
1939	6	7	0.91	6	0.756	2.105	6	0.146	5.396	2.236	-0.668	-0.311	0.791	1.204	-0.08
1940	6	7	0.886	6	0.763	2.34	6	0.112	6.178	2.451	-0.671	-0.175	0.857	1.126	-0.082
1941	6	7	0.839	6	0.749	2.758	5	0.152	5.943	2.685	-0.671	-0.043	0.928	1.037	-0.066
1942	6	7	0.804	6	0.742	3.035	5	0.181	5.859	2.824	-0.644	0.082	1.007	0.964	-0.027
1943	6	7	0.751	6	0.729	3.448	5	0.208	5.952	2.981	-0.602	0.166	1.092	0.886	0.066
1944	6	7	0.718	6	0.748	3.695	5	0.199	6.339	3.055	-0.524	0.26	1.197	0.831	0.192
1945	6	7	0.687	6	0.759	3.923	5	0.192	6.678	3.11	-0.468	0.289	1.266	0.789	0.3
1946	6	7	0.687	6	0.798	3.921	5	0.154	7.207	3.08	-0.383	0.328	1.342	0.777	0.419
194	6	7	0.689	6	0.831	3.908	5	0.122	7.751	3.036	-0.304	0.345	1.407	0.771	0.533

7																
1948	6	7	0.709	6	0.85	3.759	5	0.101	8.012	2.974	-0.258	0.351	1.431	0.775	0.574
1949	6	7	0.739	6	0.866	3.54	5	0.084	8.206	2.895	-0.222	0.357	1.445	0.784	0.592
1950	6	7	0.782	6	0.866	3.209	5	0.08	7.965	2.827	-0.235	0.356	1.409	0.795	0.513
1951	6	7	0.827	6	0.867	2.856	5	0.077	7.709	2.745	-0.255	0.359	1.366	0.814	0.414
1952	6	7	0.859	6	0.859	2.588	5	0.08	7.324	2.695	-0.294	0.352	1.305	0.826	0.286
1953	6	7	0.887	6	0.849	2.33	5	0.086	6.904	2.632	-0.351	0.351	1.228	0.849	0.116
1954	6	7	0.903	6	0.837	2.178	5	0.094	6.55	2.578	-0.407	0.348	1.149	0.869	-0.056
1955	6	7	0.908	6	0.823	2.126	5	0.103	6.273	2.531	-0.464	0.346	1.07	0.887	-0.229
1956	6	7	0.911	6	0.816	2.089	5	0.106	6.161	2.465	-0.501	0.359	1.008	0.904	-0.374
1957	6	7	0.908	6	0.807	2.121	5	0.111	6.096	2.403	-0.541	0.373	0.947	0.917	-0.521
1958	6	7	0.906	6	0.804	2.145	5	0.109	6.137	2.329	-0.564	0.401	0.901	0.925	-0.641
1959	6	7	0.899	6	0.801	2.218	5	0.108	6.225	2.255	-0.587	0.429	0.855	0.934	-0.761
196	6	7	0.891	6	0.8	2.289	5	0.105	6.347	2.186	-0.6	0.463	0.82	0.936	-0.857

0															
1961	6	7	0.886	6	0.802	2.34	5	0.098	6.554	2.12	-0.598	0.482	0.794	0.954	-0.927
1962	6	7	0.872	6	0.798	2.471	5	0.098	6.669	2.09	-0.608	0.49	0.759	0.967	-0.999
1963	6	7	0.855	6	0.795	2.614	5	0.096	6.84	2.058	-0.616	0.488	0.722	0.992	-1.068
1964	6	7	0.847	6	0.794	2.688	5	0.093	6.977	2.058	-0.606	0.461	0.699	1.025	-1.092
1965	6	7	0.837	6	0.794	2.768	5	0.089	7.136	2.056	-0.596	0.431	0.675	1.063	-1.114
1966	6	7	0.839	6	0.797	2.755	5	0.084	7.265	2.074	-0.569	0.385	0.669	1.099	-1.096
1967	6	7	0.846	6	0.8	2.693	5	0.078	7.354	2.097	-0.534	0.34	0.671	1.125	-1.058
1968	6	7	0.852	6	0.803	2.642	6	0.073	7.436	2.118	-0.499	0.291	0.677	1.151	-1.024
1969	6	7	0.875	6	0.81	2.44	6	0.076	7.163	2.153	-0.441	0.248	0.709	1.147	-0.929
1970	6	7	0.894	6	0.815	2.26	6	0.08	6.903	2.21	-0.38	0.194	0.753	1.127	-0.825
1971	6	7	0.909	6	0.818	2.11	6	0.084	6.667	2.262	-0.322	0.143	0.795	1.097	-0.726
1972	6	7	0.922	6	0.821	1.969	6	0.088	6.428	2.319	-0.262	0.089	0.842	1.052	-0.611
197	6	7	0.93	6	0.822	1.879	6	0.093	6.234	2.375	-0.203	0.034	0.889	1.004	-0.499

3																
1974	6	7	0.936	6	0.821	1.813	6	0.099	6.045	2.411	-0.158	-0.017	0.923	0.954	-0.409
1975	6	7	0.939	6	0.818	1.777	6	0.105	5.882	2.44	-0.123	-0.063	0.948	0.902	-0.343
1976	6	7	0.941	6	0.816	1.747	6	0.111	5.741	2.467	-0.08	-0.107	0.982	0.849	-0.251
1977	6	7	0.944	6	0.811	1.714	6	0.116	5.604	2.474	-0.064	-0.135	0.992	0.798	-0.226
1978	6	7	0.944	6	0.808	1.713	6	0.12	5.527	2.496	-0.043	-0.158	1.011	0.746	-0.17
1979	6	7	0.944	6	0.804	1.712	6	0.123	5.462	2.511	-0.029	-0.173	1.024	0.694	-0.136
1980	6	7	0.942	6	0.801	1.743	6	0.126	5.444	2.533	-0.021	-0.186	1.03	0.657	-0.116
1981	6	7	0.94	6	0.797	1.763	6	0.129	5.412	2.547	-0.022	-0.196	1.028	0.626	-0.116
1982	6	7	0.936	6	0.792	1.819	6	0.131	5.411	2.568	-0.026	-0.212	1.018	0.616	-0.13
1983	6	7	0.929	6	0.788	1.897	6	0.134	5.437	2.593	-0.024	-0.232	1.011	0.62	-0.139
1984	6	7	0.92	6	0.783	1.996	6	0.138	5.464	2.621	-0.032	-0.256	0.993	0.632	-0.169
1985	6	7	0.909	6	0.776	2.113	6	0.142	5.505	2.647	-0.043	-0.284	0.967	0.663	-0.215
198	6	7	0.895	6	0.77	2.248	6	0.147	5.566	2.675	-0.056	-0.311	0.941	0.693	-0.26

6															
198<br>7	6	7	0.88	6	0.762	2.396	6	0.151	5.632	2.7	-0.078	-0.336	0.906	0.727	-0.327
198<br>8	6	7	0.868	6	0.753	2.508	6	0.155	5.674	2.703	-0.11	-0.351	0.864	0.762	-0.413
198<br>9	6	7	0.851	6	0.744	2.653	6	0.158	5.749	2.711	-0.144	-0.365	0.822	0.792	-0.501
199<br>0	6	7	0.844	6	0.742	2.714	6	0.16	5.787	2.706	-0.153	-0.371	0.803	0.82	-0.539
199<br>1	6	7	0.833	6	0.736	2.802	6	0.164	5.811	2.695	-0.177	-0.383	0.77	0.846	-0.605
199<br>2	6	7	0.834	6	0.735	2.794	6	0.166	5.773	2.649	-0.189	-0.382	0.743	0.883	-0.661
199<br>3	6	7	0.834	6	0.732	2.794	6	0.173	5.681	2.627	-0.182	-0.41	0.729	0.912	-0.659
199<br>4	6	7	0.856	6	0.736	2.607	6	0.18	5.42	2.515	-0.165	-0.427	0.72	0.965	-0.672
199<br>5	6	7	0.873	6	0.733	2.461	6	0.191	5.154	2.398	-0.152	-0.456	0.705	1.019	-0.689
199<br>6	6	7	0.893	6	0.725	2.277	6	0.206	4.797	2.282	-0.115	-0.504	0.702	1.065	-0.645
199<br>7	6	7	0.907	6	0.712	2.131	6	0.222	4.459	2.159	-0.079	-0.556	0.696	1.11	-0.602
199<br>8	6	7	0.92	6	0.693	1.997	6	0.244	4.084	2.06	-0.027	-0.628	0.703	1.133	-0.505
199	6	7	0.928	6	0.669	1.906	6	0.269	3.725	1.954	0.02	-0.702	0.706	1.149	-0.412

9																
200<br>0	6	7	0.932	6	0.643	1.861	6	0.297	3.407	1.861	0.084	-0.785	0.722	1.156	-0.278
200<br>1	6	7	0.932	6	0.617	1.864	6	0.323	3.155	1.774	0.155	-0.862	0.748	1.15	-0.124
200<br>2	6	7	0.924	6	0.593	1.953	6	0.347	3.024	1.699	0.236	-0.937	0.788	1.14	0.052
200<br>3	6	7	0.907	6	0.572	2.132	6	0.369	3.006	1.619	0.321	-1.004	0.833	1.13	0.232
200<br>4	6	7	0.892	6	0.556	2.278	6	0.385	3.014	1.572	0.372	-1.048	0.861	1.11	0.344
200<br>5	6	7	0.874	6	0.544	2.451	6	0.396	3.089	1.518	0.429	-1.081	0.895	1.092	0.462
200<br>6	6	7	0.874	6	0.539	2.447	6	0.402	3.03	1.525	0.434	-1.091	0.9	1.054	0.484
200<br>7	6	7	0.891	6	0.541	2.295	6	0.398	2.908	1.512	0.42	-1.067	0.893	1.023	0.45
200<br>8	6	7	0.91	6	0.543	2.106	6	0.398	2.728	1.565	0.378	-1.048	0.872	0.969	0.381
200<br>9	6	7	0.936	6	0.557	1.817	6	0.382	2.574	1.611	0.322	-0.987	0.845	0.926	0.261
201<br>0	6	7	0.954	6	0.568	1.579	6	0.371	2.429	1.691	0.234	-0.932	0.794	0.875	0.088
201<br>1	6	7	0.963	6	0.58	1.439	6	0.357	2.411	1.775	0.14	-0.869	0.739	0.829	-0.103
201	6	7	0.967	6	0.595	1.389	6	0.339	2.515	1.835	0.056	-0.797	0.69	0.798	-0.283

2															
2013	6	7	0.962	6	0.608	1.456	6	0.321	2.735	1.903	-0.028	-0.727	0.642	0.765	-0.465
2014	6	7	0.959	6	0.624	1.514	6	0.298	2.989	1.93	-0.092	-0.644	0.608	0.741	-0.614
2015	6	7	0.951	6	0.636	1.619	6	0.278	3.272	1.962	-0.154	-0.564	0.579	0.7	-0.749
2016	6	7	0.944	6	0.649	1.713	6	0.255	3.584	1.963	-0.207	-0.47	0.555	0.667	-0.875
2017	6	7	0.946	6	0.66	1.684	6	0.236	3.738	1.987	-0.226	-0.396	0.565	0.595	-0.907
2018	6	7	0.951	6	0.668	1.622	6	0.221	3.839	1.975	-0.245	-0.321	0.569	0.535	-0.931
2019	6	7	0.961	6	0.676	1.484	6	0.208	3.837	1.979	-0.242	-0.263	0.592	0.459	-0.897
2020	6	7	0.972	6	0.674	1.293	6	0.212	3.605	1.952	-0.226	-0.265	0.61	0.417	-0.818
2021	6	7	0.981	6	0.669	1.114	6	0.22	3.337	1.933	-0.203	-0.285	0.628	0.378	-0.72
2022	6	7	0.99	6	0.656	0.881	6	0.245	2.851	1.895	-0.141	-0.379	0.663	0.384	-0.529
2023	6	7	0.992	6	0.63	0.804	6	0.282	2.411	1.855	-0.079	-0.513	0.686	0.423	-0.336
2024	6	7	0.991	6	0.595	0.854	6	0.328	2.045	1.785	-0.004	-0.671	0.714	0.485	-0.116
202	6	7	0.981	6	0.551	1.113	6	0.381	1.852	1.708	0.062	-0.841	0.724	0.583	0.069

5															
2026	6	7	0.961	6	0.511	1.473	6	0.428	1.828	1.625	0.116	-0.992	0.74	0.679	0.237
2027	6	7	0.927	6	0.475	1.916	6	0.467	1.952	1.534	0.17	-1.123	0.76	0.781	0.392
2028	6	6	0.916	6	0.483	2.043	7	0.462	2.134	1.51	0.165	-1.179	0.759	0.84	0.437
2029	6	6	0.902	6	0.488	2.18	7	0.458	2.31	1.479	0.17	-1.211	0.772	0.896	0.49
2030	6	7	0.906	6	0.475	2.148	6	0.473	2.157	1.543	0.108	-1.171	0.759	0.895	0.426
2031	6	7	0.931	6	0.512	1.873	6	0.435	2.198	1.611	0.056	-1.078	0.771	0.873	0.372
2032	6	7	0.948	6	0.552	1.668	6	0.396	2.335	1.779	-0.017	-0.98	0.779	0.814	0.307
2033	6	7	0.964	6	0.605	1.437	6	0.339	2.595	1.955	-0.099	-0.829	0.796	0.721	0.22
2034	6	7	0.969	6	0.652	1.343	6	0.285	2.998	2.119	-0.162	-0.678	0.824	0.629	0.156
2035	6	7	0.963	6	0.689	1.45	6	0.232	3.628	2.317	-0.215	-0.525	0.869	0.514	0.13
2036	6	7	0.964	6	0.718	1.429	6	0.192	4.064	2.382	-0.229	-0.389	0.922	0.445	0.135
2037	6	7	0.957	6	0.736	1.544	6	0.16	4.591	2.471	-0.243	-0.269	0.975	0.368	0.156
203	6	7	0.966	6	0.764	1.397	6	0.142	4.755	2.411	-0.205	-0.184	1.043	0.358	0.237

8																
2039	6	7	0.97	6	0.78	1.336	6	0.137	4.811	2.356	-0.167	-0.164	1.093	0.376	0.331
2040	6	7	0.98	6	0.8	1.13	6	0.133	4.725	2.195	-0.101	-0.141	1.152	0.436	0.442
2041	6	7	0.985	6	0.798	1.019	6	0.144	4.438	2.001	-0.04	-0.214	1.175	0.56	0.548
2042	6	7	0.985	6	0.785	1.028	6	0.159	4.215	1.823	-0.001	-0.298	1.176	0.683	0.63
2043	6	7	0.975	6	0.763	1.241	6	0.181	4.12	1.631	0.052	-0.412	1.179	0.829	0.734
2044	6	7	0.961	6	0.737	1.482	6	0.205	4.039	1.51	0.063	-0.517	1.159	0.925	0.799
2045	6	7	0.938	6	0.709	1.787	6	0.23	4.04	1.38	0.076	-0.615	1.142	1.018	0.864
2046	6	7	0.925	6	0.691	1.946	6	0.247	4.006	1.315	0.062	-0.665	1.128	1.032	0.913
2047	6	7	0.929	6	0.683	1.898	6	0.25	3.908	1.242	0.035	-0.651	1.121	0.993	0.932
2048	6	7	0.934	6	0.681	1.832	6	0.25	3.834	1.213	0.001	-0.625	1.122	0.919	0.968
2049	6	7	0.952	6	0.698	1.614	6	0.228	3.849	1.202	-0.032	-0.508	1.156	0.749	1.012
2050	6	7	0.963	6	0.716	1.44	6	0.205	3.94	1.17	-0.078	-0.374	1.188	0.577	1.04
205	6	7	0.969	6	0.736	1.343	6	0.178	4.179	1.144	-0.129	-0.211	1.225	0.374	1.063

1															
205<br>2	7	7	0.942	6	0.482	1.74	4	0.332	2.485	0.004	-0.17	-0.011	0.817	0.059	0.044
205<br>3	7	7	0.968	6	0.532	1.36	4	0.298	2.517	0.19	-0.229	0.105	0.84	-0.042	-0.02
205<br>4	7	7	0.982	6	0.581	1.086	4	0.261	2.688	0.383	-0.274	0.201	0.874	-0.144	-0.044
205<br>5	7	7	0.987	6	0.622	0.951	4	0.228	2.961	0.568	-0.333	0.293	0.892	-0.245	-0.093
205<br>6	7	7	0.989	6	0.653	0.901	4	0.196	3.305	0.745	-0.389	0.347	0.902	-0.336	-0.127
205<br>7	7	7	0.989	6	0.674	0.919	4	0.171	3.66	0.904	-0.43	0.388	0.907	-0.412	-0.166
205<br>8	7	7	0.988	6	0.69	0.947	4	0.145	4.069	1.071	-0.454	0.403	0.922	-0.481	-0.161
205<br>9	7	7	0.987	6	0.698	0.964	4	0.125	4.4	1.211	-0.463	0.391	0.93	-0.518	-0.158
206<br>0	7	7	0.985	6	0.7	1.004	6	0.124	4.462	1.349	-0.472	0.37	0.934	-0.548	-0.154
206<br>1	7	7	0.986	6	0.705	1	6	0.126	4.435	1.476	-0.445	0.331	0.956	-0.558	-0.102
206<br>2	7	7	0.985	6	0.705	1.027	6	0.129	4.425	1.596	-0.428	0.29	0.968	-0.56	-0.071
206<br>3	7	7	0.984	6	0.706	1.053	6	0.131	4.417	1.697	-0.401	0.251	0.982	-0.552	-0.038
206	7	7	0.98	6	0.712	1.133	6	0.132	4.505	1.819	-0.352	0.213	1.019	-0.55	0.042

4															
2065	7	7	0.978	6	0.717	1.183	6	0.131	4.584	1.892	-0.326	0.193	1.043	-0.533	0.094
2066	7	7	0.973	6	0.725	1.282	6	0.129	4.735	1.974	-0.289	0.174	1.078	-0.52	0.169
2067	7	7	0.968	6	0.733	1.371	6	0.128	4.865	2.042	-0.259	0.151	1.113	-0.492	0.256
2068	7	7	0.963	6	0.738	1.444	6	0.128	4.948	2.101	-0.239	0.123	1.135	-0.456	0.321
2069	7	7	0.963	6	0.744	1.444	6	0.132	4.898	2.128	-0.217	0.074	1.151	-0.39	0.397
2070	7	7	0.964	6	0.745	1.425	6	0.143	4.731	2.145	-0.198	-0.005	1.154	-0.305	0.47
2071	7	7	0.97	6	0.745	1.331	6	0.156	4.455	2.122	-0.181	-0.091	1.147	-0.196	0.527
2072	7	7	0.97	6	0.735	1.33	6	0.181	4.127	2.094	-0.155	-0.225	1.135	-0.078	0.618
2073	7	7	0.973	6	0.723	1.269	6	0.209	3.755	1.976	-0.093	-0.347	1.131	0.062	0.713
2074	7	7	0.974	6	0.696	1.257	6	0.243	3.363	1.838	-0.049	-0.478	1.106	0.198	0.776
2075	7	7	0.973	6	0.662	1.272	6	0.276	3.021	1.639	0.006	-0.582	1.082	0.311	0.828
2076	7	7	0.963	6	0.625	1.451	6	0.309	2.859	1.445	0.066	-0.68	1.067	0.406	0.898
207	7	7	0.947	6	0.588	1.675	6	0.336	2.793	1.23	0.124	-0.746	1.053	0.461	0.956

7															
2078	7	7	0.927	6	0.552	1.916	6	0.358	2.782	1.007	0.169	-0.79	1.034	0.498	0.988
2079	7	7	0.898	6	0.516	2.227	6	0.376	2.858	0.791	0.208	-0.822	1.014	0.517	1.016
2080	7	7	0.864	6	0.484	2.542	6	0.383	3.011	0.575	0.241	-0.828	0.995	0.527	1.026
2081	7	7	0.833	6	0.455	2.807	6	0.39	3.115	0.399	0.243	-0.834	0.967	0.525	1.013
2082	7	7	0.799	6	0.426	3.081	6	0.39	3.261	0.223	0.239	-0.829	0.936	0.529	0.984
2083	7	7	0.771	6	0.409	3.296	6	0.383	3.431	0.091	0.231	-0.812	0.92	0.527	0.962
2084	7	7	0.744	6	0.393	3.499	6	0.371	3.614	-0.041	0.216	-0.784	0.9	0.529	0.921
2085	7	7	0.731	6	0.388	3.594	6	0.351	3.792	-0.133	0.201	-0.736	0.897	0.52	0.885
2086	7	7	0.728	6	0.388	3.62	6	0.322	3.992	-0.221	0.18	-0.658	0.898	0.501	0.832
2087	7	7	0.732	6	0.392	3.593	4	0.305	4.095	-0.286	0.156	-0.572	0.906	0.472	0.777
2088	7	7	0.747	6	0.403	3.478	4	0.337	3.84	-0.341	0.128	-0.433	0.925	0.415	0.704
2089	7	7	0.796	6	0.429	3.101	4	0.34	3.566	-0.284	0.102	-0.313	0.951	0.315	0.65
209	7	7	0.839	6	0.455	2.755	4	0.345	3.306	-0.23	0.068	-0.168	0.976	0.203	0.574

0															
2091	7	7	0.889	6	0.491	2.309	4	0.332	3.093	-0.101	0.033	-0.032	1.002	0.062	0.503
2092	7	7	0.922	6	0.524	1.98	4	0.32	2.968	0.02	-0.011	0.106	1.019	-0.077	0.411
2093	7	7	0.947	6	0.559	1.676	4	0.297	2.939	0.18	-0.062	0.223	1.023	-0.219	0.309
2094	7	7	0.96	6	0.593	1.487	4	0.272	3.05	0.344	-0.103	0.317	1.031	-0.345	0.23
2095	7	7	0.967	6	0.626	1.381	4	0.244	3.266	0.521	-0.147	0.398	1.033	-0.468	0.15
2096	7	7	0.972	6	0.653	1.3	4	0.215	3.525	0.697	-0.18	0.432	1.03	-0.556	0.094
2097	7	7	0.971	6	0.672	1.311	4	0.193	3.808	0.835	-0.213	0.47	1.03	-0.625	0.054
2098	7	7	0.97	6	0.684	1.33	4	0.175	4.061	0.96	-0.256	0.49	1.013	-0.676	-0.008
2099	7	7	0.967	6	0.694	1.377	4	0.159	4.331	1.062	-0.281	0.505	1.015	-0.717	-0.014
2100	7	7	0.963	6	0.701	1.443	4	0.144	4.613	1.162	-0.307	0.515	1.015	-0.756	-0.021
2101	7	7	0.958	6	0.704	1.518	4	0.137	4.792	1.209	-0.327	0.531	1.016	-0.784	-0.021
2102	7	7	0.953	6	0.707	1.598	4	0.128	5.013	1.264	-0.331	0.533	1.03	-0.818	0.02
210	7	7	0.947	6	0.708	1.669	4	0.127	5.112	1.273	-0.337	0.545	1.038	-0.842	0.042

3															
2104	7	7	0.945	6	0.707	1.698	4	0.124	5.181	1.281	-0.33	0.519	1.047	-0.86	0.102
2105	7	7	0.949	6	0.706	1.646	4	0.136	4.944	1.185	-0.281	0.498	1.067	-0.839	0.167
2106	7	7	0.955	6	0.695	1.572	4	0.151	4.629	1.072	-0.244	0.455	1.067	-0.804	0.206
2107	7	7	0.96	6	0.675	1.492	4	0.172	4.222	0.915	-0.177	0.386	1.072	-0.739	0.265
2108	7	7	0.961	6	0.648	1.48	4	0.196	3.875	0.754	-0.11	0.308	1.073	-0.668	0.324
2109	7	7	0.958	6	0.614	1.527	4	0.219	3.589	0.595	-0.042	0.213	1.069	-0.587	0.381
2110	7	7	0.948	6	0.579	1.663	4	0.243	3.404	0.439	0.029	0.119	1.067	-0.501	0.44
2111	7	7	0.933	6	0.545	1.849	4	0.261	3.324	0.309	0.089	0.018	1.061	-0.422	0.498
2112	7	7	0.909	6	0.519	2.118	4	0.274	3.395	0.198	0.167	-0.072	1.075	-0.339	0.586
2113	7	7	0.899	6	0.505	2.217	4	0.267	3.494	0.179	0.185	-0.168	1.067	-0.286	0.653
2114	7	7	0.886	6	0.493	2.338	4	0.257	3.639	0.167	0.207	-0.258	1.065	-0.229	0.725
2115	7	7	0.881	6	0.49	2.388	6	0.269	3.584	0.206	0.214	-0.341	1.064	-0.184	0.802
211	7	7	0.876	6	0.484	2.428	6	0.291	3.445	0.239	0.213	-0.418	1.057	-0.134	0.86

6															
2117	7	7	0.877	6	0.481	2.425	6	0.311	3.297	0.28	0.206	-0.483	1.046	-0.086	0.896
2118	7	7	0.881	6	0.478	2.385	6	0.325	3.153	0.317	0.191	-0.529	1.032	-0.047	0.911
2119	7	7	0.889	6	0.473	2.313	6	0.338	2.984	0.346	0.173	-0.569	1.012	-0.006	0.899
2120	7	7	0.903	6	0.474	2.174	6	0.341	2.835	0.372	0.144	-0.571	0.992	0.005	0.862
2121	7	7	0.909	6	0.476	2.114	6	0.338	2.799	0.378	0.146	-0.566	0.989	0.013	0.84
2122	7	7	0.916	6	0.482	2.038	6	0.331	2.79	0.384	0.146	-0.542	0.99	0.001	0.815
2123	7	7	0.92	6	0.482	1.992	6	0.319	2.815	0.347	0.143	-0.505	0.984	-0.009	0.767
2124	7	7	0.923	6	0.48	1.96	6	0.308	2.846	0.307	0.138	-0.47	0.977	-0.022	0.717
2125	7	7	0.915	6	0.474	2.052	6	0.299	2.976	0.236	0.148	-0.446	0.977	-0.018	0.695
2126	7	7	0.9	6	0.464	2.203	6	0.296	3.102	0.169	0.169	-0.45	0.978	0.006	0.698
2127	7	7	0.88	6	0.445	2.392	6	0.293	3.226	0.059	0.173	-0.457	0.959	0.053	0.663
2128	7	7	0.847	6	0.425	2.688	6	0.299	3.392	-0.036	0.201	-0.504	0.951	0.134	0.68
212	7	7	0.823	6	0.407	2.884	6	0.309	3.433	-0.097	0.212	-0.557	0.929	0.209	0.671

9															
2130	7	7	0.795	6	0.39	3.109	6	0.32	3.503	-0.154	0.226	-0.615	0.908	0.296	0.666
2131	7	7	0.797	6	0.384	3.096	6	0.329	3.405	-0.148	0.224	-0.652	0.885	0.357	0.632
2132	7	7	0.801	6	0.383	3.062	6	0.334	3.336	-0.135	0.225	-0.676	0.869	0.412	0.6
2133	7	7	0.837	6	0.398	2.771	6	0.335	3.119	-0.048	0.222	-0.671	0.866	0.431	0.562
2134	7	7	0.878	6	0.421	2.414	6	0.324	2.938	0.045	0.215	-0.634	0.869	0.439	0.506
2135	7	7	0.922	6	0.45	1.971	6	0.314	2.694	0.18	0.204	-0.593	0.874	0.431	0.443
2136	7	7	0.961	6	0.489	1.473	6	0.287	2.535	0.317	0.181	-0.501	0.881	0.404	0.341
2137	7	7	0.986	6	0.532	0.979	6	0.263	2.385	0.493	0.151	-0.408	0.886	0.357	0.227
2138	7	7	0.997	6	0.576	0.572	6	0.233	2.379	0.666	0.113	-0.292	0.891	0.298	0.092
2139	7	7	1	6	0.623	0.279	6	0.204	2.514	0.857	0.064	-0.163	0.896	0.218	-0.046
2140	7	7	1	6	0.665	0.149	6	0.175	2.817	1.048	0.015	-0.032	0.902	0.134	-0.185
2141	7	7	1	6	0.701	0.181	6	0.152	3.242	1.244	-0.05	0.09	0.899	0.047	-0.324
214	7	7	0.999	6	0.723	0.353	6	0.137	3.687	1.429	-0.118	0.174	0.88	-0.016	-0.464

2															
2143	7	7	0.995	6	0.735	0.664	6	0.123	4.24	1.622	-0.199	0.249	0.856	-0.078	-0.601
2144	7	7	0.988	6	0.727	0.941	6	0.126	4.45	1.801	-0.267	0.213	0.81	-0.073	-0.698
2145	7	7	0.985	6	0.721	1.029	6	0.129	4.468	1.886	-0.303	0.169	0.787	-0.019	-0.719
2146	7	7	0.984	6	0.711	1.05	6	0.144	4.241	1.968	-0.323	0.056	0.758	0.074	-0.697
2147	7	7	0.989	6	0.7	0.908	6	0.173	3.701	1.932	-0.318	-0.101	0.724	0.226	-0.642
2148	7	7	0.991	6	0.682	0.839	6	0.211	3.186	1.901	-0.297	-0.278	0.699	0.377	-0.547
2149	7	7	0.994	6	0.658	0.71	6	0.251	2.637	1.783	-0.233	-0.44	0.703	0.516	-0.387
2150	7	7	0.995	6	0.629	0.679	6	0.289	2.236	1.662	-0.173	-0.572	0.716	0.599	-0.221
2151	7	7	0.994	6	0.595	0.724	6	0.323	1.948	1.477	-0.098	-0.681	0.735	0.679	-0.054
2152	7	7	0.994	6	0.574	0.733	6	0.337	1.798	1.3	-0.023	-0.713	0.783	0.651	0.133
2153	7	7	0.989	6	0.549	0.893	6	0.345	1.823	1.038	0.06	-0.725	0.821	0.646	0.282
2154	7	7	0.974	6	0.522	1.249	6	0.348	2.062	0.782	0.15	-0.732	0.868	0.628	0.45
215	7	7	0.939	6	0.489	1.78	6	0.345	2.478	0.496	0.223	-0.729	0.895	0.634	0.559

5															
215 6	7	7	0.867	6	0.454	2.512	6	0.337	3.109	0.213	0.299	-0.727	0.924	0.645	0.671
215 7	7	7	0.771	6	0.416	3.296	6	0.324	3.792	-0.054	0.349	-0.724	0.932	0.679	0.723
215 8	7	7	0.654	6	0.38	4.17	4	0.305	4.609	-0.311	0.4	-0.703	0.945	0.724	0.764
215 9	7	4	0.55	6	0.353	4.953	7	0.344	5.002	-0.533	0.435	-0.685	0.949	0.765	0.78
216 0	7	4	0.513	6	0.407	5.245	7	0.313	5.772	-0.747	0.459	-0.622	0.953	0.823	0.75
216 1	7	4	0.531	6	0.425	5.101	7	0.303	5.776	-0.793	0.432	-0.59	0.927	0.832	0.666
216 2	7	4	0.55	6	0.444	4.955	7	0.301	5.729	-0.823	0.413	-0.535	0.916	0.841	0.592
216 3	7	4	0.608	6	0.443	4.51	7	0.319	5.166	-0.746	0.367	-0.481	0.894	0.825	0.486
216 4	7	4	0.659	6	0.437	4.134	7	0.342	4.623	-0.654	0.335	-0.422	0.887	0.808	0.406
216 5	7	4	0.72	6	0.417	3.682	7	0.374	3.9	-0.51	0.285	-0.37	0.868	0.788	0.302
216 6	7	7	0.774	6	0.405	3.275	4	0.392	3.339	-0.365	0.237	-0.33	0.848	0.785	0.201
216 7	7	7	0.847	6	0.439	2.688	4	0.363	3.066	-0.207	0.186	-0.295	0.827	0.785	0.103
216	7	7	0.898	6	0.47	2.225	4	0.328	2.947	-0.045	0.148	-0.295	0.806	0.812	0.029

8																
2169	7	7	0.922	6	0.497	1.972	4	0.303	2.959	0.065	0.128	-0.287	0.804	0.857	-0.006
2170	7	7	0.939	6	0.52	1.783	4	0.279	3.029	0.171	0.107	-0.294	0.797	0.914	-0.041
2171	7	7	0.946	6	0.54	1.688	4	0.263	3.13	0.243	0.099	-0.291	0.803	0.967	-0.052
2172	7	7	0.953	6	0.559	1.601	4	0.248	3.23	0.312	0.09	-0.285	0.808	1.013	-0.065
2173	7	7	0.961	6	0.579	1.484	4	0.236	3.282	0.372	0.086	-0.258	0.825	1.028	-0.063
2174	7	7	0.971	6	0.601	1.317	4	0.224	3.294	0.438	0.087	-0.214	0.854	1.013	-0.047
2175	7	7	0.981	6	0.627	1.119	4	0.21	3.305	0.515	0.095	-0.162	0.893	0.978	-0.014
2176	7	7	0.989	6	0.65	0.906	4	0.2	3.267	0.586	0.093	-0.091	0.93	0.91	0.008
2177	7	7	0.995	6	0.674	0.677	4	0.185	3.263	0.684	0.087	-0.034	0.961	0.831	0.016
2178	7	7	0.998	6	0.695	0.487	4	0.172	3.281	0.778	0.078	0.023	0.988	0.746	0.021
2179	7	7	0.999	6	0.71	0.333	4	0.16	3.317	0.871	0.054	0.059	0.996	0.671	-0.001
2180	7	7	1	6	0.723	0.215	4	0.148	3.385	0.962	0.029	0.088	1.002	0.6	-0.024
218	7	7	1	6	0.734	0.131	4	0.137	3.486	1.051	0.003	0.1	1.001	0.543	-0.046

1																
2182	7	7	1	6	0.739	0.08	4	0.129	3.563	1.124	-0.042	0.11	0.981	0.497	-0.111
2183	7	7	1	6	0.743	0.059	6	0.125	3.624	1.191	-0.087	0.117	0.96	0.458	-0.172
2184	7	7	1	6	0.748	0.062	6	0.124	3.659	1.263	-0.13	0.133	0.944	0.417	-0.232
2185	7	7	1	6	0.756	0.08	6	0.122	3.734	1.354	-0.166	0.152	0.938	0.368	-0.267
2186	7	7	1	6	0.763	0.128	6	0.118	3.87	1.449	-0.201	0.18	0.937	0.315	-0.301
2187	7	7	1	6	0.768	0.221	6	0.114	4.039	1.546	-0.245	0.206	0.925	0.27	-0.356
2188	7	7	0.999	6	0.772	0.332	6	0.11	4.231	1.653	-0.277	0.227	0.923	0.227	-0.387
2189	7	7	0.999	6	0.774	0.429	6	0.108	4.366	1.761	-0.292	0.222	0.927	0.213	-0.389
2190	7	7	0.998	6	0.772	0.519	6	0.112	4.384	1.866	-0.302	0.173	0.919	0.241	-0.386
2191	7	7	0.996	6	0.769	0.607	6	0.117	4.365	1.954	-0.304	0.109	0.91	0.293	-0.378
2192	7	7	0.994	6	0.757	0.726	6	0.137	4.146	2.024	-0.311	-0.037	0.866	0.423	-0.385
2193	7	7	0.992	6	0.749	0.811	6	0.159	3.904	2.028	-0.285	-0.184	0.838	0.586	-0.356
219	7	7	0.984	6	0.728	1.051	6	0.193	3.71	2.018	-0.268	-0.364	0.789	0.781	-0.348

4															
2195	7	7	0.969	6	0.702	1.355	6	0.229	3.592	1.972	-0.235	-0.537	0.75	0.974	-0.316
2196	7	7	0.938	6	0.669	1.784	6	0.27	3.599	1.923	-0.205	-0.707	0.71	1.158	-0.288
2197	7	7	0.904	6	0.637	2.161	6	0.306	3.629	1.84	-0.175	-0.835	0.675	1.305	-0.266
2198	7	7	0.879	6	0.612	2.408	6	0.333	3.626	1.758	-0.146	-0.921	0.655	1.392	-0.235
2199	7	7	0.857	6	0.588	2.601	6	0.355	3.613	1.653	-0.114	-0.983	0.64	1.464	-0.206
2200	7	7	0.885	6	0.587	2.353	6	0.352	3.375	1.564	-0.072	-0.96	0.667	1.415	-0.134
2201	7	7	0.917	6	0.59	2.026	6	0.34	3.13	1.445	-0.024	-0.902	0.7	1.346	-0.061
2202	7	7	0.954	6	0.603	1.576	6	0.316	2.866	1.331	0.024	-0.801	0.75	1.221	0.023
2203	7	7	0.981	6	0.62	1.108	6	0.284	2.67	1.214	0.067	-0.662	0.806	1.057	0.102
2204	7	7	0.993	6	0.635	0.757	6	0.251	2.616	1.098	0.109	-0.511	0.864	0.883	0.179
2205	7	7	0.997	6	0.649	0.529	6	0.219	2.705	1.01	0.144	-0.358	0.926	0.698	0.254
2206	7	7	0.999	6	0.659	0.432	6	0.188	2.936	0.916	0.167	-0.2	0.974	0.533	0.293
220	7	7	0.999	6	0.667	0.435	4	0.168	3.197	0.848	0.182	-0.05	1.023	0.364	0.33

7															
2208	7	7	0.998	6	0.675	0.505	4	0.187	3.07	0.784	0.187	0.114	1.063	0.236	0.321
2209	7	7	0.998	6	0.694	0.462	4	0.181	3.153	0.86	0.141	0.237	1.083	0.078	0.264
2210	7	7	0.998	6	0.716	0.498	4	0.173	3.337	0.944	0.096	0.364	1.104	-0.055	0.199
2211	7	7	0.998	6	0.744	0.486	4	0.148	3.721	1.133	0.031	0.437	1.106	-0.155	0.112
2212	7	7	0.997	6	0.766	0.557	4	0.125	4.184	1.319	-0.038	0.499	1.101	-0.23	0.01
2213	7	7	0.995	6	0.78	0.658	4	0.099	4.781	1.552	-0.114	0.513	1.076	-0.256	-0.107
2214	7	7	0.99	6	0.784	0.872	6	0.081	5.405	1.801	-0.168	0.505	1.066	-0.267	-0.174
2215	7	7	0.976	6	0.77	1.208	5	0.096	5.364	2.053	-0.227	0.486	1.043	-0.262	-0.263
2216	7	7	0.947	6	0.738	1.676	5	0.148	4.883	2.31	-0.274	0.45	1.029	-0.266	-0.314
2217	7	7	0.924	6	0.72	1.948	5	0.176	4.761	2.448	-0.286	0.433	1.024	-0.233	-0.347
2218	7	7	0.897	6	0.695	2.236	5	0.209	4.64	2.574	-0.306	0.403	1.008	-0.205	-0.388
2219	7	7	0.904	6	0.703	2.168	5	0.195	4.728	2.601	-0.289	0.332	0.996	-0.127	-0.376
222	7	7	0.911	6	0.709	2.088	5	0.18	4.826	2.619	-0.275	0.24	0.974	-0.041	-0.368

0																
2221	7	7	0.937	6	0.727	1.805	5	0.142	5.071	2.565	-0.247	0.11	0.943	0.083	-0.333
2222	7	7	0.954	6	0.734	1.585	6	0.126	5.114	2.507	-0.216	-0.035	0.913	0.199	-0.284
2223	7	7	0.967	6	0.732	1.385	6	0.156	4.478	2.421	-0.184	-0.186	0.877	0.327	-0.23
2224	7	7	0.974	6	0.719	1.248	6	0.186	3.954	2.328	-0.163	-0.307	0.844	0.412	-0.188
2225	7	7	0.978	6	0.703	1.17	6	0.213	3.56	2.25	-0.14	-0.407	0.826	0.47	-0.124
2226	7	7	0.982	6	0.688	1.096	6	0.235	3.25	2.174	-0.121	-0.474	0.816	0.495	-0.064
2227	7	7	0.987	6	0.682	0.962	6	0.241	3.041	2.099	-0.115	-0.477	0.818	0.469	-0.032
2228	7	7	0.991	6	0.682	0.833	6	0.242	2.908	2.036	-0.1	-0.46	0.837	0.422	0.022
2229	7	7	0.995	6	0.693	0.694	6	0.228	2.912	1.987	-0.084	-0.394	0.874	0.334	0.072
2230	7	7	0.997	6	0.703	0.558	6	0.213	2.944	1.93	-0.084	-0.313	0.898	0.249	0.081
2231	7	7	0.998	6	0.716	0.496	6	0.196	3.091	1.899	-0.075	-0.224	0.936	0.147	0.108
2232	7	7	0.998	6	0.725	0.441	6	0.183	3.188	1.861	-0.076	-0.159	0.954	0.094	0.102
223	7	7	0.998	6	0.726	0.483	6	0.179	3.28	1.889	-0.091	-0.134	0.956	0.047	0.088

3																
2234	7	7	0.998	6	0.722	0.507	6	0.181	3.272	1.91	-0.109	-0.141	0.942	0.043	0.061
2235	7	7	0.997	6	0.708	0.543	6	0.197	3.107	1.94	-0.136	-0.213	0.901	0.101	0.019
2236	7	7	0.996	6	0.692	0.625	6	0.216	2.95	1.978	-0.15	-0.307	0.866	0.177	0.002
2237	7	7	0.994	6	0.669	0.739	6	0.247	2.729	1.99	-0.155	-0.44	0.823	0.304	-0.003
2238	7	7	0.988	6	0.645	0.942	6	0.28	2.61	2.016	-0.142	-0.577	0.797	0.42	0.033
2239	7	7	0.978	6	0.622	1.174	6	0.314	2.54	2.007	-0.108	-0.712	0.788	0.553	0.101
2240	7	7	0.969	6	0.603	1.351	6	0.339	2.505	1.993	-0.086	-0.801	0.783	0.624	0.154
2241	7	7	0.967	6	0.595	1.387	6	0.35	2.45	1.893	-0.03	-0.848	0.81	0.695	0.24
2242	7	7	0.968	6	0.593	1.368	6	0.352	2.409	1.789	0.018	-0.86	0.841	0.724	0.317
2243	7	7	0.973	6	0.6	1.269	6	0.341	2.4	1.677	0.072	-0.826	0.891	0.699	0.406
2244	7	7	0.978	6	0.61	1.183	6	0.327	2.433	1.564	0.123	-0.777	0.943	0.656	0.491
2245	7	7	0.984	6	0.622	1.044	6	0.308	2.449	1.467	0.151	-0.702	0.985	0.573	0.541
224	7	7	0.99	6	0.631	0.864	6	0.288	2.432	1.355	0.158	-0.612	1.01	0.482	0.544

6															
2247	7	7	0.993	6	0.642	0.758	6	0.269	2.496	1.282	0.165	-0.522	1.041	0.37	0.562
2248	7	7	0.995	6	0.65	0.672	6	0.25	2.584	1.205	0.164	-0.428	1.064	0.262	0.56
2249	7	7	0.996	6	0.656	0.632	6	0.236	2.674	1.157	0.157	-0.35	1.079	0.156	0.553
2250	7	7	0.996	6	0.659	0.636	6	0.225	2.786	1.111	0.152	-0.282	1.093	0.061	0.549
2251	7	7	0.994	6	0.657	0.702	6	0.222	2.871	1.079	0.16	-0.257	1.104	0	0.571
2252	7	7	0.993	6	0.65	0.745	6	0.223	2.885	1.038	0.159	-0.244	1.102	-0.045	0.573
2253	7	7	0.992	6	0.636	0.795	6	0.233	2.8	0.994	0.164	-0.273	1.088	-0.049	0.579
2254	7	7	0.991	6	0.618	0.854	6	0.248	2.678	0.949	0.171	-0.32	1.07	-0.034	0.588
2255	7	7	0.989	6	0.594	0.9	6	0.267	2.5	0.889	0.172	-0.378	1.038	-0.002	0.576
2256	7	7	0.988	6	0.568	0.939	6	0.287	2.305	0.825	0.168	-0.44	0.998	0.046	0.547
2257	7	7	0.984	6	0.545	1.03	6	0.3	2.22	0.733	0.178	-0.485	0.969	0.104	0.527
2258	7	7	0.981	6	0.523	1.116	6	0.31	2.16	0.64	0.182	-0.519	0.936	0.164	0.488
225	7	7	0.977	6	0.51	1.195	6	0.306	2.219	0.54	0.191	-0.516	0.916	0.226	0.438

9															
226<br>0	7	7	0.972	6	0.501	1.288	6	0.296	2.34	0.446	0.203	-0.501	0.903	0.286	0.393
226<br>1	7	7	0.97	6	0.505	1.328	6	0.277	2.528	0.386	0.22	-0.458	0.908	0.334	0.354
226<br>2	7	7	0.968	6	0.513	1.358	6	0.252	2.779	0.336	0.242	-0.397	0.925	0.381	0.321
226<br>3	7	7	0.97	6	0.525	1.322	4	0.243	2.859	0.314	0.248	-0.332	0.934	0.419	0.263
226<br>4	7	7	0.972	6	0.546	1.297	4	0.248	2.874	0.318	0.28	-0.259	0.972	0.455	0.256
226<br>5	7	7	0.98	6	0.569	1.123	4	0.239	2.86	0.385	0.277	-0.208	0.987	0.471	0.211
226<br>6	7	7	0.987	6	0.592	0.974	4	0.229	2.87	0.452	0.275	-0.161	1.001	0.492	0.167
226<br>7	7	7	0.991	6	0.613	0.823	4	0.215	2.923	0.537	0.271	-0.139	1.01	0.508	0.137
226<br>8	7	7	0.995	6	0.631	0.696	4	0.201	2.987	0.619	0.265	-0.124	1.016	0.526	0.105
226<br>9	7	7	0.996	6	0.642	0.611	4	0.191	3.033	0.677	0.253	-0.123	1.01	0.545	0.069
227<br>0	7	7	0.997	6	0.649	0.537	4	0.184	3.057	0.726	0.233	-0.126	0.997	0.555	0.025
227<br>1	7	7	0.998	6	0.651	0.494	4	0.18	3.067	0.755	0.214	-0.129	0.982	0.567	-0.017
227	7	7	0.998	6	0.652	0.448	4	0.177	3.06	0.78	0.194	-0.131	0.969	0.554	-0.048

2															
2273	7	7	0.998	6	0.653	0.46	4	0.18	3.033	0.757	0.19	-0.11	0.976	0.547	-0.04
2274	7	7	0.998	6	0.653	0.469	4	0.185	2.99	0.731	0.182	-0.084	0.983	0.529	-0.035
2275	7	7	0.998	6	0.652	0.514	4	0.196	2.923	0.674	0.178	-0.032	0.999	0.504	-0.024
2276	7	7	0.997	6	0.652	0.57	4	0.207	2.867	0.618	0.173	0.026	1.017	0.473	-0.014
2277	7	7	0.995	6	0.651	0.651	4	0.22	2.825	0.559	0.172	0.103	1.042	0.431	-0.004
2278	7	7	0.993	6	0.652	0.753	4	0.232	2.817	0.504	0.173	0.19	1.072	0.385	0.008
2279	7	7	0.99	6	0.653	0.873	4	0.244	2.838	0.457	0.177	0.28	1.102	0.332	0.016
2280	7	7	0.985	6	0.659	1.013	4	0.251	2.942	0.428	0.197	0.374	1.151	0.286	0.054
2281	7	7	0.987	6	0.673	0.968	4	0.242	3.01	0.491	0.201	0.431	1.17	0.224	0.038
2282	7	7	0.988	6	0.691	0.938	4	0.23	3.142	0.567	0.22	0.481	1.201	0.172	0.05
2283	7	7	0.992	6	0.713	0.797	4	0.208	3.256	0.694	0.229	0.49	1.209	0.141	0.03
2284	7	7	0.995	6	0.735	0.686	4	0.186	3.438	0.831	0.251	0.487	1.225	0.118	0.035
228	7	7	0.997	6	0.758	0.585	4	0.161	3.684	0.981	0.288	0.452	1.246	0.126	0.067

5															
2286	7	7	0.998	6	0.774	0.498	4	0.14	3.911	1.121	0.317	0.399	1.251	0.153	0.078
2287	7	7	0.998	6	0.788	0.483	4	0.122	4.207	1.247	0.36	0.34	1.269	0.196	0.12
2288	7	7	0.998	6	0.797	0.492	4	0.107	4.5	1.364	0.398	0.261	1.276	0.244	0.157
2289	7	7	0.997	6	0.798	0.548	4	0.107	4.568	1.341	0.44	0.224	1.295	0.328	0.208
2290	7	7	0.996	6	0.796	0.617	4	0.107	4.624	1.311	0.478	0.182	1.309	0.404	0.254
2291	7	7	0.995	6	0.786	0.694	4	0.117	4.501	1.196	0.502	0.161	1.315	0.486	0.288
2292	7	7	0.991	6	0.776	0.836	4	0.127	4.457	1.086	0.533	0.135	1.328	0.56	0.341
2293	7	7	0.987	6	0.756	0.954	4	0.145	4.261	0.931	0.534	0.115	1.314	0.631	0.349
2294	7	7	0.979	6	0.735	1.146	4	0.164	4.147	0.778	0.541	0.092	1.306	0.692	0.374
2295	7	7	0.971	6	0.706	1.319	4	0.189	3.958	0.615	0.52	0.068	1.273	0.754	0.353
2296	7	7	0.956	6	0.678	1.548	4	0.216	3.836	0.457	0.505	0.062	1.251	0.787	0.347
2297	7	7	0.963	6	0.657	1.453	4	0.229	3.563	0.408	0.439	0.04	1.188	0.776	0.268
229	7	7	0.967	6	0.641	1.377	4	0.24	3.34	0.371	0.382	0.038	1.14	0.744	0.21

8																
2299	7	7	0.983	6	0.644	1.055	4	0.233	3.086	0.445	0.305	0.055	1.093	0.649	0.128
2300	7	7	0.992	6	0.646	0.785	4	0.228	2.866	0.516	0.219	0.09	1.042	0.546	0.025
2301	7	7	0.998	6	0.665	0.469	4	0.209	2.783	0.675	0.126	0.146	0.999	0.403	-0.091
2302	7	7	0.999	6	0.685	0.302	4	0.19	2.864	0.84	0.032	0.215	0.958	0.271	-0.22
2303	7	7	1	6	0.707	0.275	4	0.165	3.181	1.049	-0.065	0.285	0.919	0.124	-0.357
2304	7	7	0.999	6	0.728	0.407	4	0.14	3.706	1.27	-0.154	0.341	0.881	0.029	-0.496
2305	7	7	0.995	6	0.739	0.68	4	0.116	4.386	1.487	-0.239	0.383	0.847	-0.044	-0.628
2306	7	7	0.982	6	0.735	1.084	6	0.1	5.076	1.702	-0.325	0.405	0.803	-0.078	-0.771
2307	7	7	0.964	6	0.722	1.434	5	0.104	5.305	1.88	-0.388	0.391	0.767	-0.054	-0.873
2308	7	7	0.933	6	0.698	1.853	5	0.147	4.975	2.056	-0.449	0.36	0.727	-0.013	-0.972
2309	7	7	0.918	6	0.691	2.014	5	0.16	4.941	2.151	-0.469	0.308	0.706	0.079	-1.007
2310	7	7	0.907	6	0.685	2.133	5	0.17	4.924	2.249	-0.477	0.235	0.693	0.163	-1.005
231	7	7	0.914	6	0.699	2.059	5	0.15	5.139	2.256	-0.463	0.172	0.689	0.28	-0.988

1															
2312	7	7	0.924	6	0.706	1.951	5	0.132	5.302	2.251	-0.448	0.093	0.684	0.362	-0.947
2313	7	7	0.962	6	0.741	1.455	6	0.125	5.006	2.074	-0.372	0.054	0.714	0.483	-0.861
2314	7	7	0.984	6	0.754	1.046	6	0.134	4.506	1.892	-0.296	0.013	0.743	0.579	-0.765
2315	7	7	0.994	6	0.752	0.71	6	0.139	4.08	1.652	-0.217	-0.008	0.77	0.652	-0.68
2316	7	7	0.998	6	0.737	0.524	6	0.143	3.801	1.408	-0.142	-0.024	0.794	0.712	-0.6
2317	7	7	0.998	6	0.711	0.522	6	0.146	3.691	1.164	-0.102	-0.02	0.792	0.733	-0.582
2318	7	7	0.996	6	0.683	0.609	4	0.171	3.373	0.924	-0.065	0.022	0.8	0.718	-0.568
2319	7	7	0.993	6	0.651	0.767	4	0.21	3.032	0.72	-0.042	0.069	0.803	0.676	-0.564
2320	7	7	0.985	6	0.624	1.007	4	0.258	2.774	0.526	-0.029	0.209	0.826	0.565	-0.584
2321	7	7	0.977	6	0.608	1.207	4	0.29	2.69	0.433	-0.046	0.352	0.843	0.408	-0.618
2322	7	7	0.957	6	0.595	1.54	4	0.322	2.766	0.354	-0.06	0.543	0.879	0.22	-0.651
2323	7	7	0.929	6	0.598	1.897	4	0.334	3.061	0.356	-0.081	0.742	0.925	0.017	-0.683
232	7	7	0.884	6	0.606	2.357	4	0.34	3.515	0.373	-0.087	0.944	0.987	-0.181	-0.687

4															
2325	7	7	0.854	6	0.638	2.629	4	0.313	4.056	0.498	-0.09	1.095	1.052	-0.354	-0.667
2326	7	7	0.848	6	0.678	2.677	4	0.275	4.483	0.647	-0.058	1.182	1.13	-0.452	-0.584
2327	7	7	0.856	6	0.731	2.611	4	0.218	5.029	0.894	-0.031	1.221	1.203	-0.549	-0.488
2328	7	7	0.912	6	0.779	2.087	4	0.164	5.206	1.152	0.028	1.121	1.259	-0.493	-0.341
2329	7	7	0.948	6	0.822	1.664	4	0.109	5.699	1.499	0.087	0.959	1.309	-0.429	-0.159
2330	7	7	0.966	6	0.846	1.393	4	0.072	6.322	1.834	0.143	0.729	1.333	-0.295	0.01
2331	7	7	0.969	6	0.854	1.34	6	0.065	6.484	2.084	0.208	0.47	1.345	-0.113	0.192
2332	7	7	0.952	6	0.85	1.603	6	0.084	6.23	2.318	0.259	0.198	1.34	0.077	0.348
2333	7	7	0.936	6	0.841	1.82	6	0.107	5.938	2.37	0.324	-0.046	1.334	0.298	0.5
2334	7	7	0.907	6	0.82	2.138	6	0.136	5.732	2.397	0.363	-0.27	1.31	0.476	0.612
2335	7	7	0.884	6	0.8	2.358	6	0.161	5.562	2.263	0.435	-0.436	1.313	0.672	0.748
2336	7	7	0.874	6	0.762	2.447	6	0.199	5.128	2.079	0.459	-0.608	1.266	0.846	0.795
233	7	7	0.857	6	0.727	2.601	6	0.226	4.94	1.741	0.526	-0.711	1.251	1.037	0.867

7															
2338	7	7	0.829	6	0.691	2.839	6	0.245	4.91	1.391	0.573	-0.77	1.232	1.177	0.905
2339	7	7	0.826	6	0.662	2.859	6	0.25	4.805	1.08	0.577	-0.756	1.196	1.24	0.856
2340	7	7	0.81	6	0.635	2.989	6	0.245	4.897	0.775	0.581	-0.712	1.172	1.278	0.807
2341	7	7	0.857	6	0.625	2.603	6	0.228	4.623	0.627	0.526	-0.607	1.135	1.206	0.682
2342	7	7	0.897	6	0.622	2.229	6	0.199	4.512	0.492	0.471	-0.456	1.111	1.12	0.544
2343	7	7	0.954	6	0.629	1.584	4	0.193	3.951	0.496	0.367	-0.31	1.067	0.976	0.357
2344	7	7	0.981	6	0.647	1.113	4	0.202	3.444	0.532	0.284	-0.139	1.048	0.852	0.192
2345	7	7	0.998	6	0.683	0.495	4	0.174	3.235	0.77	0.155	-0.032	1.007	0.687	0.018
2346	7	7	1	6	0.72	0.166	4	0.144	3.385	1.025	0.047	0.057	0.98	0.552	-0.122
2347	7	7	1	6	0.747	0.077	6	0.129	3.59	1.299	-0.045	0.078	0.949	0.477	-0.231
2348	7	7	1	6	0.767	0.174	6	0.128	3.763	1.589	-0.114	0.075	0.932	0.418	-0.291
2349	7	7	0.999	6	0.777	0.362	6	0.129	3.955	1.827	-0.128	0.027	0.94	0.421	-0.27
235	7	7	0.995	6	0.777	0.664	6	0.134	4.174	2.062	-0.136	-0.051	0.945	0.434	-0.231

0																
235<br>1	7	7	0.988	6	0.776	0.942	6	0.14	4.366	2.216	-0.119	-0.129	0.956	0.484	-0.18
235<br>2	7	7	0.972	6	0.77	1.285	6	0.151	4.548	2.368	-0.094	-0.229	0.971	0.517	-0.097
235<br>3	7	7	0.967	6	0.77	1.38	6	0.158	4.547	2.381	-0.057	-0.291	0.985	0.582	-0.04
235<br>4	7	7	0.962	6	0.768	1.467	6	0.166	4.525	2.386	-0.026	-0.348	0.995	0.631	0.009
235<br>5	7	7	0.966	6	0.763	1.404	6	0.173	4.374	2.337	-0.027	-0.374	0.98	0.667	-0.01
235<br>6	7	7	0.971	6	0.757	1.319	6	0.179	4.203	2.279	-0.039	-0.391	0.956	0.698	-0.053
235<br>7	7	7	0.976	6	0.749	1.218	6	0.184	4.025	2.217	-0.079	-0.39	0.913	0.714	-0.144
235<br>8	7	7	0.98	6	0.742	1.133	6	0.187	3.89	2.149	-0.129	-0.375	0.865	0.723	-0.257
235<br>9	7	7	0.979	6	0.73	1.147	6	0.19	3.835	2.104	-0.2	-0.36	0.802	0.721	-0.397
236<br>0	7	7	0.978	6	0.726	1.181	6	0.187	3.895	2.076	-0.259	-0.32	0.759	0.701	-0.512
236<br>1	7	7	0.972	6	0.721	1.292	6	0.182	4.049	2.113	-0.309	-0.285	0.731	0.658	-0.589
236<br>2	7	7	0.965	6	0.717	1.421	6	0.172	4.27	2.155	-0.358	-0.235	0.708	0.607	-0.663
236	7	7	0.961	6	0.72	1.481	6	0.158	4.507	2.202	-0.375	-0.171	0.716	0.555	-0.685

3																
2364	7	7	0.959	6	0.725	1.514	6	0.144	4.753	2.267	-0.371	-0.107	0.746	0.499	-0.661
2365	7	7	0.962	6	0.741	1.455	6	0.129	4.954	2.317	-0.33	-0.043	0.803	0.459	-0.581
2366	7	7	0.964	6	0.757	1.426	6	0.119	5.131	2.373	-0.278	-0.005	0.863	0.441	-0.476
2367	7	7	0.967	6	0.781	1.373	6	0.109	5.317	2.414	-0.193	0.03	0.947	0.436	-0.318
2368	7	7	0.966	6	0.799	1.39	6	0.109	5.364	2.442	-0.106	-0.01	1.008	0.495	-0.155
2369	7	7	0.962	6	0.817	1.455	6	0.112	5.429	2.438	0.013	-0.072	1.088	0.582	0.059
2370	7	7	0.949	6	0.824	1.651	6	0.122	5.478	2.416	0.122	-0.179	1.141	0.707	0.251
2371	7	7	0.921	6	0.815	1.99	6	0.141	5.501	2.372	0.223	-0.336	1.172	0.855	0.435
2372	7	7	0.868	6	0.797	2.507	6	0.166	5.651	2.32	0.321	-0.505	1.196	1.006	0.614
2373	7	7	0.795	6	0.767	3.11	6	0.199	5.805	2.253	0.402	-0.685	1.205	1.133	0.777
2374	7	7	0.731	6	0.733	3.594	6	0.235	5.871	2.168	0.461	-0.832	1.203	1.21	0.9
2375	7	7	0.674	6	0.693	4.023	6	0.276	5.865	2.07	0.501	-0.97	1.187	1.269	0.993
237	7	7	0.684	6	0.666	3.942	6	0.3	5.535	1.958	0.511	-1.01	1.177	1.215	1.038

6																
2377	7	7	0.728	6	0.644	3.619	6	0.316	5.042	1.809	0.504	-1.009	1.16	1.14	1.038
2378	7	7	0.825	6	0.629	2.874	6	0.322	4.215	1.631	0.451	-0.947	1.116	1.021	0.939
2379	7	7	0.924	6	0.629	1.954	6	0.306	3.396	1.44	0.373	-0.81	1.071	0.867	0.778
2380	7	7	0.977	6	0.63	1.19	6	0.283	2.793	1.25	0.288	-0.65	1.025	0.704	0.597
2381	7	7	0.998	6	0.633	0.522	6	0.249	2.385	1.082	0.156	-0.449	0.951	0.54	0.311
2382	7	7	1	6	0.636	0.24	6	0.21	2.454	0.93	0.031	-0.232	0.887	0.403	0.023
2383	7	7	0.999	6	0.635	0.302	4	0.182	2.795	0.834	-0.113	-0.02	0.815	0.259	-0.292
2384	7	7	0.996	6	0.645	0.599	4	0.207	2.872	0.789	-0.214	0.195	0.783	0.176	-0.546
2385	7	7	0.986	6	0.668	0.981	4	0.191	3.488	0.97	-0.354	0.353	0.722	0.052	-0.805
2386	7	7	0.964	6	0.693	1.43	4	0.162	4.334	1.195	-0.449	0.504	0.703	-0.046	-0.978
2387	7	7	0.933	6	0.708	1.846	4	0.115	5.488	1.539	-0.508	0.593	0.716	-0.124	-1.064
2388	7	7	0.869	6	0.679	2.495	5	0.181	5.137	1.887	-0.563	0.667	0.729	-0.18	-1.144
238	7	7	0.818	6	0.639	2.927	5	0.262	4.709	2.239	-0.545	0.685	0.785	-0.18	-1.098

9															
2390	7	7	0.751	6	0.584	3.443	5	0.345	4.497	2.582	-0.521	0.643	0.829	-0.145	-1.032
2391	7	7	0.697	6	0.563	3.85	5	0.379	4.642	2.856	-0.461	0.591	0.887	-0.066	-0.929
2392	7	7	0.649	6	0.552	4.205	5	0.392	4.888	3.1	-0.401	0.437	0.915	0.042	-0.803
2393	7	7	0.712	6	0.65	3.736	5	0.283	5.397	3.079	-0.296	0.334	0.961	0.223	-0.654
2394	7	7	0.766	6	0.723	3.332	5	0.194	5.959	3.032	-0.207	0.195	0.983	0.401	-0.522
2395	7	7	0.853	6	0.785	2.634	5	0.113	6.513	2.843	-0.131	0.089	0.99	0.564	-0.411
2396	7	7	0.913	6	0.811	2.07	6	0.097	6.307	2.635	-0.074	-0.012	0.983	0.71	-0.332
2397	7	7	0.957	6	0.814	1.531	6	0.111	5.513	2.392	-0.049	-0.066	0.961	0.794	-0.289
2398	7	7	0.983	6	0.81	1.074	6	0.118	4.923	2.15	-0.031	-0.07	0.952	0.826	-0.254
2399	7	7	0.994	6	0.8	0.735	6	0.123	4.473	1.939	-0.031	-0.058	0.939	0.821	-0.227
2400	7	7	0.999	6	0.798	0.436	6	0.114	4.331	1.741	-0.04	0.052	0.949	0.746	-0.224
2401	7	7	0.999	6	0.798	0.319	6	0.107	4.34	1.706	-0.071	0.138	0.956	0.614	-0.232
240	7	7	1	6	0.802	0.279	6	0.094	4.564	1.687	-0.097	0.265	0.982	0.463	-0.233

2															
2403	7	7	0.999	6	0.813	0.409	6	0.082	4.991	1.792	-0.109	0.373	1.03	0.296	-0.199
2404	7	7	0.996	6	0.82	0.649	6	0.071	5.546	1.903	-0.117	0.481	1.083	0.14	-0.159
2405	7	7	0.984	6	0.825	1.035	6	0.063	6.189	2.085	-0.103	0.541	1.149	0.012	-0.073
2406	7	7	0.963	6	0.823	1.444	5	0.077	6.189	2.262	-0.085	0.553	1.202	-0.071	0.018
2407	7	7	0.926	6	0.815	1.933	5	0.097	6.188	2.451	-0.067	0.538	1.249	-0.134	0.11
2408	7	7	0.888	6	0.801	2.322	5	0.113	6.238	2.621	-0.052	0.443	1.266	-0.139	0.202
2409	7	7	0.859	6	0.793	2.58	5	0.119	6.37	2.727	-0.036	0.348	1.273	-0.121	0.278
2410	7	7	0.838	6	0.781	2.766	5	0.125	6.429	2.813	-0.035	0.227	1.256	-0.088	0.326
2411	7	7	0.85	6	0.774	2.661	5	0.12	6.394	2.803	-0.042	0.127	1.222	-0.051	0.33
2412	7	7	0.866	6	0.762	2.522	5	0.117	6.27	2.782	-0.062	0.031	1.175	-0.02	0.308
2413	7	7	0.907	6	0.765	2.134	6	0.117	5.89	2.662	-0.059	-0.036	1.14	0.021	0.294
2414	7	7	0.944	6	0.758	1.709	6	0.135	5.164	2.525	-0.076	-0.1	1.085	0.067	0.234
241	7	7	0.978	6	0.758	1.17	6	0.152	4.381	2.308	-0.067	-0.144	1.043	0.136	0.188

5															
2416	7	7	0.993	6	0.75	0.783	6	0.171	3.744	2.102	-0.043	-0.2	1.011	0.221	0.17
2417	7	7	0.999	6	0.736	0.381	6	0.184	3.158	1.795	0	-0.231	0.986	0.342	0.151
2418	7	7	1	6	0.712	0.214	6	0.195	2.802	1.489	0.045	-0.267	0.962	0.473	0.136
2419	7	7	1	6	0.681	0.247	6	0.203	2.667	1.19	0.073	-0.289	0.931	0.592	0.099
2420	7	7	0.998	6	0.641	0.486	6	0.207	2.745	0.883	0.091	-0.303	0.891	0.709	0.038
2421	7	7	0.992	6	0.601	0.799	6	0.208	2.922	0.636	0.072	-0.299	0.837	0.778	-0.057
2422	7	7	0.975	6	0.558	1.232	4	0.243	2.889	0.381	0.033	-0.249	0.778	0.817	-0.2
2423	7	7	0.944	6	0.514	1.713	4	0.297	2.81	0.183	-0.041	-0.194	0.7	0.821	-0.377
2424	7	7	0.905	6	0.484	2.155	4	0.356	2.766	0.015	-0.101	-0.055	0.664	0.761	-0.531
2425	7	7	0.887	6	0.476	2.33	4	0.379	2.786	0.007	-0.204	0.066	0.613	0.643	-0.713
2426	7	7	0.849	6	0.471	2.673	4	0.404	2.979	0.006	-0.312	0.23	0.568	0.505	-0.913
2427	7	7	0.847	6	0.501	2.691	4	0.386	3.21	0.146	-0.401	0.376	0.557	0.348	-1.051
242	7	7	0.837	6	0.535	2.77	4	0.361	3.56	0.302	-0.474	0.522	0.561	0.2	-1.16

8															
2429	7	7	0.852	6	0.592	2.643	4	0.301	3.991	0.566	-0.53	0.618	0.581	0.075	-1.223
2430	7	7	0.867	6	0.642	2.517	4	0.24	4.489	0.844	-0.566	0.678	0.607	-0.004	-1.251
2431	7	7	0.881	6	0.684	2.382	4	0.17	5.164	1.192	-0.587	0.703	0.647	-0.078	-1.239
2432	7	7	0.903	6	0.703	2.17	5	0.113	5.827	1.562	-0.572	0.667	0.702	-0.101	-1.15
2433	7	7	0.908	6	0.705	2.119	5	0.152	5.182	1.861	-0.54	0.635	0.764	-0.101	-1.052
2434	7	7	0.9	6	0.692	2.209	5	0.196	4.735	2.16	-0.5	0.584	0.827	-0.093	-0.933
2435	7	7	0.901	6	0.697	2.195	5	0.204	4.656	2.348	-0.437	0.531	0.896	-0.066	-0.785
2436	7	7	0.89	6	0.699	2.299	5	0.211	4.69	2.527	-0.38	0.468	0.958	-0.04	-0.642
2437	7	7	0.902	6	0.724	2.187	5	0.185	4.912	2.591	-0.313	0.396	1.01	0.009	-0.495
2438	7	7	0.91	6	0.745	2.099	5	0.158	5.197	2.644	-0.246	0.293	1.053	0.059	-0.336
2439	7	7	0.927	6	0.77	1.922	5	0.123	5.584	2.631	-0.181	0.192	1.085	0.126	-0.195
2440	7	7	0.943	6	0.779	1.723	6	0.101	5.81	2.585	-0.138	0.046	1.081	0.209	-0.088
244	7	7	0.963	6	0.781	1.45	6	0.124	5.139	2.478	-0.104	-0.088	1.06	0.31	-0.017

1															
2442	7	7	0.975	6	0.769	1.237	6	0.153	4.466	2.356	-0.083	-0.234	1.022	0.418	0.027
2443	7	7	0.985	6	0.747	1.012	6	0.182	3.835	2.194	-0.089	-0.345	0.964	0.521	0.01
2444	7	7	0.99	6	0.721	0.881	6	0.212	3.331	2.036	-0.094	-0.444	0.911	0.616	-0.008
2445	7	7	0.993	6	0.696	0.749	6	0.233	2.939	1.852	-0.137	-0.487	0.839	0.694	-0.108
2446	7	7	0.995	6	0.677	0.687	6	0.243	2.734	1.676	-0.184	-0.49	0.773	0.755	-0.226
2447	7	7	0.994	6	0.661	0.717	6	0.248	2.679	1.499	-0.244	-0.469	0.706	0.806	-0.363
2448	7	7	0.993	6	0.661	0.748	6	0.234	2.827	1.356	-0.281	-0.4	0.677	0.839	-0.465
2449	7	7	0.989	6	0.66	0.891	6	0.229	3.011	1.363	-0.359	-0.36	0.626	0.831	-0.589
2450	7	7	0.985	6	0.667	1.015	6	0.218	3.254	1.39	-0.421	-0.308	0.596	0.822	-0.684
2451	7	7	0.98	6	0.676	1.139	6	0.212	3.461	1.561	-0.477	-0.294	0.58	0.789	-0.724
2452	7	7	0.972	6	0.683	1.296	6	0.204	3.712	1.742	-0.521	-0.283	0.575	0.76	-0.739
2453	7	7	0.959	6	0.683	1.506	6	0.197	3.993	1.989	-0.545	-0.3	0.591	0.726	-0.692
245	7	7	0.937	6	0.673	1.799	6	0.189	4.341	2.246	-0.553	-0.329	0.621	0.686	-0.603

4																
2455	7	7	0.9	6	0.656	2.207	6	0.179	4.807	2.501	-0.549	-0.363	0.661	0.65	-0.489
2456	7	7	0.84	6	0.621	2.744	5	0.192	5.097	2.738	-0.557	-0.4	0.689	0.591	-0.39
2457	7	7	0.851	6	0.643	2.657	5	0.176	5.244	2.764	-0.509	-0.385	0.753	0.587	-0.255
2458	7	7	0.859	6	0.659	2.582	5	0.167	5.328	2.772	-0.479	-0.366	0.801	0.57	-0.151
2459	7	7	0.912	6	0.699	2.086	6	0.155	5.094	2.626	-0.433	-0.315	0.846	0.586	-0.077
2460	7	7	0.95	6	0.729	1.639	6	0.152	4.773	2.469	-0.399	-0.264	0.879	0.602	-0.028
2461	7	7	0.981	6	0.754	1.112	6	0.15	4.344	2.229	-0.374	-0.205	0.886	0.649	-0.04
2462	7	7	0.994	6	0.768	0.728	6	0.147	4.038	1.991	-0.346	-0.155	0.893	0.708	-0.05
2463	7	7	0.998	6	0.773	0.472	6	0.143	3.846	1.744	-0.309	-0.112	0.897	0.778	-0.058
2464	7	7	0.999	6	0.768	0.382	6	0.139	3.804	1.487	-0.287	-0.07	0.885	0.869	-0.106
2465	7	7	0.999	6	0.759	0.409	6	0.138	3.814	1.361	-0.289	-0.053	0.86	0.913	-0.156
2466	7	7	0.998	6	0.751	0.452	6	0.135	3.88	1.245	-0.279	-0.028	0.848	0.949	-0.183
246	7	7	0.998	6	0.748	0.445	6	0.132	3.921	1.218	-0.302	0.012	0.83	0.92	-0.232

7															
2468	7	7	0.998	6	0.745	0.459	6	0.126	4.006	1.183	-0.336	0.067	0.806	0.88	-0.306
2469	7	7	0.999	6	0.748	0.43	4	0.119	4.111	1.204	-0.381	0.15	0.795	0.777	-0.374
2470	7	7	0.998	6	0.747	0.511	4	0.124	4.112	1.213	-0.447	0.255	0.771	0.666	-0.488
2471	7	7	0.995	6	0.746	0.683	4	0.126	4.237	1.236	-0.52	0.371	0.751	0.532	-0.608
2472	7	7	0.987	6	0.742	0.976	4	0.128	4.491	1.261	-0.593	0.478	0.725	0.429	-0.74
2473	7	7	0.974	6	0.738	1.261	4	0.128	4.765	1.281	-0.65	0.567	0.711	0.355	-0.837
2474	7	7	0.961	6	0.736	1.479	4	0.124	5.034	1.315	-0.687	0.627	0.706	0.319	-0.902
2475	7	7	0.962	6	0.743	1.457	4	0.117	5.161	1.364	-0.689	0.606	0.708	0.374	-0.897
2476	7	7	0.969	6	0.752	1.355	4	0.107	5.253	1.422	-0.677	0.556	0.715	0.46	-0.864
2477	7	7	0.984	6	0.769	1.046	4	0.093	5.282	1.511	-0.618	0.421	0.742	0.615	-0.724
2478	7	7	0.993	6	0.787	0.757	6	0.094	5.002	1.622	-0.525	0.258	0.796	0.768	-0.499
2479	7	7	0.994	6	0.799	0.719	6	0.109	4.701	1.753	-0.415	0.065	0.861	0.936	-0.226
248	7	7	0.989	6	0.806	0.893	6	0.123	4.659	1.89	-0.292	-0.098	0.954	1.009	0.106

0															
2481	7	7	0.974	6	0.815	1.264	6	0.129	4.952	1.933	-0.141	-0.201	1.076	1.056	0.462
2482	7	7	0.937	6	0.825	1.803	6	0.128	5.538	1.972	-0.001	-0.24	1.212	1.004	0.815
2483	7	7	0.897	6	0.832	2.234	6	0.122	6.07	1.872	0.123	-0.218	1.331	0.925	1.093
2484	7	7	0.836	6	0.836	2.777	6	0.118	6.699	1.758	0.232	-0.189	1.436	0.834	1.342
2485	7	7	0.81	6	0.826	2.994	6	0.12	6.852	1.544	0.295	-0.176	1.475	0.792	1.458
2486	7	7	0.836	6	0.807	2.775	6	0.123	6.534	1.282	0.294	-0.145	1.453	0.777	1.422
2487	7	7	0.871	6	0.776	2.48	6	0.129	6.075	1.003	0.266	-0.122	1.396	0.778	1.318
2488	7	7	0.919	6	0.744	2.003	4	0.134	5.427	0.707	0.188	-0.014	1.313	0.782	1.078
2489	7	7	0.97	6	0.72	1.329	4	0.161	4.33	0.611	0.035	0.077	1.19	0.723	0.764
2490	7	7	0.988	6	0.701	0.939	4	0.191	3.535	0.525	-0.124	0.222	1.076	0.656	0.42
2491	7	7	0.995	6	0.709	0.666	4	0.194	3.262	0.611	-0.296	0.379	0.983	0.55	0.095
2492	7	7	0.993	6	0.718	0.758	4	0.193	3.389	0.708	-0.461	0.549	0.9	0.449	-0.221
249	7	7	0.985	6	0.74	1.008	4	0.168	3.971	0.911	-0.602	0.705	0.852	0.342	-0.467

3															
2494	7	7	0.967	6	0.753	1.384	4	0.138	4.783	1.141	-0.71	0.836	0.832	0.251	-0.639
2495	7	7	0.93	6	0.745	1.881	5	0.108	5.749	1.4	-0.797	0.955	0.832	0.163	-0.765
2496	7	7	0.898	6	0.715	2.22	5	0.178	5.005	1.686	-0.837	1.008	0.861	0.103	-0.779
2497	7	7	0.866	6	0.685	2.519	5	0.233	4.678	1.891	-0.844	1.061	0.906	0.074	-0.759
2498	7	7	0.838	6	0.65	2.761	5	0.286	4.402	2.1	-0.836	1.078	0.955	0.056	-0.698
2499	7	7	0.824	6	0.63	2.881	5	0.317	4.25	2.255	-0.816	1.063	0.998	0.057	-0.618
2500	7	7	0.808	6	0.61	3.01	5	0.345	4.147	2.411	-0.79	1.033	1.042	0.059	-0.52
2501	7	7	0.801	6	0.598	3.063	5	0.361	4.074	2.526	-0.767	0.978	1.068	0.07	-0.429
2502	7	7	0.792	6	0.58	3.131	5	0.381	3.974	2.622	-0.761	0.911	1.076	0.081	-0.367
2503	7	7	0.787	6	0.564	3.169	5	0.397	3.872	2.691	-0.764	0.841	1.068	0.1	-0.328
2504	7	7	0.781	6	0.539	3.217	5	0.423	3.704	2.74	-0.785	0.77	1.043	0.106	-0.321
2505	7	7	0.814	6	0.566	2.961	5	0.391	3.7	2.674	-0.785	0.736	1.028	0.139	-0.318
250	7	7	0.84	6	0.583	2.742	5	0.369	3.661	2.598	-0.797	0.712	1.003	0.161	-0.342

6																
2507	7	7	0.879	6	0.631	2.407	5	0.315	3.797	2.465	-0.782	0.729	1.007	0.174	-0.335
2508	7	7	0.907	6	0.668	2.137	5	0.271	3.943	2.325	-0.778	0.757	1.003	0.182	-0.351
2509	7	7	0.929	6	0.709	1.896	5	0.222	4.214	2.174	-0.757	0.808	1.019	0.177	-0.342
2510	7	7	0.947	6	0.751	1.674	5	0.173	4.608	2.05	-0.704	0.856	1.067	0.166	-0.264
2511	7	7	0.957	6	0.783	1.534	5	0.137	5.023	1.94	-0.654	0.904	1.114	0.149	-0.189
2512	7	7	0.966	6	0.82	1.391	5	0.097	5.664	1.868	-0.552	0.919	1.204	0.142	0.002
2513	7	7	0.965	6	0.835	1.411	5	0.084	6.015	1.89	-0.48	0.897	1.27	0.115	0.174
2514	7	7	0.958	6	0.852	1.516	5	0.068	6.562	1.923	-0.389	0.849	1.349	0.098	0.392
2515	7	7	0.945	6	0.863	1.697	5	0.058	7.084	1.972	-0.31	0.768	1.409	0.087	0.602
2516	7	7	0.927	6	0.87	1.924	5	0.05	7.626	2.009	-0.244	0.68	1.455	0.083	0.787
2517	7	7	0.914	6	0.873	2.058	6	0.049	7.839	1.99	-0.195	0.594	1.479	0.09	0.93
2518	7	7	0.92	6	0.869	1.994	6	0.055	7.524	1.937	-0.189	0.527	1.464	0.09	0.978
251	7	7	0.938	6	0.863	1.794	6	0.061	7.088	1.814	-0.192	0.479	1.438	0.106	0.989

9															
2520	7	7	0.968	6	0.849	1.361	6	0.066	6.477	1.65	-0.257	0.487	1.365	0.098	0.862
2521	7	7	0.986	6	0.832	0.984	4	0.07	5.925	1.465	-0.335	0.517	1.28	0.089	0.694
2522	7	7	0.994	6	0.807	0.74	4	0.093	5.068	1.263	-0.442	0.58	1.176	0.07	0.459
2523	7	7	0.993	6	0.779	0.747	4	0.114	4.588	1.132	-0.578	0.661	1.059	0.03	0.175
2524	7	7	0.983	6	0.746	1.057	4	0.14	4.402	1	-0.72	0.754	0.939	-0.008	-0.127
2525	7	7	0.952	6	0.712	1.612	4	0.152	4.703	0.972	-0.875	0.838	0.821	-0.057	-0.432
2526	7	7	0.896	6	0.681	2.244	4	0.156	5.184	0.972	-0.999	0.907	0.727	-0.082	-0.681
2527	7	7	0.803	6	0.633	3.048	5	0.17	5.683	1.048	-1.141	0.958	0.628	-0.12	-0.939
2528	7	7	0.755	6	0.596	3.418	5	0.232	5.308	1.171	-1.218	0.947	0.572	-0.101	-1.067
2529	7	7	0.719	6	0.567	3.688	5	0.278	5.114	1.261	-1.275	0.92	0.53	-0.052	-1.164
2530	7	7	0.701	6	0.544	3.821	5	0.315	4.916	1.355	-1.317	0.854	0.489	0.031	-1.233
2531	7	7	0.761	6	0.582	3.375	5	0.27	4.912	1.37	-1.28	0.754	0.496	0.167	-1.173
253	7	7	0.82	6	0.619	2.911	5	0.224	4.944	1.387	-1.236	0.637	0.506	0.308	-1.092

2															
253 3	7	7	0.897	6	0.678	2.236	5	0.14	5.397	1.305	-1.135	0.514	0.545	0.478	-0.934
253 4	7	7	0.945	6	0.711	1.706	4	0.108	5.471	1.218	-1.035	0.398	0.588	0.616	-0.766
253 5	7	7	0.975	6	0.731	1.238	4	0.121	4.826	1.066	-0.894	0.296	0.657	0.766	-0.545
253 6	7	7	0.984	6	0.728	1.052	4	0.139	4.361	0.892	-0.782	0.246	0.717	0.839	-0.369
253 7	7	7	0.985	6	0.716	1.022	4	0.16	4.019	0.716	-0.672	0.219	0.775	0.888	-0.197
253 8	7	7	0.979	6	0.697	1.15	4	0.187	3.778	0.529	-0.579	0.229	0.828	0.897	-0.057
253 9	7	7	0.971	6	0.675	1.316	4	0.215	3.601	0.386	-0.544	0.277	0.848	0.845	-0.002
254 0	7	7	0.955	6	0.649	1.561	4	0.25	3.471	0.237	-0.521	0.343	0.863	0.78	0.029
254 1	7	7	0.939	6	0.625	1.772	4	0.281	3.371	0.141	-0.553	0.428	0.845	0.674	-0.021
254 2	7	7	0.906	6	0.592	2.148	4	0.322	3.364	0.027	-0.611	0.528	0.803	0.576	-0.135
254 3	7	7	0.856	6	0.559	2.606	4	0.36	3.483	-0.054	-0.706	0.626	0.737	0.465	-0.3
254 4	7	7	0.791	6	0.53	3.144	4	0.395	3.731	-0.12	-0.79	0.732	0.683	0.369	-0.455
254	7	7	0.715	6	0.507	3.714	4	0.423	4.079	-0.159	-0.876	0.832	0.631	0.271	-0.612

5															
254<br>6	7	7	0.629	6	0.487	4.354	4	0.447	4.524	-0.19	-0.955	0.936	0.584	0.182	-0.763
254<br>7	7	7	0.566	6	0.48	4.828	4	0.458	4.921	-0.184	-1.003	1.046	0.569	0.096	-0.862
254<br>8	7	7	0.501	6	0.474	5.34	4	0.466	5.374	-0.174	-1.048	1.159	0.559	0.015	-0.957
254<br>9	7	7	0.472	6	0.488	5.575	4	0.451	5.733	-0.094	-1.076	1.271	0.571	-0.077	-1.006
255<br>0	7	7	0.456	6	0.507	5.716	4	0.427	6.059	0.003	-1.087	1.383	0.6	-0.159	-1.025
255<br>1	7	7	0.443	6	0.534	5.828	4	0.387	6.468	0.154	-1.111	1.487	0.624	-0.254	-1.05
255<br>2	7	7	0.449	6	0.563	5.771	4	0.339	6.782	0.322	-1.115	1.572	0.66	-0.318	-1.04
255<br>3	7	7	0.464	6	0.585	5.645	4	0.257	7.289	0.605	-1.16	1.613	0.679	-0.403	-1.048
255<br>4	7	7	0.473	6	0.572	5.574	5	0.225	7.436	0.89	-1.2	1.634	0.695	-0.464	-1.05
255<br>5	7	7	0.486	6	0.512	5.465	5	0.357	6.186	1.207	-1.25	1.598	0.697	-0.49	-1.054
255<br>6	7	5	0.53	6	0.504	5.107	7	0.417	5.485	1.522	-1.298	1.545	0.695	-0.501	-1.055
255<br>7	7	5	0.656	6	0.623	4.152	7	0.33	5.424	1.823	-1.332	1.449	0.693	-0.475	-1.031
